# Surface Density of Mono- and Trivalent High-Mannan-Derived Targeting Structures with Different Affinities Impacts Cellular Uptake of Human Serum Albumin-Derived Nanocarriers

**DOI:** 10.1021/acs.biomac.5c01510

**Published:** 2025-10-07

**Authors:** Robert Forster, Bellinda Lantzberg, Annabelle Weldert, Laura Rosenberger, Yanira Zeyn, Danuta Kowalczyk, Seah Ling Kuan, Christian Kersten, Matthias Bros, Tanja Weil, Tanja Schirmeister, Till Opatz

**Affiliations:** † Department of Chemistry, Johannes Gutenberg-University Mainz, Duesbergweg 10-14, 55128 Mainz, Germany; ‡ 28308 Max Planck Institute for Polymer Research, Ackermannweg 10, 55128 Mainz, Germany; § Department for Pharmaceutical and Biomedical Sciences, Johannes Gutenberg-University Mainz, Staudingerweg 5, 55128 Mainz, Germany; ∥ Department of Dermatology, University Medical Center of the Johannes Gutenberg-University Mainz, Obere Zahlbacher Straße 63, 55131 Mainz, Germany

## Abstract

Actively targeted delivery of nanocarriers (NC) modified with targeting structures (TS) binding to cell surface receptors, specific to target cells, enables enhanced selectivity and efficacy of cellular uptake. This is influenced by the ligand density on the NC surface. Herein, the impact of type, valency, and surface density of high-mannan derived TS on the C-type lectin receptor (CLR)-mediated uptake of human serum albumin (HSA)-based NCs in immune cell populations was investigated. Monovalent and trivalent TSs were prepared via efficient synthesis protocols and investigated regarding their affinity versus isolated carbohydrate recognition domains (CRD) of CD206 and CD209 within a NanoDSF study. Conjugation to HSA resulted in low valency and saturated NCs with a well-defined mannose epitope count. An *in vitro* study with bone-marrow-derived dendritic cells and splenic immune cells revealed the impact of the NC surface modification on cellular uptake and cell selectivity, allowing insights into the design of TSs and NCs.

## Graphical abstract

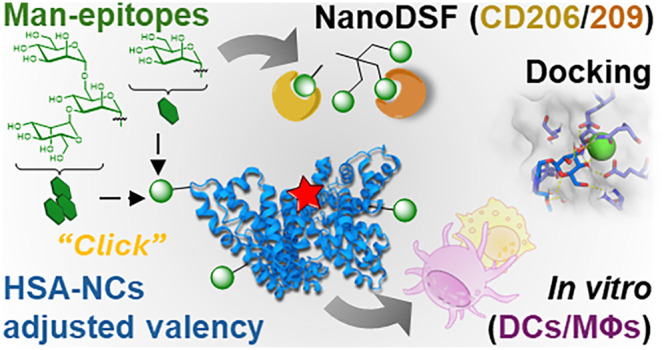

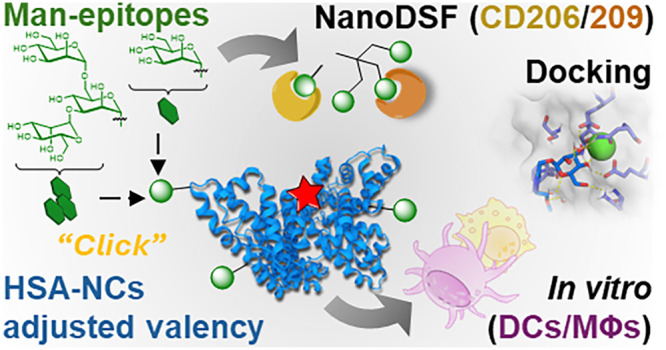

## 1 Introduction

Actively targeted drug delivery in immunotherapy aims at modulating immune responses by using drug-loaded nanoparticulate carriers (NCs) functionalized with targeting structures (TS).^1–4^ Such polymer-, lipid-, or protein-based NCs protect the cargo, improve biodistribution and therapeutic efficacy.^5–9^ Mannose-based TS enable selective delivery to antigen-presenting cells (APC) like dendritic cells (DC) or macrophages (MΦ) mediated by C-type lectin receptors (CLR).^10,11^ CLR engagement allows internalization and intracellular distribution of NCs prior to the release and processing, as well as presentation of NC-derived antigens and, in the case of sufficient APC activation, downstream T-cell responses. This makes them attractive targets for APC-specific therapies (e.g., tumor immunotherapy).^12–18^ Targeting of DCs or M2-polarized MΦs focuses on highly expressed CLRs like CD206 (MMR-1) and CD209 (DC-SIGN), due to involvement of either CLR in antigen internalization and subsequent presentation via major histocompatibility complex (MHC) molecules for T-cell activation.^19–29^ CD206 contains eight carbohydrate recognition domains (CRD), with CRD4 being responsible for most of the binding activity.^30–33^ CD209 contains one CRD and forms a homotetramer (Figure 1A).^34–36^ Glycan array studies revealed binding of epitopes of a nonamannose substructure from naturally occurring high-mannan-type glycans,^37–44^ with CD206 preferring shorter, terminally α-(1→2)-linked and CD209 internal α-(1→2)- and α-(1→3)-linked epitopes with canonical binding modes engaging hydroxyl groups 3 and 4 of the mannose units (Man).^45–49^ Increased affinity for the α-(1→3)- and α-(1→6)-branched trisaccharide (Man_3_) epitopes results from secondary contacts (Figure 1A).^31^ CLR targeting thus strongly depends on epitope geometry.^50–52^ Singular binding interactions of isolated epitopes and CRDs provide low intrinsic affinity and specificity (*K*_D_ in the mM range).^53–56^ Presentation of multiple CRDs and formation of oligomers and cell surface clusters by CLRs offer extended binding regions^57–59^ and enable multivalent interactions with multimerized epitopes for high functional affinity (avidity) and specificity (*K*_D_ in the μM to pM range) due to avidity effects such as clustering, chelation, and statistical rebinding (compare Figure 1C for avidity effects of potential relevance for the present study). Clustering refers to the ability of multivalent ligands to simultaneously bind to multiple receptors, while chelation enables binding to oligomeric receptors via simultaneous occupation of multiple binding sites. Statistical rebinding refers to the ability of a multivalent ligand to reform individual ligand–receptor bonds upon dissociation using adjacent epitopes, resulting in an increased probability of binding interactions. Acting as an effective increase in local epitope concentration, this leads to a higher apparent binding affinity.^60–70^ Similar considerations apply for engagement of CLRs with multivalent NCs presenting mannose TSs, as biodistribution and cellular uptake are markedly influenced not only by size, surface charge and valency of the NC, but also by surface density, conjugation strategy, and orientation of the TSs.^71–82^ Formation of TS-CLR complexes (Figure 1B) resulting from multivalent interactions between NCs, presenting multimerized TSs, and cell surfaces, expressing multiple CLRs can be described in thermodynamic terms by the Gibbs free energy, Δ*G*, which is related to avidity of the system via the dissociation constant, *K*_D_ (Δ*G* = *RT* ln­(*K*_D_)).^81,83^ Models describing Δ*G* in terms of multivalent binding interactions highlight the number of formed complexes as the main driving force, with each contributing favorable binding enthalpy Δ*H* to overcompensate the unfavorable decrease in entropy Δ*S* upon binding (Δ*G* = Δ*H* – *T*Δ*S*).^64,84^ Negative values of Δ*G* describe the equilibrium of the binding process to favor the TS-CRD complex (Δ*G* < 0 kJ/mol → *K*_D_ < 1 M). The entropy loss, Δ*S*, results from the mobility restriction for TSs entering the bound state. Being smaller for TSs already constrained by high surface density or conjugation via rigid linkers, the usage of flexible linkers, which offer the mobility required for adaptation to the target and efficient binding, imparts higher entropic penalties.^85–87^ Based on thermodynamic considerations, targeting efficacy of multivalent NCs is assumed to benefit from high TS numbers (TS valency) and CLR expression (receptor availability).^81^ On the contrary, targeting efficacy is hampered on the NC level by its size increase inherent to higher surface modification and further impacted by its shape and surface charge.^88^ On the cellular level, targeting efficacy is decreased by steric hindrance of closely packed TSs (TS overcrowding), higher consumption of cell membrane receptors per NC binding event (receptor depletion) and slow recycling kinetics of such receptors (receptor recycling).^89^ Thus, cellular uptake follows two general trends depending on TSs density: (a) “optimum density with a plateau” and (b) “optimum density with a maximum” (Figure 1D). The underlying mechanisms governing these trends have been comprehensively analyzed by Alkilany et al.^76^ Steric hindrance of TSs on NCs with saturated surface also depends on the conjugation strategy employed,^90^ with nonoriented TSs experiencing reduced steric hindrance resulting in trend (a) and with oriented, closely packed ligands experiencing overcrowding effects resulting in trend (b).^76^ Ligand density furthermore influences the operating uptake mechanism responsible for internalization of NCs.^91–93^ Thus, avidity of NCs represents not a simple “additive” phenomenon summing up intrinsic affinities of TS,^94^ as already formed binding interactions influence subsequent formations (Figure 1B), either by positive (first favors second interaction) or negative cooperativity (first hinders second interaction) (TS cooperativity), with avidity effects falling into the first category.^81,83,95^ This complex interplay determining biodistribution and cellular uptake of targeted NCs is sensitive to small changes in NC valency and TS surface density with the major contributors summarized in Figure 1D. Chung et al. demonstrated low-valency human serum albumin (HSA)-derived NCs with six Man TSs achieving high uptake in lung metastases via selective CD206-mediated targeting of tumor-associated MΦs, while slightly higher valency of eight TSs shifted accumulation to the liver.^96^ Such investigations of targeted delivery approaches are often directed at systemic distribution with limited focus on interactions of multivalent NCs at the cellular level.^81^ The latter are often studied in experiments using surface plasmon resonance (SPR)^63,97,98^ on artificial or isolated membranes,^99–101^ to model the disposition of natural cellular membranes.^81^ Given the relevance of low-valency NCs and limited understanding of multivalent interactions occurring at the cellular level, the impact of TS surface density and NC valency on cellular uptake, being a crucial step for targeted delivery approaches, needs to be investigated.^76^ Heading out from reported HSA-NCs presenting monovalent TSs with the Man_3_-epitope for CLR targeted transport of a toll-like receptor (TLR) 7/8 agonist for APC selective uptake,^102^ we used similar systems to present quantifiable amounts of well-defined monovalent and trivalent TSs with Man and Man_3_ epitopes at low and high degree of functionalization for this study. HSA as basis of NCs enables surface modification with controlled stoichiometry.^103^ Quantification of the number of TS per NC was a prerequisite to investigate which effect (i) TS surface density and NC valency, (ii) epitope type, and (iii) epitope clustering have on targeting properties. First, a docking study for both epitopes allowed insights into TS conformation and potential epitope binding modes (sections 3.1 and 3.3). Then, a NanoDSF study revealed binding affinities of all unconjugated TSs for isolated CRDs of CD206 and CD209 (section 3.2). Furthermore, an *in vitro* study of bone marrow-derived DCs (BMDC) allowed the investigation of cellular uptake of TS-HSA conjugates with low degree of functionalization (low valency) and high degree of functionalization (saturated valency) under consideration of influences (i)–(iii) (compare Figure 1D). Cellular uptake levels served as a measure of binding strength to determine the cell targeting ability of NCs within a biologically relevant environment and enabled interpretation in the context of the discussed trends (a) and (b). Finally, an *in vitro* study of splenic immune cells enabled comparisons within a more heterogeneous cell population and indications about APC targeting selectivity in the presence of off-target immune cells.

**Figure 1:**
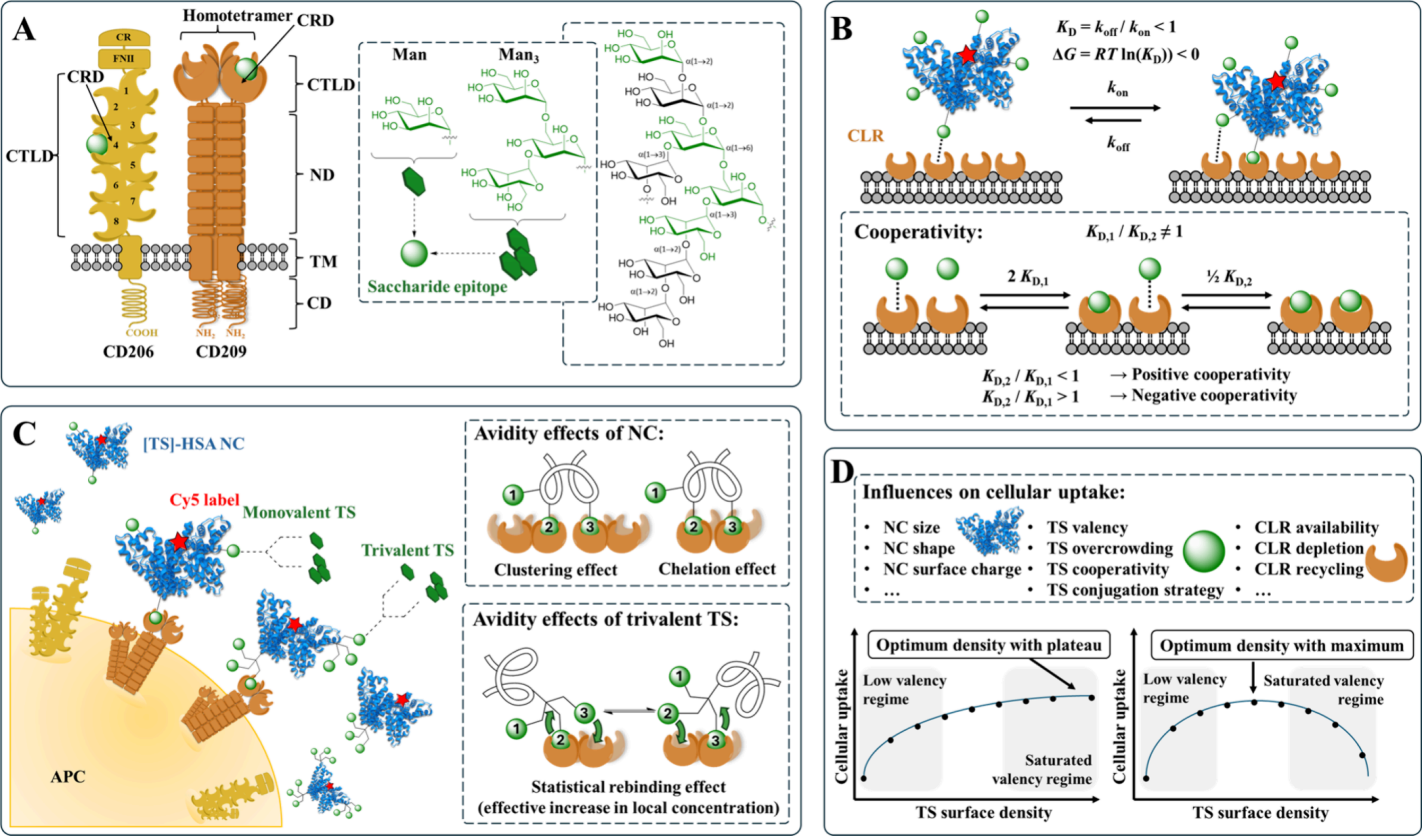
(A) Functional domains of CD206 and CD209: Cysteine-rich (CR), fibronectin type II (FNII), C-type lectin (CTLD), carbohydrate recognition (CRD), neck (ND), transmembrane (TM), and cytoplasmic domain (CD).^11,104,105^ Saccharide epitopes Man and Man_3_ of the nonasaccharide substructure from high-mannan glycans chosen as TS for CLR binding. (B) Multivalent interactions of NC and cell surface form multiple TS-CRD complexes governed by avidity of NC as a product of intrinsic affinities of individual TS and valency of NC resulting in avidity effects. Formation of initial complex exerts influence on subsequent formations.^81^ (C) APC targeting with HSA-NCs presenting mono- and trivalent TSs carrying Man or Man_3_ epitopes.^11^ Cooperative avidity effects like simultaneous clustering of one CRD of two different CLRs or chelation of two CRDs of one respective CLR (NC multivalency) as well as an effective increase in the local concentration of epitopes by statistical rebinding (TS multivalency) influence uptake.^64–70^ (D) Further properties of NCs and TS influencing cellular uptake, with dependency on TS surface density resulting in two trends: (a) increase until constant threshold or (b) increase until maximum, followed by decline.^76^

## 2 Experimental Section

### 2.1 General Experimental Conditions

For the synthesis of the HSA-based compounds, all reactions were performed without taking precautions to exclude air and moisture, unless stated otherwise. Organic solvents (CH_3_CN, HPLC grade; CH_2_Cl_2_, HPLC grade; DMF, peptide grade; DMSO, analysis grade) were obtained from Thermo Fisher Scientific (Waltham, MA, USA) and used without further purification. Milli-Q water (MQ) was obtained from a Millipore purification system. For the synthesis of carbohydrate-based compounds, reactions involving sensitive and reactive species were conducted under an atmosphere of argon-gas in dried glass ware utilizing the standard Schlenk technique. Dried solvents were used, obtained from a SPS 5 solvent drying system (M. Braun Inertgas-Systeme, Garching, BY, Germany) for toluene and DCM. Further dried solvents were obtained from Acros Organics (Geel, Belgium) in AcroSeal bottles over molecular sieves for DMF, MeOH and pyridine. Glycosylation reactions were conducted in the presence of activated spherical molecular sieve beads (diameter 1–2 mm, pore size 3 Å) supplied from Alfa Aesar (Haverhill, MA, USA). The stated temperatures refer to the temperatures measured with a contact thermometer in the used heating mantle or cooling bath. Heated reactions were conducted in an aluminum block placed on the stirring plate. Glycosylation reactions conducted in a temperature range between −40 °C to −20 °C were placed in an acetone bath, which had its temperature adjusted using a FT902 cryostat (Julabo, Seelbach, BW, Germany). Zemplén-deacylations were neutralized with the ion-exchange resin Amberlite IR 120 (*H*-Form) supplied by Merck Millipore (Burlington, MA, USA). The resin was thoroughly washed with MeOH, H_2_O, 1 M HCl, and MeOH in that order prior to use utilizing a glass frit. To remove molecular sieves, ion-exchange resin, and other solids prior to reaction work up, reaction mixtures were filtered through a glass frit using Celite Hyflo Super Cel diatomaceous earth, supplied from Sigma-Aldrich (St. Louis, MO, USA).

### 2.2 Synthesis of Saccharide Targeting Structures

To present one or three Man or Man_3_ epitopes, the clickable monovalent TSs ManN_3_, Man_3_N_3_, and the Newkome-type^106^ glycodendrons (Man)_3_N_3_, and (Man_3_)_3_N_3_ were prepared (Scheme 1). The contained flexible ethylene glycol-based linkers enable conjugation to dibenzocyclooctyne (DBCO)-modified HSA derived NCs via strain-promoted azide alkyne cycloaddition (SPAAC). Their synthesis involves the preparation of saccharide, linker, and predendron building blocks using a set of efficient and scalable synthesis protocols evolving around the copper-catalyzed azide alkyne cycloaddition (CuAAC) reaction for their assembly (Scheme S1 in the Supporting Information for details on the synthesis steps and reaction conditions). The building blocks are summarized in the upper part and the assembled TS in the lower part of Scheme 1. The TSs were obtained with significantly improved yields compared to prior reports (e.g., Man_3_N_3_: 4% over eight steps).^75^ Higher efficiency and robustness of the synthesis route up to the multigram scale results from telescoped reaction protocols (two-step propargylation and Zemplén-deacylation sequence^107^ and three-step silylation, benzoylation, desilylation sequence^108^). All building blocks and intermediates were characterized by ^1^H NMR, ^13^C NMR, 2D NMR, and high-resolution mass spectrometry (HR-MS). The unconjugated TSs were used in the NanoDSF study and for conjugation to the [DBCO]-HSA precursor.

**Scheme 1:**
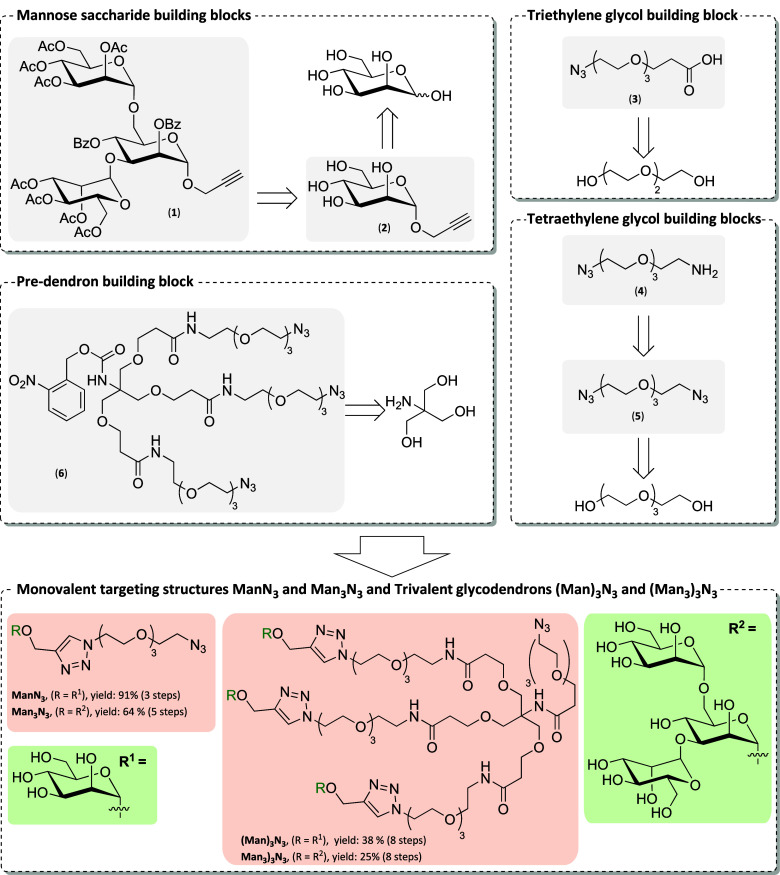
Retrosynthesis of Building Blocks (Grey Highlight) Involving Mannoses (**1**) and (**2**), Linkers (**3**), (**4**), and (**5**), and Predendron (**6**) for Assembly of Monovalent and Trivalent TSs (Orange Highlight) with Abbreviated Residues (Green Highlight)^a^

a Yields and step count are stated for each TS. Synthesis and analytical data are displayed in section 4.1 of the Supporting Information.

### 2.3 Differential Scanning Fluorimetry (NanoDSF) Study of Unconjugated TS

Thermal shift experiments were performed by using a Prometheus NanoDSF instrument (NanoTemper, Munich, Germany). Each sample, containing 12 μM CD209 CRD or CD206 CRD4 (recombinant protein expression and purification displayed in section 6.1 of the Supporting Information) together with varying ligand concentrations in binding buffer (150 mM NaCl, 25 mM Tris-Cl, pH 7.8, and 25 mM CaCl_2_), was loaded into NanoDSF capillaries. Each ligand concentration was measured in triplicate. Samples were subjected to a controlled temperature increase from 20 to 85 °C at a rate of 1.5 °C per minute. Protein unfolding was monitored by measuring the fluorescence emission ratio at 350 and 330 nm. The FoldAffinity tool developed by Niebling et al.^109^ was used to analyze the melting curves (data displayed on section 6.2 of the Supporting Information) and determine the unfolded fraction at each ligand concentration, evaluated at 53 °C for CD206 and at 63 °C for CD209. Data were analyzed according to the vertical slice method developed by Bai et al.^110^ The unfolded fraction was plotted against the ligand concentration, and EC_50_ values were determined by fitting the data to the Hill equation using GraphPad Prism 8.0.1. *K*_D_ values were calculated using eq 1 ^110^ based on the unfolded fraction in the absence of ligand (*f*_uo_) and the total protein concentration ([*P*]). Associated errors were determined according to the standard error propagation rules.

$$K_{D}=\left(1-f_{\mathrm{uo}}\right)\times \left({EC}_{50}-\frac{\left[P\right]}{2}\right)$$ 1

### 2.4 Matrix-Assisted Laser Desorption Ionization – Time of Flight Mass Spectrometry (MALDI-ToF-MS)

Mass spectra for HSA-based compounds were acquired on a ToF MS rapifleX spectrometer (Bruker Corporation, Ettlingen, BW, Germany) using sinapinic acid as the matrix. The molecular mass for all compounds was calculated as the *m*/*z* value using the peak center representing the singly charged species.

### 2.5 Agarose Gel Electrophoresis

An 1% agarose gel (0.5 g agarose, 50 mL 1 × TAE buffer) was prepared, and protein (∼1–3 μg) samples were loaded. The gel was run at 150 V for 33 min. The gel was imaged using the Cyanine 5 (Cy5)-channel and stained with coomassie brilliant blue before imaging using a ChemiDoc Imaging system (Bio-Rad Laboratories, Hercules, CA, USA) using the coomassie brilliant blue channel.^111^

### 2.6 Dynamic Light Scattering (DLS)

Measurements of HSA-based compounds (10 μM in MQ) were performed at 25 °C using a Zetasizer Nano S (Malvern Instruments, Malvern, England) equipped with a He/Ne Laser (λ = 633 nm) and a narrow band filter at a fixed scattering angle of 173 °. Volume distribution of DLS measurements of compounds were used to determine the compounds size distribution.

### 2.7 Synthesis of HSA Conjugates

Synthesis procedures were adapted^102^ and modified, as provided in detail in the SI (section 4.2 in the SI). Briefly, native HSA was dye-labeled using Sulfo-Cy5-Maleimide at Cys34 in 50 mM PB, pH 7.4, followed by ring-opening for stabilization in 50 mM borate buffer, pH 9.2 (HSA). The HSA was purified via spin filtration (Vivaspin, 10 kDa cutoff). A fixed number of DBCO-groups (13 or 47, as determined by the average molecular weight from MALDI-ToF-MS analysis) were statistically attached to lysine residues randomly distributed on the NC surface via DBCO-PEG_4_-NHS in 50 mM PB, pH 7.4 yielding DBCO-HSA. Subsequently, synthetic azide-functionalized TS (ManN_3_, Man_3_N_3_, (Man)_3_N_3_, or (Man_3_)_3_N_3_; MQ, 10 mg/mL) was added to DBCO-HSA (1 mg/mL) in 50 mM PB, 8 M urea, 2 mM EDTA. Equivalents of TS and reaction time were strictly controlled and adjusted based on reaction progress monitoring via MALDI-ToF-MS. For conjugates with *n*(TS) = 1–9, remaining free DBCO groups were capped using excess N_3_–PEG_3_–OH. All HSA conjugates were purified via spin filtration (Vivaspin, 10 kDa cutoff) and obtained with recovery rates ranging from 38% to quantitative. The degree of modification of HSA with TS was controlled qualitatively via agarose gel electrophoresis (Figure S1 in the Supporting Information) and quantitatively via MALDI-ToF-MS analysis. In total, five groups of NCs were prepared following the described procedure, involving the control group C [C I = Cy5-labeled HSA, C II = DBCO-HSA with 13 DBCO groups attached, and C III = HO-PEG_3_-HSA, in which the 13 DBCO groups were saturated with capping reagent], the ManN_3_ modified group M [M I, M II, M III, M VI, M IX, M_sat_], the (Man)_3_N_3_ modified group [MD I, MD II, MD III, MD_sat_], the Man_3_N_3_ modified group (TM I, TM II, TM III, TM IV, TM IX, TM_sat_] and the (Man_3_)_3_N_3_ modified group [TMD I, TMD II, TMD III, TMD_sat_], where the Roman numeral always represents the number of TS groups attached. This systematic matrix of NCs with known amounts of epitopes of different structures at variable TS density and NC valency enables the subsequent interpretation of in vitro experiments.

### 2.8 Cell Culture

Murine bone marrow cells (2·10^5^/mL for GM-CSF supplemented culture) were seeded in 12-well suspension culture plates (Greiner Bio-One, Frickenhausen, BW, Germany) in culture medium (IMDM, 2 mM l-glutamine, 100 U/mL penicillin G, 100 μg/mL streptomycin (Sigma-Aldrich, Deisenhofen, BY, Germany) and 50 μM β-mercaptoethanol (Carl Roth, Karlsruhe, BW, Germany) containing 5% FBS (PAN-Biotech, Aidenbach, BY, Germany)) supplemented with recombinant murine GM-CSF (10 ng/mL) (Miltenyi Biotec, Bergisch Gladbach, NRW, Germany). Cells were kept at 37 °C, 95% relative humidity, and 5% CO_2_. Culture media was replenished on day 3 and 6 with GM-CSF supplemented cell culture medium.

### 2.9 Spleen Cell Isolation

Murine spleens were mechanically disrupted with a pestle and pressed through a cell strainer with a pore size of 40 μm (Greiner Bio-One, Frickenhausen, BW, Germany) to obtain a single-cell suspension. Erythrocytes were lysed using 2.00 mL of Gey’s Red Cell Lysis buffer (H_2_O dest., 100 μM EDTA, 10.0 mM KHCO_3_ and 155 mM NH_4_Cl) for 1 min at room temperature. Subsequently, cells were washed using an IMDM-based culture medium containing 5% FBS (PAN Biotech, Aidenbach, Germany), 2.00 mM l-glutamine, 100 IU/mL penicillin, 100 μg/mL streptomycin, and 50.0 μM β-mercaptoethanol. Isolated splenocytes (2 × 10^6^/500 μL) were used for *in vitro* experiments and stained for FACS analysis.

### 2.10 Fluorescence Activated Cell Sorting (FACS)

After treatment, GM-CSF BMDCs and splenocytes were harvested, washed with staining buffer (PBS, 1% FBS, 0.5 mM EDTA) and incubated with rat antimouse CD16/CD32 antibody (clone 2.4G2; 15 min, 4 °C) to prevent antibody binding to Fcγ receptors. Following this, the samples were incubated with fluorescence-labeled antibodies (20 min, 4 °C). Then, samples were washed with PBS and incubated with a flexible viability dye (FVD, 1:1000 in PBS, 30 min, 4 °C) to identify live/dead cells. Measurements were carried out using an Attune NxT flow cytometer and data were analyzed using Attune NxT software (both are from Thermo Fisher, Waltham, MA, USA).

## 3 Results and Discussion

### 3.1 Prediction of Binding Modes for Monovalent TSs

Predicted binding modes for the TS Man (**7**) and Man_3_ (**8**), with a truncated linker unit (Figure 2A), were generated by molecular docking to investigate potential influences of linker placement on binding mode. The molecular docking-predicted binding mode of the TS Man (**7**) to the CRD of CD209 showed polar interactions between the Man epitope and the Ca^2+^-cation inside the principal Ca^2+^-binding site, as well as to the surrounding amino acids Glu347, Asn349, Glu354, Lys368, and Asn365 (Figure 2B). The truncated linker is oriented toward the protein surface and interacts with water molecules inside the pocket. The predicted binding mode of the TS Man_3_ (**8**) with the CRD of CD209, indicates that the Man_3_ epitope predominantly binds through the terminally α-(1→3)-connected mannose residue, with the same interactions as the Man epitope in the prior case. The central mannose moiety forms no additional hydrogen bonds to the CRD, while the terminal α-(1→6)-linked mannose residue forms additional polar interactions with Ser360 in the secondary binding site, consistent with previous crystal structures of similar compounds (Figure 2B).^112^ Molecular docking of the TS Man (**7**) to the CRD of CD206, showed that the Man epitope similarly engages in polar interactions with the central Ca^2+^-cation and surrounding amino acids Glu725, Asn727, Glu733, and Asn747 (Figure 2C). The linker forms a hydrogen bond to Lys739. In the predicted binding mode of the TS Man_3_ (**8**) with the CRD of CD206, the characteristic polar interactions again arise from the α-(1→3)-linked mannose residue, with an additional polar interaction between the α-(1→6)-linked mannose and Glu725. The linker interacts with a binding-site water molecule. All linkers are found at the solvent-exposed protein surface, with their orientation pointing away from the primary binding site. Therefore, this attachment point seems to be suitable for linking Man moieties for glycodendrons.

**Figure 2:**
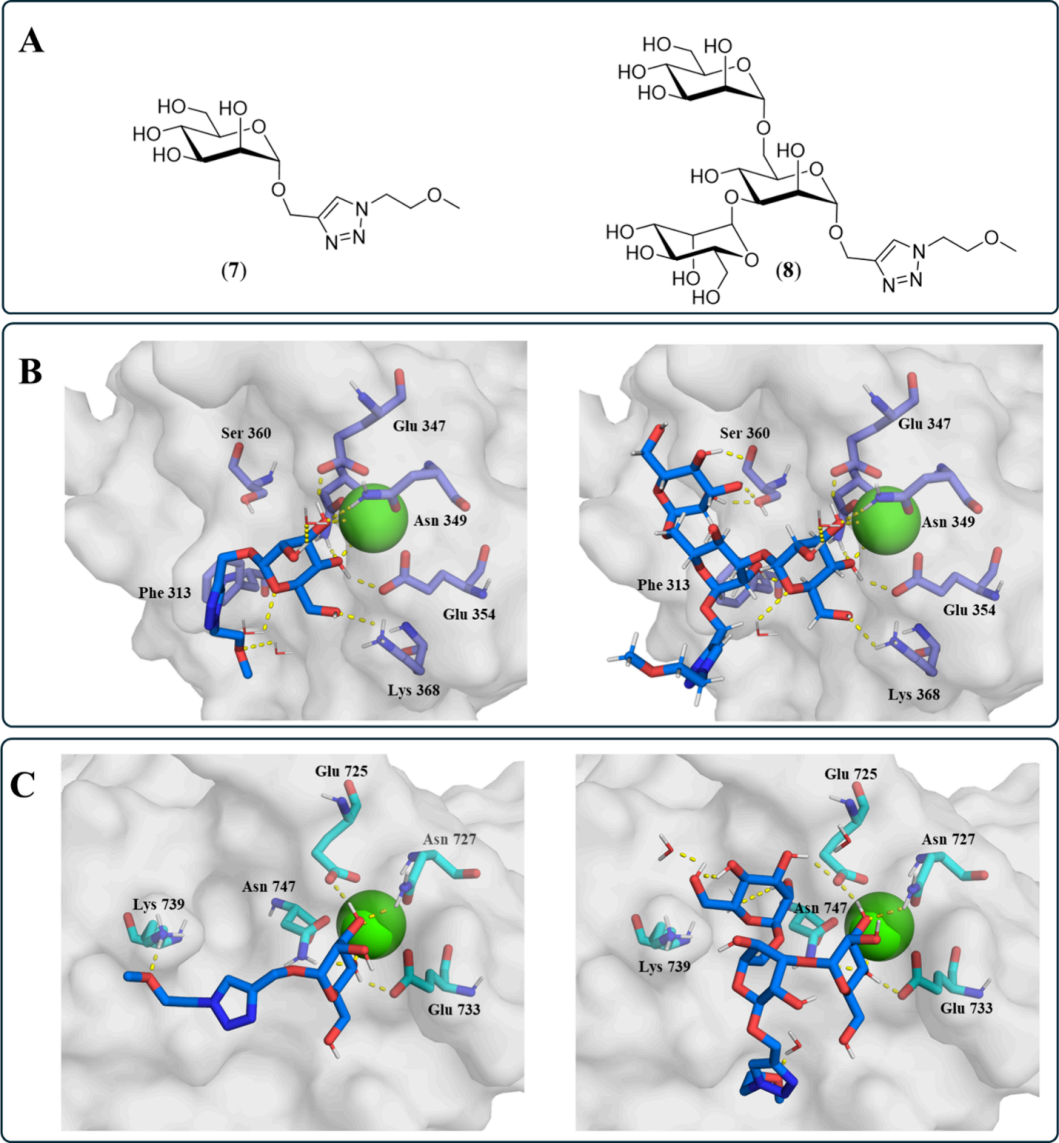
(A) TS Man (**7**) and Man_3_ (**8**) with truncated linkers used for the in silico binding mode study. (B) Predicted binding modes for TS Man (**7**) (left) and TS Man_3_ (**8**) (right) in complex with CD209 (PDB: 1SL4).^112^ (C) Predicted binding mode for TS Man (**7**) (left) and TS Man_3_ (**8**) (right) in complex with CD206 (PDB: 7JUF).^31^ TS displayed as sticks with blue C-atoms, protein surfaces in light gray, selected amino acid residues inside the CRD as sticks with purple (CD209) or light blue (CD206) C-atoms and label, Ca^2+^-cations as green spheres, water molecules as lines, and polar interactions as yellow dashed lines.

### 3.2 NanoDSF Binding Study for Mono- and Trivalent TSs

The binding affinity of CRD4 of CD206 to saccharide ligands has been mainly investigated by NMR titration and competition binding assays, yielding high μM to low mM affinities.^31,113^ For CD209, surface plasmon resonance (SPR), isothermal titration calorimetry (ITC), and solid-phase competition assays have been employed to measure the affinity of both the tetrameric extracellular domain (ECD) and the monomeric carbohydrate recognition domain (CRD) toward different mannose variations, with reported values ranging from the low millimolar range to being too weak to detect.^35,50,63,114–117^ Notably, the isolated CRD is known to have weaker affinities to its ligands than the tetrameric ECD.^36^ A growing body of literature has demonstrated that (Nano)­DSF can be utilized to obtain affinity values even in the mM range, which is challenging for other biophysical methods, while having a relatively low sample consumption.^109,110,118,119^ The method has previously been used to screen possible ligands of CD209.^115^

In this study, NanoDSF was used to investigate the binding affinity of unconjugated monovalent TSs ManN_3_ and Man_3_N_3_, as well as the trivalent TSs (Man)_3_N_3_ and (Man_3_)_3_N_3_ for isolated CRDs of CD206 and CD209. Utilization of isolated CRDs instead of the complete tetrameric and octameric receptors allowed an investigation of the affinity without the potential occurrence of avidity effects taking place on the receptor side. For the interaction of ManN_3_ and (Man_3_)_3_N_3_ with the CRD of CD209, only a lower affinity estimate could be obtained, as the tested concentrations of TSs did not achieve sufficient saturation to determine a definitive EC_50_ value. The observed binding affinities to the isolated CRDs were in the low millimolar range, with all ligands showing stabilizing effects. This confirms that the presence of linkers does not prevent interactions between the mannose epitopes and the CRD. For the monovalent TSs, CD206 showed a slight preference for the Man over the Man_3_ epitope (1.3 mM vs 6.9 mM; Figure 3), with the affinity of the TS ManN_3_ being in accordance with *K*_i_ values measured via binding competition and NMR assays.^31,113^ Notably, the *K*_D_ value of the TS Man_3_N_3_ is slightly higher than what was determined in a solid phase competition assay for a similar structure (*K*_i_ = 0.3 mM).^31,113^ The difference might originate from differences in the methods. In the solid-phase assay, the biotinylated CRDs were immobilized on streptavidin-coated plates in a defined orientation, likely enhancing the effective presentation of the binding site.^113^ In contrast, NanoDSF measurements were conducted with isolated, solubilized monomeric CRDs without surface- or orientation-related effects. Additionally, steric effects and interactions arising from the linker moiety may also impact binding affinity.^115^

**Figure 3:**
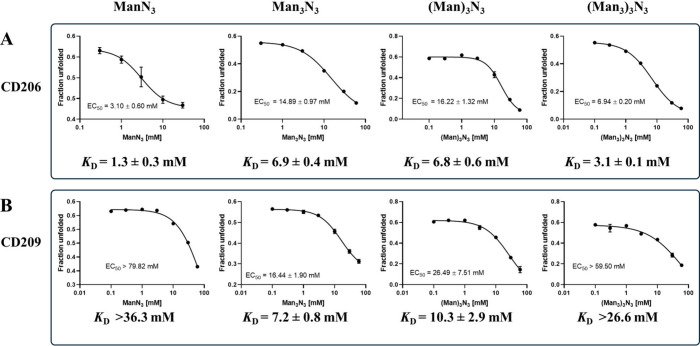
NanoDSF study for binding of mono- and trivalent TSs ManN_3_, Man_3_N_3_, (Man)_3_N_3_, and (Man_3_)_3_N_3_ to the CRDs of CD206 and CD209. (A) Affinity of mono- and trivalent TSs for CRD4 of CD206. (B) Affinity of mono- and trivalent TSs for CRD of CD209. The unfolded fraction of each CRD at different TS concentrations was determined at 53 °C for CD206 and at 63 °C for CD209. The unfolded fraction was plotted against TS concentration, and EC_50_ values were determined using GraphPad Prism 8.0.1. The respective *K*_D_ values were calculated from EC_50_, as described in section 2.3, and are given for each case.

For binding to CD209, the opposite trend compared to CD206 could be observed, favoring Man_3_N_3_ over ManN_3_ (>36.3 mM vs 7.2 mM), which is consistent with previous publications.^114,117^ The preference of CD209 for Man_3_N_3_ might result from the additional interactions formed by the α-(1→6)-connected mannose unit in Man_3_ observed in the docking studies (Figure 2). The multivalent presentation of carbohydrate residues did not lead to a large increase in the affinity. For CD206, the affinities of the trivalent TSs were in the same range as for the monovalent TSs, with (Man)_3_ having slightly lower affinity than (Man_3_)_3_ (6.8 mM vs 3.1 mM, Figure 3). For CD209, there was a slight trend indicating an increase in affinity of the trivalent (Man)_3_-TS compared to the monovalent Man-TS (10.3 mM vs >36.3 mM, Figure 3). However, for the Man_3_ epitope, the trivalent (Man_3_)_3_-TS exhibited reduced affinity compared to the monovalent Man_3_-TS (>26.6 mM vs 7.2 mM, Figure 3). Li et al. reported comparable, monovalent Man and Man_3_-TSs involving a triazole moiety used for an SPR assay with tetrameric extracellular domains (ECDs) of CD209 immobilized and all four CRDs oriented toward the solvent, thus mimicking the natural presentation on cell surfaces.^117^ The affinities determined for the monovalent TSs were in a similar range to those measured by NanoDSF (Man too weak to detect and Man_3_ with IC_50_ = 1.5 mM). However, when the TSs were presented in a trivalent manner, an increase in binding affinity was observed (e.g.: 1.5 mM monovalent vs 2.4 μM trivalent for trimannose),^117^ which was not the case in the NanoDSF experiments. Overall, these results suggest that the multivalent presentation of the receptors is crucial to benefit from avidity effects that cannot result from the multivalent ligand presentation alone (Figure 1C). Additionally, in solution, steric hindrance between individual CRDs may limit their simultaneous engagement with a multivalent ligand. The binding interaction between a CRD and a carbohydrate epitope could reduce the accessibility of the remaining epitopes for binding to additional CRDs, also constricted by an increased entropy penalty in comparison to immobilized or preorganized tetrameric CRDs. Intramolecular interactions between the carbohydrate moieties could further interfere with receptor recognition or present key epitopes in suboptimal orientations. In summary, all tested TSs demonstrated measurable binding to the isolated CRDs, confirming that overall, the mannose epitopes remained accessible and functional despite linker modifications and multivalent presentation.

### 3.3 Prediction of Distances of Mannose Epitopes of a Trivalent TS

As initial prediction for the avidity effects expected of the trivalent TSs (Figure 1C) interacting with the tetrameric CD209 receptor, a set of conformers (*n* = 1000) of the TS (Man)_3_N_3_ was generated using Omega.^120,121^ This set of conformers allowed measurement of the possible distances spanned by two of the involved mannose epitopes (measured between hydroxy groups at C-4) as well as minimal and maximal distance of one epitope from the focal point of the glycodendron (measured between central carbon and hydroxy groups at C-4) within the set of conformers (Figure 4, overall distribution shown in Figure S8 in the Supporting Information). Calculation of average distances, for all epitopes, enabled a comparison with the average distance between two Ca^2+^-ions within the two closest CRDs of the CD209 homotetramer. For chelation effects to originate from the trivalent TS, the distance of about 40 Å needs to be spanned by the glycodendron to enable interaction of two mannose epitopes at two CRDs at the same time, based on a model of the CD209 tetramer (Figure 4B), which is in line with SAXS and MD studies.^98^ With the conformers of the TS (Man)_3_N_3_ spanning shorter distances on average (Figure 4A), the glycodendrons should not be capable of engaging in chelating effects. The occurrence of clustering effects involving multiple CD209 homotetramers originating from trivalent TS is highly unlikely. In line with this, NanoDSF, with isolated, monomeric CRDs did not reveal a pronounced affinity gain for trivalent ligands (Figure 3). While this experiment cannot provide a direct test of chelation, because only single-site binding events per CRD are monitored, the NanoDSF data do not contradict the modeling prediction. Therefore, potential increases in binding strengths in a biological setting with correctly immobilized full receptors are most likely a result of increased local concentration of mannose epitopes at one CRD via statistical rebinding (Figure 1C).^87^ Avidity effects originating from NCs cannot be predicted within this simplified model and could possibly involve clustering or chelation effect (Figure 1C). Within a biological setting, cellular uptake of NCs could benefit from avidity effects originating from the TSs boosting the effects resulting from the NCs themselves.^98^

**Figure 4:**
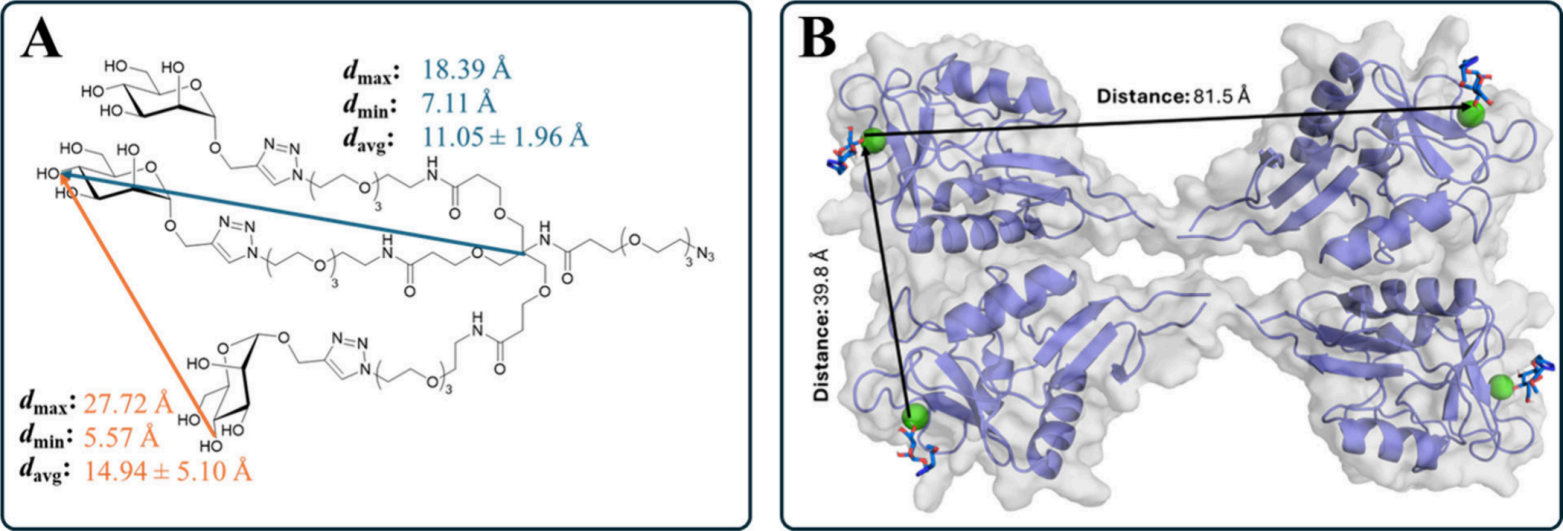
(A) Minimal, maximal, and average distances spanned by two Man epitopes measured between hydroxy groups at C-4 (orange line), as well as minimal, maximal, and average distances of one epitope from the focal point of the glycodendron measured between central carbon and hydroxy groups at C-4 (blue line) obtained from a set of conformers (*n* = 1000) of TS (Man)_3_N_3_. (B) Model of tetrameric CD209 using CD209 monomers (PDB: 1SL4)^112^ aligned on tetrameric DC-SIGNR (PDB: 1XAR, C_α_ RMSD: 0.41 Å).^104^

### 3.4 Targeting Capability of TS-Decorated NCs *In Vitro*

To investigate the influence of the designed TSs in a more application-focused setting, we proceeded with *in vitro* experiments using receptors organized in their natural form. For this purpose, we synthesized different HSA-based NCs decorated with all tested TSs (section 4.2 in the Supporting Information) either within the low-valency regime carrying one to three mono- and trivalent TSs (TS I–III), six and nine monovalent TSs (TS VI, TS IX) per NC or within the saturated valency regime saturated with the maximum possible number of TSs per NC (38–45, TS_sat_). The resulting systems thus contain comparable amounts of Man and Man_3_ epitopes per NC, either monovalent or clustered within the glycodendrons, statistically distributed on the surface. The low degrees of modification are supposed to allow a systematic study of potential contributions of avidity effects of the trivalent TSs to receptor interaction and cellular uptake, which would be otherwise superimposed by avidity effects originating from NCs with high valency. Untreated cells (UT) or cells treated with the TLR7/8 agonist R848 as well as cells treated with control conjugates, which are Cy5-labeled only (C I), DBCO-modified (13 groups, C II) or DBCO-modified and capped via SPAAC with triethylene glycol residues (C III), were used as controls for comparison. An *in vitro* study comprising overnight incubation of Granulocyte macrophage colony-stimulating factor (GM-CSF) BMDCs together with the Cy5-labeled TS-HSA conjugates and following FACS analysis was conducted (Figure 5 and Figure 6). Generally, cellular uptake of TS-HSA conjugates resulted from the attachment of TSs, with the untargeted HSA-derived NCs providing only limited unspecific uptake, as seen from controls C I, C II, and C III (Figure 5C). Cellular uptake of the TS-HSA conjugates does not induce changes in expression levels of the CLRs CD206 and CD209, as well as of the activation marker CD86 (Figure 5B), ensuring consistent receptor availability upon treatment and supporting NCs potential immunocompatibility. Observed effects on uptake levels thus are assumed not to be impacted by receptor availability. Also, an influence of slow receptor recycling, which affects their availability, is unlikely for CD206 and CD209 due to the reported rapid recycling kinetics and the long incubation time overnight.^122,123^ Additionally, receptor depletion, which is most relevant for nano rods and large NCs (>60 nm), is supposed to be neglectable as well due to the size (∼7 nm) and globular shape of the HSA-NCs.^76,124–126^ In the following, the cellular uptake as a measure of targeting capability and thus indirectly also binding strength of the four groups of TS-HSA conjugates (M, TM, MD, TMD, Figure 5C) is analyzed and evaluated regarding their general uptake trend and the influence of (i) TS surface density and NC valency, (ii) the epitope type, and (iii) epitope clustering. The general uptake trends of NCs in BMDCs functionalized with increasing numbers of statistically attached TSs are visualized with a linear plot of the cellular uptake against the number of TSs per NC for all conjugates (Figure 5A) and by plots grouping respective conjugates to enable comparison of NC valency and surface density within each TS type (Figure 5C). For Man-decorated NCs, the addition of statistically distributed TS increases the surface density and leads to an approximately logarithmic increase in cellular uptake at the beginning of the low-valency regime with a significant benefit from M VI onward compared to control conjugate C III. However, the benefit of each additional Man decreases as the total number increases, and the uptake rate slows down. Although a significant increase continues into the saturated valency regime, it becomes less pronounced relative to the TS number and eventually approaches a plateau. For the herein tested Man-decorated NCs, M_sat_ showed the highest cellular uptake. For Man_3_-decorated NCs, increasing surface density is also accompanied by an increase in cellular uptake with a significant benefit for TM II. However, a plateau in cell uptake is already reached at a lower TS number from conjugate TM VI onward with no additional significant increase afterward. The onset of this plateau was suggested to occur around a modification rate of 9 TM groups in similar conjugates in GM-CSF BMDC at the same applied concentration prior to this study.^102^ In summary, increased surface density of our monovalent TSs results in increased cellular uptake, with the greatest gain in the low valency regime and maximum cellular uptake being achieved from conjugate TM VI onward. The observed situation resembles the trend of “optimum density with plateau” (Figure 1D). For NCs decorated with our trivalent TS (Man)_3_ and (Man_3_)_3_, there is a significantly enhanced cell uptake compared with unmodified NCs for the conjugates MD I and TMD I, respectively. The further increase in the number of TS in the low-valency regime is not accompanied by any significant changes in cellular uptake. At the transition into the saturated valency regime, both conjugate MD_sat_ and conjugate TMD_sat_ show a significant decrease in cellular uptake; in the case of TMD even to the same level as control conjugate C III. Since no visual precipitation or signs of increased aggregation via DLS (Table S2 and Table S4 in the Supporting Information) could be detected, this strong decrease in cellular uptake could reflect the decline in the trend “optimum density with maximum” (Figure 1D) triggered by effects discussed above, like steric overcrowding or negative cooperativity of TSs, superimposing potential avidity effects by clustering or by the NC itself. However, the precise occurrence of the optimum surface density cannot be determined due to the minimal variation in uptake within the low-valency regime and the possibility that the optimum is already reached with the attachment of a single trivalent TS in the form of the conjugates MD I and TMD I.

**Figure 5:**
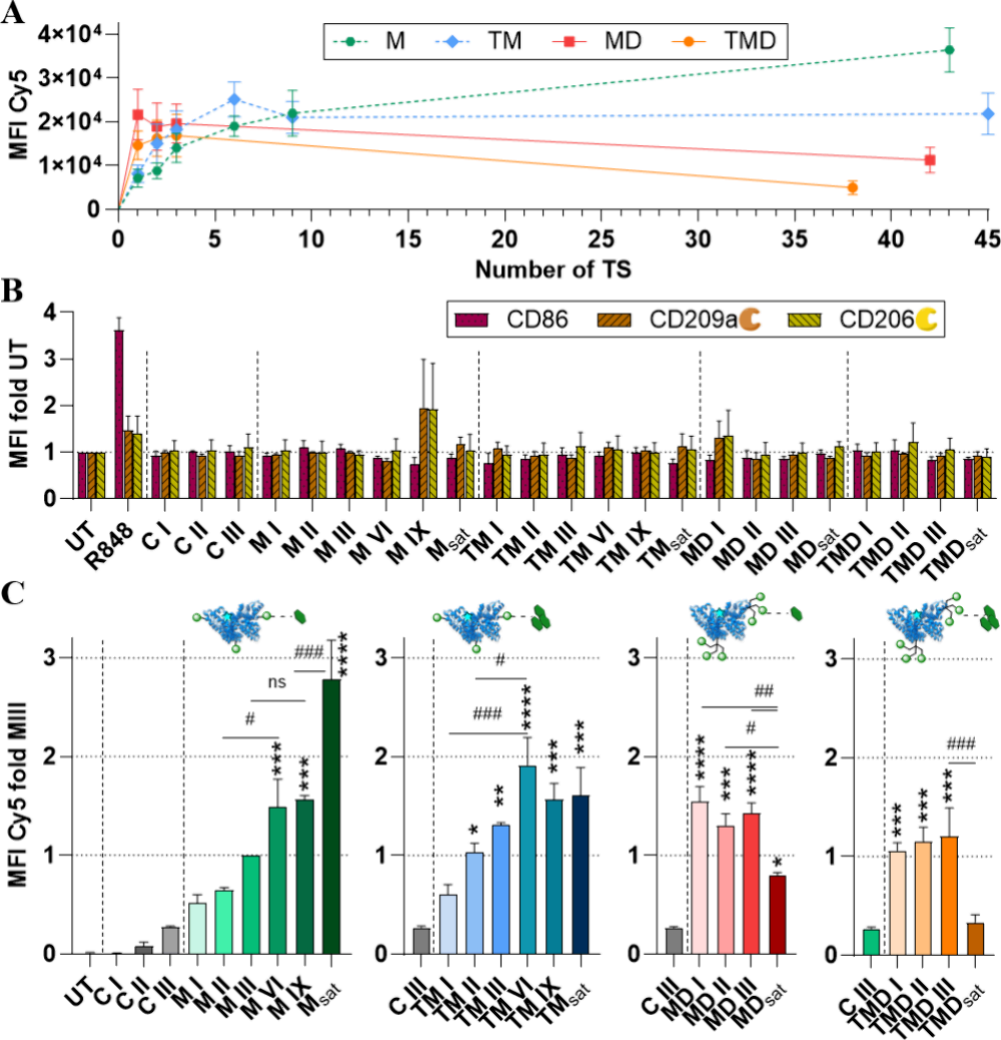
FACS analysis of cellular uptake in GM-CSF BMDCs after incubation overnight with different HSA conjugates (50 nM). (A) Linear plot of the cellular TS-HSA conjugate uptake versus the number of ligands attached. (B) Receptor expression of targeted CLRs (CD206, CD209) and activation marker CD86 upon treatment. (C) Normalized cellular TS-HSA conjugate uptake comparing the NC valency and surface density within each TS type. Data represented as mean ± SEM, *n* = 3; statistical differences are indicated between conjugates #, and vs control conjugate C III* (one-way ANOVA, Tukey test). *,# *p* < 0.05, **,# *p* < 0.01, ***,# *p* < 0.001, ****,# *p* < 0.0001. UT = Untreated control.

**Figure 6:**
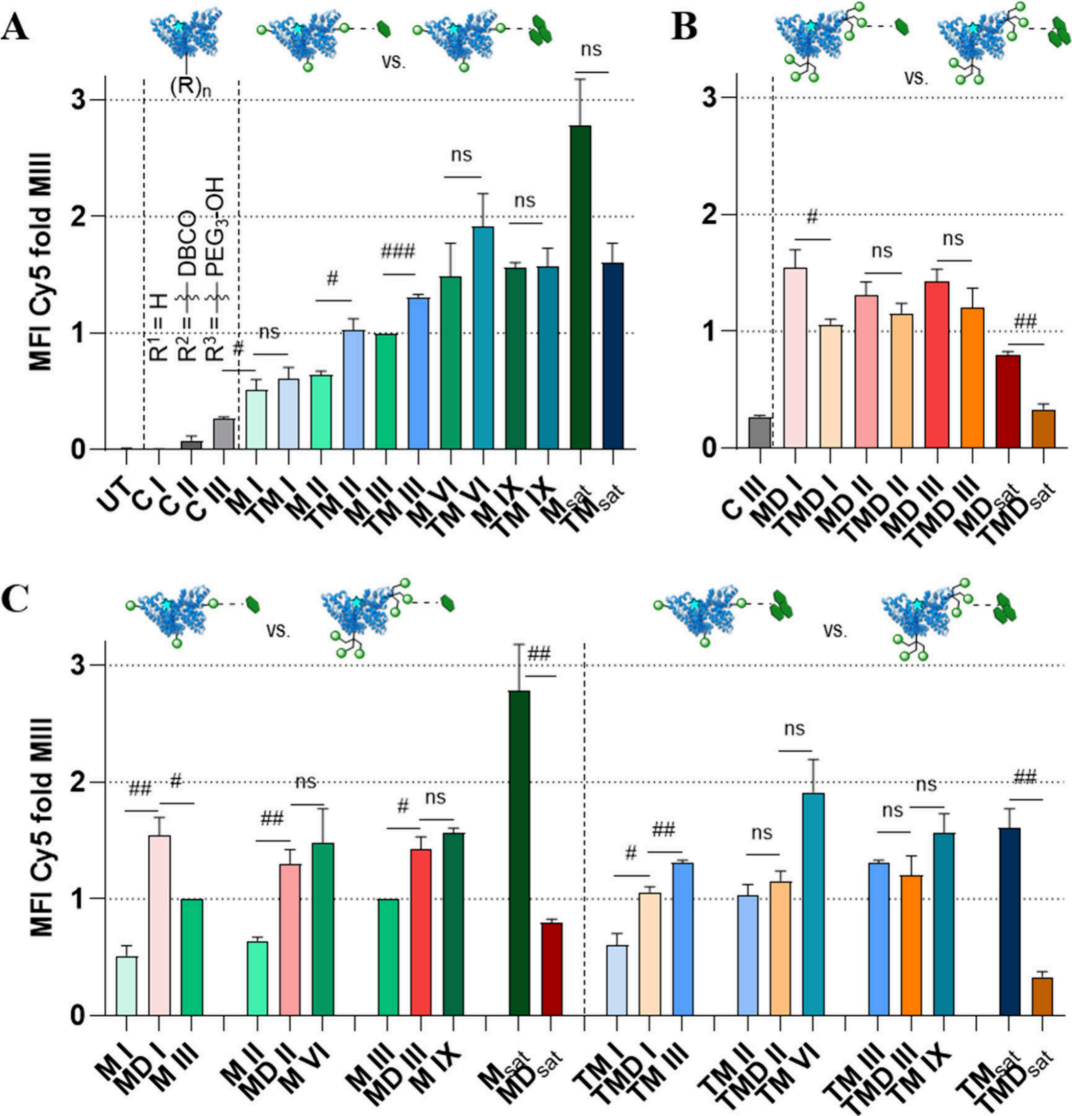
FACS analysis of cellular uptake in GM-CSF BMDCs after incubation overnight with different HSA conjugates (50 nM). Normalized cellular TS-HSA conjugate uptake was calculated by comparing epitope type in (A) M/TM conjugates and (B MD/TMD conjugates and (C) epitope clustering. Data are presented as mean ± SEM, *n* = 3; statistical differences are indicated between conjugates # (*t* test). # *p* < 0.05, # *p* < 0.01, # *p* < 0.001, # *p* < 0.0001. UT = Untreated control.

The difference in cellular uptake mediated by epitope type (Man vs Man_3_) is evident from the direct comparison of the respective conjugates decorated with these NCs, as plotted in Figure 6A. Within the low-valency regime, there is a consistent trend that the structurally more complex Man_3_ epitope induces a slightly higher cellular uptake, which is statistically significant in a direct comparison of conjugates M II/TM II and M III/TM III, respectively. This is in accordance with literature reports attributing higher affinity to Man_3_ ^31,51^ and with our docking studies (Figure 2). These differences in cellular uptake are attenuated for conjugates M IX/TM IX and the ones with a higher degree of functionalization, with TM conjugates reaching the onset of the observed plateau already within the low-valency regime and at lower MFI levels (Figure 5A and C). This earlier onset of negative effects, like overcrowding, for the TM conjugates could be due to the higher steric demands of the larger epitope. This is also supported by a comparison of the conjugates carrying the different epitope types in their trivalent form (Figure 6B). Conjugates from the TMD group tend to have lower cellular uptake compared with the MD group throughout both regimes of valency, with statistically significant differences between MD I/TMD I and MD_sat_/TMD_sat_, respectively. This is presumably due to the higher steric hindrance for the larger Man_3_-epitopes when clustered within the glycodendrons, thereby superimposing the inherently higher binding affinity of the Man_3_ epitope. The effects of epitope clustering become apparent when conjugates carrying the glycodendron are compared with conjugates carrying the same number of monovalent TS or the same overall number of epitopes presented (Figure 6C). With increasing TS surface density, the ratio of presented epitopes per NC between the groups M vs MD and TM vs TMD remains the same (1:3), but the absolute difference in epitope number increases from 2 to 4 to 6. The conjugate MD I, with one trivalent TS, thus presenting three clustered Man epitopes, shows significantly higher cellular uptake than M I, which has one monovalent TS and thus a single Man epitope. Compared to conjugate M III, which carries three randomly distributed monovalent TSs and thus the same number of epitopes, MD I still achieves higher uptake. This suggests that clustering of epitopes enhances uptake through avidity effects here.

However, while the trivalent Man is superior to the monovalent Man in the subsequent low-valency regime with equivalent TS modification rate (e.g., MD II > M II), this does not apply to conjugates with equivalent epitope count (e.g., MD II vs M VI). And since the conjugates with clustered Man (MD) do not show a significant increase in the cell uptake trend with increasing TS density (Figure 5A), the conjugates with monovalent Man prove to be more effective from a modification rate of two to three onward and especially in the saturated valency regime. For the Man_3_ epitope, e.g., when comparing conjugates TM I vs TMD I vs TM III, enhancing cellular uptake comes with epitope count (e.g., TM I vs TMD I), but not with clustering of the epitopes (e.g., TMD I vs TM III) until TM conjugates enter the region of saturated cellular uptake from TM VI onward. In the saturated valency regime, TM_sat_ outperforms TMD_sat_ because of the above-mentioned negative effects, hampering cellular uptake. In summary, when evaluating the targeting capability of the four investigated TSs on our HSA-based NCs in BMDCs, monovalent TSs lead to a general cellular uptake trend “optimum density with plateau”, with TM conjugates reaching cellular uptake saturation within the low-valency regime and M conjugates demonstrating the overall highest absolute cellular uptake within the saturated regime. Trivalent TSs on our NCs appear to induce negative effects, which increasingly outweigh the intended avidity benefits of epitope clustering early on and with increasing modification rate. Since this trend appears for both trivalent TSs independent of the type of epitope attached, the design of the dendron backbone may be the cause, potentially a result of excessive flexibility, spatially unfavorable arrangement of epitopes, or a too limited span of dendron arms to enable avidity effects. This results in a general trend of “optimum density with a maximum”. Regarding the epitope type, TM conjugates show a higher uptake at the beginning of the low valency regime, but this advantage diminishes with increasing TS count and reverses in the saturated regime.

To test the robustness of the general uptake trends derived for the tested TS-decorated NCs as well as the cell-type selectivity, we performed an additional *in vitro* experiment using a more heterogeneous cell population. We incubated spleen cells derived from murine spleens with TS-conjugates (50 nM) overnight, followed by FACS analysis (Figure 7). In general, the data reveals a higher variability, as expected, and a higher cellular uptake of the control conjugate C III. Among nontargeted splenic immune cells, comprising PMN-, NK-, B-, and T-cells, PMNs exhibit some uptake of TS-conjugates, presumably due to their role as rapid-acting phagocytes in innate immunity. This tendency is slightly more pronounced in M conjugates than in TM/MD/TMD conjugates. However, the absolute cellular uptake among nontargeted splenic immune cells remains low compared to targeted cells. For targeted APCs (DCs and MΦ), the mono- and trivalent TS-conjugates show uptake trends similar to those of BMDCs. It is noteworthy that slight variations occur during the onset of the saturated regime. MD conjugates show a decreased uptake into target cells, whereas M, TM, and TMD conjugates show a similar relative efficiency as in BMDCs.

**Figure 7:**
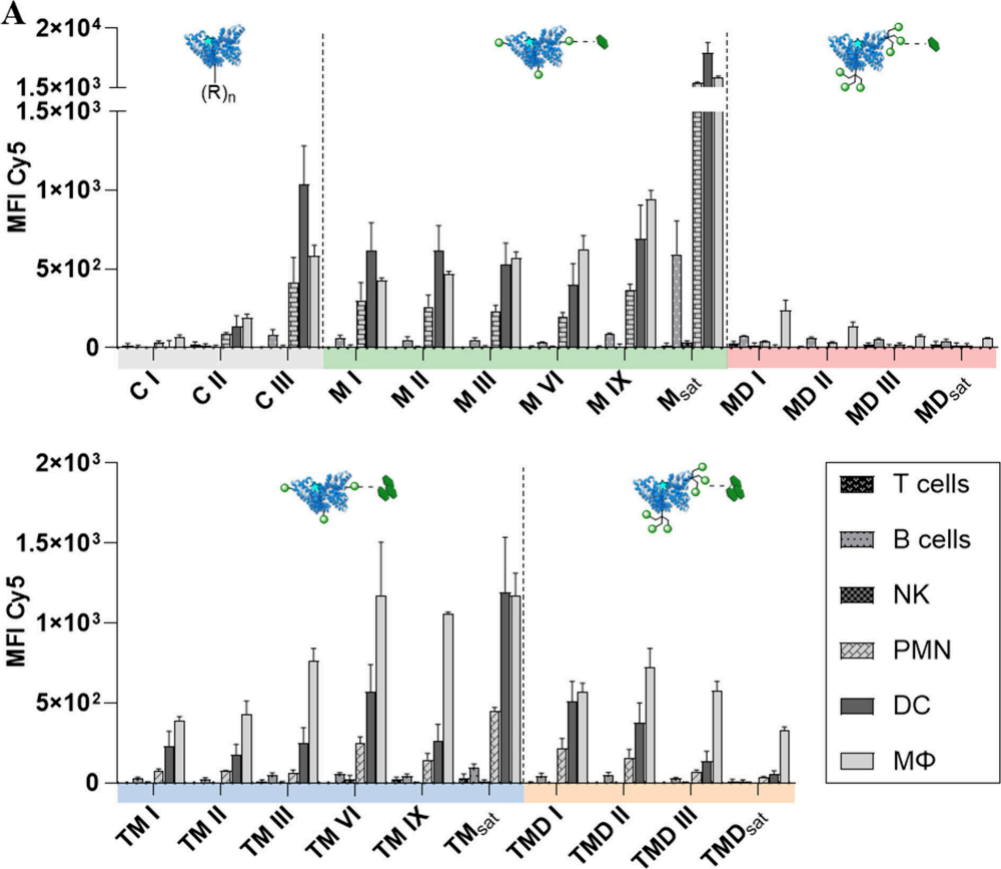
FACS analysis of cellular uptake in spleen cells after overnight incubation with different HSA conjugates (50 nM, (top) M/MD- and (bottom­( TM/TMD-HSA conjugates) differentiated into cell types (target cells: macrophages MΦ, DCs; nontarget immune cells: T cells, B cells, NK, and PMN). Data are presented as mean ± SEM, *n* = 3.

## 4 Conclusion

Within the present study, mono- and trivalent TSs, presenting the Man- and Man_3_-epitopes, were synthesized using robust and high yielding procedures. Successful conjugation using SPAAC reactions allowed the preparation of HSA-derived NCs presenting a defined number of randomly dispersed TSs. A NanoDSF investigation provided an approach to measure carbohydrate–lectin interactions in homogeneous solution, revealing binding interactions of unconjugated TSs and isolated CRDs of CD206 and CD209, with affinities in the millimolar range, consistent with earlier reports.^17,55,56^ Due to the absence of multivalent CRD presentation, no increase in avidity was observed for the trivalent TSs, stressing the requirement of membrane-like CRD presentation for the investigation of avidity effects.^127^ Thus, investigating cellular uptake of HSA conjugates using *in vitro* studies provided a realistic, biological environment to test immunocompatibility and to measure cellular uptake efficiency of the NCs under the influences of epitope type, TS surface density, clustering of epitopes, and overall valency. TS-mediated cellular uptake revealed a strong dependency from respective surface density following two trends as a result of the interplay between additional epitopes favorably contributing to and negative steric or cooperativity effects hindering binding interactions. Growing numbers of monovalent TSs lead to an increase in cellular uptake leveling out into a plateau. Growing numbers of trivalent TSs lead ultimately to a decrease in cellular uptake. Within the design of the studied conjugates, exploitation of the higher intrinsic affinity of the Man_3_- over the simpler Man-epitope is beneficial only at low surface densities, providing slight improvements in cellular uptake, with the situation reversed at saturated surface densities, due to mitigation by negative effects, potentially steric overcrowding. Furthermore, trivalent TSs cannot improve cellular uptake of the employed HSA-based NCs by induction of avidity effects due to being hampered by negative effects, especially in the saturated valency regime. To dissect the contributions of either steric overcrowding, negative cooperativity, or other unfavorable effects, which may be the cause of hampered cellular uptake upon using trivalent targeting structures, will require further investigation. Avidity effects originating from a trivalent TS carrying the Man-epitope can only be observed in the low-valency regime. Thus, utilization of these trivalent TSs seems to be beneficial in combination with low valency NCs, which offer only a few connection sites for chemical surface functionalization. In spleen cell populations, all tested TS-HSA conjugates show selectivity for CD206 and CD209 expressing target cells, namely, DCs and macrophages,^19–29^ over nontargeted splenic immune cells, with the monovalent Man showing a slightly higher off-target uptake. On a general note, for the design of targeted NCs, the optimization of TSs and surface density always needs to account for the specificities, such as size and flexibility of the NC, impacting steric hindrance and cooperativity effects. Thus, spatially demanding, multivalent TSs seem to be better adapted for use on surfaces of larger NCs, and less demanding, monovalent TSs on the surfaces of smaller NCs.^75^ A better understanding of the complex interplay between NC valency and TS surface density *in vitro* may improve the efficacy of targeted delivery approaches by providing means to influence biodistribution and cellular uptake. Thus, this investigation sheds some light on the impact of incremental changes in NC surface modification on cellular uptake and cell selectivity, allowing insights into the design of TSs and NCs.

## Supplementary Material



## Data Availability

Additional information is available from the corresponding authors on request.

## Notes

General conditions and purification methods, analytical methods, synthesis procedures, and analytical data for all steps of saccharide TS preparation and HSA conjugate preparation; Binding modes for monovalent TSs; Recombinant protein expression and purification; NanoDSF melting curves; Histograms for distance distribution of mannose epitopes in a trivalent TS; ^1^H and ^13^C NMR spectra of carbohydrate compounds; MALDI spectra of HSA conjugates (PDF)

## Abbreviations

NC: nanocarrier
TS: targeting structure
CLR: C-type lectin receptor
HSA: human serum albumin
CRD: carbohydrate recognition domain
CD206: cluster of differentiation 206
CD209: cluster of differentiation 209
NanoDSF: Nano differential scanning fluorimetry
APC: antigen-presenting cell
DC: dendritic cell
MΦ: macrophage
MMR-1: macrophage mannose receptor
DC-SIGN: dendritic cell specific ICAM-3 grabbing nonintegrin
MHC: major histocompatibility complex
Man: terminal mannose-monosaccharide
Man_3_: α-(1→3)- and α-(1→6)-branched trimannose
SPR: surface plasmon resonance
UT: untreated cells
TLR: toll-like receptor
BMDC: bone marrow-derived dendritic cell
CR: cysteine-rich domain
FNII: fibronectin type II domain
CTLD: C-type lectin domain
ND: neck domain
TM: transmembrane domain
CD: cytoplasmic domain
HPLC: high performance liquid chromatography
SPAAC: strain-promoted azide alkyne cycloaddition
DBCO: dibenzocyclooctyne
CuAAC: copper-catalyzed azide alkyne cycloaddition
TRIS: tris­(hydroxymethyl)­aminomethane
HRMS: high-resolution mass spectrometry
MALDI-ToF: matrix assisted laser desorption ionization – time of flight mass spectrometry
DLS: dynamic light scattering
C: control conjugate
M: mannose conjugate
TM: trimannose conjugate
MD: mannose glycodendron conjugate
TMD: trimannose glycodendron conjugate
FACS: fluorescence-activated cell sorting
ITC: isothermal titration calorimetry
ECD: tetrameric extracellular domain
NMR: nuclear magnetic resonance
EC_50_: half maximal effective concentration
Cy5: cyanine 5 dye
GM-CSF: Granulocyte macrophage colony-stimulating factor
MFI: mean fluorescence intensity
PMN-cell: polymorphonuclear neutrophil
NK-cell: natural killer cell
