# Supplementary material for: Surface Density of Mono- and Trivalent High-Mannan-Derived Targeting Structures with Different Affinities Impacts Cellular Uptake of Human Serum Albumin-Derived Nanocarriers
Source: Biomacromolecules. 2025 Oct 7;26(11):8087–102. doi: 10.1021/acs.biomac.5c01510 (PMC12606641; doi:10.1021/acs.biomac.5c01510)
Supplement: Supplementary file 1 [file bm5c01510_si_001.pdf]

Supporting Information

Surface Density of Mono- and Trivalent High-Mannan Derived Targeting Structures with Different Affinity Impacts Cellular Uptake of Human Serum Albumin Derived Nanocarriers

Robert Forster[†,#], Bellinda Lantzberg[‡,#], Annabelle Weldert[§,#], Laura Rosenberger[ǁ], Yanira Zeyn[ǁ], Danuta Kowalczyk[†], Seah Ling Kuan[‡], Christian Kersten[§,], Matthias Bros[ǁ,*], Tanja Weil[‡,*], Tanja Schirmeister[§,*], Till Opatz[†,*]*

[†] Department of Chemistry, Johannes Gutenberg-University Mainz, Duesbergweg 10 – 14, 55128 Mainz, Germany.

[‡] Max Planck Institute for Polymer Research, Ackermannweg 10, 55128 Mainz, Germany.

[§] Department for Pharmaceutical and Biomedical Sciences, Johannes Gutenberg-University Mainz, Staudingerweg 5, 55128 Mainz, Germany.

[ǁ] Department of Dermatology, University Medical Center of the Johannes Gutenberg-University Mainz, Obere Zahlbacher Straße 63, 55131 Mainz, Germany.

[#] Equal contribution

[*] E-mail: opatz@uni-mainz.de; schirmei@uni-mainz.de; weil@mpip-mainz.mpg.de; mbros@uni-mainz.de; kerstec@uni-mainz.de.

1 Table of contents

1 Table of contents ... 2
2 General conditions and purification methods ... 3
3 Analytical methods ... 4
4 Synthesis procedures ... 6
4.1 Synthesis of the mono- and trivalent targeting structures ... 6
4.2 Synthesis of the TS_n-HSA neoglycoalbumins ... 49
5 Prediction of binding modes for monovalent TSs ... 56
6 NanoDSF binding study for mono- and trivalent TSs ... 56
6.1 Recombinant protein expression and purification. ... 56
6.2 Differential Scanning Fluorimetry (NanoDSF). ... 58
7 Prediction of distances of mannose epitopes of a trivalent TS ... 60
8 Appendix ... 61
8.1 List of references ... 61
8.2 List of abbreviations ... 65
8.3 NMR spectra ... 67
8.4 MALDI-ToF-MS spectra ... 98

2 General conditions and purification methods

Chemicals: All chemicals were purchased from ABCR (Karlsruhe, BW, Germany), *Acros Organics* (Geel, Belgium), *Alfa Aesar* (Haverhill, MA, USA), *Arctom Scientific* (Westlake Village, CA, USA), *Carbolution* (St. Ingbert, SL, Germany), *Carl Roth* (Karlsruhe, BW, Germany), *Sigma Aldrich* (St. Louis, MO, USA), *TCI* (Tokyo, Japan), *Thermo Fisher Scientific* (Waltham, MA, USA) or *VWR* (Radnor, PA, USA) and used without further purification. Deuterated solvents for NMR experiments were obtained from *Deutero* (Kastellaun, RP, Germany) for $CDCl_3$, CD_3OD and D_2O. Deuterated chloroform was stored over molecular sieves and powdered, basic aluminum oxide (Brockmann activity I, pore size 58 Å) supplied from *Sigma Aldrich* (St. Louis, MO, USA).

Materials: PE- or PE-Cy7-labeled anti-CD11c (clone N418), eFl450- or FITC-CD86 (GL-1), PerCP-eFl710-CD80 (16-10A1), eFl506-CD3 (17A2), SB600-CD11b (M1/70), SB702-CD19 (eBio1D3), PE-NK1.1 (PK136), PE-eFl610-Ly6G (1A8-L6g), FITC-CD209a (MMD3), PE-Cy7-CD206 (C068C2), and eFl780-FVD used for flow cytometric analysis were purchased from BD Biosciences (Franklin Lakes, NJ, USA), Bio Legend (San Diego, CA, USA) or Thermo Fisher Scientific (Waltham, MA, USA).

Analytical high performance liquid chromatography (HPLC-ELS-MS): For carbohydrate synthesis monitoring of reaction progress was accomplished utilizing a 1260 Infinity II HPLC system, consisting of a 1260 Infinity Solvent rack, 1260 Infinity HiP Degasser (G4225A), 1260 Infinity Binary Pump (G1312B) operated at a flow rate of 0.7 mL/min, 1260 Infinity II Vial sampler (G7129A) adjusted to an injection volume of 1.5 µL., 1260 Infinity TCC (G1316A) operated at 40 °C, 1260 Infinity II DAD HS (G7117C) for multiple wavelength detection in combination with an 1290 Infinity II ELSD (G7102A) and an Infinity Lab 6100 Series LCMSD (G6125B) via electro spray ionization (ESI) (*Agilent Technologies Deutschland GmbH*, Waldbronn, BW, Germany). Separation was conducted using an analytical reversed phase Ascentis Express C_{18}-column (30 mm x 2.1 mm, 2.7 µm) (*Supelco Deutschland GmbH*, Bad Homburg v. d. Höhe, HE, Germany). The mobile phase was composed of a gradient mixture consisting of A = H_2O with 0.1 % HCOOH and B = MeCN using a gradient profile starting with ratio A:B = 10:90 until t = 0.2 min, followed by a linear increase to ratio A:B = 90:10 until t = 7.5 min followed by an isocratic hold until t = 10 min. Solvents were obtained from commercial suppliers in optima HPLC-MS grade quality: MeCN (*VWR*, Radnor, PA, USA), H_2O (*VWR*, Radnor, PA, USA) and HCOOH (*Thermo Fisher Scientific*, Waltham, MA, USA).

Flash column chromatography (FCC): For carbohydrate synthesis purification of larger amounts of crude products via manual normal phase flash column chromatography was accomplished with nitrogen overpressure following the method of *Still et al.*[1] Silica gel (particle size 35–70 µm) obtained from *Acros Organics* (Geel, Belgium) was utilized as stationary phase and binary, isocratic mixtures of cyclohexane (^{c}Hex) and ethyl acetate (EtOAc) as mobile phase. The solvents were obtained with "technical grade" quality and purified via distillation prior to use. Purification of small amounts of crude products via normal phase flash column chromatography was accomplished utilizing an Isolera Four Flash Purification System (*Biotage,* Uppsala, Sweden) equipped with pumps for variable gradient mixtures at flow rates of 1–200 mL/min, a DAD for detection at wavelengths between 200 – 800 nm and an integrated fraction collector. Normal phase separation was conducted using SNAP KP-SIL cartridges (*Biotage*, Uppsala, Sweden) packed with 10 g, 25 g, 50 g and 100 g of silica gel suitable for the separation of < 0.20 g, 0.20 – 0.50, 0.50 – 1.00 g or > 1.00 g of crude material. Binary eluent mixtures consisting of cyclohexane, ethyl acetate or DCM and MeOH were used. Purification of small amounts of crude products via reversed phase flash column chromatography was accomplished utilizing an

Isolera One Flash Purification System (*Biotage*, Uppsala, Sweden) equipped with pumps for variable gradient mixtures at flow rates of 1–200 mL/min, a DAD for detection at wavelengths between 200 – 400 nm and an integrated fraction collector. Reversed phase separation used SNAP C_{18} cartridges (*Biotage*, Uppsala, Sweden) packed with 12 g, 30 g and 60 g of stationary phase suitable for the separation of < 0.20 g, 0.20 – 0.50 or > 1.00 g of crude material. Binary eluent mixtures consisting of MeCN and H_2O were used. Isolation of purified material was accomplished via lyophilization utilizing an Alpha 2-4LDPlus freeze dryer (*Martin Christ Gefriertrocknungsanlagen*, Osterode, NI, Germany).

Preparative high performance liquid chromatography (HPLC): HPLC purifications were carried out using a Shimadzu HPLC system equipped with a semi preparative reversed phase Eclipse XBD-C_{18}-column (9.4 mm x 250 mm, 5 µm) (*Agilent Technologies Deutschland GmbH*, Waldbronn, BW, Germany).

Thin layer chromatography (TLC): For carbohydrate synthesis monitoring of reaction progress and chromatographic separation via normal phase TLC was conducted using silica gel 60 F_{254} modified aluminum plates and reversed phase TLC on silica gel 60 RP-18 F_{254S} modified aluminum plates (*Merck KGaA*, Darmstadt, Germany) containing manganese doted zinc silicate for detection upon UV irradiation. Mobile phases consisted of binary eluent mixtures as described below with the composition stated as volumetric ratio (*v*:*v*). For further substance detection the following staining reagents were applied, and the plates heated with a heat gun: "Potassium permanganate staining reagent": $KMnO_4$ (1.00 g), K_2CO_3 (7.00 g) dissolved in H_2O (100 mL) adding a NaOH-solution (5 %, 2.00 mL) and "Carbohydrate staining reagent": 3-Methoxyphenol (0.20 mL) dissolved in EtOH (100 mL) adding conc. H_2SO_4 (7.00 mL). Ratio of Fronts values (R_f) were stated for each compound.

3 Analytical methods

Absorbance spectroscopy measurements: The absorbance was measured using a NANODROP 2000c spectrophotometer (*Thermo Fisher scientific*, Waltham, MA, USA) or Spark 20M microplate reader (*Tecan*, Wiesbaden, HE, Germany).

High resolution mass spectrometry (HR-ESI-MS): HR-ESI-MS was recorded using a Synapt G2-Si mass spectrometer (*Waters Corporation*, Eschborn, HE, Germany). High resolution exact mass determination for carbohydrate structures was performed using a G6545A Q-ToF (*Agilent Technologies Deutschland GmbH*, Waldbronn, BW, Germany) via electro spray ionization (ESI). Sample injection was achieved using a 1260 Infinity II HPLC-System (*Agilent Technologies Deutschland GmbH*, Waldbronn, BW, Germany) with a G7111B 1260 Quaternary Pump, G7129A 1260 Vialsampler und G7116A 1260 Multicolumn Thermostat. Mass calibration was done on the day of sample measurement utilizing an external standard.

Infrared spectroscopy (IR): Infrared spectra were measured utilizing a Tensor 27 FT-IR spectrometer with built in Diamant-ATR-unit (*Bruker Corporation*, Ettlingen, BW, Germany). The absorption frequencies of characteristic oscillation bands are stated as wave number $\tilde{\nu}$ [cm^{-1}]. Spectra were processed using the Opus software (*Bruker Corporation*, Ettlingen, BW, Germany) for baseline correction and peak detection.

Melting range determination: For carbohydrate compounds obtained as crystalline solid the melting range Θ [°C] was determined using a KSP1N melting point meter (*A. Krüss Optronic*, Hamburg, HH, Germany). Samples were transferred into a capillary tube and heated with a rate of 1 °C/min. The melting process was observed and temperature values for onset and end of melting are given.

Nuclear magnetic resonance spectroscopy (NMR): For carbohydrate structures NMR-spectra were recorded utilizing an AC300 (*Bruker Corporation*, Ettlingen, BW, Germany) with 5 mm Dual-^{13}C-head and B-ACS 60 sample changer for 300 MHz ^{1}H-NMR-, 75.5 MHz ^{13}C-NMR- as well as 2D-NMR-experiments, an Avance-II 400 (*Bruker Corporation*, Ettlingen, BW, Germany) with 5 mm BBFO-head with z-gradient and ATM as well as SampleXPress 60 sample changer for 400 MHz ^{1}H-NMR-, 100.6 MHz ^{13}C-NMR- and 2D-NMR experiments and an Avance-III 600 (*Bruker Corporation*, Ettlingen, BW, Germany) with 5 mm TCI-Cryoprobe with z-Gradient and ATM as well as SampleXPress Lite 16 sample changer for 600 MHz ^{1}H-NMR-, 151.0 MHz ^{13}C-NMR- and 2D-NMR experiments. Samples were dissolved in an appropriate deuterated solvent ($CDCl_3$, CD_3OD, D_2O). Spectra were processed using the MestReNova software (*Mestrelab Research*, Santiago de Compostela, Spain) for baseline correction (Whittaker smoother) and automated phase correction. Spectra were referenced to the signal of the respective deuterated solvent ($CDCl_3$: ^{1}H: $\delta = 7.26$ ppm, CD_3OD: ^{1}H: $\delta = 3.31$ ppm, D_2O: ^{1}H: $\delta = 4.79$ ppm) and using the absolute reference function of the analysis software. Observed signals are described using their chemical shifts (δ) in ppm, multiplicity (s-singlet, d-doublet, t-triplet, dd-double of doublet, etc. and m-multiplet; app-apparent and br-broad), integral and locator of ^{1}H- and ^{13}C-position in the molecule. Signal assignment was supported utilizing 2D-NMR spectra: COSY, HSQC, HSQC-NoDec, HMBC, TOCSY, NOESY (data not shown).

Polarimetry: For carbohydrate compound specific optical rotation values $[\alpha]_{\mathrm{D}}^{\theta}$ of all optical active carbohydrate compounds were determined using an automated 241 MC polarimeter (*PerkinElmer Inc.*, Waltham, MA, USA). Measurements were undertaken utilizing a sodium-vapor lamp at the sodium D-double line ($\lambda_{D1} = 589.5924$ nm und $\lambda_{D2} = 588.9951$ nm) with an aperture adjustment of 600 nm and a lid width of 2 nm. Optical rotation values α were determined at an integration time of 20 seconds. Measurements were carried out at ambient temperature with the actual value stated for each optical rotation value. Glass cuvettes ($l = 10$ cm, $V = 1.00$ mL) were filled with a sample solution ($c = 0.01$ g/mL) in an appropriate solvent ($CHCl_3$, MeOH, H_2O). The polarimeter was calibrated with the pure solvent prior to the measurement. The unit of the specific optical rotation $[\alpha]_{\lambda}^{\theta}$ of [°·cm^2/10·g] is neglected in the following.

4 Synthesis procedures

4.1 Synthesis of the mono- and trivalent targeting structures

The synthesis of the mannose-based saccharide building blocks (**1**) and (**2**), the HOOC-PEG$_3$-N$_3$ building block (**3**), the H$_2$N-PEG$_4$-N$_3$ building block (**4**), the N$_3$-PEG$_4$-N$_3$ building block (**5**), and the trivalent pre-dendron (**6**) is summarized in Scheme S 1. The sequences include a total of 15 reaction steps involving three telescoped reaction procedures reducing needed purification steps and improving overall yield. Starting from D-mannose, glycosyl donor (**S1**) was prepared via a telescoped, two-step procedure involving a peracetylation using Ac$_2$O and pyridine[2, 3] as well as a glycosylation using PhSH and BF$_3$·OEt$_2$ in DCM.[4, 5] Furthermore, the monosaccharide building block ManPrg (**2**) was prepared via a telescoped, three-step procedure involving a peracetylation using Ac$_2$O and pyridine, a glycosylation using PrgOH and BF$_3$·OEt$_2$ in DCM as well as a Zemplén deacylation utilizing NaOMe in MeOH.[4, 6-13] Starting from this deprotected building block, glycosyl acceptor (**S2**) was prepared via a telescoped, three-step procedure involving a di-silylation using TBDMSCl and imidazole in DMF, a di-benzoylation using BzCl and DMAP in a mixture of DCM and pyridine as well as a di-desilylation using (HF)$_X$·pyridine complex in AcOH and THF.[14-22] Glycosylation of glycosyl donor (**S1**) with glycosyl acceptor (**S2**) using a promotor system consisting of NIS and AgOTf in DCM delivered the trisaccharide building block Man$_3$Prg (**1**).[18, 19, 23] Starting with PEG$_4$, the N$_3$-PEG$_4$-N$_3$ building block (**5**) was obtained via a di-mesylation using MsCl and NEt$_3$ in DCM followed by a nucleophilic substitution using NaN$_3$ and TBAI in DMF.[6, 24-26] Staudinger reduction of one azide group using one equivalent of PPh$_3$ in a mixture of Et$_2$O, THF am 1 M HCl gave access to H$_2$N-PEG$_4$-N$_3$ building block (**4**).[24, 26] Starting with PEG$_3$, the protected precursor (**S3**) was prepared via a Micheal addition using TBA and Na in THF followed by a tosylation using TsCl and NEt$_3$ in DCM.[26-28] The HOOC-PEG$_3$-N$_3$ building block (**3**) was obtained via a nucleophilic addition using NaN$_3$ in DMF followed by cleavage of the tert-butyl ester using TFA in DCM.[26, 27, 29] Starting with TRIS, a Micheal addition using TBA and 5 M NaOH in DMSO yields the Lin's amine analogue (**S4**).[26, 30-32] Protection of the amine using the respective chloroformate ester, derived from *o*-NBA and TCF, gave access to the orthogonally protected precursor (**S5**).[26, 33, 34] Cleavage of the *tert*-butyl esters using TFA in DCM delivered the tricarboxylic acid (**S6**).[26, 29, 35] Finally, an amide coupling reaction using HATU and DIPEA in DMF allowed to connect the H$_2$N-PEG$_4$-N$_3$ building block (**4**) to obtain pre-dendron (**6**). The assembly of monovalent and trivalent TSs is summarized in Scheme S 2. The synthesis sequences include a total of 11 reaction steps to obtain all four TSs using similar reaction conditions. Synthesis of the TS ManN$_3$ was achieved by connecting the ManPrg (**2**) and the N$_3$-PEG$_4$-N$_3$ building blocks (**5**) via a CuAAC-reaction using Cu^{I}Br and PMDTA in DMF.[36-40] ManN$_3$ (**1**) was obtained with a total yield of 91 % over the longest linear sequence starting from HOOC-PEG$_4$-N$_3$ (**3**) containing three steps. The assembly of the TS Man$_3$N$_3$ was achieved by connecting the Man$_3$Prg (**1**) and the diazide tetraethylene glycol building blocks (**5**) via a CuAAC-reaction using Cu^{I}Br and PMDTA in DMF followed by a global Zemplén-deacylation using NaOMe and NaOH in MeOH.[6, 39-41] Man$_3$N$_3$ was obtained with a total yield of 64 % over the longest linear sequence starting from D-mannose containing five steps. The assembly of the trivalent glycodendrons of the Newkome-type[32] (Man)$_3$N$_3$ and (Man$_3$)$_3$N$_3$ was accomplished by connecting the respective fully protected building blocks (**S7**), which can be obtained by isolating the intermediat during the telescoped synthesis of the deprotected building block ManPrg (**2**) prior to the Zemplén-deacylation, and Man$_3$Prg (**1**) with pre-dendron (**6**) via a CuAAC-reaction using Cu^{I}Br and PMDTA in DMF resulting in (**S8**) and (**S9**).[26, 39] UV-A-Irradiation at an emission maximum of 350 nm allowed photolysis of the

photocleavable NBA-carbamate in context of a Norrish-type-II fragmentation giving access to the deprotected amines (**S10**) and (**S11**).[26, 42, 43]

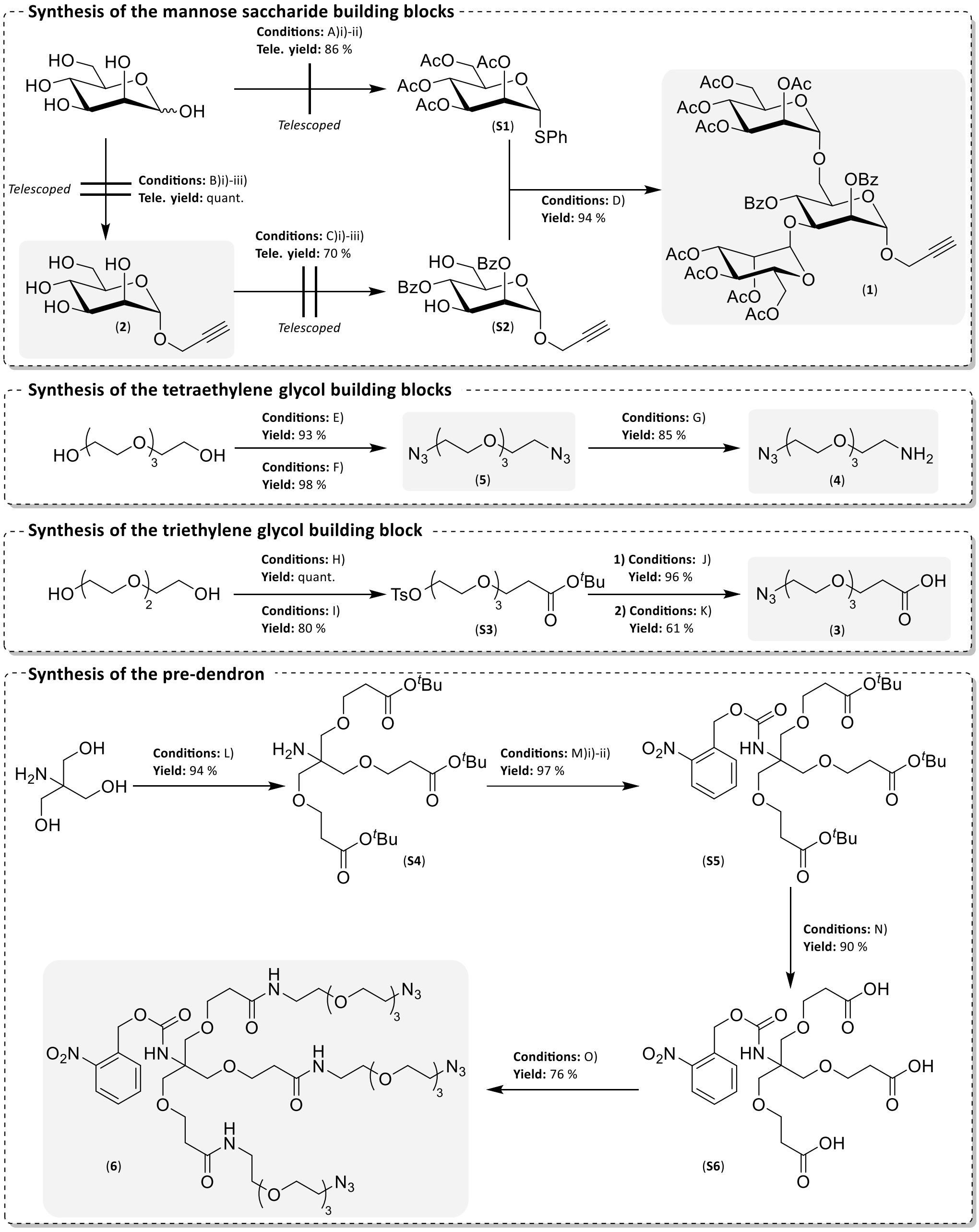

Scheme S 1: Synthesis of the mannose-based saccharide building blocks ManPrg (**2**) and Man_3Prg (**1**), H_2N-PEG_4-N_3 building block (**4**), HOOC-PEG_4-N_3 building block (**3**) and of the trivalent pre-dendron (**6**). The building blocks used to assemble the TSs are highlighted in grey. Reaction conditions: A)i) Ac_2O, pyr., Ar-atm., rt, 20 h; A)ii) $BF_3{\cdot}OEt_2$, PhSH, DCM, Ar-atm., 0 °C to rt, 24 h; B)i) Ac_2O, pyr., Ar-atm., rt, 20 h; B)ii) $BF_3{\cdot}EtO_2$, HOPrg, DCM, Ar-atm., 0 °C to rt, 20 h; B)iii) NaOMe, MeOH, Ar-atm., rt, 16 h; C)i) TBDMSCl, Imi., DMF, Ar-atm., MS 3Å, 0 °C, 4 h; C)ii) BzCl, pyr., DMAP, DCM, Ar-atm., MS 3Å, 0 °C to rt, 42 h; C)iii) $(HF)_X$·pyr., AcOH, THF, 0 °C to rt, 24 h; D) NIS, AgOTf, DCM, Ar-atm., -40 °C to -20 °C, 4 h; E) MsCl, NEt_3, DCM, Ar-atm., 0 °C to rt, 3 h; F) NaN_3, TBAI, DMF, Ar-atm., 80 °C, 4 h; G) PPh_3, Et_2O/THF/1 M HCl (8:1:4), rt, 48 h; H) TBA, Na, THF, Ar-atm., rt, 22 h; I) TsCl, NEt_3, DCM, Ar-atm., rt, 23 h; J) NaN_3, DMF, Ar-atm., rt, 21 h; K) TFA, DCM, Ar-atm., rt, 22 h; L) TBA, NaOH (5M), DMSO, Ar-atm., 0 °C to rt, 96 h; M)i) NBA, TCF, THF, 66 °C, 4 h; M)ii) $NaHCO_3$, EtOAc/H_2O (2:1), rt, 18 h; N) TFA, DCM, rt, 72 h; O) (**4**), HATU, DIPEA, DMF, Ar-atm., rt, 48 h.

Via an amide coupling reaction using HATU and DIPEA in DMF the HOOC-PEG_4-N_3 building block (**3**) was connected to obtain the precursors (**S12**) and (**S13**).[26, 44] A global Zemplén-deacylation using NaOMe and NaOH in MeOH gave access to the deprotected glycodendrimers $(Man)_3N_3$ and $(Man_3)_3N_3$.[11, 26] Both structures were obtained with a total yield of 38 % and 25 % respectively over the

longest linear sequence starting from tetraethylene glycol containing eight steps. Analytically pure samples of the intermediate products of telescope steps were isolated and used for characterization.

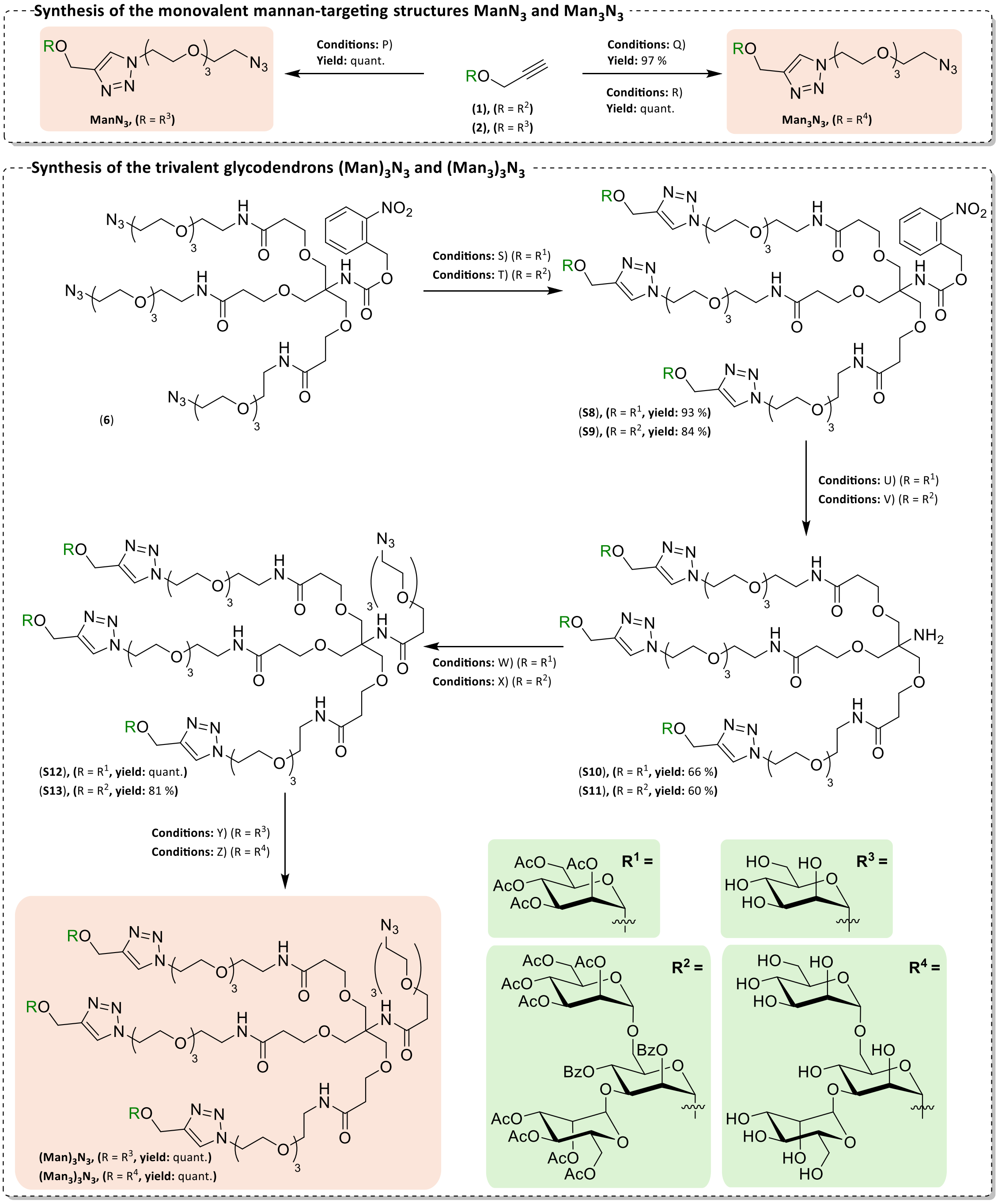

Scheme S 2: Assembly of the monovalent TSs $ManN_3$ and Man_3N_3 as well as of the trivalent glycodendrons $(Man)_3N_3$ and $(Man_3)_3N_3$. The mannose residues of the TSs are abbreviated and highlighted in green. The TSs used for NanoDSF studies and conjugation to the HSA-NC are highlighted in orange. Reaction conditions: P) Cu^IBr, NaAsc, DMF, Ar-atm., 45 °C, 20 h; Q) Cu^IBr, PMDTA, DMF, Ar-atm., 45 °C, 3 h; R) NaOMe, NaOH, MeOH, Ar-atm., rt, 24 h; S) (**S7**), Cu^IBr, PMDTA, DMF, Ar-atm., 45 °C, 4 h; T) (**1**), Cu^IBr, PMDTA, DMF, Ar-atm., 45 °C, 5 h; U) UV-A, MeCN, Ar-atm., rt, 15 h; V) UV-A, MeCN, Ar-atm., rt, 15 h; W) (**3**), HATU, DIPEA, Ar-atm., DMF, rt, 48 h; X) (**3**), HATU, DIPEA, Ar-Atm., DMF, rt, 48 h; Y) NaOMe, MeOH, Ar-atm., rt, 2 h; Z) NaOMe, NaOH, MeOH, Ar-atm., rt, 18 h.

4.1.1 Synthesis of Phenyl 2,3,4,6-Tetra-*O*-acetyl-1-thio-α-D-mannopyranoside

The title compound was synthesized utilizing a telescoped two-step protocol according to Scheme S 3 following synthesis protocols by *Krabicová et al.*[13] and *Ekholm et al.*[4]

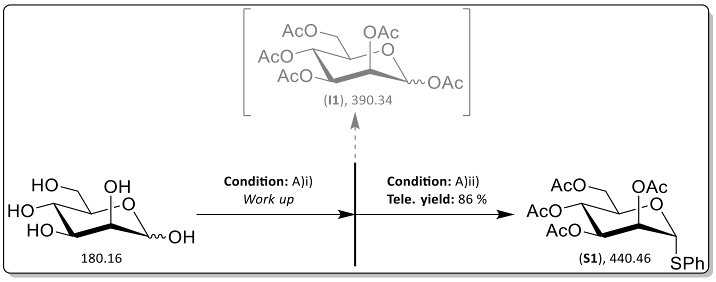

Scheme S 3: Synthesis of Phenyl 2,3,4,6-Tetra-*O*-acetyl-1-thio-α-D-mannopyranoside (**S1**). Reaction conditions A)i) Ac_2O, Pyr., Ar-atm., rt, 20 h; A)ii) $BF_3{\cdot}OEt_2$, PhSH, DCM, Ar-atm., 0 °C to rt, 24 h; telescoped yield: 86 %.

Both reaction steps were carried out in dried Schlenk vessels, which were previously equipped with magnetic stirring bars. D-Mannose (2.00 g, 11.1 mmol, 1.00 eq.) was dissolved in absolute pyridine (10.0 mL) under an atmosphere of argon and the slightly turbid solution was cooled to a temperature of 0 °C in an ice bath. Ac_2O (11.5 g, 10.5 mL, 111 mmol, 10.0 eq.) was added with a syringe. The yellowish reaction mixture was stirred for 20 hours while slowly warming up to room temperature. Complete conversion of the limiting substrate and formation of the desired product were determined by reaction control via TLC and HPLC-ELS-MS. The mixture was poured onto H_2O (1 x 100 mL) and extracted with EtOAc (3 x 100 mL). The collected organic phases were washed with saturated $NaHCO_3$ solution (3 x 100 mL), 1M HCl solution (3 x 100 mL), H_2O (3 x 100 mL) and saturated NaCl solution (1 x 100 mL). The organic phase was dried over anhydrous Na_2SO_4 and filtered. All volatile components were removed in vacuo. Toluene (3 x 20.0 mL) was added to the residue and all volatile components were again removed in vacuo. The residue was dried in fine vacuum. The first intermediate (4.60 g) was obtained in the form of colorless oil and dissolved in absolute DCM (20.0 mL) under an atmosphere of argon. PhSH (1.96 g, 1.81 mL, 17.8 mmol, 1.60 eq.) was added with a syringe and molecular sieves (MS 3Å, 0.50 g) were added successively. The mixture was stirred for 30 minutes at room temperature. The brownish solution was cooled to a temperature of 0 °C in an ice bath and $BF_3{\cdot}OEt_2$ (7.88 g, 7.03 mL, 55.5 mmol, 5.00 eq.) was added dropwise. The resulting yellowish reaction mixture was stirred for 24 hours while slowly warming up to room temperature, with a deep red solution resulting. Complete conversion of the limiting substrate and formation of the desired product were determined by reaction control via TLC and HPLC-ELS-MS. The mixture was diluted with DCM (1 x 100 mL) and poured onto ice-cold H_2O (1 x 150 mL) with stirring. The organic phase was washed with saturated $NaHCO_3$ solution (1 x 100 mL) and added to an ice-cooled 1 M NaOH solution (1 x 150 mL) while stirring vigorously. The two-phase mixture was stirred for one hour at a temperature of 0 °C. The organic phase was washed again with saturated $NaHCO_3$ solution (1 x 150 mL), 1 M NaOH solution (1 x 150 mL) and saturated NaCl solution (1 x 150 mL), dried over anhydrous Na_2SO_4 and filtered. All volatile components were removed in vacuo and the residue was dried in fine vacuum. The crude product (6.35 g) was obtained in the form of an orange oil and purified by FCC (^{c}Hex/EtOAc, isocratic at 33 % EtOAc). The pure product (**S1**) was obtained as α-anomer in the form of a colorless solid.

Yield: 4.21 g (9.55 mmol, 86 %), (Lit.[4]: 95 %), colorless solid.

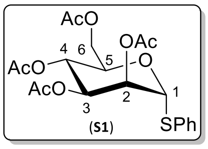

^{1}H-NMR (400 MHz, $CDCl_3$, 25 °C)**:** *δ* (ppm) = 7.51–7.47 (m, 2H, C-1-S-Ph, H_{ortho}), 7.35–7.28 (m, 3H, C-1-S-Ph, H_{meta}, H_{para}), 5.55 (dd, ^3J = 3.1, 1.6 Hz, 1H, H-2), 5.49 (d, ^3J = 1.6 Hz, 1H, H-1), 5.33 (dd, ^3J = 9.9, 9.8 Hz, 1H, H-4), 5.32 (dd, ^3J = 9.8, 3.1 Hz, 1H, H-3), 4.55 (ddd, ^3J = 9.9, 5.9, 2.4 Hz, 1H, H-5), 4.31 (dd,

^2J = 12.2, ^3J = 5.9 Hz, 1H, H-6a), 4.10 (dd, ^2J = 12.2, ^3J = 2.4 Hz, 1H, H-6b), 2.15 (s, 3H, C-4-O(C=O)C*H*$_3$), 2.07 (s, 3H, C-6-O(C=O)C*H*$_3$), 2.05 (s, 3H, C-3-O(C=O)C*H*$_3$), 2.02 (s, 3H, C-2-O(C=O)C*H*$_3$). **^{13}C-NMR** (101 MHz, $CDCl_3$, 25 °C)**:** δ (ppm) = 170.5 (1C, C-6-O(*C*=O)CH_3), 169.9 (1C, C-3-O(*C*=O)CH_3), 169.8 (1C, C-4-O(*C*=O)CH_3), 169.7 (1C, C-2-O(*C*=O)CH_3), 132.6 (1C, C-1-SPh, C_{ipso}), 132.1 (2C, C-1-SPh, C_{ortho}), 129.2 (2C, C-1-SPh, C_{meta}), 128.1 (1C, C-1-SPh, C_{para}), 85.7 (1C, C-1), 70.9 (1C, C-2), 69.5 (1C, C-5) 69.4 (1C, C-3), 66.4 (1C, C-4), 62.5 (1C, C-6), 20.9 (1C, C-4-O(C=O)*C*H_3), 20.7 (1C, C-6-O(C=O)*C*H_3), 20.7 (1C, C-3-O(C=O)*C*H_3), 20.7 (1C, C-2-O(C=O)*C*H_3). **HR-MS** (ESI$^+$)**:** $m/z_{cal.}$ = 463.1033 [M+Na]$^+$, $m/z_{exp.}$ = 463.1031 [M+Na]$^+$; $m/z_{cal.}$ = 331.1029 [M-SPh]$^+$, $m/z_{exp.}$ = 331.1022 [M-SPh]$^+$. ***R_f*** (NP) = 0.33 (^{c}Hex/EtOAc 2:1). **Melting range:** Θ (°C) = 79.5 – 81.1 (EtOAc). **Optical rotation** (LM)**:** $[\alpha]_D^{21}$ = +104 ($CHCl_3$). **IR** (ATR)**:** $\tilde{\nu}$ (cm^{-1}) = 1745, 1440, 1369, 1221, 1106, 1050, 976, 915, 749, 692.

The analytical data follows literature data.[4]

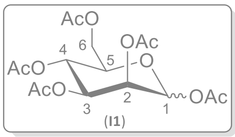

(I1)

NMR-signals of major α-anomer: ^{1}H-NMR (400 MHz, $CDCl_3$, 25 °C)**:** δ (ppm) = 6.07 (d, ^3J = 2.0 Hz, 1H, H-1), 5.35 – 5.32 (m, 2H, H-2, H-3), 5.26 – 5.23 (m, 1H, H-4), 4.27 (dd, ^2J = 12.4, ^3J = 4.9 Hz, 1H, H-6a), 4.08 (dd, ^2J = 12.4, ^3J = 2.4 Hz, 1H, H-6b), 4.06 – 4.01 (m, 1H, H-5), 2.16 (s, 3H, C-4-O(C=O)C*H*$_3$), 2.15 (s, 3H, C-1-O(C=O)C*H*$_3$), 2.08 (s, 3H, C-6-O(C=O)C*H*$_3$), 2.04 (s, 3H, C-2-O(C=O)C*H*$_3$), 1.99 (s, 3H, C-3-O(C=O)C*H*$_3$). **^{13}C-NMR** (101 MHz, $CDCl_3$, 25 °C)**:** δ (ppm) = 170.7 (C-6-O(*C*=O)CH_3), 170.1 (C-3-O(*C*=O)CH_3), 169.8 (C-4-O(*C*=O)CH_3), 169.6 (C-2-O(*C*=O)CH_3), 168.2 (C-1-O(*C*=O)CH_3), 90.7 (C-1), 70.7 (C-5), 68.8 (C-3), 68.4 (C-2), 65.6 (C-4), 62.2 (C-6), 21.0 (C-4-O(C=O)*C*H_3), 20.9 (C-1-O(C=O)*C*H_3), 20.8 (C-6-O(C=O)*C*H_3), 20.8 (C-2-O(C=O)*C*H_3), 20.7 (C-3-O(C=O)*C*H_3). **NMR-signals of minor β-anomer: ^{1}H-NMR** (400 MHz, $CDCl_3$, 25 °C)**:** δ (ppm) = 5.85 (d, ^3J = 1.2 Hz, 1H, H-1), 5.47 (dd, ^3J = 3.3, 1.2 Hz, 1H, H-2), 5.31–5.27 (m, 1H, H-4), 5.12 (dd, ^3J = 10.0, 3.3 Hz, 1H, H-3), 4.29 (dd, ^2J = 12.3 Hz, ^3J = 5.3 Hz, 1H, H-6a), 4.14 (dd, ^2J = 12.3 Hz, ^3J = 2.4 Hz, 1H, H-6b), 3.79 (ddd, ^3J = 9.9, 5.3, 2.4 Hz, 1H, H-5), 2.20 (s, 3H, C-4-O(C=O)C*H*$_3$), 2.09 (s, 3H, C-1-O(C=O)C*H*$_3$), 2.08 (s, 3H, C-6-O(C=O)C*H*$_3$), 2.04 (s, 3H, C-2-O(C=O)C*H*$_3$), 1.99 (s, 3H, C-3-O(C=O)C*H*$_3$). **^{13}C-NMR** (101 MHz, $CDCl_3$, 25 °C)**:** δ (ppm) = 170.7 (1C, C-6-O(*C*=O)-CH_3), 170.3 (1C, C-3-O(*C*=O)-CH_3), 169.9 (1C, C-4-O(*C*=O)-CH_3), 169.7 (1C, C-2-O(*C*=O)-CH_3), 168.5 (1C, C-1-O(*C*=O)-CH_3), 90.5 (1C, C-1), 73.4 (1C, C-5), 70.7 (1C, C-3), 68.3 (1C, C-2), 65.5 (1C, C-4), 62.2 (1C, C-6), 21.0 (1C, C-4-O(C=O)*C*H_3), 20.9 (1C, C-1-O(C=O)*C*H_3), 20.8 (1C, C-6-O(C=O)*C*H_3), 20.8 (1C, C-2-O(C=O)*C*H_3), 20.6 (1C, C-3-O(C=O)*C*H_3). **Analytical data of obtained anomeric mixture (α:β 4.2:1): HR-MS** (ESI$^+$)**:** $m/z_{cal.}$ = 413.1054 [M+Na]$^+$, $m/z_{exp.}$ = 413.1058 [M+Na]$^+$. ***R_f*** (NP) = 0.45 (^{c}Hex/EtOAc 1:1), 0.25 (^{c}Hex/EtOAc 2:1). **Melting range:** Θ (°C) = 60.0 – 61.0 (DCM). **Optical rotation** (LM)**:** $[\alpha]_D^{21}$ = +48.0 ($CHCl_3$). **IR** (ATR)**:** (cm^{-1}) = 2991, 1745, 1434, 1370, 1213, 1148, 1088, 1052, 1026, 974, 912, 786, 732, 686, 601, 562, 500, 467, 449.

The analytical data follows literature data.[6, 11, 45, 46]

4.1.2 Synthesis of Propargyl α-D-Mannopyranoside

The title compound was synthesized utilizing a telescoped three-strep protocol according to Scheme S 4 following synthesis protocols by *Krabicová et al.*[13], *Poláková et al.*[10] and *Reintjens et al.*[12].

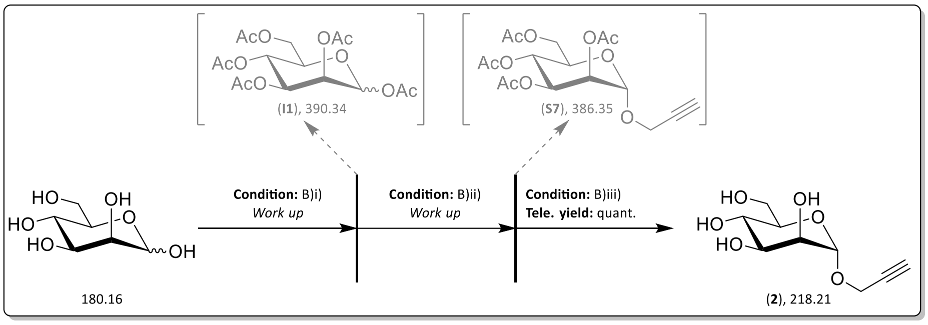

Scheme S 4: Synthesis of Propargyl α-D-Mannopyranoside (**2**). Reaction conditions: B)i) Ac_2O, Pyr., Ar-atm., rt, 20 h; B)ii) $BF_3{\cdot}EtO_2$, HOPrg, DCM, Ar-atm., 0 °C to rt, 20 h; B)iii) NaOMe, MeOH, Ar-atm., rt, 16 h; telescoped yield: quant.

All three reaction steps were carried out in dried Schlenk vessels, which were previously equipped with magnetic stirring bars. D-Mannose (1.00 g, 5.55 mmol, 1.00 eq.) was dissolved in absolute pyridine (5.00 mL) under an atmosphere of argon and the slightly turbid solution was cooled to a temperature of 0 °C in an ice bath. Ac_2O (5.67 g, 5.23 mL, 55.5 mmol, 10.0 eq.) was added with a syringe. The yellowish reaction mixture was stirred for 20 hours while slowly warming up to room temperature. Complete conversion of the limiting substrate and formation of the desired product were determined by reaction control via TLC and HPLC-ELS-MS. The mixture was poured onto H_2O (1 x 50.0 mL) and extracted with EtOAc (3 x 50.0 mL). The collected organic phases were washed with saturated $NaHCO_3$ solution (3 x 50.0 mL), 1M HCl solution (3 x 50.0 mL), H_2O (3 x 50.0 mL) and saturated NaCl solution (1 x 50.0 mL). The organic phase was dried over anhydrous Na_2SO_4 and filtered. All volatile components were removed in vacuo. Toluene (3 x 10.0 mL) was added to the residue and all volatile components were again removed in vacuo. The residue was dried in fine vacuum. The first intermediate (2.23 g) was obtained in the form of colorless oil and dissolved in absolute DCM (20.0 mL) under argon atmosphere. The colorless solution was cooled to a temperature of 0 °C in an ice bath. PrgOH (1.56 g, 1.60 mL, 27.8 mmol, 5.00 eq.) and $BF_3{\cdot}OEt_2$ (7.88 g, 7.03 mL, 55.5 mmol, 10.0 eq.) were added successively with a syringe. The resulting yellowish reaction mixture was stirred for 20 hours with slow warming to room temperature, during which time the solution turned dark red. Complete conversion of the limiting substrate and formation of the desired product were determined by reaction control via TLC and HPLC-ELS-MS. The mixture was diluted with DCM (1 x 100 mL) and washed with H_2O (3 x 100 mL), saturated $NaHCO_3$ solution (3 x 100 mL) and saturated NaCl solution (1 x 100 mL). The organic phase was dried over anhydrous Na_2SO_4 and filtered. All volatile components were removed in vacuo. The second intermediate (2.25 g) was obtained in the form of yellowish oil and dissolved in a mixture of absolute DCM (5.00 mL) and absolute MeOH (20.0 mL) under argon atmosphere. NaOMe (71.0 mg, 2.22 mmol, 0.40 eq.) was added in argon countercurrent and the reaction mixture was stirred for 20 hours at room temperature. Complete conversion of the limiting substrate and formation of the desired product were determined by reaction control via TLC and HPLC-ELS-MS. The reaction was terminated by adding the ion exchange resin Amberlite IR 120 (200 mg). The mixture was stirred for ten minutes until a constant pH-value (pH = 6 – 7) was reached. The reaction mixture was filtered over Celite through a glass frit and eluted with MeOH (1 x 100 mL). All volatile components were removed in vacuo and the residue was dried in fine vacuum. The crude product (1.54 g) was obtained in the form of a yellowish foam, dissolved in H_2O (1 x 50.0 mL) and extracted with a mixture of ^{c}Hex and EtOAc (2 x 50.0 mL, 10:1v:v) and purified after lyophilization of the aqueous phase by FCC (liquid feed from

MeOH, DCM/MeOH, gradient from 0 % to 20 % MeOH, Isolera Four Purification System, SNAP KP-Sil 100 g cartridge). The pure product (**2**) was obtained as an α-anomer in the form of a colorless solid.

Yield: 1.21 g (5.55 mmol, quant.), (Lit.[6, 11]: 98 %), colorless solid.

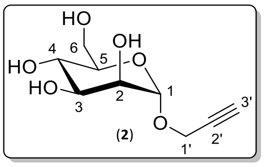

^{1}H-NMR (400 MHz, CD_3OD, 25 °C)**:** *δ* (ppm) = 4.96 (d, ^3J = 1.7 Hz, 1H, H-1), 4.27 (d, ^4J = 2.4 Hz, 2H, H-1‘), 3.84 (dd, ^2J = 11.8, ^3J = 2.3 Hz, 1H, H-6a), 3.79 (dd, ^2J = 3.2, 1.7 Hz, 1H, H-2), 3.70 (dd, ^2J = 11.8 Hz, ^3J = 5.9 Hz, 1H, H-6b), 3.67 (dd, ^3J = 9.1, 3.2 Hz, 1H, H-3), 3.62 (t_{app}, ^3J = 9.4 Hz, 1H, H-4), 3.51 (ddd, ^3J = 8.8, 5.9, 2.3 Hz, 1H, H-5), 2.86 (t, ^4J = 2.4 Hz, 1H, H-3‘). **^{13}C-NMR** (101 MHz, CD_3OD, 25 °C)**:** *δ* (ppm) = 99.8 (1C, C-1), 80.0 (1C, C-2‘), 76.0 (1C, C-3‘), 75.1 (1C, C-5), 72.5 (1C, C-3), 72.0 (1C, C-2), 68.5 (1C, C-4), 62.8 (1C, C-6), 54.8 (1C, C-1‘). **HR-MS** (ESI$^+$)**:** $m/z_{cal.}$ = 241.0683 [M+Na]$^+$, $m/z_{exp.}$ = 241.0682 [M+Na]$^+$. ***R$_f$*** (NP) = 0.13 (DCM/MeOH 10:1). **Melting range:** Θ (°C) = 119.0 – 120.1 °C (H_2O). **Optical rotation** (LM): $[\alpha]_D^{20}$ = +116.7 ° (MeOH). **IR** (ATR): (cm^{-1}) = 3288, 2929, 2118, 1599, 1351, 1213, 1131, 1058, 1008, 970, 919, 881, 813, 683. The analytical data follows literature data.[6, 11, 12]

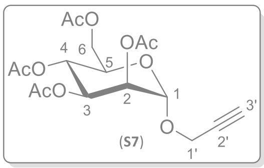

^{1}H-NMR (400 MHz, $CDCl_3$, 25 °C)**:** *δ* (ppm) = 5.35 (dd, ^3J = 9.5, 3.2 Hz, 1H, H-3), 5.31 (dd, ^3J = 9.5, 9.3 Hz, 1H, H-4), 5.28 (dd, ^3J = 3.2, 1.7 Hz, 1H, H-2), 5.03 (d, ^3J = 1.7 Hz, 1H, H-1), 4.29 (dd, ^2J = 12.3 Hz, ^3J = 5.1 Hz, 1H, H-6a), 4.27 (d, ^2J = 12.6 Hz, ^4J = 2.4 Hz, 2H, H-1‘), 4.11 (dd, ^2J = 12.3, ^3J = 2.5 Hz, 1H, H-6b), 4.02 (ddd, ^3J = 9.3, 5.1, 2.5 Hz, 1H, H-5), 2.47 (t, ^4J = 2.4 Hz, 1H, H-3‘), 2.16 (s, 3H, C-3-O(C=O)C*H*$_3$), 2.11 (s, 3H, C-6-O(C=O)C*H*$_3$), 2.04 (s, 3H, C-2-O(C=O)C*H*$_3$), 1.99 (s, 3H, C-4-O(C=O)C*H*$_3$). **^{13}C-NMR** (101 MHz, $CDCl_3$, 25 °C)**:** *δ* (ppm) = 170.8 (1C, C-6-O(*C*=O)CH$_3$), 170.1 (1C, C-3-O(*C*=O)CH$_3$), 170.0 (1C, C-4-O(*C*=O)CH$_3$), 169.8 (1C, C-2-O(*C*=O)CH$_3$), 96.4 (1C, C-1), 78.1 (1C, C-2‘), 75.7 (1C, C-3‘), 69.5 (1C, C-2), 69.1 (1C, C-5), 69.1 (1C, C-3), 66.2 (1C, C-4), 62.5 (1C, C-6), 55.1 (1C, C-1‘), 21.0 (1C, C-3-O(C=O)*C*H$_3$), 20.9 (1C, C-6-O(C=O)*C*H$_3$), 20.8 (1C, C-2-O(C=O)*C*H$_3$), 20.8 (1C, C-4-O(C=O)*C*H$_3$). **HR-MS** (ESI$^+$)**:** $m/z_{cal.}$ = 409.1105 [M+Na]$^+$, $m/z_{exp.}$ = 409.1104 [M+Na]$^+$. ***R$_f$*** (NP) = 0.59 (^{c}Hex/EtOAc 1:1). **Melting range** (°C) = 103.0 – 104.5 (EtOAc). **Optical rotation** (LM): $[\alpha]_D^{19}$ = +58.3 ($CHCl_3$). **IR** (ATR): (cm^{-1}) = 3272, 2958, 1747, 1437, 1371, 1224, 1137, 1078, 1050, 982, 919, 797, 691, 601.

The analytical data follows literature data.[6-9]

4.1.3 Synthesis of Propargyl 2,4-Di-*O*-benzoyl-α-D-mannopyranoside

The title compound was synthesized utilizing a telescoped three-strep protocol according to Scheme S 5 following modified synthesis protocols by *Ramos-Soriano et al.*[18], *Teumelsan et al.*[19], *van den Bos et al.*[21] and *Zhang et al.*[22]

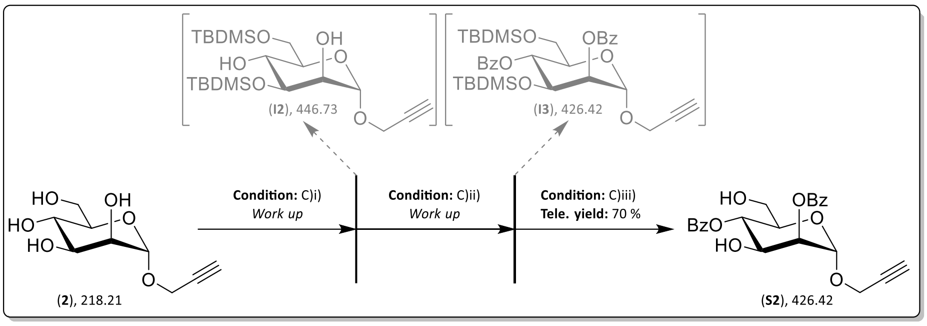

Scheme S 5: Synthesis of Propargyl 2,4-Di-*O*-benzoyl-α-D-mannopyranoside (**S2**). Reaction conditions: C)i) TBDMSCl, Imi., DMF, Ar-atm., MS 3Å, 0 °C, 4 h; C)ii) BzCl, Pyr., DMAP, DCM, Ar-atm., MS 3Å, 0 °C to rt, 42 h; C)iii) $(HF)_X$·Pyr., AcOH, THF, 0 °C to rt, 24 h; telescoped yield: 70 %.

The first two reaction steps were carried out in dried Schlenk vessels, the third in a Teflon vessel, which had previously been fitted with magnetic stirring bars. Propargyl α-D-mannopyranoside (**2**, 0.10 g, 0.46 mmol, 1.00 eq.) was dissolved in absolute DMF (1.00 mL) under an atmosphere of argon and the mixture was cooled to a temperature of 0 °C in an ice bath. Successively TBDMSCl (0.21 g, 1.37 mmol, 3.00 eq.) and imidazole (0.19 g, 2.75 mmol, 6.00 eq.) were added. The yellowish reaction mixture was stirred at this temperature for three hours. Complete conversion of the limiting substrate and formation of the desired product were determined by reaction control via TLC and HPLC-ELS-MS. The mixture was diluted with DCM (1 x 50.0 mL) and the organic phase was washed with saturated $NaHCO_3$ solution (1 x 30.0 mL), H_2O (2 x 30.0 mL) and saturated NaCl solution (1 x 30.0 mL). The collected aqueous phases were extracted with DCM (1 x 30.0 mL). The collected organic phases were dried over anhydrous Na_2SO_4 and filtered. All volatiles were removed in vacuo. Toluene (2 x 5.00 mL) was added to the residue and again all volatiles were removed in vacuo. The residue was dried in fine vacuum. The first intermediate (0.24 g) was obtained in the form of colorless oil and was dissolved in absolute DCM (2.00 mL) under an atmosphere of argon and molecular sieves (MS 3 Å, 0.25 g) were added. The mixture was stirred for ten minutes at room temperature and then cooled to a temperature of 0 °C in an ice bath. Absolute pyridine (0.50 mL), DMAP (11.2 mg, 92.0 µmol, 0.20 eq.) and benzoyl chloride (0.36 g, 0.29 mL, 2.53 mmol, 5.50 eq.) were added successively. Cooling was removed and the reaction mixture was stirred at room temperature for 24 hours to obtain a cloudy suspension. Complete conversion of the limiting substrate and formation of the desired product were determined by reaction control via TLC and HPLC-ELS-MS. The cloudy reaction mixture was filtered through a glass frit via Celite and eluted with EtOAc (1 x 100 mL). The organic phase was washed with H_2O (2 x 50.0 mL), 1 M HCl solution (1 x 50.0 mL), saturated $NaHCO_3$ solution (1 x 50.0 mL) and saturated NaCl solution (1 x 30.0 mL). The organic phase was dried over anhydrous Na_2SO_4 and filtered. All volatiles were removed in vacuo. Toluene (2 x 5.00 mL) was added to the residue and again all volatiles were removed in vacuo. The residue was dried in fine vacuum. The second intermediate (0.49 g) was obtained in the form of yellowish oil and dissolved in THF (5.00 mL). The resulting colorless solution was cooled to a temperature of 0 °C in an ice bath. AcOH (0.20 mL) and $(HF)_X$·Pyr. (70 %, 1.31 g, 1.19 mL, 46.0 mmol, 100 eq.) were added with a syringe and the resulting yellowish solution was stirred for 24 hours while slowly warming up to room temperature. Complete conversion of the limiting substrate and formation of the desired product were determined by reaction control via TLC and HPLC-ELS-MS. The solution

was diluted with DCM (1 x 100 mL) and the organic phase was poured onto an ice-cooled, saturated $NaHCO_3$ solution (100 mL). The organic phase was washed with saturated $NaHCO_3$ solution (2 x 50.0 mL), 1 M HCl solution (1 x 50.0 mL), saturated $NaHCO_3$ solution (1 x 50.0 mL) and saturated NaCl solution (1 x 50.0 mL). The collected organic phases were dried over anhydrous Na_2SO_4 and filtered. All volatiles were removed in vacuo. Toluene (2 x 5.00 mL) was added to the residue and again all volatiles were removed in vacuo. The residue was dried in fine vacuum. The crude product (0.17 g) was obtained in the form of a yellowish oil and purified using RP-FCC (MeCN/H_2O, gradient 5 % to 55 % to 100 % MeCN, Isolera One Flash Purification System, SNAP C_{18} 30 g cartridge). The pure product (**S2**) was obtained in the form of a colorless lyophilizate.

Yield: 0.14 g (0.32 mmol, 70 %), colorless lyophilizate.

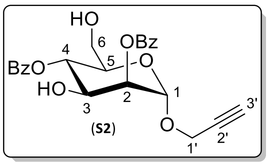

^{1}H-NMR (400 MHz, $CDCl_3$, 25 °C)**:** δ (ppm) = 8.15 – 8.04 (m, 4H, H_{ortho}), 7.65 – 7.56 (m, 2H, H_{para}), 7.52 – 7.42 (m, 4H, H_{meta}), 5.52 (t, ^3J = 10.0 Hz, 1H, H-4), 5.45 (dd, ^3J = 3.5, 1.7 Hz, 1H, H-2), 5.25 (d, ^3J = 1.7 Hz, 1H, H-1), 4.44 (dd, ^3J = 9.9, 3.5 Hz, 1H, H-3), 4.33 (d, ^4J = 2.4 Hz, 2H, H-1‘), 4.00 (ddd, ^3J = 10.1, 4.2, 2.3 Hz, 1H, H-5), 3.82 (dd, ^2J = 12.7 Hz, ^3J = 2.3 Hz, 1H, H-6a), 3.74 (d, ^2J = 12.7 Hz, ^3J = 4.2 Hz, 1H), 2.51 (t, ^4J = 2.4 Hz, 1H). **^{13}C-NMR** (101 MHz, $CDCl_3$, 25 °C)**:** δ (ppm) = 167.3 (1C, C-4-O(*C*=O)Ph), 166.0 (1C, C-2-O(*C*=O)Ph), 133.8 (1C, C-2-O(C=O)Ph, C_{para}), 133.6 (1C, C-4-O(C=O)Ph, C_{para}), 129.9 (2C, C-2-O(C=O)Ph, C_{ortho}), 129.1 (1C, C-2-O(C=O)Ph, C_{ipso}), 129.0 (1C, C-4-O(C=O)Ph, C_{ipso}), 128.6 (2C, C-2-O(C=O)Ph, C_{meta}), 128.6 (2C, C-4-O(C=O)Ph, C_{meta}), 96.6 (1C, C-1), 78.3 (1C, C-2‘), 75.5 (1C, C-3‘), 72.6 (1C, C-2), 71.1 (1C, C-5), 70.2 (1C, C-4), 68.6 (1C, C-3), 61.3 (1C, C-6), 55.3 (1C, C-1‘). **HR-MS** (ESI$^+$)**:** $m/z_{cal.}$ = 449.1207 [M+Na]$^+$, $m/z_{exp.}$ = 449.1206 [M+Na]$^+$. **Optical rotation** (LM): $[\boldsymbol{\alpha}]_D^{20}$ = -16.2 ($CHCl_3$). $\boldsymbol{R_f}$ (NP) = 0.30 (^{c}Hex/EtOAc 2:1). **IR** (ATR): (cm^{-1}) = 3491, 3297, 2927, 1720, 1602, 1452, 1318, 1265, 1178, 1114, 1069, 1027, 1012, 971, 912, 794, 711, 686.

The analytical data follows literature data.[6]

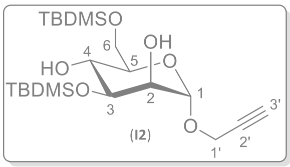

^{1}H-NMR (400 MHz, $CDCl_3$, 25 °C)**:** δ (ppm) = 5.05 (d, ^3J = 1.5 Hz, 1H, H-1), 4.24 (d, ^4J = 2.4 Hz, 2H, H-1‘), 3.91 – 3.81 (m, 3H, H-3, H-6a, H-6b), 3.79 (ddd, ^3J = 3.7, 1.5, 1.2 Hz, 1H, H-2), 3.73 (td, ^3J = 9.1, 8.6, 2.1 Hz, 1H, H-4), 3.60 (dt, ^3J = 9.1, 5.5 Hz, 1H, H-5), 2.69 (d, ^3J = 2.1 Hz, 1H, C-4-O*H*), 2.60 (d, ^3J = 1.2 Hz, 1H, C-2-O*H*), 2.43 (t, ^4J = 2.4 Hz, 1H, H-3‘), 0.92 (s, 9H, C-3-O-Si-C(CH_3)$_3$), 0.90 (s, 9H, C-6-O-Si-C(CH_3)$_3$), 0.16 (s, 3H, C-3-O-Si-CH_3), 0.14 (s, 3H, C-3-O-Si-CH_3), 0.09 (s, 6H, C-6-O-Si-CH_3). **^{13}C-NMR** (101 MHz, $CDCl_3$, 25 °C)**:** δ (ppm) = 97.6 (1C, C-1), 79.0 (1C, C-2‘), 74.9 (1C, C-3‘), 73.1 (1C, C-3), 71.2 (1C, C-2), 71.1 (1C, C-5), 70.6 (1C, C-4), 65.0 (1C, C-6), 54.2 (1C, C-1‘), 26.0 (3C, C-6-O-Si-C(*C*H_3)$_3$), 26.0 (3C, C-3-O-Si-C(*C*H_3)$_3$), 18.4 (1C, C-6-O-Si-*C*(CH_3)$_3$), 18.2 (1C, C-3-O-Si-*C*(CH_3)$_3$), -4.3 (1C, C-3-O-Si-*C*H_3), -4.7 (1C, C-3-O-Si-*C*H_3), -5.3 (1C, C-6-O-Si-*C*H_3), -5.3 (1C, C-6-O-Si-*C*H_3). **HR-MS** (ESI$^+$)**:** $m/z_{cal.}$ = 469.2412 [M+Na]$^+$, $m/z_{exp.}$ = 469.2414 [M+Na]$^+$. $\boldsymbol{R_f}$ (NP) = 0.41 (^{c}Hex/EtOAc 4:1). **Melting range:** Θ (°C) = 78.5 – 80.0 (EtOAc). **Optical rotation** (LM)**:** $[\boldsymbol{\alpha}]_D^{20}$ = +58.7 ($CHCl_3$). **IR** (ATR): (cm^{-1}) = 3508, 3312, 2953, 2929, 2858, 1472, 1254, 1215, 1138, 1108, 1048, 1012, 970, 916, 868, 836, 779, 671.

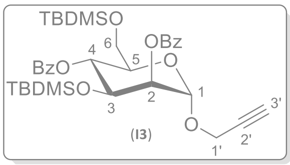

^{1}H-NMR (400 MHz, $CDCl_3$, 25 °C)**:** δ (ppm) = 8.14 – 8.10 (m, 2H, C-2-O(C=O)Ph, H_{ortho}), 8.07 – 8.02 (m, 2H, C-4-O(C=O)Ph, H_{ortho}), 7.61 – 7.54 (m, 2H, H_{para}), 7.50 – 7.41 (m, 4H, H_{meta}), 5.60 (t_{app}, ^3J = 9.8 Hz, 1H, H-4), 5.39 (dd, ^3J = 3.6, 1.8 Hz, 1H, H-2), 5.16 (d, ^3J = 1.8 Hz, 1H, H-1), 4.35 (dd, ^3J = 9.8, 3.6 Hz, 1H, H-3), 4.34 (d, ^4J = 2.4 Hz, C-1‘) 3.94 (ddd, ^3J = 9.8, 5.7, 2.8 Hz, 1H, H-5), 3.79 (dd, ^2J = 11.3 Hz, ^3J = 5.7 Hz, 1H, H-6a), 3.75 (dd, ^2J = 11.3 Hz,

^{3}J = 2.8 Hz, 1H, H-6b), 2.49 (t, ^{4}J = 2.4 Hz, 1H, H-3‘), 0.86 (s, 9H, C-6-O-Si-C(C*H*$_3$)$_3$), 0.61 (s, 9H, C-3-O-Si-C(C*H*$_3$)$_3$), 0.04 (s, 3H, C-3-O-Si-C*H*$_3$), 0.00 (s, 3H, C-6-O-Si-C*H*$_3$), -0.01 (s, 3H, -C-6-O-Si-C*H*$_3$), -0.15 (s, 3H, C-3-O-Si-C*H*$_3$). **^{13}C-NMR** (101 MHz, CDCl$_3$, 25 °C)**:** δ (ppm) = 166.0 (1C, C-2-O(*C*=O)Ph), 165.3 (1C, C-4-O(*C*=O)Ph), 133.3 (1C, C-2-O(C=O)Ph, C$_{para}$), 133.2 (1C, C-4-O(C=O)Ph, C$_{para}$), 130.1 (2C, C-2-O(C=O)Ph, C$_{ortho}$), 130.0 (1C, C-2-O(C=O)Ph, C$_{ipso}$), 129.9 (1C, C-4-O(C=O)Ph, C$_{ipso}$), 129.9 (2C, C-4-O(C=O)Ph, C$_{ortho}$), 128.6 (2C, C-2-O(C=O)Ph, C$_{meta}$), 128.5 (2C, C-4-O(C=O)Ph, C$_{meta}$), 96.2 (1C, C-1), 78.6 (1C, C-2‘), 75.4 (1C, C-3‘), 72.6 (1C, C-2), 72.3 (1C, C-5), 70.1 (1C, C-4), 69.1 (1C, C-3), 62.9 (1C, C-6), 54.4 (1C, C-1‘), 26.0 (3C, C-6-O-Si-C(*C*H$_3$)$_3$), 25.4 (3C, C-3-O-Si-C(*C*H$_3$)$_3$), 18.4 (1C, C-6-O-Si-*C*(CH$_3$)$_3$), 17.7 (1C, C-3-O-Si-*C*(CH$_3$)$_3$), -4.60 (1C, C-3-O-Si-*C*H$_3$), -4.98 (1C, C-3-O-Si-*C*H$_3$), -5.28 (1C, C-6-O-Si-*C*H$_3$), -5.33 (1C, C-6-O-Si-*C*H$_3$). **HR-MS** (ESI$^+$)**:** $m/z_{\text{cal.}}$ = 677.2936 [M+Na]$^+$, $m/z_{\text{exp.}}$ = 677.2929 [M+Na]$^+$. ***R$_f$*** (NP) = 0.53 (^{c}Hex/EtOAc 10:1). **Optical rotation** (LM)**:** $[\alpha]_D^{20}$ = -10.4 (CHCl$_3$). **IR** (ATR)**:** (cm^{-1}) = 2929, 2857, 1728, 1602, 1452, 1389, 1361, 1323, 1261, 1108, 1062, 1028, 990, 878, 837, 778, 710, 677.

4.1.4 Synthesis of Propargyl 2,4-Di-*O*-benzoyl-3,6-di-*O*-(2,3,4,6-tetra-*O*-acetyl-α-D-mannopyranosyl)-α-D-mannopyranoside

The title compound was synthesized according to following modified synthesis protocols by *Kanaya et al.*[23], *Ramos-Soriano et al.*[18] and *Teumelsan et al.*[19]

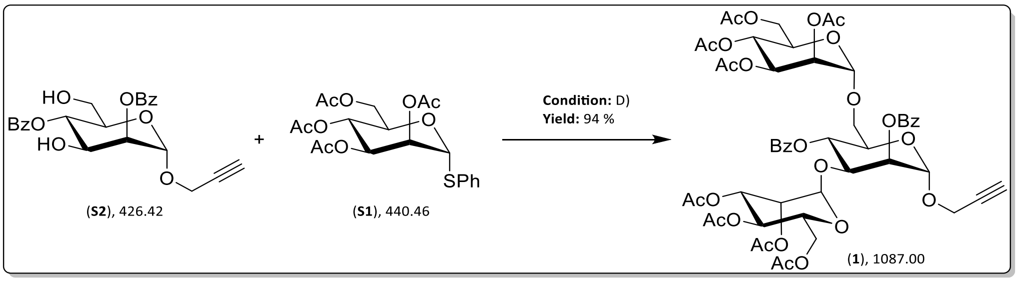

Scheme S 6: Synthesis of Propargyl 2,4-Di-*O*-benzoyl-3,6-di-*O*-(2,3,4,6-tetra-*O*-acetyl-α-D-mannopyranosyl)-α-D-mannopyranoside (**1**). Reaction conditions: D) NIS, AgOTf, DCM, Ar-atm., -40 °C to -20 °C, 4 h; yield: 94 %.

The reaction was carried out in a dried Schlenk vessel, which was previously fitted with a magnetic stir bar. Propargyl 2,4-Di-O-benzoyl-α-D-mannopyranoside (**1**, 0.10 g, 0.23 mmol, 1.00 eq.) and phenyl 2,3,4,6-tetra-O-acetyl-1-thio-α-D-mannopyranoside (**S1**, 0.31 g, 0.70 mmol, 3.00 eq.) were added toluene (3 x 2.50 mL) and all volatiles were removed in vacuo. The residue was dried in fine vacuum for half an hour and dissolved in absolute DCM (5.00 mL) under an atmosphere of argon. Molecular sieves (MS 3 Å, 0.25 g) were added and the solution stirred for half an hour at room temperature. The solution was cooled down to a temperature of -40 °C in an acetone bath using a cryostat. NIS (0.24 g, 1.06 mmol, 4.50 eq.) was added to the mixture in small portions. AgOTf (28.0 mg, 0.11 mmol, 0.45 eq.) was dissolved in absolute toluene (0.50 mL) under an atmosphere of argon in another dried Schlenk vessel and the solution was added to the cooled reaction mixture with a syringe. The resulting colorless suspension was stirred for two hours while slowly warming up to a temperature of -20 °C, whereby a red suspension was obtained. The reaction mixture was stirred for a further two hours at this temperature. Complete conversion of the limiting substrate and formation of the desired product were determined by reaction control via TLC and HPLC-ELS-MS. The reaction was terminated by addition of NEt$_3$ (0.07 g, 0.10 mL, 0.70 mmol, 3.00 eq.). The resulting orange suspension was filtered through a glass frit over *Celite* and eluted with DCM (1 x 50.0 mL). The organic phase was washed with saturated $Na_2S_2O_3$ solution (2 x 50.0 mL), saturated $NaHCO_3$ solution (2 x 50.0 mL) and saturated NaCl solution (1 x 50.0 mL), dried over anhydrous Na_2SO_4 and filtered. All volatiles were removed in vacuo and the residue was dried in fine vacuum. The crude product (0.51 g) was obtained in the form of a dark orange

oil and purified using FCC (^{c}Hex/EtOAc, gradient 0 % to 70 % to 100 % EtOAc, Isolera Four Flash Purification System, SNAP KP Sil 50 g cartridge) and RP-FCC (MeCN/H_2O, gradient 10 % to 70 % to 100 % MeCN, Isolera One Flash Purification System, SNAP C_{18} 30 g cartridge). The pure product (**1**) was obtained in the form of a colorless lyophilizate.

Yield: 0.24 g (0.22 mmol, 94 %), colorless lyophilizate.

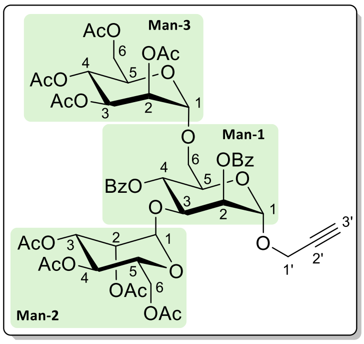

^{1}H-NMR (600 MHz, $CDCl_3$, 25 °C)**:** δ (ppm) = 8.17 – 8.13 (m, 2H, C-2$_{\text{Man-1}}$-O(C=O)Ph, H$_{ortho}$), 8.05 – 8.00 (m, 2H, C-4$_{\text{Man-1}}$-O(C=O)Ph, H$_{ortho}$), 7.65 – 7.61 (m, 1H, C-2$_{\text{Man-1}}$-O(C=O)Ph, H$_{para}$), 7.60 – 7.57 (m, 1H, C-4$_{\text{Man-1}}$-O(C=O)Ph, H$_{para}$), 7.56 – 7.52 (m, 2H, C-2$_{\text{Man-1}}$-O(C=O)Ph, H$_{meta}$), 7.47 – 7.43 (m, 2H, C-4$_{\text{Man-1}}$-O(C=O)Ph, H$_{meta}$), 5.63 (t_{app}, ^{3}J = 10.0 Hz, 1H, H-4$_{\text{Man-1}}$), 5.54 (dd, ^{3}J = 3.5, 1.8 Hz, 1H, H-2$_{\text{Man-1}}$), 5.33 (dd, ^{3}J = 10.1, 3.4 Hz, 1H, H-3$_{\text{Man-3}}$), 5.24 (d, ^{3}J = 1.8 Hz, 1H, H-1$_{\text{Man-1}}$), 5.27 – 5.21 (m, 2H, H-2$_{\text{Man-3}}$, H-4$_{\text{Man-3}}$), 5.11 – 5.06 (dd, 2H, H-3$_{\text{Man-2}}$, H-4$_{\text{Man-2}}$), 4.97 (d, ^{3}J = 1.9 Hz, 1H, H-1$_{\text{Man-2}}$), 4.88 – 4.85 (m, 1H, H-2$_{\text{Man-2}}$), 4.80 (d, ^{3}J = 1.8 Hz, 1H, H-1$_{\text{Man-3}}$), 4.49 (dd, ^{3}J = 9.8, 3.4 Hz, 1H, H-3$_{\text{Man-1}}$), 4.36 (d, ^{4}J = 2.4 Hz, 2H, H-1‘), 4.22 – 4.13 (m, 3H, H-5$_{\text{Man-1}}$, H-6a$_{\text{Man-2}}$, H-6a$_{\text{Man-3}}$), 4.11 (ddd, ^{3}J = 9.4, 5.7, 1.8 Hz, 1H, H-5$_{\text{Man-2}}$), 4.06 (ddd, ^{3}J = 10.2, 5.5, 2.1 Hz, 1H, H-5$_{\text{Man-3}}$), 4.01 (ddd, ^{2}J = 12.2, ^{3}J = 5.7, 2.0 Hz, 2H, H-6b$_{\text{Man-2}}$, H-6b$_{\text{Man-3}}$), 3.90 (dd, ^{2}J = 10.8 Hz, ^{3}J = 6.8 Hz, 1H, H-6a$_{\text{Man-1}}$), 3.61 (dd, ^{2}J = 10.8 Hz, ^{3}J = 2.2 Hz, 1H, H-6b$_{\text{Man-1}}$), 2.55 (t, ^{4}J = 2.4 Hz, 1H, H-3‘), 2.14 (s, 3H, C-6$_{\text{Man-3}}$-O(C=O)C*H*3), 2.11 (s, 3H, C-4$_{\text{Man-3}}$-O(C=O)C*H*3), 2.04 (s, 3H, C-3$_{\text{Man-3}}$-O(C=O)C*H*3), 1.98 (s, 3H, C-3$_{\text{Man-2}}$-O(C=O)C*H*3), 1.94 (s, 3H, C-6$_{\text{Man-2}}$-O(C=O)C*H*3), 1.94 (s, 3H, C-4$_{\text{Man-2}}$-O(C=O)C*H*3), 1.84 (s, 3H, C-2$_{\text{Man-3}}$-O(C=O)C*H*3), 1.82 (s, 3H, C-2$_{\text{Man-2}}$-O(C=O)C*H*3). **^{13}C-NMR** (151 MHz, $CDCl_3$, 25 °C)**:** δ (ppm) = 170.8 (1C, C-6$_{\text{Man-2}}$-O(*C*=O)CH3), 170.6 (1C, C-6$_{\text{Man-3}}$-O(*C*=O)CH3), 170.0 (1C, C-4$_{\text{Man-3}}$-O(*C*=O)CH3), 169.8 (1C, C-3$_{\text{Man-3}}$-O(*C*=O)CH3), 169.8 (1C, C-3$_{\text{Man-2}}$-O(*C*=O)CH3), 169.7 (1C, C-4$_{\text{Man-2}}$-O(*C*=O)CH3), 169.1 (1C, C-2$_{\text{Man-3}}$-O(*C*=O)CH3), 169.1 (1C, C-2$_{\text{Man-2}}$-O(*C*=O)CH3), 165.9 (1C, C-2$_{\text{Man-1}}$-O(*C*=O)Ph), 165.2 (1C, C-4$_{\text{Man-1}}$-O(*C*=O)Ph), 133.7 (1C, C-2$_{\text{Man-1}}$-O(C=O)Ph, C$_{para}$), 133.6 (1C, C-4$_{\text{Man-1}}$-O(C=O)Ph, C$_{para}$), 130.0 (2C, C-2$_{\text{Man-1}}$-O(C=O)Ph, C$_{ortho}$), 129.9 (2C, C-4$_{\text{Man-1}}$-O(C=O)Ph, C$_{ortho}$), 129.0 (1C, C-2$_{\text{Man-1}}$-O(C=O)Ph, C$_{ipso}$), 128.8 (2C, C-2$_{\text{Man-1}}$-O(C=O)Ph, C$_{meta}$), 128.6 (1C, C-4$_{\text{Man-1}}$-O(C=O)Ph, C$_{ipso}$), 128.5 (2C, C-4$_{\text{Man-1}}$-O(C=O)Ph, C$_{meta}$), 99.4 (1C, C-1$_{\text{Man-2}}$), 97.2 (1C, C-1$_{\text{Man-3}}$), 96.1 (1C, C-1$_{\text{Man-1}}$), 78.0 (1C, C-2‘), 75.7 (1C, C-3‘), 74.9 (1C, C-3$_{\text{Man-1}}$), 71.5 (1C, C-2$_{\text{Man-1}}$), 70.0 (1C, C-5$_{\text{Man-1}}$), 69.3 (2C, C-5$_{\text{Man-2}}$, C-2$_{\text{Man-3}}$), 69.2 (1C, C-2$_{\text{Man-2}}$), 69.0 (1C, C-3$_{\text{Man-3}}$), 68.7 (1C, C-4$_{\text{Man-1}}$), 68.6 (1C, C-5$_{\text{Man-3}}$), 68.2 (1C, C-3$_{\text{Man-2}}$), 66.7 (1C, C-6$_{\text{Man-1}}$), 66.0 (1C, C-4$_{\text{Man-2}}$), 65.8 (1C, C-4$_{\text{Man-3}}$), 62.4 (1C, C-6$_{\text{Man-2}}$), 62.3 (1C, C-6$_{\text{Man-3}}$), 55.0 (1C, C-1‘), 20.9 (1C, C-4$_{\text{Man-3}}$-O(C=O)*C*H3), 20.8 (1C, C-6$_{\text{Man-3}}$-O(C=O)*C*H3), 20.8 (1C, C-3$_{\text{Man-3}}$-O(C=O)*C*H3), 20.7 (1C, C-6$_{\text{Man-2}}$-O(C=O)*C*H3), 20.7 (1C, C-3$_{\text{Man-2}}$-O(C=O)*C*H3), 20.6 (1C, C-4$_{\text{Man-2}}$-O(C=O)*C*H3), 20.5 (1C, C-2$_{\text{Man-2}}$-O(C=O)*C*H3), 20.5 (1C, C-2$_{\text{Man-3}}$-O(C=O)*C*H3). **HR-MS** (ESI^{+})**:** $m/z_{\text{cal.}}$ = 1109.3117 $[M+Na]^{+}$, $m/z_{\text{exp.}}$ = 1109.3117 $[M+Na]^{+}$. **Optical rotation** (LM): $[\boldsymbol{\alpha}]_{D}^{20}$ = + 12.0 ($CHCl_3$). $\boldsymbol{R_f}$ (NP) = 0.19 (^{c}Hex/EtOAc 1:1). **IR** (ATR)**:** (cm^{-1}) = 3278, 2948, 1750, 1602, 1452, 1370, 1319, 1225, 1139, 1046, 981, 916, 715, 601.

4.1.5 Synthesis of 1,11-Di[(methanesulfonyl)oxy]-3,6,9-trioxaundecane

The title compound was synthesized according to Scheme S 7 following modified synthesis protocols by *Bakleh et al.*[24] and *Kramer et al.*[6]

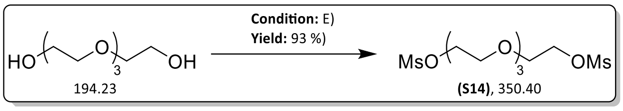

Scheme S 7: Synthesis of 1,11-Di[(methanesulfonyl)oxy]-3,6,8-trioxaundecane (**S14**). Reaction control: E) MsCl, NEt_3, DCM, Ar-atm., 0 °C to rt, 3 h; yield: 93 %.

The reaction was carried out in a dried Schlenk vessel, which was previously fitted with a magnetic stirring rod. Tetraethylene glycol (5.00 g, 25.7 mmol, 1.00 eq.) was dried in fine vacuum for half an hour with stirring and then dissolved in absolute DCM (125 mL) under an atmosphere of argon. The mixture was cooled to a temperature of 0 °C in an ice bath. Successively, MsCl (6.49 g, 4.38 mL, 56.6 mmol, 2.20 eq.) and NEt_3 (7.81 g, 10.8 mL 77.2 mmol, 3.00 eq.) were added slowly with stirring. The reaction mixture was stirred for three hours while slowly warming up to room temperature. Complete conversion of the limiting substrate and formation of the desired product were determined by reaction control via TLC and HPLC-ELS-MS. The reaction was terminated by the addition of ice-cooled H_2O (1 x 125 mL). The organic phase was separated and washed with ice-cooled 2 M HCl (1 x 125 mL), saturated $NaHCO_3$ solution (1 x 125 mL) and saturated NaCl solution (1 x 125 mL). The organic phase was dried over anhydrous Na_2SO_4, filtered and all volatile components were removed in vacuo. The residue was dried in a fine vacuum. The pure product (**S14**) was obtained in the form of yellowish oil.

Yield: 8.37 g (23.9 mmol, 93 %), (Lit.[6, 24]: 98 %), yellowish oil.

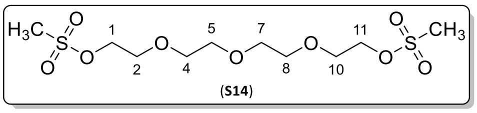

^{1}H-NMR (400 MHz, $CDCl_3$, 25 °C)**:** δ (ppm) = 4.38 – 4.34 (m, 4H, H-1, H-11), 3.77 – 3.73 (m, 4H, H-2, H-10), 3.67 – 3.60 (m, 8H, H-4, H-5, H-7, H-8), 3.06 (s, 6H, -C*H*3). **^{13}C-NMR** (101 MHz, $CDCl_3$, 25 °C): δ (ppm) = 70.7 (2C, C-5, C-7), 70.6 (2C, C-4, C-8), 69.4 (2C, C-2, C-10), 69.1 (2C, C-1, C-11), 37.7 (2C, -*C*H3). **HR-MS** (ESI$^+$)**:** $m/z_{cal.}$ = 373.0597 [M+Na]$^+$, $m/z_{exp.}$ = 373.0600 [M+Na]$^+$. ***R**f* (NP) = 0.08 (^{c}Hex/EtOAc 1:2). **IR** (ATR)**:** (cm^{-1}) = 3026, 2877, 1454, 1346, 1249, 1172, 1134, 1108, 1015, 974, 919, 803, 732, 528, 483, 456, 420.

The analytical data follows literature data.[6, 24]

4.1.6 Synthesis of 1,11-Diazido-3,6,9-trioxaundecane

The title compound was synthesized according to Scheme S 8 following modified synthesis protocols by *Bakleh et al.*[24] and *Kramer et al.*[6]

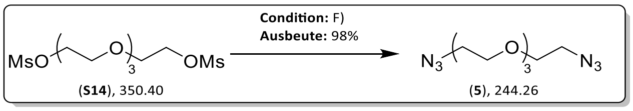

Scheme S 8: Synthesis of 1,11-Diazido-3,6,9-trioxaundecane (**5**). Reaction conditions: F) NaN_3, TBAI, DMF, Ar-atm., 80 °C, 4 h; yield: 98 %.

The reaction was carried out in a dried Schlenk vessel, which was previously fitted with a magnetic stir bar. 1,11-Di[(methanesulfonyl)oxy]-3,6,9-trioxaundecane (**S14**, 2.00 g, 5.71 mmol, 1.00 eq.) was dissolved in absolute DMF (10.0 mL) under atmosphere of argon. NaN_3 (0.93 g, 14.3 mmol, 2.50 eq.) and TBAI (0.11 g, 0.29 mmol, 0.05 eq.) were added. The orange reaction mixture was stirred for four hours at a temperature of 80 °C, resulting in a colorless precipitation. Complete conversion of the limiting substrate and formation of the desired product were determined by reaction control via TLC and HPLC-ELS-MS. The mixture was filtered over *Celite* and eluted with Et_2O (1 x 50.0 mL). All

volatiles were removed first in vacuo and then in fine vacuum. Toluene (3 x 50.0 mL) was added to the residue and again all volatiles were removed in fine vacuum. The residue was dissolved in Et_2O (1 x 50.0 mL) and filtered through *Celite* to remove insoluble salts. All volatiles were removed in vacuo and the residue was dried in fine vacuum. The pure product (**5**) was obtained in the form of yellowish oil.

Yield: 1.37 g (5.61 mmol, 98 %), (Lit.[6, 24]: 98 %), yellowish oil.

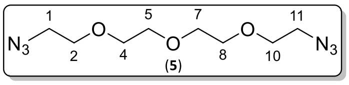

^{1}H-NMR (300 MHz, $CDCl_3$, 25 °C)**:** δ (ppm) = 3.72 – 3.63 (m, 12H, H-2, H-4, H-5, H-7, H-8, H-10), 3.38 (t, ^3J = 5.1 Hz, 4H, H-1, H-11). **^{13}C-NMR** (75.5 MHz, $CDCl_3$, 25 °C)**:** δ (ppm) = 70.8 (4C, C-4, C-5, C-7, C-8), 70.2 (2C, C-2, C-10), 50.8 (2C, C-1, C-11). **HR-MS** (ESI$^+$)**:** $m/z_{cal.}$ = 267.1176 [M+Na]$^+$, $m/z_{exp.}$ = 267.1175 [M+Na]$^+$. $\boldsymbol{R_f}$ (NP) = 0.37 (^{c}Hex/EtOAc 2:1). **IR** (ATR): (cm^{-1}) = 2868, 2097, 1443, 1346, 1285, 1122, 992, 936, 853, 648, 556, 436, 419.

The analytical data follows literature data.[6, 24-26]

4.1.7 Synthesis of 11-Amino-1-azido-3,6,9-trioxaundecane

The title compound was synthesized according to Scheme S 9 following modified synthesis protocols *Bakleh et al.*[24] and *Krumb et al.*[26]

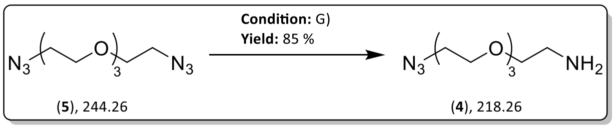

Scheme S 9: Synthesis of 11-Amino-1-azido-3,6,9-trioxaundecane (**4**). Reaction conditions: A) PPh_3, Et_2O/THF/1 M HCl (8:1:4), rt, 48 h; yield: 85 %.

The reaction was carried out in a reaction vessel, which was previously equipped with a magnetic stirring bar. 1,11-Diazido-3,6,9-trioxaundecane (**5**, 3.00 g, 12.3 mmol, 1.00 eq.) was dissolved in a mixture of Et_2O (20.0 mL) and THF (5.00 mL). An aqueous 1 M HCl solution (20.0 mL) was added to the yellowish solution. PPh_3 (3.22 g, 12.3 mmol, 1.00 eq.) was dissolved in Et_2O (20.0 mL) and added to the mixture within one hour using a dropping funnel, accompanied by decolorization of the reaction mixture. The reaction solution was stirred for 48 hours at room temperature. Complete conversion of the limiting substrate and formation of the iminophosphorane intermediate and the byproduct triphenylphosphine oxide were observed by reaction control via TLC and HPLC-ELS-MS. A two-phase mixture with colorless organic and yellowish aqueous phase was obtained. The organic phase was discarded, and the aqueous phase extracted with Et_2O (5 x 50.0 mL) until the byproduct could no longer be detected by TLC. The aqueous phase was adjusted to a pH of 14 while stirring using ground NaOH pellets and then extracted with DCM (5 x 50.0 mL). The collected organic phases were dried over anhydrous Na_2SO_4 and filtered. All volatile components were removed in a vacuum and the residue was dried in fine vacuum. The crude product (3.56 g) was obtained in the form of yellowish oil and purified by FCC (DCM/MeOH + NEt_3, isocratic at 5 % MeOH and 1 $\%_{v/v}$ additive). The pure product (**4**) was obtained in the form of yellowish oil.

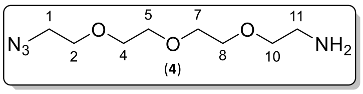

^{1}H-NMR (300 MHz, $CDCl_3$, 25 °C)**:** δ (ppm) = 3.67 – 3.55 (m, 10H, H-2, H-4, H-5, H-7, H-8), 3.48 (t, ^3J = 5.2 Hz, 2H, H-11), 3.36 (t, ^3J = 5.1 Hz, 2H, H-1), 2.83 (t, ^3J = 5.2 Hz, 2H, H-10), 1.89 (s, 2H, $-NH_2$). **^{13}C-NMR** (75.5 MHz, $CDCl_3$, 25 °C)**:** δ (ppm) = 73.1 (1C, C-11), 70.7, 70.6, 70.6, 70.2, 70.0 (5C, C-2, C-4, C-5, C-7, C-8), 50.6 (1C, C-1), 41.6 (1C, C-10). **HR-MS** (ESI$^+$)**:** $m/z_{cal.}$ = 219.1452 [M+H]$^+$, $m/z_{exp.}$ = 219.1457 [M+H]$^+$. $\boldsymbol{R_f}$ (NP) = 0.25 (DCM/MeOH + NEt_3 20:1 + 1 $\%_{v/v}$ additive). **IR** (ATR): (cm^{-1}) = 3366, 2866, 2098, 1593, 1454, 1348, 1286, 1105, 932, 851, 729, 644, 557.

The analytical data follows literature data.[24, 26]

4.1.8 Synthesis of *tert*-Butyl-12-Hydroxy-4,7,10-trioxadodecanoate

The title compound was synthesized according to Scheme S 10 following modified synthesis protocols by *Tavernaro et al.*[27] and *Krumb et al.*[26]

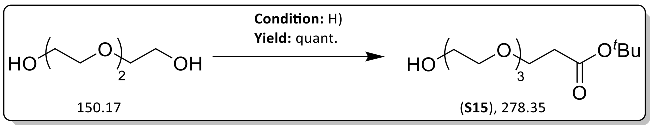

Scheme S 10: Synthesis of *tert*-Butyl-12-Hydroxy-4,7,10-trioxadodecanoate (**S15**). Reaction conditions H) TBA, Na, THF, Ar-atm., rt, 22 h; yield: quant.

The reaction was carried out in a dried Schlenk vessel, which was previously fitted with a magnetic stirring bar. Triethylene glycol (5.00 g, 4.46 mL, 33.3 mmol, 3.30 eq.) was dried in fine vacuum for half an hour with stirring and then dissolved in absolute THF (25.0 mL) under an atmosphere of argon. Elemental sodium (23.0 mg, 1.00 mmol, 0.03 eq.) was added with stirring and completely dissolved. TBA (1.29 g, 1.47 mL, 10.1 mmol, 1.00 eq.) was then added dropwise with syringe to the yellowish solution. The reaction was stirred for 22 hours in the absence of light at room temperature. Complete conversion of the limiting substrate and formation of the desired product were determined by reaction control via TLC and HPLC-ELS-MS. A 1 M HCl solution (1 x 1.30 mL) was added while stirring. All volatile components were removed in vacuo and the residue was dried in fine vacuum. The crude product (6.40 g) was obtained in the form of yellowish oil and dissolved in saturated NaCl solution (50.0 mL). The aqueous phase was extracted with EtOAc (4 x 50 mL). The collected organic phases were dried over anhydrous Na_2SO_4 and filtered. All volatiles were removed in vacuo and the residue was dried in a fine vacuum. The pure product (**S15**) was obtained in the form of colorless oil.

Yield: 2.81 g (10.1 mmol, quant.), (Lit.[26, 27]: 86 – 100 %), colorless oil.

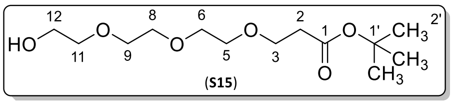

^{1}H-NMR (300 MHz, $CDCl_3$, 25 °C)**:** δ (ppm) = 3.77 – 3.73 (m, 2H, H-12), 3.73 – 3.71 (m, 2H, H-3), 3.71 – 3.62 (m, 8H, H-5, H-6, H-8, H-9), 3.62 – 3.59 (m, 2H, H-11), 2.51 (t, ^3J = 6.6 Hz, 2H, H-2), 1.44 (s, 9H, H-2‘). **^{13}C-NMR** (75.5 MHz, $CDCl_3$, 25 °C)**:** δ (ppm) = 170.9 (1C, C-1), 80.6 (1C, C-1‘), 72.5 (1C, C-11), 70.6, 70.5, 70.4, 70.4 (4C, C-5, C-6, C-8, C-9), 66.9 (1C, C-3), 61.8 (1C, C-12), 36.2 (1C, C-2), 28.1 (3C, C-2‘). **HR-MS** (ESI$^+$)**:** $m/z_{cal.}$ = 279.1802 $[M+H]^+$, $m/z_{exp.}$ = 279.1803 $[M+H]^+$; $m/z_{cal.}$ = 301.1622 $[M+Na]^+$, $m/z_{exp.}$ = 301.1629 $[M+Na]^+$. ***R_f*** (NP) = 0.13 (^{c}Hex/EtOAc 1:1). **IR** (ATR)**:** (cm^{-1}) = 3459, 2872, 1729, 1456, 1393, 1368, 1254, 1160, 1120, 1069, 939, 887, 847, 475.

The analytical data follows literature data.[26, 27]

4.1.9 Synthesis of *tert*-Butyl-12-[(*p*-*t*oluenesulfonyl)oxy]-4,7,10-trioxadodecanoate

The title compound was synthesized according to Scheme S 11 following modified synthesis protocols by *Tsakama et al.*[28] and *Krumb et al.*[26]

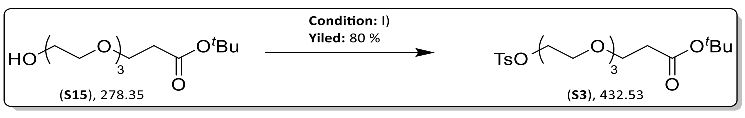

Scheme S 11: Synthesis of *tert*-Butyl-12-[(*p*-toluenesulfonyl)oxy]-4,7,10-trioxadodecanoate (**S3**). Reaction conditions: A) TsCl, NEt_3, DCM, Ar-atm., rt, 23 h; yield: 80 %.

The reaction was carried out in a dried Schlenk vessel, which was previously fitted with a magnetic stirring bar. *tert*-Butyl-12-Hydroxy-4,7,10-trioxadodecanoate (**S15**, 2.66 g, 9.56 mmol, 1.00 eq.) was

dissolved in absolute DCM (12.0 mL) under an atmosphere of argon and NEt_3 (2.32 g, 3.17 mL, 22.9 mmol, 2.40 eq.) was added. The solution was cooled to a temperature of 0 °C in an ice bath and TsCl (3.64 g, 19.1 mmol, 2.00 eq.) was added slowly. The reaction mixture was stirred for 23 hours at room temperature, resulting in a dark orange solution and formation of a colorless precipitate. Complete conversion of the limiting substrate and formation of the desired product were determined by reaction control via TLC and HPLC-ELS-MS. The precipitate was separated by filtration over *Celite* through a glass frit and eluted with DCM (1 x 100 mL). The resulting organic phase was washed with H_2O (3 x 100 mL) and saturated NaCl solution (1 x 100 mL). The collected aqueous phases were extracted with DCM (2 x 100 mL) and the collected organic phases were dried over anhydrous Na_2SO_4 and filtered. All volatile components were removed in vacuo and the residue was dried in fine vacuum. The crude product (5.43 g) was obtained in the form of an orange oil and purified using FCC (^{c}Hex/EtOAc, gradient 0 % to 35 % to 100 % EtOAc, Isolera Four Flash Purification System, SNAP KP-Sil 100 g cartridge). The pure product (**S3**) was obtained in the form of yellowish oil.

Yield: 3.32 g (7.65 mmol, 80 %), (Lit.[26, 28]: 69 %), yellowish oil.

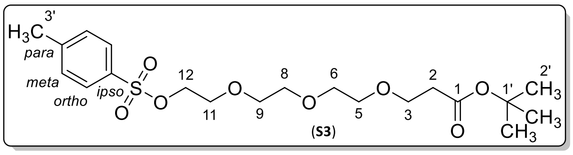

^{1}H-NMR (400 MHz, $CDCl_3$, 25 °C)**:** δ (ppm) = 7.82 – 7.77 (m, 2H, H_{ortho}), 7.36 – 7.32 (m, 2H, H_{meta}), 4.18 – 4.11 (m, 2H, H-12), 3.72 – 3.62 (m, 4H, H-3, H-11), 3.62 – 3.51 (m, 8H, H-5, H-6, H-8, H-9), 2.49 (t, ^3J = 6.6 Hz, 2H, H-2), 2.45 (s, 3H, H-5‘), 1.44 (s, 9H, H-2‘). **^{13}C-NMR** (101 MHz, $CDCl_3$, 25 °C)**:** δ (ppm) = 170.9 (1C, C-1), 144.8 (1C, C_{para}), 133.0 (1C, C_{ipso}), 129.8 (2C, C_{meta}), 128.0 (2C, C_{ortho}), 80.5 (1C, C-1‘), 70.8, 70.5, 70.3, 69.2 (5C, C-5, C-6, C-8, C-9, C-12), 68.7 (1C, C-11), 66.9 (1C, C-3), 36.2 (1C, C-2), 28.1 (1C, C-2‘), 21.6 (1C, C-3‘). **HR-MS** (ESI$^+$)**:** $m/z_{cal.}$ = 455.1710 [M+Na]$^+$, $m/z_{exp.}$ = 455.1710 [M+Na]$^+$. ***R_f*** (NP) = 0.22 (^{c}Hex/EtOAc 2:1). **IR** (ATR)**:** (cm^{-1}) = 2875, 1728, 1598, 1454, 1359, 1290, 1253, 1190, 1177, 1118, 1098, 1018, 923, 817, 776, 664, 583, 555.

The analytical data follows literature data.[26, 28]

4.1.10 Synthesis of *tert*-Butyl-12-azido-4,7,10-trioxadodecanoate

The title compound was synthesized according to Scheme S 12 following modified synthesis protocols by *Tavernaro et al.*[27] and *Krumb et al.*[26]

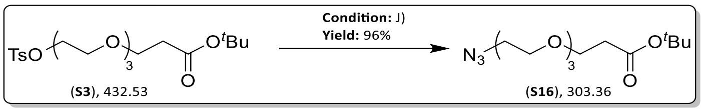

Scheme S 12: Synthesis of *tert*-Butyl-12-azido-4,7,10-trioxadodecanoate (**S16**). Reaction conditions: J) NaN_3, DMF, Ar-atm., rt, 21 h; yield: 96 %.

The reaction was carried out in a dried Schlenk vessel previously equipped with a magnetic stirring bar. *tert*-Butyl-12-[(p-toluenesulfonyl)oxy]-4,7,10-trioxadodecanoate (**S3**, 3.14 g, 7.26 mmol, 1.00 eq) was dissolved in absolute DMF (30.0 mL) under an atmosphere of argon. NaN_3 (0.61 g, 9.44 mmol, 1.30 eq.) was added. The reaction mixture was stirred for 21 hours at 60 °C, resulting in a yellowish solution. Complete conversion of the limiting substrate and formation of the desired product were determined by reaction control via TLC and HPLC-ELS-MS. All volatiles were removed in fine vacuum. Toluene (3 x 10.0 mL) was added to the residue and all volatiles were again removed in fine vacuum. The crude product (3.86 g) was obtained in the form of orange oil and dissolved in H_2O (1 x 100 mL). The aqueous phase was extracted with Et_2O (3 x 50.0 mL). The collected organic phases were dried over anhydrous Na_2SO_4 and filtered. All volatiles were removed in vacuo, and the residue was dried in fine vacuum. The pure product (**S16**) was obtained in the form of yellowish oil.

Yield: 2.11 g (6.97 mmol, 96 %), (Lit.[26, 27]: 57 – 91 %), yellowish oil.

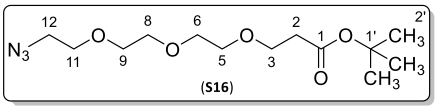

^{1}H-NMR (400 MHz, $CDCl_3$, 25 °C)**:** δ (ppm) = 3.73 – 3.69 (m, 2H, H-3), 3.69 – 3.66 (m, 2H, H-11), 3.66 – 3.58 (m, 8H, H-5, H-6, H-8, H-9), 3.38 (t, ^3J = 5.1 Hz, 2H, H-12), 2.49 (t, ^3J = 6.6 Hz, 2H, H-2), 1.44 (s, 9H, H-2‘). **^{13}C-NMR** (101 MHz, $CDCl_3$, 25 °C)**:** δ (ppm) = 170.9 (1C, C-1), 80.5 (1C, C-1‘), 70.7, 70.6, 70.6, 70.4 (4C, C-5, C-6, C-8, C-9), 70.0 (1C, C-11), 66.9 (1C, C-3), 50.7 (1C, C-12), 36.3 (1C, C-2), 28.1 (1C, C-2‘). **HR-MS** (ESI$^+$)**:** $m/z_{cal.}$ = 326.1686 [M+Na]$^+$, $m/z_{exp.}$ = 326.1692 [M+Na]$^+$; $m/z_{cal.}$ = 327.1716 [M(^{13}C)+Na]$^+$, $m/z_{exp.}$ = 327.1720 [M(^{13}C)+Na]$^+$. ***R$_f$*** (NP) = 0.26 (^{c}Hex/EtOAc 3:1). **IR** (ATR)**:** (cm^{-1}) = 2870, 2104, 1729, 1456, 1393, 1367, 1283, 1255, 1120, 940, 848, 557.

The analytical data follows literature data.[26, 27]

4.1.11 Synthesis of 12-Azido-4,7,10-trioxadodecanic acid

The title compound was synthesized according to Scheme S 13 following modified synthesis protocols by *Landeros et al.*[29] sowie *Krumb et al.*[26]

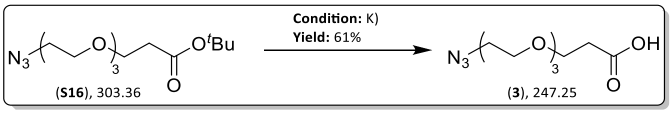

Scheme S 13: Synthesis of 12-Azido-4,7,10-trioxadodecanic acid (**3**). Reaction conditions: K) TFA, DCM, Ar-atm., rt, 22 h; yield: 61 %.

The reaction was carried out in a dried Schlenk vessel previously equipped with a magnetic stirring bar. *tert*-Butyl-12-azido-4,7,10-trioxadodecanoic acid (**S16**, 1.00 g, 3.30 mmol, 1.00 eq.) was dissolved in absolute DCM (5.00 mL) under an atmosphere of argon. TFA (1.51 g, 1.02 mL, 13.2 mmol, 4.00 eq.) was added dropwise to the yellowish solution using a syringe. The reaction mixture was stirred for 22 hours. Complete conversion of the limiting substrate and the formation of the desired product were determined by reaction control via TLC and HPLC-ELS-MS. All volatiles were removed in fine vacuum. Toluene (3 x 10.0 mL) was added to the residue and all volatiles were again removed in fine vacuum. The crude product (0.62 g) was obtained as an orange oil and purified by FCC (^{c}Hex/EtOAc + HCOOH, isocratic at 75 % EtOAc and 1 $\%_{v/v}$ additive). The pure product (**3**) was obtained in the form of colorless oil.

Yield: 0.50 g (2.02 mmol, 61 %), (Lit.[26, 29]: 41 – 94 %), colorless oil.

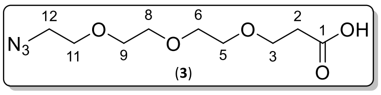

^{1}H-NMR (300 MHz, $CDCl_3$, 25 °C)**:** δ (ppm) = 3.76 (t, ^3J = 6.3 Hz, 2H, H-3), 3.71 – 3.62 (m, 10H, H-5, H-6, H-8, H-9, H-11), 3.39 (t, ^3J = 5.1 Hz, 2H, H-12), 2.64 (t, ^3J = 6.3 Hz, 2H, H-2). **^{13}C-NMR** (75.5 MHz, $CDCl_3$, 25 °C)**:** δ (ppm) = 176.2 (1C, C-1), 70.6, 70.6, 70.4 (4C, C-5, C-6, C-8, C-9), 70.0 (1C, C-11), 66.3 (1C, C-3), 50.7 (1C, C-12), 34.8 (1C, C-2). **HR-MS** (ESI$^+$)**:** $m/z_{ber.}$ = 270.1060 [M+Na]$^+$, $m/z_{exp.}$ = 270.1072 [M+Na]$^+$, $m/z_{Dif.}$ = 4.44 ppm. ***R$_f$*** (NP) = 0.30 (^{c}Hex/EtOAc + HCOOH 1:3 + 1 % additive). **IR** (ATR)**:** (cm^{-1}) = 3489, 2876, 2195, 2105, 1960, 1731, 1442, 1349, 1285, 1192, 1118, 938, 835.

The analytical data follows literature data.[26, 29]

4.1.12 Synthesis of *tert*-Butyl-6-amino-6-(1-*tert*-butyl-4-oxapentanoate-5-yl)-4,8-dioxa-undecane-1,11-dioate

The title compound was synthesized according to Scheme S 14 following modified synthesis protocols by *Cardona et al.*[32], *Appel et al.*[31] sowie *Krumb et al.*[26]

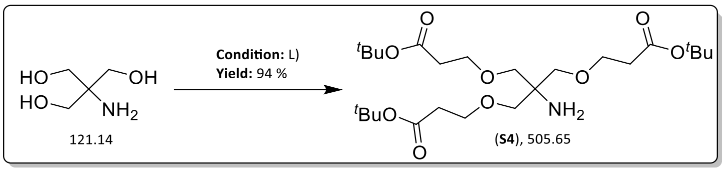

Scheme S 14: Synthesis of *tert*-Butyl-6-amino-6-(1-*tert*-butyl-4-oxapentanoate-5-yl)-4,8-dioxaundecane-1,11-dioate (**S4**). Reaction conditions: L) TBA, NaOH (5 M), DMSO, Ar-atm., 0 °C to rt, 96 h; yield: 94 %.

The reaction was carried out in a dried Schlenk flask previously equipped with a magnetic stirring bar. NaOH (0.10 g, 2.50 mmol, 0.12 eq.) was dissolved in H_2O (0.50 mL) under an atmosphere of argon and this 5 M NaOH solution was degassed in an ultrasonic bath with an argon stream for 30 minutes. TRIS (2.50 g, 20.6 mmol, 1.00 eq.) was dissolved in absolute DMSO (5.00 mL) under an atmosphere of argon and the solution was degassed in an ultrasonic bath with an argon stream for 30 minutes. The latter solution was cooled to 0 °C in an ice bath, and the ice bath was removed. Once the solidified solution had melted, the 5 M NaOH solution was added with stirring using a syringe. After the addition was complete, TBA (26.4 g, 29.9 mL, 206 mmol, 10.0 eq.) was added dropwise to the mixture. The reaction mixture was stirred at room temperature under exclusion of light for 96 hours, resulting in a milky, cloudy solution. Complete conversion of the limiting substrate and the formation of the desired product were determined by reaction control via TLC and HPLC-ELS-MS. All volatiles were removed in fine vacuum. Toluene (3 x 10.0 mL) was added to the residue and all volatiles were again removed under medium vacuum. Further volatiles were removed by lyophilization. The residue was dissolved in EtOAc (1 x 100 mL), and the organic phase was washed with saturated NaCl solution (3 x 100 mL), dried over anhydrous Na_2SO_4, and filtered. All volatiles were removed under reduced pressure, and the residue was dried in fine vacuum. The crude product (10.6 g) was obtained as a colorless oil and purified by FCC (^{c}Hex/EtOAc + NEt_3, isocratic at 25 % EtOAc and 5 $\%_{v/v}$ additive). The pure product (**S4**) was obtained in the form of colorless oil.

Yield: 9.81 g (19.4 mmol, 94 %), (Lit.[26, 31, 32]: 33-50 %), colorless oil.

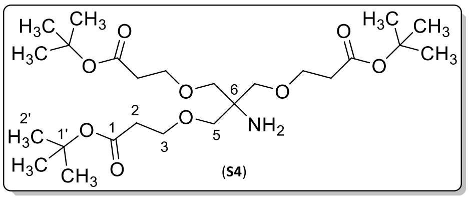

^{1}H-NMR (400 MHz, $CDCl_3$, 25 °C)**:** δ (ppm) = 3.63 (t, ^3J = 6.4 Hz, 6H, H-3), 3.30 (s, 6H, H-5), 2.45 (t, ^3J = 6.4 Hz, 6H, H-2), 1.65 (s, 2H, -N*H*$_2$), 1.44 (s, 27H, H-2'). **^{13}C-NMR** (101 MHz, $CDCl_3$, 25 °C)**:** δ (ppm) = 171.1 (3C, C-1), 80.6 (3C, C-1'), 73.0 (3C, C-5), 67.3 (3C, C-3), 56.1 (1C, C-6), 36.5 (3C, C-2), 28.2 (9C, C-2'). **HR-MS** (ESI$^+$)**:** $m/z_{cal.}$ = 506.3324 [M+H]$^+$, $m/z_{exp.}$ = 506.3318 [M+H]$^+$; $m/z_{cal.}$ = 507.3357 [M(^{13}C)+H]$^+$, $m/z_{exp.}$ = 507.3348 [M(^{13}C)+H]$^+$; $m/z_{cal.}$ = 528.3143 [M+Na]$^+$, $m/z_{exp.}$ = 528.3134 [M+Na]$^+$; $m/z_{cal.}$ = 529.3176 [M(^{13}C)+Na]$^+$, $m/z_{exp.}$ = 529.3167 [M(^{13}C)+Na]$^+$. ***R_f*** = 0.46 (^{c}Hex/EtOAc + NEt_3 3:1 + 5 $\%_{v/v}$ additive). **IR** (ATR)**:** (cm^{-1}) = 2979, 2874, 2255, 1725, 1458, 1393, 1367, 1331, 1255, 1157, 1110, 1069, 907, 846, 728, 648, 464.

The analytical data follows literature data.[26, 31]

4.1.13 Synthesis of 2-Nitrobenzyl-(6-(1-*tert*-butyl-4-oxapentanoat-5-yl)-4,8-dioxa-1,11-di-*tert*-butylundecanoat-6-yl)-carbamate

The title compound was synthesized utilizing a telescoped two-step procedure according to Scheme S 15 following modified synthesis protocols by *Odaka et al.*[34], *Baldoli et al.*[33] and *Krumb et al.*[26]

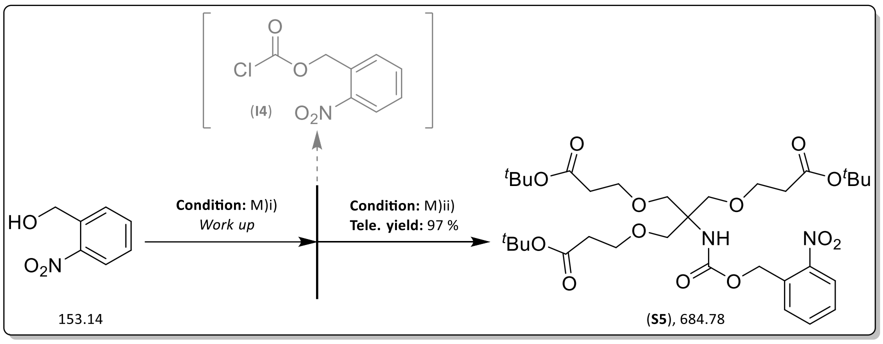

Scheme S 15: Synthesis of 2-Nitrobenzyl-(6-(1-*tert*-butyl-4-oxapentanoate-5-yl)-4,8-dioxa-1,11-di-*tert*-butylundecanoate-6-yl)-carbamate **(S5)**. Reaction conditions: M)i) TCF, THF, 66 °C, 4 h; M)ii) **(S4)**, $NaHCO_3$, EtOAc/H_2O (2:1), RT, 18 h; telescoped yield: 97 %.

The reaction was carried out in a dried Schlenk vessel previously equipped with a magnetic stirring bar. (2-Nitrophenyl)methanol (0.45 g, 2.94 mmol, 1.50 eq.) was dissolved in absolute THF (20.0 mL) under an atmosphere of argon. TCF (0.58 g, 0.35 mL, 2.94 mmol, 1.50 eq.) was slowly added dropwise with a syringe. The yellow reaction solution was stirred for four hours while heated to reflux, with a color change to orange occurring. Complete conversion of the limiting substrate and the formation of the desired product were determined by reaction control via TLC and HPLC-ELS-MS. All volatiles were removed in vacuo and the residue was dried in fine vacuum. The residue (1.12 g) was obtained in the form of an orange oil and dissolved in DCM (50.0 mL). The organic phase was washed with H_2O (2 x 25.0 mL) and saturated NaCl solution (1 x 25.0 mL), dried over anhydrous Na_2SO_4, and filtered. All volatiles were removed in vacuo, and the residue was dried in fine vacuum. The intermediate (0.69 g) was obtained in the form of yellow oil and dissolved in EtOAc (1.00 mL). *tert*-Butyl-6-amino-6-(1-*tert*-butyl-4-oxapentanoate-5-yl)-4,8-dioxaundecane-1,11-dioate (**S4**, 1.00 g, 1.98 mmol, 1.00 eq.) and $NaHCO_3$ (0.33 g, 3.93 mmol, 2.00 eq.) were dissolved in a mixture of EtOAc (2.60 mL) and H_2O (1.80 mL) with stirring. The solution of the intermediate was added dropwise via syringe. The resulting yellow reaction mixture was stirred for 18 hours at room temperature. Complete conversion of the limiting substrate and the formation of the desired product were determined by reaction control via TLC and HPLC-ELS-MS. The reaction mixture was diluted with EtOAc (1 x 50.0 mL) and washed with H_2O (1 x 10.0 mL). The aqueous phase was extracted with EtOAc (3 x 30.0 mL). The collected organic phases were washed with H_2O (1 x 30.0 mL) and saturated NaCl solution (1 x 30.0 mL), dried over anhydrous Na_2SO_4, and filtered. All volatiles were removed in vacuo, and the residue was dried in fine vacuum. The crude product (1.52 g) was obtained as a yellowish oil and purified by FCC (^{c}Hex/EtOAc, isocratic at 20 % EtOAc). The pure product (**S5**) was obtained in the form of colorless oil.

Yield: 1.31 g (1.91 mmol, 97 %), colorless oil.

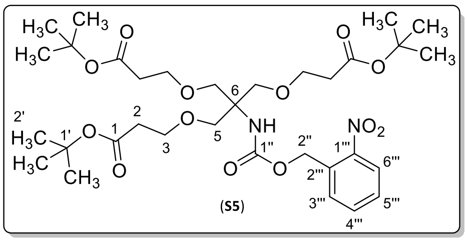

^{1}H-NMR (400 MHz, $CDCl_3$, 25 °C)**:** *δ* (ppm) = 8.15 – 8.08 (m, 1H, H-6’’’), 7.67 – 7.66 (m, 1H, H-5’’’), 7.66 – 7.64 (m, 1H, H-3’’’), 7.50 – 7.38 (m, 1H, H-4’’’), 5.51 (s, 1H, -N*H*-), 5.47 (s, 2H, H-2’’), 3.66 (s, 6H, H-5), 3.64 (d, ^3J = 6.3 Hz, 6H, H-3), 2.45 (t, ^3J = 6.3 Hz, 6H, H-2), 1.44 (s, 27H, H-2’). **^{13}C-NMR** (101 MHz, $CDCl_3$, 25 °C)**:** *δ* (ppm) = 170.8 (3C, C-1), 154.4 (1C, C-1’’), 147.0 (1C,

C-1’’’), 134.0 (1C, C-2’’’), 133.8 (1C, C-3’’’), 128.2 (1C, C-5’’’), 128.1 (1C, C-4’’’), 124.9 (1C, C-6’’’), 80.5 (1C, C-1’), 69.3 (1C, C-5), 67.1 (1C, C-3), 62.6 (1C, C-2’’), 58.8 (1C, C-6), 36.2 (1C, C-2), 28.1 (1C, C-2’). **HR-MS** (ESI$^+$)**:** *m/z*$_{cal.}$ = 707.3362 [M+Na]$^+$, *m/z*$_{exp.}$ = 707.3352 [M+Na]$^+$. ***R$_f$*** (NP) = 0.28 (^{c}Hex/EtOAc 5:1). **IR** (ATR)**:** (cm^{-1}) = 2978, 2930, 2870, 2252, 2246, 1725, 1528, 1440, 1347, 1266, 1159, 1109, 905, 860, 808, 786, 725, 649, 480.

The analytical data follows literature data.[26]

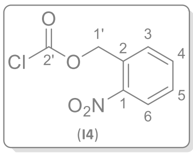

^{1}H-NMR (400 MHz, $CDCl_3$, 25 °C)**:** *δ* (ppm) = 8.21 – 8.18 (m, 1H, H-6), 7.77 – 7.70 (m, 1H, H-4), 7.67 – 7.63 (m, 1H, H-3), 7.61 – 7.54 (m, 1H, H-5), 5.75 (s, 2H, H-1’). **^{13}C-NMR** (101 MHz, $CDCl_3$, 25 °C)**:** *δ* (ppm) = 150.5 (1C. C-2’’), 147.1 (1C, C-1), 134.3 (1C, C-4), 129.7 (1C, C-5), 129.6 (1C, C-2), 129.0 (1C, C-3), 125.5 (1C, C-6), 69.5 (1C, C-1’). **HR-MS** (ESI$^+$)**:** *m/z*$_{cal.}$ = 136.0399 [M-O(C=O)Cl]$^+$, *m/z*$_{exp.}$ = 136.0396 [M-O(C=O)Cl]$^+$. ***R$_f$*** (NP) = 0.65 (^{c}Hex/DCM 1:3). **IR** (ATR): (cm^{-1}) = 1775, 1526, 1344, 1309, 1138, 857, 791, 729, 686.

The analytical data follows literature data.[47]

4.1.14 Synthesis of 2-Nitrobenzyl-(6-(1-carboxy-4-oxapentan-5-yl)-4,8-dioxa-1-11-dicarboxyundecan-6-yl)-carbamate

The title compound was synthesized according to Scheme S 16 following modified synthesis protocols by *Landeros et al.*[29], *Spritzer et al.*[35] and *Krumb et al.*[26]

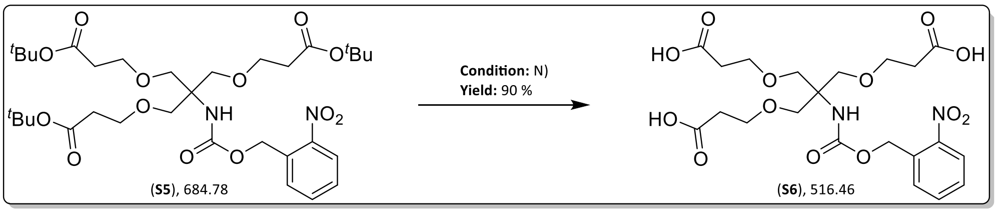

Scheme S 16: Synthesis of 2-Nitrobenzyl-(6-(1-carboxy-4-oxapentan-5-yl)-4,8-dioxa-1-11-dicarboxyundecan-6-yl)-carbamate (**S6**). Reaction conditions: N) TFA, DCM, rt, 72 h; yield: 90 %.

The reaction was carried out in a dried Schlenk vessel previously equipped with a magnetic stirring bar. 2-Nitrobenzyl-(6-(1-*tert*-butyl-4-oxapentanoate-5-yl)-4,8-dioxa-1,11-di-*tert*-butylundecanoate-6-yl)-carbamate (**S5**, 3.25 g, 4.75 mmol, 1.00 eq.) was dissolved in absolute DCM (20.0 mL) under an atmosphere of argon. TFA (5.42 g, 3.66 mL, 47.5 mmol, 10.0 eq.) was slowly added dropwise via syringe, and the reaction mixture was stirred for 72 hours at room temperature. Complete conversion of the limiting substrate and the formation of the desired product were determined by reaction control via TLC and HPLC-ELS-MS. All volatiles were removed under in vacuo. Toluene (3 x 20.0 mL) was added to the residue and all volatiles were again removed under reduced pressure and the residue dried in fine vacuum. The crude product (2.54 g) was obtained in the form of a yellowish oil and purified by FCC (^{c}Hex/EtOAc + HCOOH, isocratic at 66 % EtOAc and 1 %$_{v/v}$ additive). The pure product (**S6**) was obtained in the form of colorless oil.

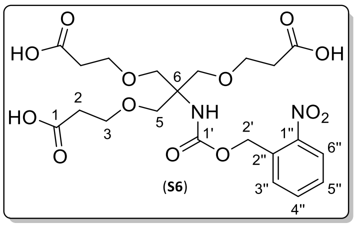

^{1}H-NMR (400 MHz, $(CD_3)_2SO$, 25 °C)**:** *δ* (ppm) = 8.16 – 8.09 (m, 1H, H-6’’), 7.85 – 7.75 (m, 1H, H-4’’), 7.71 – 7.64 (m, 1H, H-3’’), 7.64 – 7.55 (m, 1H, H-5’’), 6.85 (s, 1H, -N*H*-), 5.34 (s, 2H, H-2’), 3.57 (t, ^3J = 6.3 Hz, 6H, H-3), 3.49 (s, 6H, H-5), 3.17 (s, 3H, -OH), 2.42 (t, ^3J = 6.3 Hz, 6H, H-2). **^{13}C-NMR** (101 MHz, $(CD_3)_2SO$, 25 °C)**:** *δ* (ppm) = 172.6 (3C, C-1), 154.0 (1C, C-1‘), 146.8 (1C, C-1‘‘), 134.1 (1C, C-4‘‘), 133.3 (1C, C-2‘‘), 128.7 (1C, C-5‘‘), 128.3 (1C, C-3‘‘), 124.7 (1C, C-6‘‘), 67.9 (3C, C-5), 66.6 (3C, C-3), 61.5 (1C, C-2’), 58.9 (1C, C-6), 34.5 (3C, C-2). **HR-MS** (ESI$^-$)**:** *m/z*$_{cal.}$ = 515.1519

[M-H]$^-$, $m/z_{exp.}$ = 515.1517 [M-H]$^-$. R_f (NP) = 0.21 (^{c}Hex/EtOAc + HCOOH 1:2 + 1 $\%_{v/v}$ additive). **IR** (ATR): (cm^{-1}) = 2927, 2881, 1709, 1524, 1422, 1343, 1236, 1188, 1094, 1068, 859, 791, 732, 702, 590, 474.
The analytical data follows literature data.[26]

4.1.15 Synthesis of 2-Nitrobenzyl-(1,35-diazido-18-(17-azido-5-oxo-2,9,12,15-tetraoxa-6-azaheptadecyl)-13,23-dioxo-3,6,9,16,20,27,30,33-octaoxa-12,24-diazapentatriacontan-18-yl)-carbamate

The title compound was synthesized according to Scheme S 17 following modified synthesis protocols by *Appel et al.*[31], *Krumb et al.*[26] and *Zhou et al.*[48]

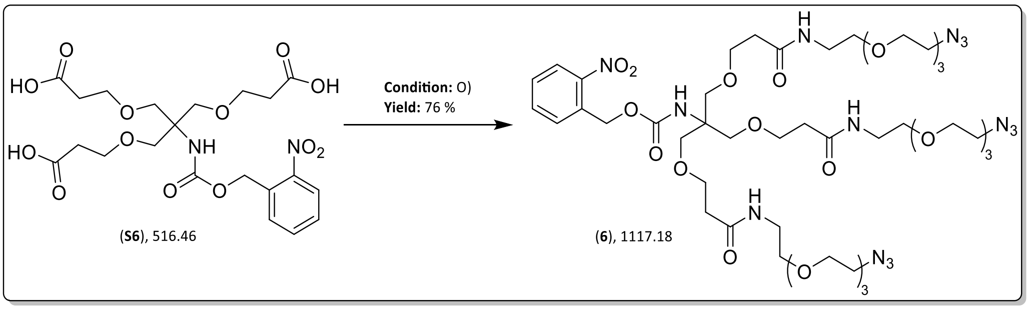

Scheme S 17: Synthesis of 2-Nitrobenzyl-(1,35-diazido-18-(17-azido-5-oxo-2,9,12,15-tetraoxa-6-azaheptadecyl)-13,23-dioxo-3,6,9,16,20,27,30,33-octaoxa-12,24-diazapentatriacontan-18-yl)-carbamate **(6)**. Reaction conditions: O) **(4)**, HATU, DIPEA, DMF, Ar-atm., rt, 48 h; yield: 76 %.

The reaction was carried out in a dried Schlenk vessel previously equipped with a magnetic stirring bar. 2-Nitrobenzyl-(6-(1-carboxy-4-oxapentan-5-yl)-4,8-dioxa-1-11-dicarboxyundecan-6-yl)-carbamate (**S6**, 0.97 g, 1.88 mmol, 1.00 eq.) was dissolved in absolute DMF (25.0 mL) under an atmosphere of argon. HATU (2.14 g, 5.64 mmol, 3.00 eq.) was added, followed by DIPEA (0.97 g, 1.28 mL, 7.52 mmol, 4.00 eq.). The reaction mixture was stirred for half an hour at room temperature. 11-Amino-1-azido-3,6,9-trioxaundecane (**4**, 1.64 g, 7.52 mmol, 4.00 eq.) was dissolved in absolute DMF (5.00 mL) under an atmosphere of argon and slowly added dropwise to the yellowish reaction mixture using a syringe. The mixture was stirred at room temperature for 48 hours. Complete conversion of the limiting substrate and the formation of the desired product were determined by reaction control via TLC and HPLC-ELS-MS. All volatiles were removed in fine vacuum. Toluene (3 x 20.0 mL) was added to the residue and all volatiles were again removed under fine vacuum. The crude product (4.02 g) was obtained in the form of an orange oil and purified by FCC (EtOAc/MeOH, gradient from 10 % to 30 % MeOH) followed by RP-FCC (MeCN/H_2O, gradient from 10 % to 60 % to 100 % MeCN, Isolera One Purification System, SNAP C_{18} 60 g cartridge). The pure product (**6**) was obtained in the form of colorless oil

Yield: 1.60 g (1.43 mmol, 76 %), colorless oil.

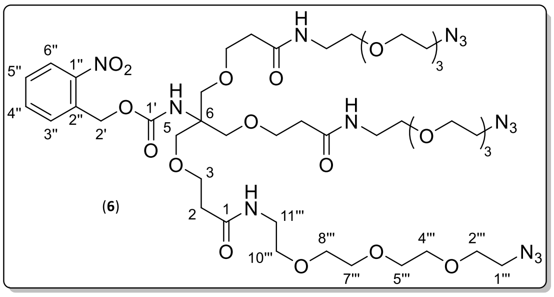

^{1}H-NMR (600 MHz, $CDCl_3$, 25 °C)**:** δ (ppm) = 8.15 – 8.02 (m, 1H, H-6''), 7.69 – 7.64 (m, 2H, H-3'', H-4''), 7.49 – 7.45 (m, 1H, H-5''), 6.70 (t, ^3J = 5.4 Hz, 3H, -N*H*-C-11'''), 5.71 (s, 1H, -N*H*-C-6), 5.45 (s, 2H, H-2'), 3.70 (t, ^3J = 5.9 Hz, 6H, H-2), 3.67 – 3.60 (m, 36H, H-5, H-2''', H-4''', H-5''', H-7''', H-8'''), 3.54 (t, ^3J = 5.4 Hz, 6H, H-10'''), 3.43 (q, ^3J = 5.4 Hz, 6H, H-11'''), 3.38 (t, ^3J = 5.1 Hz, 6H, H-1'''), 2.42 (t, ^3J = 5.9 Hz, 6H, H-3). **^{13}C-NMR** (151 MHz, $CDCl_3$, 25 °C)**:** δ (ppm)

= 171.2 (3C, C-1), 154.6 (1C, C-1'), 147.2 (1C, C-1''), 133.9 (1C, C-4''), 133.4 (1C, C-2''), 128.7 (1C, C-3''), 128.5 (1C, C-5''), 125.0 (3C, C-6''), 70.7, 70.6, 70.5, 70.2, 70.0 (15C, C-2''', C-4''', C-5''', C-7''', C-8'''), 69.8 (3C, C-10'''), 69.2 (3C, C-5), 67.4 (3C, C-2), 62.9 (1C, C-2'), 58.9 (1C, C-6), 50.6 (3C, C-1'''), 39.2 (3C, C-11'''), 36.6 (3C, C-3). **HR-MS** (ESI)**:** $m/z_{cal.}$ = 581.2598 $[M+2Na]^{2+}$, $m/z_{exp.}$ = 581.2600 $[M+2Na]^{2+}$; $m/z_{cal.}$ = 1139.5303 $[M+Na]^{+}$, $m/z_{exp.}$ = 1139.5307 $[M+Na]^{+}$. ***R_f*** (NP) = 0.44 (EtOAc/MeOH 7:3). **IR** (ATR)**:** (cm^{-1}) = 3310, 2872, 2101, 1726, 1651, 1525, 1344, 1283, 1092, 912, 858, 793, 729, 646, 556.

The analytical data follows literature data.[26]

4.1.16 Synthesis of (1-(11-Azido-3,6,9-trioxaundec-1-yl)-1*H*-1,2,3-triazol-4-yl)-methyl α-D-Mannopyranoside

The title compound was synthesized according to Scheme S 18 following a modified synthesis protocol by *Yamamoto et al.*[39]

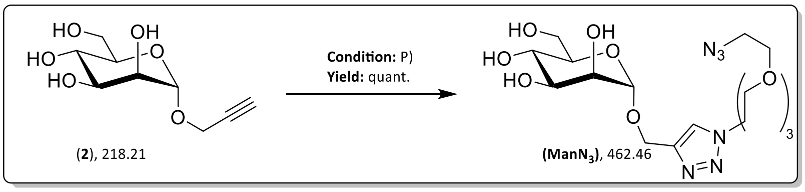

Scheme S 18: Synthesis of (1-(11-Azido-3,6,9-trioxaundec-1-yl)-1*H*-1,2,3-triazol-4-yl)-methyl α-D-Mannopyranoside (**ManN$_3$**). Reaction conditions: P) Cu^{I}Br, NaAsc, DMF, Ar-atm., 45 °C, 20 h; yield: quant.

The reaction was carried out in a dried Schlenk vessel previously equipped with a magnetic stirring bar. Propargyl α-D-mannopyranoside (**2**, 0.10 g, 0.46 mmol, 1.00 eq.) and 1,11-diazido-3,6,9-trioxaundecane (**5**, 0.56 g, 2.29 mmol, 5.00 eq.) were dissolved under an atmosphere of argon in absolute DMF (10.0 mL), which had previously been degassed for half an hour in an ultrasonic bath with an argon stream. The mixture was degassed by three freeze-pump-thaw cycles and then brought under an atmosphere of argon. PDMTA (4.00 mg, 5.00 µL, 23.0 µmol, 0.05 eq.) was added using an Eppendorf pipette. The mixture was warmed up to a temperature of 45 °C, and Cu^{I}Br (3.30 g, 23.0 µmol, 0.05 eq.) was added. The turquoise-colored reaction mixture was stirred at this temperature for 20 hours. Complete conversion of the limiting substrate and the formation of the desired product were determined by reaction control via TLC and HPLC-ELS-MS. The solution was cooled to room temperature. All volatiles were removed in fine vacuum. Toluene (3 x 5.00 mL) was added to the residue, and all volatiles were again removed in fine vacuum. The crude product (0.74 g) was obtained as a yellow liquid and purified by FCC (EtOAc/MeOH, gradient from 0 % to 5 % to 10 % MeOH, Isolera Four Purification System, SNAP KP-Sil 50 g cartridge) and RP-FCC (MeCN/H_2O, gradient from 3 % to 5 % to 100 % MeCN, Isolera One Purification System, SNAP C_{18} 30 g cartridge). The pure product (**ManN$_3$**) was obtained in the form of colorless oil. The excess of the starting material 1,11-diazido-3,6,9-trioxaundecane (**5**) was also recovered with a recovery rate of 92 %.

Yield: 0.21 g (0.46 mmol, quant.), colorless oil.

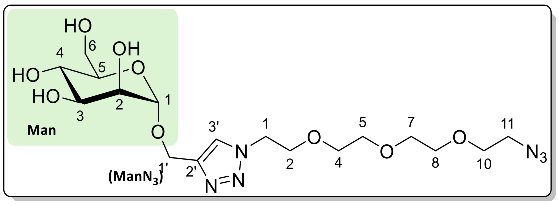

^{1}H-NMR (600 MHz, CD_3OD, 25 °C)**:** δ (ppm) = 8.08 (s, 1H, H-3'), 4.85 (d, ^{3}J = 1.7 Hz, 1H, H-1$_{Man}$), 4.80 (d, ^{2}J = 12.4 Hz, 1H, H-1a'), 4.65 (d, ^{2}J = 12.4 Hz, 1H, H-1b'), 4.59 (t, ^{3}J = 5.3 Hz, 2H, H-1), 3.90 (t, ^{3}J = 5.1 Hz, 2H, H-2), 3.85 (dd, ^{2}J = 11.8 Hz, ^{3}J = 2.2 Hz, 1H, H-6a$_{Man}$), 3.78 (dd, ^{3}J = 3.4, 1.7 Hz, 1H, H-2$_{Man}$), 3.72 (dd, ^{2}J = 11.8 Hz, ^{3}J = 5.9 Hz, 1H, H-6b$_{Man}$), 3.67 (dd, ^{3}J = 9.1, 3.4 Hz, 1H, H-3$_{Man}$), 3.67 – 3.65 (m, 2H, H-10), 3.65 – 3.59 (m, 9H, H-4$_{Man}$, H-4, H-5, H-7, H-8),

3.57 (ddd, $^3J = 9.7$, 5.8, 2.2 Hz, 1H, H-5$_{Man}$), 3.37 (t, $^3J = 4.9$ Hz, 2H, H-11). **^{13}C-NMR** (151 MHz, CD_3OD, 25 °C)**:** δ (ppm) = 142.9 (1C, C-2‘), 124.1 (1C, C-3‘), 98.6 (1C, C-1$_{Man}$), 72.8 (1C, C-5$_{Man}$), 70.4 (1C, C-3$_{Man}$), 69.9 (1C, C-2$_{Man}$), 69.4, 69.4, 69.3 (4C, C-4, C-5, C-7, C-8), 69.0 (1C, C-10), 68.2 (1C, C-2), 66.4 (1C, C-4$_{Man}$), 60.8 (1C, C-6$_{Man}$), 58.5 (1C, C-1‘), 49.6 (1C, C-11), 49.3 (1C, C-1). **HR-MS** (ESI$^+$)**:** $m/z_{cal.} = 485.1966$ [M+Na]$^+$, $m/z_{exp.} = 485.1963$ [M+Na]$^+$. ***R_f*** (NP) = 0.23 (EtOAc/MeOH 10:3). **Optical rotation** (LM): $[\alpha]_D^{22}$ = +50.1 (MeOH). **IR** (ATR): (cm^{-1}) = 3370, 2875, 2108, 1452, 1350, 1300, 1127, 1100, 1060, 816, 680, 557, 511.

The analytical data follows literature data.[37]

4.1.17 Synthesis of (1-(11-Azido-3,6,9-trioxaundec-1-yl)-1*H*-1,2,3-triazol-4-yl)-methyl 2,4-Di-*O*-benzoyl-3,6-di-*O*-(2,3,4,6-tetra-*O*-acetyl-α-D-mannopyranosyl)-α-D-mannopyranoside

The title compound was synthesized according to Scheme S 19 following modified synthesis protocols by *Kramer et al.*[6] and *Yamamoto et al.*[39]

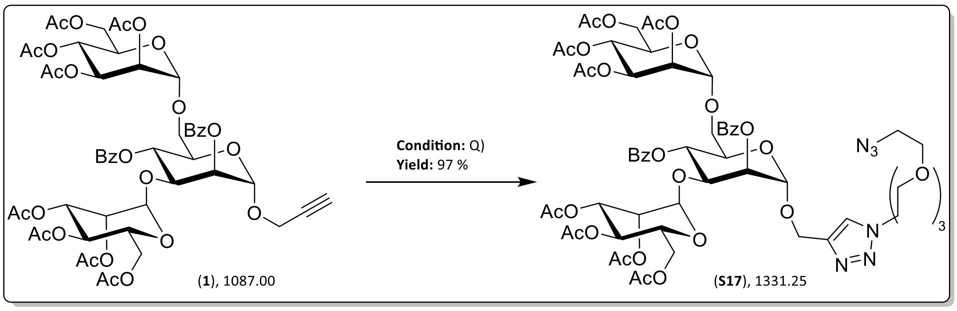

Scheme S 19: Synthesis of (1-(11-Azido-3,6,9-trioxaundec-1-yl)-1*H*-1,2,3-triazol-4-yl)-methyl 2,4-Di-*O*-benzoyl-3,6-di-*O*-(2,3,4,6-tetra-*O*-acetyl-α-D-mannopyranosyl)-α-D-mannopyranoside (**S17**). Reaction conditions: Q) (**5**), Cu^{I}Br, PMDTA, DMF, Ar-atm., 45 °C, 3 h; yield: 97 %

The reaction was carried out in a dried Schlenk vessel previously equipped with a magnetic stirring bar. Propargyl 2,4-di-O-benzoyl-3,6-di-O-(2,3,4,6-tetra-O-acetyl-α-D-mannopyranosyl)-α-D-mannopyranoside (**1**, 0.50 g, 0.46 mmol, 1.00 eq.) was dissolved together with 1,11-diazido-3,6,9-trioxaundecane (**5**, 1.69 g, 6.90 mmol, 15.0 eq.) under an atmosphere of argon in absolute DMF (45.0 mL), which had previously been degassed for half an hour in an ultrasonic bath with an argon stream. The mixture was degassed using three freeze-pump-thaw cycles and returned to an argon atmosphere. PDMTA (0.07 g, 0.08 mL, 0.39 mmol, 0.85 eq.) was added using an Eppendorf pipette. The mixture was warmed up to a temperature of 45 °C, and Cu^{I}Br (0.03 g, 0.23 mmol, 0.50 eq.) was added. The turquoise-colored reaction solution was stirred at this temperature for three hours. Complete conversion of the limiting substrate and formation of the desired product were determined by reaction control via TLC and HPLC-ELS-MS. The solution was cooled to room temperature, diluted with EtOAc (1 x 100 mL), and washed with saturated NH_4Cl solution (2 x 50.0 mL). The aqueous phase was diluted with H_2O (1 x 50.0 mL) until all salts were dissolved and extracted with EtOAc (1 x 50.0 mL). The collected organic phases were washed with saturated NaCl solution (1 x 100 mL), dried over anhydrous Na_2SO_4, and filtered. All volatiles were removed in fine vacuum. The crude product (2.56 g) was obtained in the form of a yellowish liquid and purified by FCC (^{c}Hex/EtOAc, gradient 0 % to 100 % EtOAc, Isolera Four Flash Purification System, SNAP KP-Sil 100 g cartridge). The pure product (**S17**) was obtained in the form of yellowish oil. The excess of the starting material 1,11-diazido-3,6,9-trioxa-undecane (**5**) was also recovered with a recovery rate of 90 %.

Yield: 0.60 g (0.45 mmol, 97 %), yellowish oil.

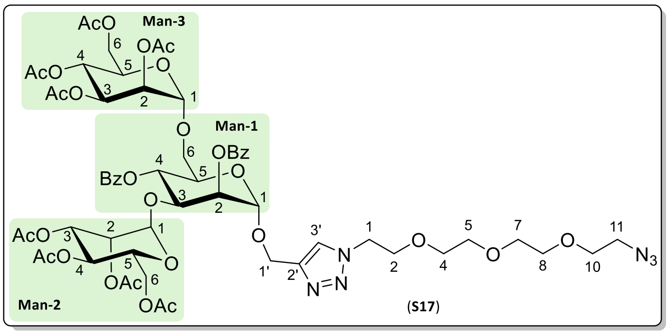

^{1}H-NMR (600 MHz, $CDCl_3$, 25 °C): δ (ppm) = 8.16 – 8.13 (m, 2H, C-2$_{\text{Man-1}}$-O(C=O)Ph, H$_{ortho}$), 8.04 – 8.01 (m, 2H, C-4$_{\text{Man-1}}$-O(C=O)Ph, H$_{ortho}$), 7.85 (s, 1H, H-3‘), 7.64 – 7.60 (m, 1H, C-2$_{\text{Man-1}}$-O(C=O)Ph, H$_{para}$), 7.60 – 7.57 (m, 1H, C-4$_{\text{Man-1}}$-O(C=O)Ph, H$_{para}$), 7.57 – 7.53 (m, 2H, C-2$_{\text{Man-1}}$-O(C=O)Ph, H$_{meta}$), 7.46 – 7.42 (m, 2H, C-4$_{\text{Man-1}}$-O(C=O)Ph, H$_{meta}$), 5.70 (t$_{\text{app}}$, ^{3}J = 10.0 Hz, 1H, H-4$_{\text{Man-1}}$), 5.52 (dd, ^{3}J = 3.4, 1.8 Hz, 1H, H-2$_{\text{Man-1}}$), 5.34 (dd, ^{3}J = 10.1, 3.5 Hz, 1H, H-3$_{\text{Man-3}}$), 5.28 (dd, ^{3}J = 3.5, 1.7 Hz, 1H, H-2$_{\text{Man-3}}$), 5.25 (t$_{\text{app}}$, ^{3}J = 10.1 Hz, 1H, H-4$_{\text{Man-3}}$), 5.17 (d, ^{3}J = 1.8 Hz, 1H, H-1$_{\text{Man-1}}$), 5.10 – 5.04 (m, 2H, H-3$_{\text{Man-2}}$, H-4$_{\text{Man-2}}$), 4.94 (d, ^{3}J = 1.9 Hz, 1H, H-1$_{\text{Man-2}}$), 4.89 (d, ^{2}J = 12.1 Hz, 1H, H-1a‘), 4.86 (d, ^{3}J = 1.7 Hz, 1H, H-1$_{\text{Man-3}}$), 4.85 (dd, ^{3}J = 3.0, 1.9 Hz, 1H, H-2$_{\text{Man-2}}$), 4.72 (d, ^{2}J = 12.1 Hz, 1H, H-1b‘), 4.58 (t, ^{3}J = 5.2 Hz, 2H, H-1), 4.46 (dd, ^{3}J = 10.0, 3.4 Hz, 1H, H-3$_{\text{Man-1}}$), 4.25 (ddd, ^{3}J = 10.0, 6.1, 2.3 Hz, 1H, H-5$_{\text{Man-1}}$), 4.18 – 4.12 (m, 2H, H-6a$_{\text{Man-2}}$, H-6a$_{\text{Man-3}}$), 4.08 – 4.03 (m, 2H, H-5$_{\text{Man-2}}$, H-5$_{\text{Man-3}}$), 3.97 (dd, ^{2}J = 12.2, ^{3}J = 2.1 Hz, 1H, H-6b$_{\text{Man-3}}$), 3.96 – 3.93 (m, 2H, H-6a$_{\text{Man-1}}$, H-6b$_{\text{Man-2}}$), 3.93 – 3.89 (m, 2H, H-2), 3.69 – 3.59 (m, 11H, H-6b$_{\text{Man-1}}$, H-4, H-5, H-7, H-8, H-10), 3.38 (t, ^{3}J = 5.0 Hz, 2H, H-11), 2.11 (s, 3H, C-2$_{\text{Man-3}}$-O(C=O)CH_3), 2.04 (s, 3H, C-4$_{\text{Man-3}}$-O(C=O)CH_3), 2.03 (s, 3H, C-6$_{\text{Man-3}}$-O(C=O)CH_3), 1.96 (s, 3H, C-3$_{\text{Man-3}}$-O(C=O)CH_3), 1.93 (s, 3H, C-6$_{\text{Man-2}}$-O(C=O)CH_3), 1.92 (s, 3H, C-4$_{\text{Man-2}}$-O(C=O)CH_3), 1.84 (s, 3H, C-2$_{\text{Man-2}}$-O(C=O)CH_3), 1.81 (s, 3H, C-3$_{\text{Man-2}}$-O(C=O)CH_3). **^{13}C-NMR** (151 MHz, $CDCl_3$, 25 °C): δ (ppm) = 170.7 (1C, C-6$_{\text{Man-2}}$-O(C=O)CH_3), 170.6 (1C, C-6$_{\text{Man-3}}$-O(C=O)CH_3), 169.9 (1C, C-2$_{\text{Man-3}}$-O(C=O)CH_3), 169.9 (1C, C-4$_{\text{Man-3}}$-O(C=O)CH_3), 169.8 (1C, C-4$_{\text{Man-2}}$-O(C=O)CH_3), 169.6 (1C, C-3$_{\text{Man-3}}$-O(C=O)CH_3), 169.1 (1C, C-2$_{\text{Man-2}}$-O(C=O)CH_3), 169.1 (1C, C-3$_{\text{Man-2}}$-O(C=O)CH_3), 165.9 (1C, C-2$_{\text{Man-1}}$-O(C=O)Ph), 165.2 (1C, C-4$_{\text{Man-1}}$-O(C=O)Ph), 142.9 (1C, C-2‘), 133.6 (1C, C-2$_{\text{Man-1}}$-O(C=O)Ph, C$_{para}$), 133.6 (1C, C-4$_{\text{Man-1}}$-O(C=O)Ph, C$_{para}$), 130.0 (2C, C-2$_{\text{Man-1}}$-O(C=O)Ph, C$_{ortho}$), 129.9 (2C, C-4$_{\text{Man-1}}$-O(C=O)Ph, C$_{ortho}$), 129.1 (1C, C-4$_{\text{Man-1}}$-O(C=O)Ph, C$_{ipso}$), 128.8 (2C, C-2$_{\text{Man-1}}$-O(C=O)Ph, C$_{meta}$), 128.7 (1C, C-2$_{\text{Man-1}}$-O(C=O)Ph, C$_{ipso}$), 128.5 (2C, C-4$_{\text{Man-1}}$-O(C=O)Ph, C$_{meta}$), 124.5 (1C, C-3‘), 99.4 (1C, C-1$_{\text{Man-2}}$), 97.3 (1C, C-1$_{\text{Man-3}}$), 96.7 (1C, C-1$_{\text{Man-1}}$), 75.5 (1C, C-3$_{\text{Man-1}}$), 71.6 (1C, C-2$_{\text{Man-1}}$), 70.7, 70.6, 70.6, 70.5, 70.1 (5C, C-4, C-5, C-7, C-8, C-10), 69.4 (1C, C-5$_{\text{Man-1}}$), 69.4 (1C, C-2), 69.3 (1C, C-5$_{\text{Man-2}}$), 69.2 (1C, C-2$_{\text{Man-3}}$), 69.2 (1C, C-2$_{\text{Man-2}}$), 69.2 (1C, C-3$_{\text{Man-3}}$), 68.6 (1C, C-5$_{\text{Man-3}}$), 68.2 (1C, C-3$_{\text{Man-2}}$), 66.5 (1C, C-6$_{\text{Man-1}}$), 65.9 (1C, C-4$_{\text{Man-2}}$), 65.8 (1C, C-4$_{\text{Man-3}}$), 62.3 (1C, C-6$_{\text{Man-3}}$), 62.2 (1C, C-6$_{\text{Man-2}}$), 60.5 (1C, C-1‘), 50.6 (1C, C-11), 50.2 (1C, C-1), 20.9 (1C, C-2$_{\text{Man-3}}$-O(C=O)CH_3), 20.8 (1C, C-6$_{\text{Man-3}}$-O(C=O)CH_3), 20.7 (1C, C-3$_{\text{Man-3}}$-O(C=O)CH_3), 20.7 (1C, C-4$_{\text{Man-3}}$-O(C=O)CH_3), 20.6 (1C, C-6$_{\text{Man-2}}$-O(C=O)CH_3), 20.5(1C, C-2$_{\text{Man-2}}$-O(C=O)CH_3), 20.5 (1C, C-3$_{\text{Man-2}}$-O(C=O)CH_3). **HR-MS** (ESI^{+}): $m/z_{\text{cal.}}$ = 1353.4392 [M+Na]$^{+}$, $m/z_{\text{exp.}}$ = 1353.4375 [M+Na]$^{+}$. $\boldsymbol{R_f}$ (NP) = 0.25 (EtOAc). **Optical rotation** (LM): $[\alpha]_D^{20}$ = 10.6 ($CHCl_3$). **IR** (ATR): (cm^{-1}) = 2942, 2103, 1749, 1451, 1370, 1226, 1134, 1087, 1070, 1048, 980, 936, 715, 653, 600, 529, 479, 466, 450.

4.1.18 Synthesis of (1-(11-Azido-3,6,9-trioxaundec-1-yl)-1*H*-1,2,3-triazol-4-yl)-methyl 3,6-Di-*O*-(α-D-mannopyranosyl)-α-D-mannopyranoside

The title compound was synthesized according to Scheme S 20 following modified synthesis protocols by *Kramer et al.*[6], *Lindhorst et al.*[40] and *Reina et al.*[41]

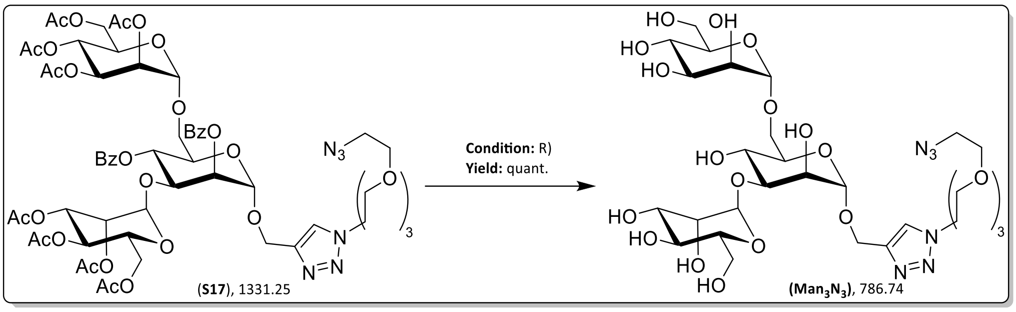

Scheme S 20: Synthesis of (1-(11-Azido-3,6,9-trioxaundec-1-yl)-1*H*-1,2,3-triazol-4-yl)-methyl 3,6-Di-*O*-(α-D-mannopyranosyl)-α-D-mannopyranoside **(Man_3N_3)**. Reaction conditions: R) NaOMe, NaOH, MeOH, Ar-atm., rt, 24 h; yield: quant.

The reaction was carried out in a dried Schlenk vessel previously equipped with a magnetic stirring bar. 1-(11-Azido-3,6,9-trioxaundec-1-yl)-1H-1,2,3-triazol-4-yl)-methyl 2,4-Di-O-benzoyl-3,6-di-O-(2,3,4,6-tetra-O-acetyl-α-D-mannopyranosyl)-α-D-mannopyranoside (**S17**, 0.27 g, 0.21 mmol, 1.00 eq.) was dissolved in absolute MeOH (5.00 mL) under an atmosphere of argon. NaOMe (11.3 mg, 0.21 mmol, 1.00 eq.) and NaOH (8.40 mg, 0.21 mmol, 1.00 eq.) were added successively. The reaction mixture was stirred for four hours at a temperature of 40 °C. Complete conversion of the limiting substrate and formation of the desired product were determined by TLC and HPLC-ELS-MS. The reaction was terminated by adding the ion exchange resin *Amberlite IR 120* (0.20 g), and the mixture was stirred for ten minutes until a constant pH value (pH = 6–7) was reached. The reaction mixture was filtered through a glass frit with *Celite* and eluted with MeOH (1 x 100 mL). All volatiles were removed in vacuo. The residue was obtained as a yellowish oil, dissolved in H_2O (1 x 20.0 mL), and extracted with Et_2O (3 x 20.0 mL). The crude product (0.10 g) was obtained as a yellowish oil by lyophilization of the aqueous phase and purified by RP-FCC (MeCN/H_2O, gradient from 5 % to 60 % to 100 % MeCN, Isolera One Flash Purification System, SNAP C_{18} 12 g cartridge). The pure product (**Man_3N_3**) was obtained in the form of a colorless lyophilizate.

Yield: 0.17 g (0.21 mmol, quant.), colorless lyophilizate.

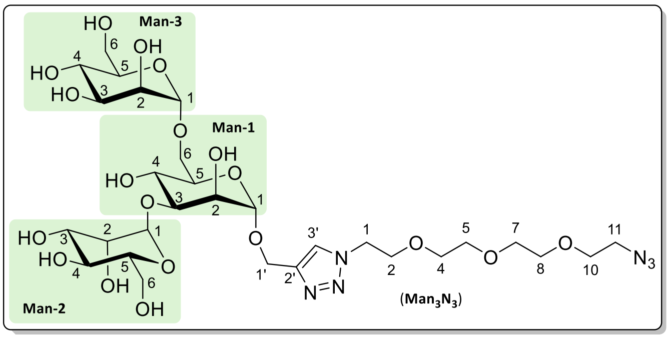

^{1}H-NMR (600 MHz, D_2O, 25 °C)**:** δ (ppm) = 8.07 (s, 1H, H-3‘), 5.03 (s, 1H, H-1$_{\text{Man-2}}$), 4.89 (s, 1H, H-1$_{\text{Man-1}}$), 4.85 (s, 1H, H-1$_{\text{Man-3}}$), 4.78 – 4.77 (m, 1H, H-1a‘), 4.68 (d, ^2J = 12.6 Hz, 1H, H-1b‘), 4.60 (t, ^3J = 5.0 Hz, 2H, H-1), 4.05 (s, 1H, H-2$_{\text{Man-1}}$), 4.01 (dd, ^3J = 3.5, 1.6 Hz, 1H, H-2$_{\text{Man-2}}$), 3.98 – 3.92 (m, 4H, H-2, H-2$_{\text{Man-3}}$, H-6a$_{\text{Man-1}}$), 3.90 – 3.85 (m, 1H, H-4$_{\text{Man-1}}$), 3.85 – 3.78 (m, 4H, H-3$_{\text{Man-1}}$, H-3$_{\text{Man-2}}$, H-3$_{\text{Man-3}}$, H-6a$_{\text{Man-3}}$), 3.76 – 3.69 (m, 3H, H-5$_{\text{Man-1}}$, H-5$_{\text{Man-2}}$, H-6b$_{\text{Man-3}}$), 3.68 – 3.55 (m, 16H, H-4, H-5, H-7, H-8, H-10, H-6b$_{\text{Man-1}}$, H-4$_{\text{Man-2}}$, H-6$_{\text{Man-2}}$, H-4$_{\text{Man-3}}$, H-5$_{\text{Man-3}}$), 3.43 (t, ^3J = 4.9 Hz, 2H, H-11). **^{13}C-NMR** (151 MHz, D_2O, 25 °C)**:** δ (ppm) = 143.5 (1C, C-2‘), 125.5 (1C, C-3‘), 102.4 (1C, C-1$_{\text{Man-2}}$), 99.7 (1C, C-1$_{\text{Man-1}}$), 99.4 (1C, C-1$_{\text{Man-3}}$), 78.6 (1C, C-3$_{\text{Man-1}}$), 73.3 (1C, C-5$_{\text{Man-2}}$), 72.7 (1C, C-5$_{\text{Man-3}}$), 71.3 (1C, C-5$_{\text{Man-1}}$), 70.6 (1C, C-3$_{\text{Man-3}}$), 70.3 (1C, C-3$_{\text{Man-2}}$), 70.0 (1C, C-2$_{\text{Man-2}}$), 69.9 (1C, C-2$_{\text{Man-3}}$), 69.7 (1C, C-6$_{\text{Man-2}}$), 69.6 (4C, C-4, C-5, C-7, C-8),

69.5 (1C, C-2$_{Man-1}$), 69.2 (1C, C-10), 68.8 (1C, C-2), 66.7 (2C, C-4$_{Man-2}$, C-4$_{Man-3}$), 65.5 (1C, C-4$_{Man-1}$), 65.0 (1C, C-6$_{Man-1}$), 60.9 (1C, C-6$_{Man-3}$), 60.0 (1C, C-1'), 50.1 (1C, C-11), 50.1 (1C, C-1). **HR-MS** (ESI$^+$)**:** $m/z_{cal.}$ = 809.3032 [M+Na]$^+$, $m/z_{exp.}$ = 809.3024 [M+Na]$^+$. $\boldsymbol{R_f}$ (RP) = 0.60 (MeCN/H_2O 1:4). **Optical rotation** (LM): $[\alpha]_D^{23}$ = +45.6 (MeOH). **IR** (ATR): (cm^{-1}) = 3327, 2921, 2107, 1592, 1349, 1025, 979, 807, 501.

The analytical data follows literature data.[6]

4.1.19 Synthesis of 2-Nitrobenzyl (1,35-Bis(4-(2,3,4,6-tetra-*O*-acetyl-α-D-mannopyranosyloxymethyl)-1*H*-1,2,3-triazol-1-yl)-18-(17-(4-(2,3,4,6-tetra-*O*-acetyl-α-D-mannopyranosyloxymethyl)-1*H*-1,2,3-triazol-1-yl))-5-oxo-2,9,12,15-tetraoxa-6-azaheptadec-1-yl)-13,23-dioxo-3,6,9,16,20,27,30,33-octaoxa-12,24-diazapentatriacontan-18-yl)carbamate

The title compound was synthesized according to Scheme S 21 following modified synthesis protocols by *Yamamoto et al.*[39] and *Krumb et al.*[26]

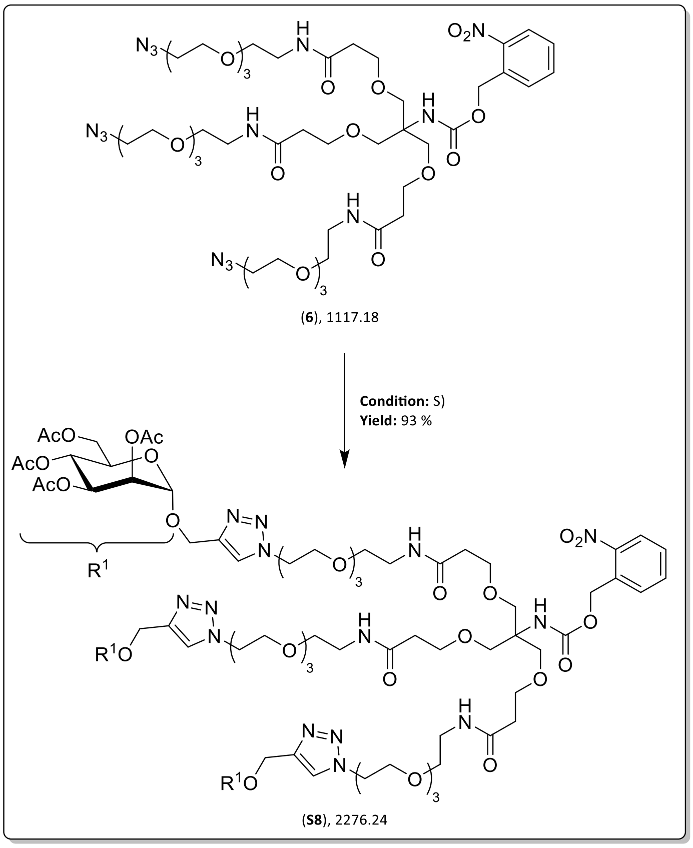

Scheme S 21: Synthesis of 2-Nitrobenzyl (1,35-Bis(4-(2,3,4,6-tetra-*O*-acetyl-α-D-mannopyranosyloxymethyl)-1*H*-1,2,3-triazol-1-yl)-18-(17-(4-(2,3,4,6-tetra-*O*-acetyl-α-D-mannopyranosyloxymethyl)-1*H*-1,2,3-triazol-1-yl))-5-oxo-2,9,12,15-tetraoxa-6-azaheptadec-1-yl)-13,23-dioxo-3,6,9,16,20,27,30,33-octaoxa-12,24-diazapentatriacontan-18-yl)carbamate (**S8**). Reaction conditions S) (**S7**), CuBr, PMDTA, DMF, Ar-atm., 45 °C, 4 h; yield: 93 %.

The reaction was carried out in a dried Schlenk vessel, which was previously equipped with a magnetic stirring bar. 2-Nitrobenzyl-(1,35-diazido-18-(17-azido-5-oxo-2,9,12,15-tetraoxa-6-azaheptadecyl)-13,23-dioxo-3,6,9,16,20,27,30,33-octaoxa-12,24-diazapentatria-contan-18-yl)-carbamate (**6**, 0.28 g, 0.25 mmol, 1.00 eq.) and Propargyl 2,3,4,6-Tetra-*O*-acetyl-α-D-mannopyranoside (**S7**, 0.34 g, 0.88 mmol, 3.50 eq.) were dissolved under an atmosphere of argon in absolute DMF (20.0 mL), which had previously been purged for half an hour with an argon gas stream in an ultrasonic bath. PMDTA (0.09 g, 0.10 mL, 0.50 mmol, 2.00 eq.) was added via syringe. The yellowish solution was degassed for

half an hour with an argon gas stream in an ultrasonic bath and then warmed to a temperature of 45 °C. $Cu^{I}Br$ (0.01 g, 0.06 mmol, 0.25 eq.) was added. The greenish reaction mixture was stirred at this temperature for four hours. Complete conversion of the limiting substrate and the formation of the desired product were determined by reaction control via TLC and HPLC-ELS-MS. All volatiles were removed in fine vacuum. Toluene (3 x 10.0 mL) was added to the residue and all volatiles were again removed in fine vacuum. The residue was taken up in EtOAc (1 x 50.0 mL), and the organic phase was washed with saturated NH_4Cl solution (3 x 50.0 mL). The collected aqueous phases were diluted with H_2O (1 x 50.0 mL) until all salts were dissolved and extracted with EtOAc (3 x 50.0 mL). The collected organic phases were dried over anhydrous Na_2SO_4 and filtered. All volatiles were removed in vacuo, and the residue was dried in fine vacuum. The crude product (0.64 g) was obtained in the form of yellowish oil and purified by RP-FCC (MeCN/H_2O, gradient from 10 % to 40 % to 100 % MeCN, Isolera One Purification System, SNAP C_{18} 60 g cartridge). The pure product (**S8**) was obtained in the form of colorless oil.

Yield: 0.53 g (0.23 mmol, 93 %), colorless oil.

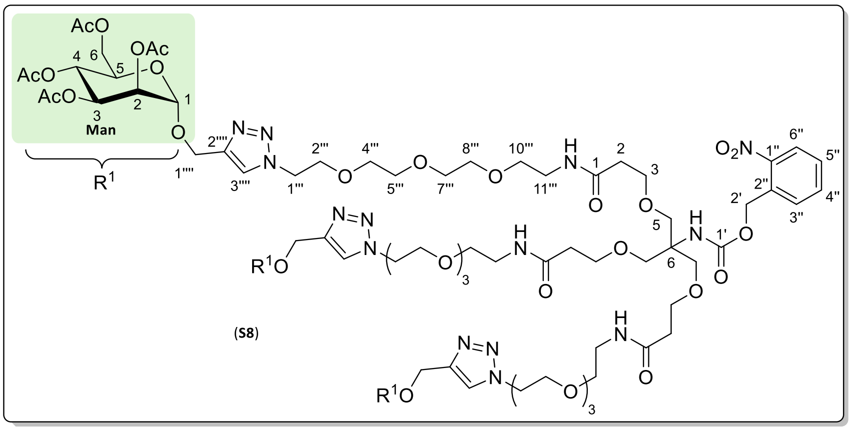

^{1}H-NMR (600 MHz, $CDCl_3$, 25 °C)**:** δ (ppm) = 8.09 – 8.07 (m, 1H, H-6‘‘), 7.76 (s, 3H, H-3‘‘‘‘), 7.68 – 7.63 (m, 2H, H-4‘‘, H-5‘‘), 7.48 – 7.44 (m, 1H, H-3‘‘), 6.73 (t, ^3J = 5.7 Hz, 3H, -N*H*-C-11‘‘‘), 5.74 (s, 1H, -N*H*-C-6), 5.43 (s, 2H, H-2‘), 5.31 – 5.28 (m, 6H, H-3$_{Man}$, H-4$_{Man}$), 5.22 (t$_{app}$, ^3J = 2.2 Hz, 3H, H-2$_{Man}$), 4.96 (d, ^3J = 1.7 Hz, 3H, H-1$_{Man}$), 4.82 (d, ^2J = 12.2 Hz, 3H, H-1a‘‘‘‘), 4.66 (d, ^2J = 12.2 Hz, 3H, H-1b‘‘‘‘), 4.55 (t, ^3J = 5.1 Hz, 6H, H-1‘‘‘), 4.29 (dd, ^2J = 12.2 Hz, ^3J = 5.0 Hz, 3H, H-6a$_{Man}$), 4.10 (dd, ^2J = 12.2 Hz, ^3J = 2.5 Hz, 3H, H-6b$_{Man}$), 4.08 – 4.05 (m, 3H, H-5$_{Man}$), 3.88 (t, ^3J = 5.1 Hz, 6H, H-2‘‘‘), 3.68 (t, ^3J = 5.8 Hz, 6H, H-2), 3.64 – 3.57 (m, 30H, H-4‘‘‘, H-5‘‘‘, H-7‘‘‘, H-8‘‘‘, H-5), 3.53 (t, ^3J = 5.4 Hz, 6H, H-10‘‘‘), 3.41 (q, ^3J = 5.4 Hz, 6H. H-11‘‘‘), 2.40 (t, ^3J = 5.8 Hz, 6H, H-3), 2.14 (s, 9H, C-2$_{Man}$-O(C=O)C*H*$_3$), 2.11 (s, 9H, C-6$_{Man}$-O(C=O)C*H*$_3$), 2.02 (s, 9H, C-3$_{Man}$-O(C=O)C*H*$_3$), 1.96 (s, 9H, C-4$_{Man}$-O(C=O)C*H*$_3$). **^{13}C-NMR** (151 MHz, $CDCl_3$, 25 °C)**:** δ (ppm) = 171.2 (3C, C-1), 170.7 (3C, C-6$_{Man}$-O(*C*=O)CH$_3$), 170.1 (3C, C-2$_{Man}$-O(*C*=O)CH$_3$), 169.9 (3C, C-3$_{Man}$-O(*C*=O)CH$_3$), 169.7 (3C, C-4$_{Man}$-O(*C*=O)CH$_3$), 154.6 (1C, C-1‘), 147.2 (1C, C-1‘‘), 143.3 (3C, C-2‘‘‘‘), 133.9 (1C, C-4‘), 133.3 (1C, C-2‘), 128.8 (1C, C-5‘), 128.5 (1C, C-3‘), 125.0 (1C, C-5‘), 124.2 (3C, C-3‘‘‘‘), 96.8 (3C, C-1$_{Man}$), 70.5, 70.4, 70.4, 70.1 (12C, C-4‘‘‘, C-5‘‘‘, C-7‘‘‘, C-8‘‘‘), 69.8 (3C, C-10‘‘‘), 69.4 (3C, C-5), 69.4 (3C, C-2‘‘‘), 69.3 (3C, C-2$_{Man}$), 69.0 (3C, C-4$_{Man}$), 68.6 (3C, C-5$_{Man}$), 67.4 (3C, C-2), 66.0 (3C, C-3$_{Man}$), 62.9 (1C, C-2‘), 62.3 (3C, C-6$_{Man}$), 60.9 (3C, C-1‘‘‘‘), 58.9 (1C, C-6), 50.3 (3C, C-1‘‘‘), 39.2 (3C, C-11‘‘‘), 36.6 (3C, C-3), 20.9 (3C, C-2$_{Man}$-O(C=O)*C*H$_3$), 20.8 (3C, C-6$_{Man}$-O(C=O)*C*H$_3$), 20.7 (3C, C-4$_{Man}$-O(C=O)*C*H$_3$), 20.7 (3C, C-3$_{Man}$-O(C=O)*C*H$_3$). **HR-MS** (ESI$^+$)**:** $m/z_{cal.}$ = 1138.4598 [M+2H]$^{2+}$, $m/z_{exp.}$ = 1138.4606 [M+2H]$^{2+}$; $m/z_{cal.}$ = 1138.9615 [M+2H]$^{2+}$, $m/z_{exp.}$ = 1138.9626 [M+2H]$^{2+}$; $m/z_{cal.}$ = 2298.8942 [M+Na]$^+$, $m/z_{exp.}$ = 2298.9003 [M+Na]$^+$; $m/z_{cal.}$ = 2298.8976 [M+Na]$^+$, $m/z_{exp.}$ = 2298.9020 [M+Na]$^+$. $\boldsymbol{R_f}$ (NP) = 0.25 (DCM/MeOH 15:1). **Optical rotation** (LM): $[\boldsymbol{\alpha}]_{\mathbf{D}}^{\mathbf{26}}$ = +30.6 ($CHCl_3$). **IR** (ATR): (cm^{-1}) = 3378, 2878, 2184, 2102, 1998, 1746, 1656, 1528, 1434, 1370, 1226, 1132, 1087, 1048, 979, 920, 734.

The analytical data follows literature data.[26]

4.1.20 Synthesis of *N*-(1-(4-(2,3,4,6-Tetra-*O*-acetyl-α-D-mannopyranosyloxymethyl)-1*H*-1,2,3-triazol-1-yl)-3,6,9-trioxaundec-11-yl)-6-amino-6-(17-(4-(2,3,4,6-tetra-*O*-acetyl-α-D-mannopyranosyloxymethyl)-1*H*-1,2,3-triazol-1-yl)-5-oxo-2,9,12,15-tetraoxa-6-azaheptadec-1-yl)-11-oxo-4,8,15,18,21-pentaoxa-12-aza-(23-(4-(2,3,4,6-tetra-*O*-acetyl-α-D-mannopyranosyloxymethyl)-1*H*-1,2,3-triazol-1-yl)-tricosanamide

The title compound was synthesized according to Scheme S 22 following modified synthesis protocols by *Amit et al.*[42, 43] and *Krumb et al.*[26]

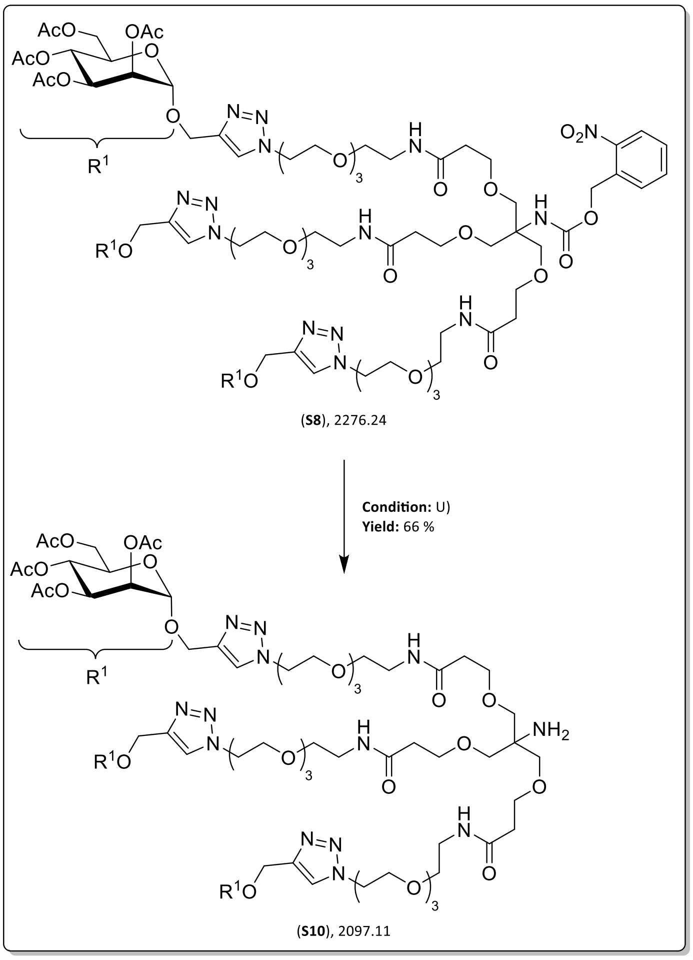

Scheme S 22: Synthesis of *N*-(1-(4-(2,3,4,6-Tetra-*O*-acetyl-α-D-mannopyranosyloxymethyl)-1*H*-1,2,3-triazol-1-yl)-3,6,9-trioxaundec-11-yl)-6-amino-6-(17-(4-(2,3,4,6-tetra-*O*-acetyl-α-D-mannopyranosyloxymethyl)-1*H*-1,2,3-triazol-1-yl)-5-oxo-2,9,12,15-tetraoxa-6-azaheptadec-1-yl)-11-oxo-4,8,15,18,21-pentaoxa-12-aza-(23-(4-(2,3,4,6-tetra-*O*-acetyl-α-D-mannopyranosyloxymethyl)-1*H*-1,2,3-triazol-1-yl)-tricosanamide (**S10**). Reaction conditions U) UV-A, MeCN, Ar-atm., rt, 15 h; yield: 70 %.

The reaction was carried out in a quartz tube which had previously been equipped with a magnetic stirring bar. 2-Nitrobenzyl (1,35-Bis(4-(2,3,4,6-tetra-*O*-acetyl-α-D-mannopyranosyloxymethyl)-1*H*-1,2,3-triazol-1-yl)-18-(17-(4-(2,3,4,6-tetra-*O*-acetyl-α-D-mannopyranosyloxymethyl)-1*H*-1,2,3-triazol-1-yl))-5-oxo-2,9,12,15-tetraoxa-6-azaheptadec-1-yl)-13,23-dioxo-3,6,9,16,20,27,-30,33-octaoxa-12,24-diazapentatriacontan-18-yl)carbamate (**S8**, 0.45 g, 0.20 mmol, 1.00 eq.) was dissolved under an atmosphere or argon in a mixture of MeCN and H_2O (1:1$_{v/v}$, 10.0 mL) that had previously been degassed for half an hour with an argon gas stream in an ultrasonic bath. The colorless solution was again degassed for half an hour with an argon gas stream in an ultrasonic bath. The reaction mixture was irradiated with

UV-A radiation (λ_{max} = 350 nm) with stirring at room temperature for 15 hours in a Rayonet photoreactor. Complete conversion of the limiting substrate and formation of the desired product were determined by TLC and HPLC-ELS-MS. The orange solution was diluted with MeCN (10.0 mL) and H_2O (10.0 mL) and transferred into a flask. All volatile components were removed by lyophilization. The crude product (0.34 g) was obtained in the form of an orange lyophilizate and purified by FCC (DCM/MeOH, isocratic at 10 % MeOH). The pure product (**S10**) was obtained in the form of orange oil.

Yield: 0.29 g (0.14 mmol, 70 %), orange oil.

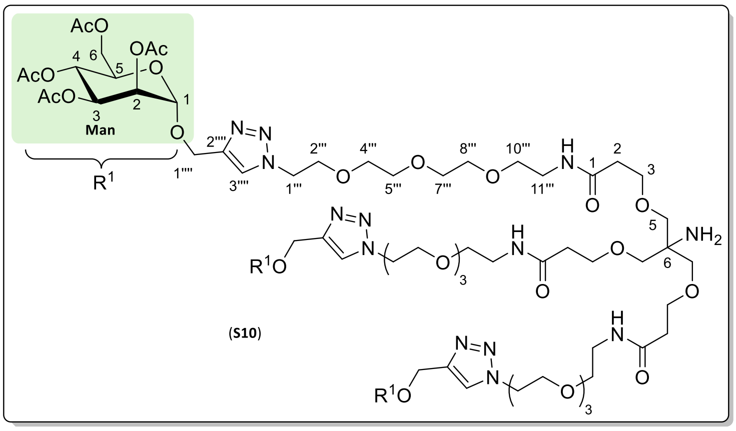

^{1}H-NMR (600 MHz, $CDCl_3$, 25 °C)**:** δ (ppm) = 7.77 (s, 3H, H-3‘‘‘), 7.17 (s, 2H, C-6-NH_2), 6.77 (t, ^3J = 5.7 Hz, 3H, -N*H*-C-11‘‘‘), 5.30 – 5.28 (m, 6H, H-3$_{Man}$, H-4$_{Man}$), 5.21 (dd, ^3J = 2.9, 1.7 Hz, 3H, H-2$_{Man}$), 4.95 (d, ^3J = 1.7 Hz, 3H, H-1$_{Man}$), 4.82 (d, ^2J = 12.2 Hz, 3H, H-1a‘‘‘‘), 4.66 (d, ^2J = 12.2 Hz, 3H, H-1b‘‘‘‘), 4.55 (t, ^3J = 5.3 Hz, 6H, H-1‘‘‘), 4.29 (dd, ^2J = 12.2 Hz, ^3J = 5.0 Hz, 3H, H-6a$_{Man}$), 4.10 (dd, ^2J = 12.2 Hz, ^3J = 2.4 Hz, 3H, H-6b$_{Man}$), 4.08 – 4.04 (m, 3H, H-5$_{Man}$), 3.88 (t, ^3J = 5.1 Hz, 6H, H-2‘‘‘), 3.70 (t, ^3J = 5.9 Hz, 6H, H-2), 3.65 – 3.55 (m, 12H, H-4‘‘‘, H-5‘‘‘, H-7‘‘‘, H-8‘‘‘), 3.53 (t, ^3J = 5.1 Hz, 6H, H-10‘‘‘), 3.43 (s, 6H. H-5), 3.42 – 3.39 (m, 6H, H-11‘‘‘), 2.41 (t, ^3J = 5.9 Hz, 6H, H-3), 2.14 (s, 9H, C-2$_{Man}$-O(C=O)C*H*$_3$), 2.11 (s, 9H, C-6$_{Man}$-O(C=O)C*H*$_3$), 2.02 (s, 9H, C-3$_{Man}$-O(C=O)C*H*$_3$), 1.96 (s, 9H, C-4$_{Man}$-O(C=O)C*H*$_3$). **^{13}C-NMR** (151 MHz, $CDCl_3$, 25 °C)**:** δ (ppm) = 171.5 (3C, C-1), 170.8 (3C, C-6$_{Man}$-O(*C*=O)CH_3), 170.1 (3C, C-2$_{Man}$-O(*C*=O)CH_3), 170.0 (3C, C-3$_{Man}$-O(*C*=O)CH_3), 169.7 (3C, C-4$_{Man}$-O(*C*=O)CH_3), 143.2 (3C, C-2‘‘‘‘), 124.3 (3C, C-3‘‘‘‘), 96.8 (3C, C-1$_{Man}$), 70.4, 70.4, 70.3, 70.1 (12C, C-4‘‘‘, C-5‘‘‘, C-7‘‘‘, C-8‘‘‘), 69.9 (3C, C-5), 69.7 (3C, C-10‘‘‘), 69.4 (3C, C-2‘‘‘), 69.4 (3C, C-2$_{Man}$), 69.1 (3C, C-4$_{Man}$), 68.6 (3C, C-5$_{Man}$), 67.5 (3C, C-2), 66.0 (3C, C-3$_{Man}$), 62.3 (3C, C-6$_{Man}$), 60.8 (3C, C-1‘‘‘‘), 58.7 (3C, C-6), 50.3 (3C, C-1‘‘‘), 39.1 (3C, C-11‘‘‘), 36.5 (3C, C-3), 20.9 (3C, C-2$_{Man}$-O(C=O)*C*H_3), 20.8 (3C, C-6$_{Man}$-O(C=O)*C*H_3), 20.7 (3C, C-4$_{Man}$-O(C=O)*C*H_3), 20.7 (3C, C-3$_{Man}$-O(C=O)*C*H_3). **HR-MS** (ESI$^+$)**:** $m/z_{cal.}$ = 1048.9489 $[M+2H]^{2+}$, $m/z_{exp.}$ = 1048.9513 $[M+2H]^{2+}$; $m/z_{cal.}$ = 1049.4505 $[M+2H]^{2+}$, $m/z_{exp.}$ = 1049.4531 $[M+2H]^{2+}$. ***R_f*** (NP) = 0.41 (DCM/MeOH 10:1). **Optical rotation** (LM): $[\alpha]_D^{26}$ = +30.6 ($CHCl_3$). **IR** (ATR)**:** (cm^{-1}) = 3384, 2874, 2175, 2106, 2017, 1745, 1650, 1547, 1435, 1370, 1224, 1131, 1085, 1048, 980, 913, 796, 728, 601, 502, 466, 440, 421.

The analytical data follows literature data.[26]

4.1.21 Synthesis of *N*-(1-Bis(17-(4-(2,3,4,6-tetra-*O*-acetyl-α-D-mannopyranosyloxy-methyl)-1*H*-1,2,3-triazol-1-yl)-5-oxo-2,9,12,15-tetraoxa-6-azaheptadec-1-yl)-18-(4-(2,3,4,6-tetra-*O*-acetyl-α-D-mannopyranosyloxymethyl)-1*H*-1,2,3-triazol-1-yl)-6-oxo-3,10,13,16-tetraoxa-7-azaoctadec-1-yl)-12-azido-4,7,10-pentaoxado-decanamide

The title compound was synthesized according to Scheme S 23 following modified synthesis protocols by *Cheng et al.*[44] and *Krumb et al.*[26]

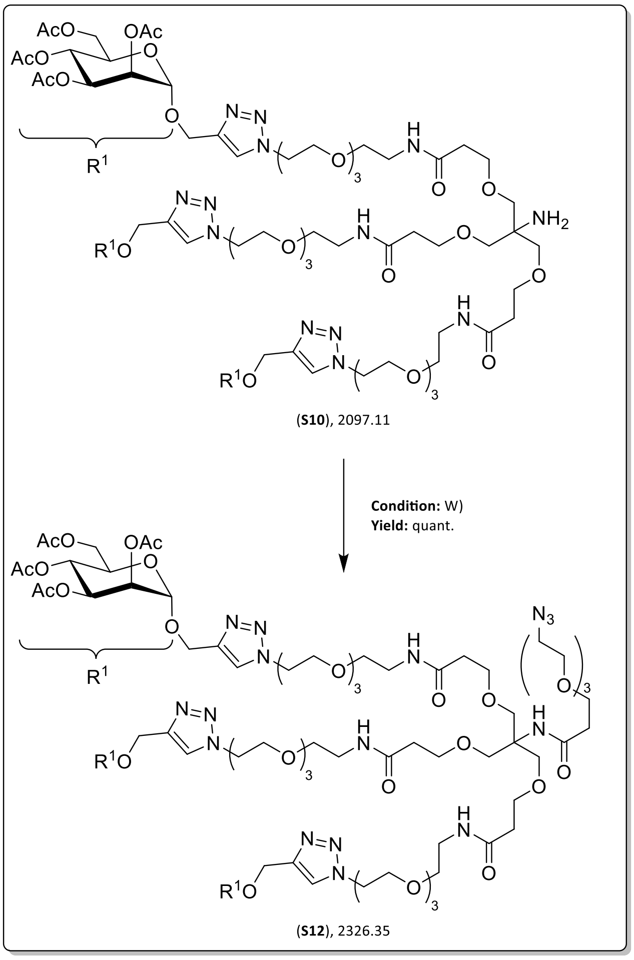

Scheme S 23: Synthesis of *N*-(1-Bis(17-(4-(2,3,4,6-tetra-*O*-acetyl-α-D-manno-pyranosyloxymethyl)-1*H*-1,2,3-triazol-1-yl)-5-oxo-2,9,12,15-tetraoxa-6-aza¬hepta¬dec-1-yl)-18-(4-(2,3,4,6-tetra-*O*-acetyl-α-D-mannopyranosyloxymethyl)-1H-1,2,3-tria-zol-1-yl)-6-oxo-3,10,13,16-tetraoxa-7-azaoctadec-1-yl)-12-azido-4,7,10-pentaoxadodecanamide (**S12**). Reaction conditions: A) (**3**), HATU, DIPEA, Ar-atm., DMF, rt, 48 h; yield: quant.

The reaction was carried out in a dried Schlenk vessel previously equipped with a magnetic stirring bar. 12-Azido-4,7,10-trioxadodecanoic acid (**3**, 1.20 g, 4.80 mmol, 10.0 eq.) was dissolved in absolute DMF (10.0 mL) under an atmosphere of argon. HATU (1.83 g, 4.80 mmol, 10.0 eq.) was added, followed by DIPEA (0.81 g, 1.10 mL, 6.24 mmol, 13.0 eq.) via syringe. The reaction mixture was stirred for half an hour at room temperature. *N*-(1-(4-(2,3,4,6-tetra-*O*-acetyl-α-D-mannopyranosyloxymethyl)-1*H*-1,2,3-triazol-1-yl)-3,6,9-trioxaundec-11-yl)-6-amino-6-(17-(4-(2,3,4,6-tetra-*O*-acetyl-α-D-manno-pyranosyl-oxymethyl)-1*H*-1,2,3-triazol-1-yl)-5-oxo-2,9,12,15-tetraoxa-6-azaheptadec-1-yl)-11-oxo-4,8,15,18,21-pentaoxa-12-aza-(23-(4-(2,3,4,6-tetra-*O*-acetyl-α-D-mannopyranosyloxy¬methyl)-1*H*-1,2,3-triazol-1-yl)-tricosanamide (**S10**, 1.00 g, 0.48 mmol, 1.00 eq.) was dissolved in absolute DMF (10.0 mL) under an atmosphere of argon and slowly added dropwise to the yellowish reaction mixture using a syringe. The mixture was stirred at room temperature for 48 hours. Complete conversion of the limiting substrate

and the formation of the desired product were determined by reaction control via TLC and HPLC-ELS-MS. All volatiles were removed in fine vacuum. Toluene (3 x 20.0 mL) was added to the residue and all volatiles were again removed in fine vacuum. The crude product (3.42 g) was obtained in the form of an orange oil and purified by FCC (DCM/MeOH, isocratic at 10 % MeOH) followed by RP-FCC (MeCN/H_2O, gradient from 10 % to 50 % to 100 % MeCN, Isolera One Purification System, SNAP C_{18} 60 g cartridge). The pure product (**S12**) was obtained in the form of yellowish oil.

Yield: 1.12 g (0.48 mmol, quant.), yellowish oil.

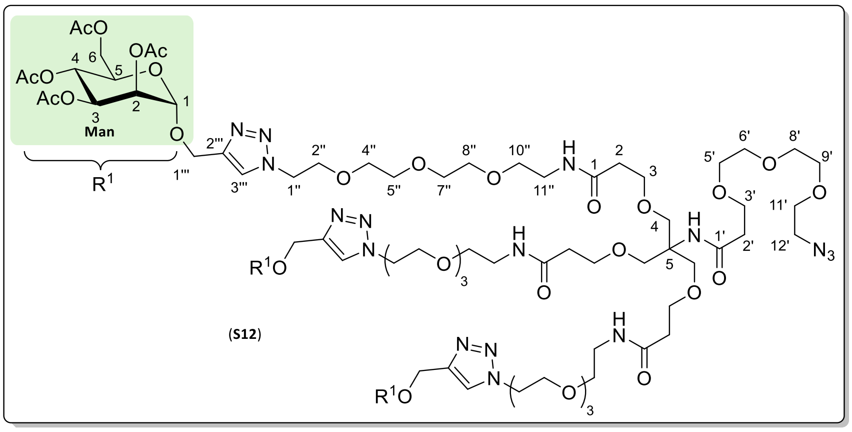

^{1}H-NMR (600 MHz, $CDCl_3$, 25 °C)**:** δ (ppm) = 7.77 (s, 3H, H-3‘‘‘), 6.76 (t, ^3J = 5.6 Hz, 3H, -N*H*-C-11‘‘), 6.58 (s, 1H, -N*H*-C-5), 5.33 – 5.26 (m, 6H, H-3$_{Man}$, H-4$_{Man}$), 5.22 (dd, ^3J = 2.4, 0.7 Hz, 3H, H-2$_{Man}$), 4.96 (d, ^3J = 1.7 Hz, 3H, H-1$_{Man}$), 4.83 (d, ^2J = 12.2 Hz, 3H, H-1a‘‘‘), 4.67 (d, ^2J = 12.2 Hz, 3H, H-1b‘‘‘), 4.56 (t, ^3J = 5.1 Hz, 6H, H-1‘‘), 4.30 (dd, ^2J = 12.2 Hz, ^3J = 4.9 Hz, 3H, H-6a$_{Man}$), 4.10 (dd, ^2J = 12.2 Hz, ^3J = 2.5 Hz, 3H, H-6b$_{Man}$), 4.09 – 4.05 (m, 3H, H-5$_{Man}$), 3.89 (t, ^3J = 5.1 Hz, 6H, H-2‘‘), 3.72 – 3.58 (m, 42H, H-4, H-3‘, H-5‘, H-6‘, H-8‘, H-9‘, H-11‘, H-4‘‘, H-5‘‘, H-7‘‘, H-8‘‘), 3.54 (t, ^3J = 5.5 Hz, 6H, H-10‘‘), 3.42 (q, ^3J = 5.5 Hz, 6H, H-11‘‘), 3.38 (t, ^3J = 5.0 Hz, 2H, H-12‘), 2.44 (t, ^3J = 6.1 Hz, 2H, H-2‘), 2.40 (t, ^3J = 5.9 Hz, 6H, H-3), 2.15 (s, 9H, C-2$_{Man}$-O(C=O)C*H*$_3$), 2.12 (s, 9H, C-6$_{Man}$-O(C=O)C*H*$_3$), 2.03 (s, 9H, C-4$_{Man}$-O(C=O)C*H*$_3$), 1.97 (s, 9H, C-3$_{Man}$-O(C=O)C*H*$_3$). **^{13}C-NMR** (151 MHz, $CDCl_3$, 25 °C)**:** δ (ppm) = 171.6 (1C, C-1‘), 171.3 (3C, C-1), 170.88 (3C, C-6$_{Man}$-O(*C*=O)CH$_3$), 170.18 (3C, C-2$_{Man}$-O(*C*=O)CH$_3$), 169.98 (3C, C-3$_{Man}$-O(*C*=O)CH$_3$), 169.78 (3C, C-4$_{Man}$-O(*C*=O)CH$_3$), 143.3 (3C, C-2‘‘‘), 124.2 (3C, C-3‘‘‘), 96.8 (3C, C-1$_{Man}$), 70.6, 70.5, 70.5, 70.5, 70.4, 70.2, 70.1, 70.0 (16C, C-5‘, C-6‘, C-8‘, C-9‘, C-4‘‘, C-5‘‘, C-7‘‘, C-8‘‘), 69.8 (6C, C-5, C-10‘‘), 69.4 (6C, C-2‘‘, C-2$_{Man}$), 69.2 (1C, C-11‘), 69.0 (3C, C-4$_{Man}$), 68.6 (3C, C-5$_{Man}$), 67.4 (3C, C-2), 67.3 (1C, C-3‘), 66.0 (3C, C-3$_{Man}$), 62.3 (3C, C-6$_{Man}$), 60.9 (3C, C-1‘‘‘), 59.7 (1C, C-5), 50.6 (1C, C-12‘), 50.3 (3C, C-1‘‘), 39.2 (3C, C-11‘‘), 37.4 (1C, C-2‘), 36.6 (3C, C-3), 20.9 (3C, C-2$_{Man}$-O(C=O)*C*H$_3$), 20.8 (3C, C-6$_{Man}$-O(C=O)*C*H$_3$), 20.7 (3C, C-4$_{Man}$-O(C=O)*C*H$_3$), 20.7 (3C, C-3$_{Man}$-O(C=O)*C*H$_3$). **HR-MS** (ESI$^+$)**:** $m/z_{cal.}$ = 797.9857 [M+3Na]$^{3+}$, $m/z_{exp.}$ = 797.9858 [M+3Na]$^{3+}$; $m/z_{cal.}$ = 798.3201 [M+3Na]$^{3+}$, $m/z_{exp.}$ = 798.3200 [M+Na]$^{3+}$; $m/z_{cal.}$ = 1185.4839 [M+2Na]$^{2+}$, $m/z_{exp.}$ = 1185.4848 [M+2Na]$^{2+}$; $m/z_{cal.}$ = 1185.9856 [M+2Na]$^{2+}$, $m/z_{exp.}$ = 1185.9840 [M+2Na]$^{2+}$. $\boldsymbol{R_f}$ (NP) = 0.23 (DCM/MeOH 15:1), 0.47 (DCM/MeOH 10:1). **Optical rotation** (LM): $[\boldsymbol{\alpha}]_{\mathbf{D}}^{\mathbf{27}}$ = +26.5 ($CHCl_3$).**IR** (ATR)**:** (cm^{-1}) = 3311, 2877, 2101, 1748, 1656, 1547, 1436, 1370, 1227, 1132, 1087, 1049, 980.

The analytical data follows literature data.[26]

4.1.22 Synthesis of *N*-(1-Bis(17-(4-(α-D-mannopyranosyloxymethyl)-1*H*-1,2,3-triazol-1-yl)-5-oxo-2,9,12,15-tetraoxa-6-azaheptadec-1-yl)-18-(4-(α-D-mannopyranosyloxymethyl)-1*H*-1,2,3-triazol-1-yl)-6-oxo-3,10,13,16-tetraoxa-7-azaoctadec-1-yl)-12-azido-4,7,10-pentaoxadodecanamide

The title compound was synthesized according to Scheme S 24 following modified synthesis protocols by *Percec et al.*[11] and *Krumb et al.*[26]

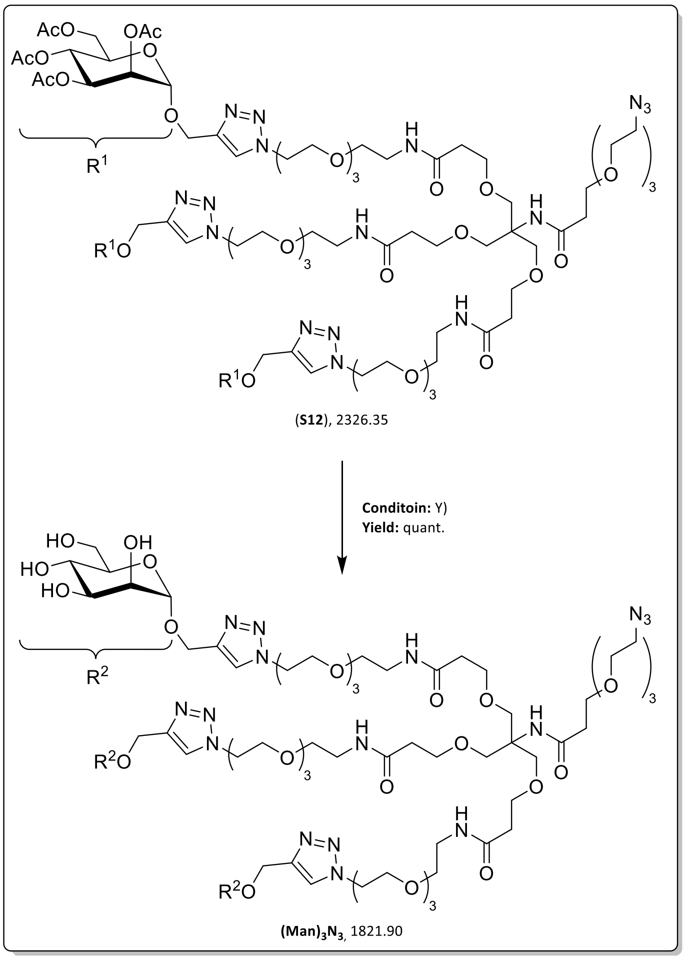

Scheme S 24: Synthesis of *N*-(1-Bis(17-(4-(α-D-mannopyranosyloxymethyl)-1*H*-1,2,3-triazol-1-yl)-5-oxo-2,9,12,15-tetraoxa-6-azaheptadec-1-yl)-18-(4-(α-D-mannopyranosyloxymethyl)-1*H*-1,2,3-triazol-1-yl)-6-oxo-3,10,13,16-tetraoxa-7-azaoctadec-1-yl)-12-azido-4,7,10-pentaoxadodecanamide **(Man)$_3$N$_3$**. Reaction conditions: Y) NaOMe, MeOH, Ar-atm., RT, 2 h; yield: quant.

The reaction was carried out in a dried Schlenk vessel, which was previously equipped with a magnetic stirring bar. *N*-(1-bis(17-(4-(2,3,4,6-tetra-*O*-acetyl-α-D-manno-pyranosyloxymethyl)-1*H*-1,2,3-triazol-1-yl)-5-oxo-2,9,12,15-tetraoxa-6-azaheptadec-1-yl)-18-(4-(2,3,4,6-tetra-*O*-acetyl-α-D-mannopyranosyloxymethyl)-1*H*-1,2,3-triazol-1-yl)-6-oxo-3,10,-13,16-tetra-oxa-7-azaoctadec-1-yl)-12-azido-4,7,10-penta¬oxa¬dodecan¬amide (**S12**, 0.10 g, 0.04 mmol, 1.00 eq.) was dissolved in absolute MeOH (10.0 mL) under an atmosphere of argon. NaOMe (2.60 mg, 0.05 mmol, 1.20 eq.) was added and the resulting solution was stirred for two hours at room temperature. Complete conversion of the limiting substrate and the formation of the desired product were determined by reaction control via TLC and HPLC-ELS-MS. The reaction was ended by addition of the ion exchange resin *Amberlite IR 120* (0.10 g), and the mixture was stirred for ten minutes until a constant pH value (pH = 6–7) was reached. The reaction mixture was filtered through a glass frit with *Celite* and eluted with MeOH (1 x 50.0 mL). All volatiles were removed in vacuo. The crude product (0.08 g) was obtained in the form of yellowish

oil and purified by RP-FCC (MeCN/H_2O, gradient from 5 % to 25 % to 100 % MeCN, Isolera One Flash Purification System, SNAP C_{18} 12 g cartridge). The pure product **$(Man)_3N_3$** was obtained in the form of colorless oil.

Yield: 0.07 g (0.04 mmol, quant.), colorless oil.

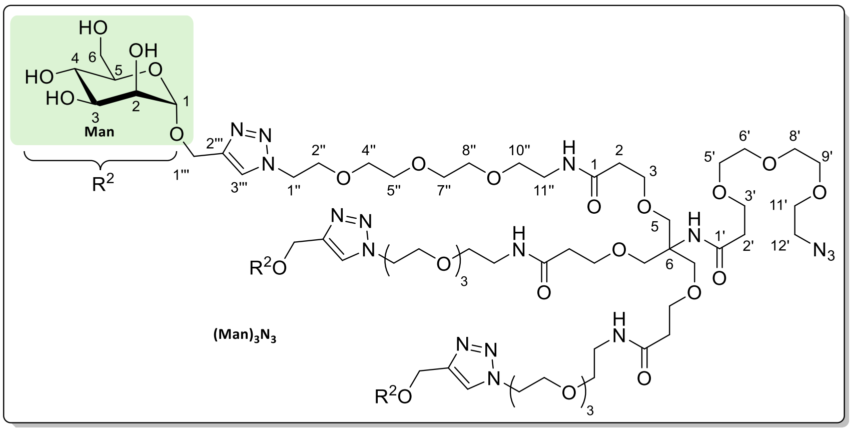

^{1}H-NMR (600 MHz, D_2O, 25 °C)**:** δ (ppm) = 8.07 (s, 3H, H-3‘‘‘), 4.92 (d, ^3J = 1.7 Hz, 3H, H-1$_{Man}$), 4.78 (d, ^2J = 12.4 Hz, 3H, H-1a‘‘‘), 4.66 (d, ^2J = 12.4 Hz, 3H, H-1a‘‘‘), 4.59 (t, ^3J = 5.0 Hz, 6H, H-1‘‘), 3.93 (t, ^3J = 5.0 Hz, 6H, H-2‘‘), 3.87 (dd, ^3J = 3.5, 1.7 Hz, 3H, H-2$_{Man}$), 3.80 (dd, ^2J = 12.2 Hz, ^3J = 2.0 Hz, 3H, H-6a$_{Man}$), 3.75 – 3.69 (m, 3H, H-3$_{Man}$), 3.69 – 3.53 (m, 66H, H-4$_{Man}$, H-5$_{Man}$, H-6b$_{Man}$, H-2, H-5, H-3‘, H-5‘, H-6‘, H-8‘, H-11‘, H-9‘, H-4‘‘, H-5‘‘, H-7‘‘, H-8‘‘, H-10‘‘), 3.44 (t, ^3J = 4.9 Hz, 2H, H-12‘), 3.34 (t, ^3J = 5.5 Hz, 6H, H-11‘‘), 2.46 (t, ^3J = 6.1 Hz, 2H, H-2‘), 2.44 (t, ^3J = 6.1 Hz, 6H, H-3). **^{13}C-NMR** (151 MHz, D_2O, 25 °C)**:** δ (ppm) = 174.1 (3C, C-1), 173.8 (1C, C-1‘), 143.5 (3C, C-2‘‘‘), 125.6 (3C, C-3‘‘‘), 99.4 (3C, C-1$_{Man}$), 73.0 (3C, C-5$_{Man}$), 70.5 (3C, C-3$_{Man}$), 69.9 (3C, C-2$_{Man}$), 69.6, 69.6, 69.6, 69.6, 69.5, 69.5 (16C, C-4‘‘, C-5‘, C-6‘, C-8‘, C-9‘, C-5‘‘, C-7‘‘, C-8‘‘), 69.4 (3C, C-5), 69.3, (1C, C-11‘) 68.8 (3C, C-10‘‘), 68.7 (3C, C-2‘‘), 68.4 (1C, C-3‘), 67.5 (3C, C-2), 66.6 (3C, C-4$_{Man}$), 60.8 (3C, C-6$_{Man}$), 60.3 (1C, C-6), 59.7 (3C, C-1‘‘‘), 50.1 (1C, C-12‘), 50.0 (3C, C-1‘‘), 39.0 (3C, C-11‘‘), 36.4 (1C, C-2‘), 36.1 (3C, C-3). **HR-MS** (ESI$^+$)**:** $m/z_{cal.}$ = 629.9434 $[M+3Na]^{3+}$, $m/z_{exp.}$ = 629.9433 $[M+3Na]^{3+}$; $m/z_{cal.}$ = 630.2779 $[M+3Na]^{3+}$, $m/z_{exp.}$ = 630.2773 $[M+3Na]^{3+}$; $m/z_{cal.}$ = 933.4205 $[M+2Na]^{2+}$, $m/z_{exp.}$ = 933.4188 $[M+3Na]^{3+}$; $m/z_{cal.}$ = 933.9222 $[M+2Na]^{2+}$, $m/z_{exp.}$ = 933.9205 $[M+3Na]^{3+}$. ***R_f*** (NP) = 0.17 (DCM/MeOH + AcOH 5:1 +0.1 %$_{v/v}$ additive); (RP) = 0.43 (MeCN/H_2O 3:7). **Optical rotation** (LM)**:** $[\alpha]_D^{22}$ = +34.0 (MeOH). **IR** (ATR)**:** (cm^{-1}) = 3628, 3348, 2915, 2875, 2114, 1652, 1596, 1457, 1352, 1305, 1249, 1099, 913, 846, 773, 741, 692, 679, 667, 639, 607, 561, 538, 516, 500, 482, 473, 457, 432, 415.

The analytical data follows literature data.[26]

4.1.23 Synthesis of 2-Nitrobenzyl (1,35-Bis(4-(2,4-di-*O*-benzoyl-3,6-di-*O*-(2,3,4,6-tetra-*O*-acetyl-α-D-mannopyranosyl)-α-D-mannopyranosyloxymethyl)-1*H*-1,2,3-triazol-1-yl)-18-(17-(4-(2,4-di-*O*-benzoyl-3,6-di-*O*-(2,3,4,6-tetra-*O*-acetyl-α-D-mannopyranosyl)-α-D-mannopyranosyloxymethyl)-1*H*-1,2,3-triazol-1-yl))-5-oxo-2,9,12,15-tetraoxa-6-azaheptadec-1-yl)-13,23-dioxo-3,6,9,16,20,-27,30,33-octaoxa-12,24-diazapentatriacontan-18-yl)carbamate

The title compound was synthesized according to Scheme S 25 following modified synthesis protocols by *Krumb et al.*[26] and *Yamamoto et al.*[39]

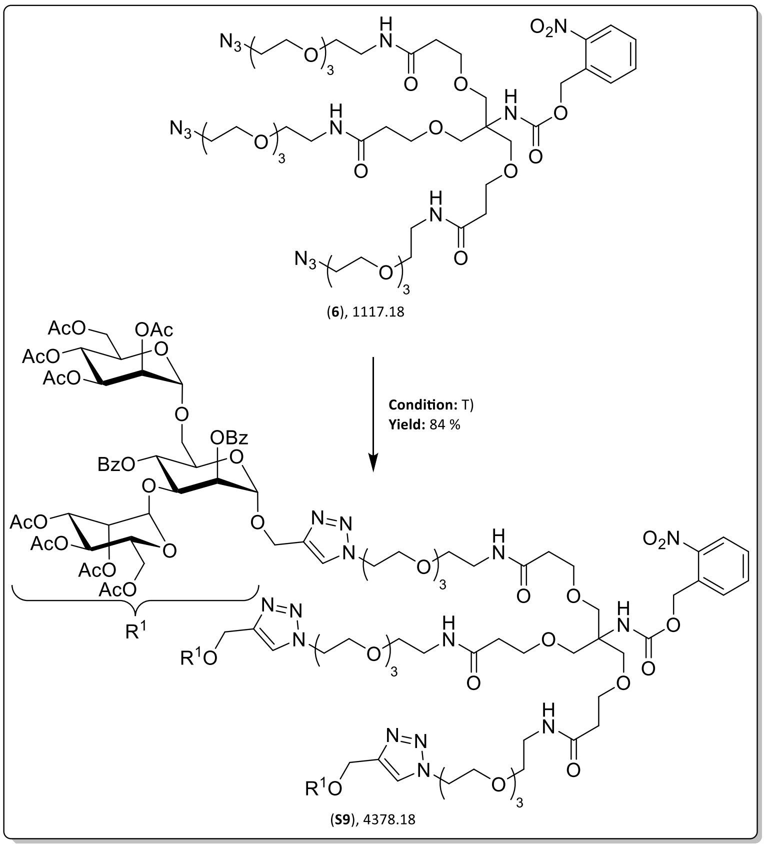

Scheme S 25: Synthesis of 2-Nitrobenzyl (1,35-Bis(4-(2,4-di-*O*-benzoyl-3,6-di-*O*-(2,3,4,6-tetra-*O*-acetyl-α-D-mannopyranosyl)-α-D-mannopyranosyloxymethyl)-1*H*-1,2,3-triazol-1-yl)-18-(17-(4-(2,4-di-*O*-benzoyl-3,6-di-*O*-(2,3,4,6-tetra-*O*-acetyl-α-DD-mannopyranosyl)-α-D-mannopyranosyloxymethyl)-1*H*-1,2,3-triazol-1-yl))-5-oxo-2,9,12,15-tetraoxa-6-azaheptadec-1-yl)-13,23-dioxo-3,6,9,16,20,27,30,33-octaoxa-12,24-diazapentatriacontan-18-yl)carbamate (**6**). Reaction condition: T) (**1**), $Cu^{I}Br$, PMDTA, DMF, Ar-Atm., 45 °C, 5 h; yield: 84 %.

The reaction was carried out in a dried Schlenk vessel, which was previously equipped with a magnetic stirring bar. 2-Nitrobenzyl-(1,35-diazido-18-(17-azido-5-oxo-2,9,12,15-tetraoxa-6-azaheptadecyl)-13,23-dioxo-3,6,9,16,20,27,30,33-octaoxa-12,24-diazapentatria-contan-18-yl)carbamate (**6**, 0.20 g, 0.18 mmol, 1.00 eq.) and Propargyl 2,4-di-*O*-benzoyl-3,6-di-*O*-(2,3,4,6-tetra-*O*-acetyl-α-D-manno¬pyranosyl)-α-D-manno¬pyranosyloxymethyl (**1**, 0.68 g, 0.63 mmol, 3.50 eq.) were dissolved under an atmosphere of argon in absolute DMF (10.0 mL), which had previously been degassed for half an hour with an argon gas stream in an ultrasonic bath. PMDTA (0.06 g, 0.08 mL, 0.36 mmol, 2.00 eq.) was added via syringe. The yellowish solution was again degassed for half an hour with an argon gas stream in an ultrasonic bath and then warmed to a temperature of 45 °C. $Cu^{I}Br$ (7.20 mg, 0.05 mmol, 0.25 eq.) was added. The greenish reaction mixture was stirred at this temperature for five hours. Complete conversion of the limiting substrate and the formation of the desired product were determined by reaction control via TLC and HPLC-ELS-MS. All volatile components were removed in fine vacuum. Toluene (3 x 10.0 mL) was added to the residue and all volatiles were again removed in fine vacuum.

The residue was taken up in EtOAc (1 x 50.0 mL), and the organic phase was washed with saturated NH_4Cl solution (3 x 50.0 mL). The collected aqueous phases were diluted with H_2O (1 x 50.0 mL) until all salts were dissolved and extracted with EtOAc (3 x 50.0 mL). The collected organic phases were dried over anhydrous Na_2SO_4 and filtered. All volatiles were removed under vacuum, and the residue was dried in fine vacuum. The crude product (0.62 g) was obtained in the form of yellowish oil and purified by FCC (DCM/MeOH, isocratic at 5 % MeOH). The pure product (**S9**) was obtained in the form of colorless oil.

Yield: 0.66 g (0.15 mmol, 84 %), colorless oil.

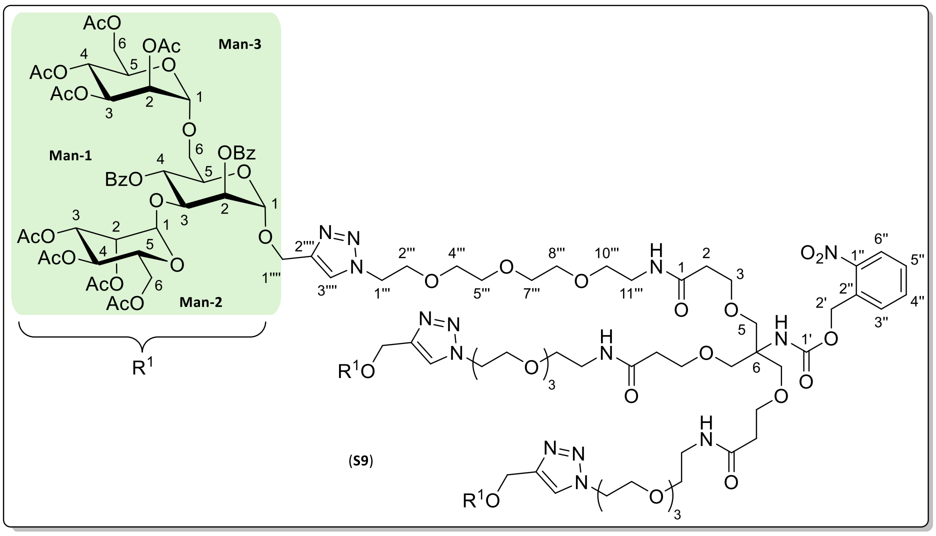

^{1}**H-NMR** (600 MHz, $CDCl_3$, 25 °C): *δ* (ppm) = 8.16 – 8.12 (m, 6H, C-2$_{\text{Man-1}}$-O(C=O)Ph, H$_{ortho}$), 8.10 – 8.07 (m, 1H, H-6‘‘), 8.05 – 8.00 (m, 6H, C-4$_{\text{Man-1}}$-O(C=O)Ph, H$_{ortho}$), 7.83 (s, 3H, H-3‘‘‘‘), 7.68 – 7.53 (m, 14H, H-4‘‘, H-5‘‘, C-2$_{\text{Man-1}}$-O(C=O)Ph, H$_{meta}$, H$_{para}$, C-4$_{\text{Man-1}}$-O(C=O)Ph, H$_{para}$), 7.51 – 7.46 (m, 1H, H-3‘‘), 7.46 – 7.42 (m, 6H, C-4$_{\text{Man-1}}$-O(C=O)Ph, H$_{meta}$), 6.70 (t, ^3J = 5.5 Hz, 3H, -N*H*-C-11‘‘‘), 5.74 (s, 1H, -N*H*-C-6), 5.70 (t$_{app}$, ^3J = 10.0 Hz, 3H, H-4$_{\text{Man-1}}$), 5.51 (dd, ^3J = 3.5, 1.8 Hz, 3H, H-2$_{\text{Man-1}}$), 5.44 (s, 2H, H-2‘), 5.34 (dd, ^3J = 10.1, 3.4 Hz, 3H, H-3$_{\text{Man-3}}$), 5.31 – 5.27 (m, 3H, H-2$_{\text{Man-3}}$), 5.24 (t, ^3J = 10.1 Hz, 3H, H-4$_{\text{Man-3}}$), 5.16 (d, ^3J = 1.7 Hz, 3H, H-1$_{\text{Man-1}}$), 5.10 – 5.04 (m, 6H, H-3$_{\text{Man-2}}$, H-4$_{\text{Man-2}}$), 4.94 (d, ^3J = 1.9 Hz, 3H, H-1$_{\text{Man-2}}$), 4.88 (d, ^2J = 12.1 Hz, 3H, H-1a‘‘‘‘), 4.85 (d, ^3J = 1.8 Hz, 3H, H-1$_{\text{Man-3}}$), 4.84 (t$_{app}$, ^3J = 2.4 Hz, 3H, H-2$_{\text{Man-2}}$), 4.72 (d, ^2J = 12.1 Hz, 3H, H-1b‘‘‘‘), 4.57 (t, ^3J = 5.3 Hz, 6H, H-1‘‘‘), 4.45 (dd, ^3J = 9.8, 3.4 Hz, 3H, H-3$_{\text{Man-1}}$), 4.25 (ddd, ^3J = 10.3, 6.0, 2.2 Hz, 3H, H-5$_{\text{Man-1}}$), 4.16 – 4.11 (m, 6H, H-6a$_{\text{Man-2}}$, H-6a$_{\text{Man-3}}$), 4.07 – 4.02 (m, 6H, H-5$_{\text{Man-2}}$, H-5$_{\text{Man-3}}$), 3.98 – 3.90 (m, 15H, H-5a, H-2a‘‘‘, H-6a$_{\text{Man-1}}$, H-6b$_{\text{Man-2}}$, H-6b$_{\text{Man-3}}$), 3.70 (t, ^3J = 5.9 Hz, 6H, H-2), 3.66 – 3.56 (m, 33H, H-5b, H-2b‘‘‘, H-4‘‘‘, H-5‘‘‘, H-7‘‘‘, H-8‘‘‘, H-6b$_{\text{Man-1}}$), 3.53 (t, ^3J = 5.4 Hz, 6H, H-10‘‘‘), 3.43 (q, ^3J = 5.5 Hz, 6H, H-11‘‘‘), 2.42 (t, ^3J = 5.9 Hz, 6H, H-3), 2.11 (s, 9H, C-4$_{\text{Man-3}}$-O(C=O)C*H*$_3$), 2.04 (s, 9H, C-6$_{\text{Man-3}}$-O(C=O)C*H*$_3$), 2.03 (s, 9H, C-3$_{\text{Man-3}}$-O(C=O)C*H*$_3$), 1.96 (s, 9H, C-6$_{\text{Man-2}}$-O(C=O)C*H*$_3$), 1.92 (s, 9H, C-3$_{\text{Man-2}}$-O(C=O)C*H*$_3$), 1.91 (s, 9H, C-4$_{\text{Man-1}}$-O(C=O)C*H*$_3$), 1.84 (s, 9H, C-2$_{\text{Man-2}}$-O(C=O)C*H*$_3$), 1.81 (s, 9H, C-2$_{\text{Man-3}}$-O(C=O)C*H*$_3$). ^{13}**C-NMR** (151 MHz, $CDCl_3$, 25 °C): *δ* (ppm) = 171.2 (3C, C-1), 170.8 (3C, C-6$_{\text{Man-2}}$-O(*C*=O)CH$_3$), 170.6 (3C, C-6$_{\text{Man-3}}$-O(*C*=O)CH$_3$), 170.0 (3C, C-4$_{\text{Man-3}}$-O(*C*=O)CH$_3$), 169.9 (3C, C-3$_{\text{Man-3}}$-O(*C*=O)CH$_3$), 169.8 (3C, C-3$_{\text{Man-2}}$-O(*C*=O)CH$_3$), 169.6 (3C, C-4$_{\text{Man-2}}$-O(*C*=O)CH$_3$), 169.2 (3C, C-2$_{\text{Man-3}}$-O(*C*=O)CH$_3$), 169.1 (3C, C-2$_{\text{Man-2}}$-O(*C*=O)CH$_3$), 165.9 (3C, C-2$_{\text{Man-1}}$-O(*C*=O)Ph), 165.2 (3C, C-4$_{\text{Man-1}}$-O(*C*=O)Ph), 154.6 (1C, C-1‘), 147.2 (1C, C-1‘‘), 142.9 (3C, C-2‘‘‘‘), 133.9 (1C, C-4‘‘), 133.7 (3C, C-2$_{\text{Man-1}}$-O(C=O)Ph, C$_{para}$), 133.6 (3C, C-4$_{\text{Man-1}}$-O(C=O)Ph, C$_{para}$), 133.3 (1C, C-2‘‘), 133.0 (6C, C-2$_{\text{Man-1}}$-O(C=O)Ph, C$_{ortho}$), 129.9 (6C, C-4$_{\text{Man-1}}$-O(C=O)Ph, C$_{ortho}$), 129.0 (3C, C-2$_{\text{Man-1}}$-O(C=O)Ph, C$_{ipso}$), 128.8 (6C,

C-2$_{\text{Man-1}}$-O(C=O)Ph, C$_{meta}$), 128.8 (1C, C-5‘‘), 128.7 (3C, C-4$_{\text{Man-1}}$-O(C=O)Ph, C$_{ipso}$), 128.5 (1C, C-2‘‘), 128.5 (6C, C-4$_{\text{Man-1}}$-O(C=O)Ph, C$_{meta}$), 125.0 (1C, C-6‘‘), 124.4 (3C, C-3‘‘‘‘), 99.4 (3C, C-1$_{\text{Man-2}}$), 97.3 (3C, C-1$_{\text{Man-3}}$), 96.6 (3C, C-1$_{\text{Man-1}}$), 75.5 (3C, C-3$_{\text{Man-1}}$), 71.6 (3C, C-2$_{\text{Man-1}}$), 70.5, 70.5, 70.4, 70.1 (12C, C-4‘‘‘, C-5‘‘‘, C-7‘‘‘, C-8‘‘‘), 69.8 (3C, C-10‘‘‘), 69.5 (3C, C-5$_{\text{Man-1}}$), 69.4 (3C, C-5), 69.3 (3C, C-2‘‘‘), 69.2 (3C, C-2$_{\text{Man-2}}$), 69.2 (3C, C-2$_{\text{Man-3}}$), 69.2 (3C, C-3$_{\text{Man-3}}$), 68.6 (3C, C-5$_{\text{Man-2}}$), 68.5 (3C, C-4$_{\text{Man-1}}$), 68.2 (3C, C-3$_{\text{Man-2}}$), 67.4 (3C, C-2), 66.4 (3C, C-6$_{\text{Man-1}}$), 65.9 (3C, C-4$_{\text{Man-2}}$), 65.7 (3C, C-4$_{\text{Man-3}}$), 62.9 (1C, C-2‘), 62.3 (3C, C-6$_{\text{Man-2}}$), 62.2 (3C, C-6$_{\text{Man-3}}$), 60.4 (3C, C-1‘‘‘‘), 58.9 (3C, C-6), 50.2 (3C, C-1‘‘‘), 39.2 (3C, C-11‘‘‘), 36.6 (3C, C-3), 20.9 (3C, C-4$_{\text{Man-3}}$-O(C=O)CH$_3$), 20.8 (3C, C-6$_{\text{Man-3}}$-O(C=O)CH$_3$), 20.7 (3C, C-3$_{\text{Man-3}}$-O(C=O)CH$_3$), 20.7 (3C, C-6$_{\text{Man-2}}$-O(C=O)CH$_3$), 20.7 (3C, C-3$_{\text{Man-2}}$-O(C=O)CH$_3$), 20.6 (3C, C-4$_{\text{Man-2}}$-O(C=O)CH$_3$), 20.5 (3C, C-2$_{\text{Man-2}}$-O(C=O)CH$_3$), 20.5 (3C, C-2$_{\text{Man-3}}$-O(C=O)CH$_3$). **HR-MS** (ESI$^+$)**:** $m/z_{\text{cal.}}$ = 1459.8437 $[M+3H]^{3+}$, $m/z_{\text{exp.}}$ = 1459.8437 $[M+3H]^{3+}$; $m/z_{\text{cal.}}$ = 1460.1782 $[M+3H]^{3+}$, $m/z_{\text{exp.}}$ = 1460.1718 $[M+3H]^{3+}$; $m/z_{\text{cal.}}$ = 2189.2620 $[M+2H]^{2+}$, $m/z_{\text{exp.}}$ = 2189.2609 $[M+2H]^{2+}$; $m/z_{\text{cal.}}$ = 2189.7636 $[M+2H]^{2+}$, $m/z_{\text{exp.}}$ = 2189.7631 $[M+2H]^{2+}$. $\boldsymbol{R_f}$ (NP) = 0.23 (EtOAc/MeOH 5:1), 0.43 (DCM/MeOH 10:1). **Optical rotation** (LM)**:** $[\boldsymbol{\alpha}]_{\mathbf{D}}^{\mathbf{22}}$ = +9.50 ($CHCl_3$). **IR** (ATR)**:** (cm^{-1}) = 2925, 2869, 1749, 1667, 1529, 1452, 1370, 1226, 1047, 981, 753, 714.

4.1.24 Synthesis of *N*-(1-(4-(2,4-Di-*O*-benzoyl-3,6-di-*O*-(2,3,4,6-tetra-*O*-acetyl-α-D-mannopyranosyl)-α-D-mannopyranosyloxymethyl)-1*H*-1,2,3-triazol-1-yl)-3,6,9-trioxaundec-11-yl)-6-amino-6-(17-(4-(2,4-di-*O*-benzoyl-3,6-di-*O*-(2,3,4,6-tetra-*O*-acetyl-α-D-mannopyranosyl)-α-D-mannopyranosyloxymethyl)-1*H*-1,2,3-triazol-1-yl)-5-oxo-2,9,12,15-tetraoxa-6-azaheptadec-1-yl)-11-oxo-4,8,15,18,-21-pentaoxa-12-aza-(23-(4-(2,4-di-*O*-benzoyl-3,6-di-*O*-(2,3,4,6-tetra-*O*-acetyl-α-D-mannopyranosyl)-α-D-mannopyranosyloxymethyl)-1*H*-1,2,3-triazol-1-yl)-tricosanamide

The title compound was synthesized according to Scheme S 26 following modified synthesis protocols by *Amit et al.*[42, 43] and *Krumb et al.*[26]

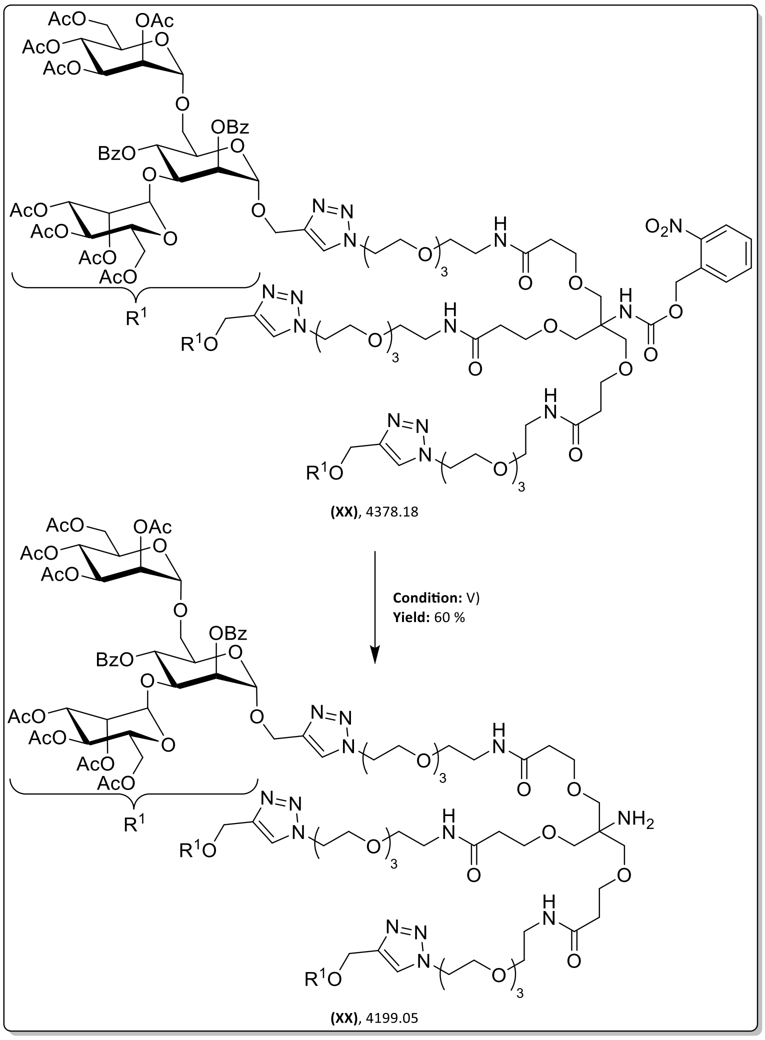

Scheme S 26: Synthesis of *N*-(1-(4-(2,4-Di-*O*-benzoyl-3,6-di-*O*-(2,3,4,6-tetra-*O*-acetyl-α-D-mannopyranosyl)-α-D-mannopyranosyloxymethyl)-1*H*-1,2,3-triazol-1-yl)-3,6,9-trioxaundec-11-yl)-6-amino-6-(17-(4-(2,4-di-*O*-benzoyl-3,6-di-*O*-(2,3,4,6-tetra-*O*-acetyl-α-D-mannopyranosyl)-α-D-mannopyranosyloxymethyl)-1*H*-1,2,3-triazol-1-yl)-5-oxo-2,9,12,15-tetraoxa-6-azaheptadec-1-yl)-11-oxo-4,8,15,18,21-pentaoxa-12-aza-(23-(4-(2,4-di-*O*-benzoyl-3,6-di-*O*-(2,3,4,6-tetra-*O*-acetyl-α-D-mannopyranosyl)-α-D-mannopyranosyloxymethyl)-1*H*-1,2,3-triazol-1-yl)tricosanamide (**S11**). Reaction conditions: V) UV-A, MeCN, Ar-atm., rt, 15 h; yield: 60 %.

The reaction was carried out in a quartz tube, which had previously been equipped with a magnetic stir bar. 2-Nitrobenzyl (1,35-Bis(4-(2,4-di-*O*-benzoyl-3,6-di-*O*-(2,3,4,6-tetra-*O*-acetyl-α-D-mannopyranos-yl)-α-D-mannopyranosyloxymethyl)-1*H*-1,2,3-triazol-1-yl)-18-(17-(4-(2,4-di-*O*-benzoyl-3,6-di-*O*-(2,3,4,6-tetra-O-acetyl-α-D-mannopyranosyl)-α-D-mannopyranosyloxymethyl)-1*H*-1,2,3-triazol-1-yl))-

5-oxo-2,9,12,15-tetraoxa-6-azaheptadec-1-yl)-13,23-dioxo-3,6,9,16,20,27,30,33-octaoxa-12,24-diaza-pentatriacontan-18-yl)¬carbamate (**S9**, 0.20 g, 0.05 mmol, 1.00 eq.) was dissolved under an atmosphere of argon in a mixture of MeCN and H_2O (1:1$_{v/v}$, 5.00 mL) that had previously been degassed for half an hour with an argon gas stream in an ultrasonic bath. The colorless solution was again degassed for half an hour with an argon gas stream in an ultrasonic bath. The reaction mixture was irradiated with UV-A radiation (λ_{max} = 350 nm) for 15 hours in a Rayonet photoreactor with stirring at room temperature. Complete conversion of the limiting substrate and formation of the desired product were determined by reaction control via TLC and HPLC-ELS-MS. The orange solution was diluted with MeCN (10.0 mL) and H_2O (10.0 mL) and transferred to a flask. All volatile components were removed by lyophilization. The crude product (0.18 g) was obtained in the form of an orange lyophilizate and purified by FCC (DCM/MeOH, isocratic at 5 % MeOH). The pure product (**S11**) was obtained in the form of orange oil.
Yield: 0.13 g (0.03 mmol, 60 %), orange oil.

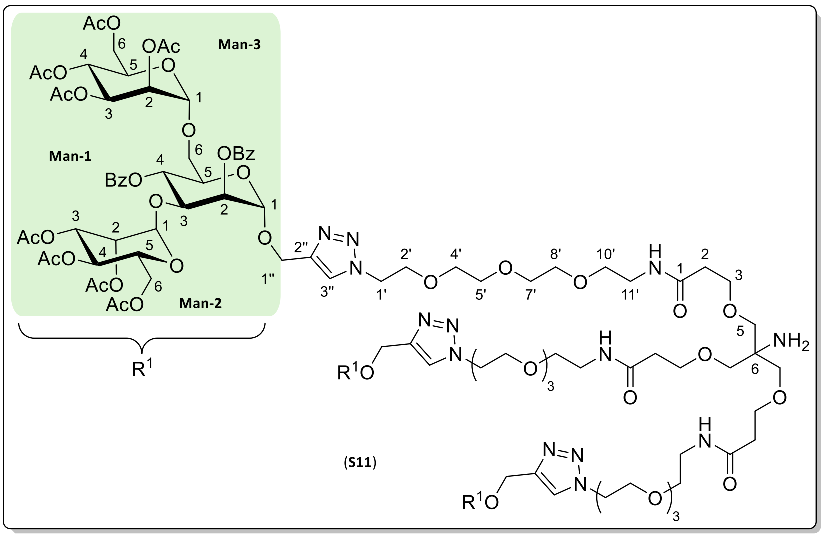

^{1}H-NMR (600 MHz, $CDCl_3$, 25 °C)**:** δ (ppm) = 8.19 – 8.10 (m, 6H, C-2$_{Man-1}$-O(C=O)Ph, H$_{ortho}$), 8.06 – 7.99 (m, 6H, C-4$_{Man-1}$-O(C=O)Ph, H$_{ortho}$), 7.83 (s, 3H, H-3‘‘), 7.64 – 7.60 (m, 3H, C-2$_{Man-1}$-O(C=O)Ph, H$_{para}$), 7.60 – 7.57 (m, 3H, C-4$_{Man-1}$-O(C=O)Ph, H$_{para}$), 7.56 – 7.53 (m, 6H, C-2$_{Man-1}$-O(C=O)Ph, H$_{meta}$), 7.46 – 7.42 (m, 6H, C-4$_{Man-1}$-O(C=O)Ph, H$_{meta}$), 5.70 (t, ^3J = 10.0 Hz, 3H, H-2), 5.51 (dd, ^3J = 3.5, 1.7 Hz, 3H, H-2$_{Man-1}$), 5.34 (dd, ^3J = 10.1, 3.5 Hz, 3H, H-3$_{Man-3}$), 5.29 (dd, ^3J = 3.4, 1.7 Hz, 3H, H-2$_{Man-3}$), 5.24 (t$_{app}$, ^3J = 10.1 Hz, 3H, H-4$_{Man-3}$), 5.16 (d, ^3J = 1.7 Hz, 3H, H-1$_{Man-1}$), 5.10 – 5.04 (m, 6H, H-3$_{Man-2}$, H-4$_{Man-2}$), 4.94 (d, ^3J = 1.9 Hz, 3H, H-1$_{Man-2}$), 4.88 (d, ^2J = 12.2 Hz, 3H, H-1a‘‘), 4.85 (d, ^3J = 1.7 Hz, 3H, H-1$_{Man-3}$), 4.84 (dd, ^3J = 2.9, 1.9 Hz, 3H, H-2$_{Man-2}$), 4.72 (d, ^2J = 12.2 Hz, 3H, H-1b‘‘), 4.58 (t, ^3J = 5.3 Hz, 6H, H-1‘), 4.45 (dd, ^3J = 9.8, 3.6 Hz, 3H, H-3$_{Man-1}$), 4.25 (ddd, ^3J = 10.3, 5.9, 2.1 Hz, 3H, H-5$_{Man-1}$), 4.17 – 4.11 (m, 6H, H-6a$_{Man-2}$, H-6a$_{Man-3}$), 4.07 – 4.01 (m, 6H, H-5$_{Man-2}$, H-5$_{Man-3}$), 3.99 – 3.90 (m, 18H, H-5a, H-2a‘, H-6a$_{Man-1}$, H-6b$_{Man-2}$, H-6b$_{Man-3}$), 3.70 (t, ^3J = 5.9 Hz, 6H, H-2), 3.67 – 3.57 (m, 36H, H-5b, H-2b‘, H-4‘, H-5‘, H-7‘, H-8‘, H-6a$_{Man-1}$), 3.55 (t, ^3J = 5.5 Hz, 6H, H-10‘), 3.44 (q, ^3J = 5.5 Hz, 6H, H-11‘), 2.43 (t, ^3J = 5.0 Hz, 3H, H-3), 2.11 (s, 9H, C-4$_{Man-3}$-O(C=O)CH_3), 2.04 (s, 9H, C-6$_{Man-3}$-O(C=O)CH_3), 2.03 (s, 9H, C-3$_{Man-3}$-O(C=O)CH_3), 1.96 (s, 9H, C-6$_{Man-2}$-O(C=O)CH_3), 1.92 (s, 9H, C-3$_{Man-2}$-O(C=O)CH_3), 1.91 (s, 9H, C-4$_{Man-2}$-O(C=O)CH_3), 1.84 (s, 9H, C-2$_{Man-2}$-O(C=O)CH_3), 1.81 (s, 9H, C-2$_{Man-3}$-O(C=O)CH_3). **^{13}C-NMR** (151 MHz, $CDCl_3$, 25 °C)**:** δ (ppm) = 171.3 (3C, C-1), 170.8 (3C, C-6$_{Man-2}$-O(C=O)CH_3), 170.6 (3C, C-6$_{Man-3}$-O(C=O)CH_3), 170.0 (3C, C-4$_{Man-3}$-O(C=O)CH_3), 169.9 (3C, C-3$_{Man-3}$-O(C=O)CH_3), 169.8 (3C, C-3$_{Man-2}$-O(C=O)CH_3), 169.6 (3C, C-4$_{Man-2}$-O(C=O)CH_3), 169.2 (3C, C-2$_{Man-3}$-O(C=O)CH_3), 169.1 (3C, C-2$_{Man-2}$-O(C=O)CH_3), 165.9 (3C, C-2$_{Man-1}$-O(C=O)Ph), 165.2 (3C, C-4$_{Man-1}$-O(C=O)Ph),

142.9 (3C, C-2''), 133.7 (3C, C-2$_{\text{Man-1}}$-O(*C*=O)Ph, C$_{para}$), 133.6 (3C, C-4$_{\text{Man-1}}$-O(*C*=O)Ph, C$_{para}$), 130.0 (6C, C-2$_{\text{Man-1}}$-O(*C*=O)Ph, C$_{ortho}$), 129.9 (6C, C-4$_{\text{Man-1}}$-O(*C*=O)Ph, C$_{ortho}$), 129.0 (3C, C-2$_{\text{Man-1}}$-O(*C*=O)Ph, C$_{ipso}$), 128.8 (6C, C-2$_{\text{Man-1}}$-O(*C*=O)Ph, C$_{meta}$), 128.7 (3C, C-4$_{\text{Man-1}}$-O(*C*=O)Ph, C$_{ipso}$), 128.5 (6C, C-4$_{\text{Man-1}}$-O(*C*=O)Ph, C$_{meta}$), 124.4 (3C, C-3''), 99.4 (3C, C-1$_{\text{Man-2}}$), 97.3 (3C, C-1$_{\text{Man-2}}$), 96.6 (3C, C-1$_{\text{Man-1}}$), 75.5 (3C, C-3$_{\text{Man-1}}$), 71.6 (3C, C-2$_{\text{Man-1}}$), 70.5, 70.5, 70.4, 70.1 (12C, C-4', C-5', C-7', C-8'), 69.8 (3C, C-10'), 69.5 (3C, C-5$_{\text{Man-1}}$), 69.4 (3C, C-5), 69.3 (3C, C-2'), 69.2 (3C, C-2$_{\text{Man-2}}$), 69.2 (3C, C-2$_{\text{Man-3}}$), 69.2 (3C, C-3$_{\text{Man-3}}$), 68.6 (3C, C-5$_{\text{Man-3}}$), 68.5 (3C, C-4$_{\text{Man-1}}$), 68.2 (3C, C-3$_{\text{Man-2}}$), 67.4 (3C, C-2), 66.5 (3C, C-6$_{\text{Man-1}}$), 65.9 (3C, C-4$_{\text{Man-2}}$), 65.7 (3C, C-4$_{\text{Man-3}}$), 62.3 (3C, C-6$_{\text{Man-2}}$), 62.2 (3C, C-6$_{\text{Man-3}}$), 60.4 (3C, C-1''), 50.6 (3C, C-6), 50.2 (3C, C-1'), 39.2 (3C, C-11'), 36.7 (3C, C-3), 20.9 (3C, C-4$_{\text{Man-3}}$-O(C=O)*C*H$_3$), 20.8 (3C, C-6$_{\text{Man-3}}$-O(C=O)*C*H$_3$), 20.7 (3C, C-3$_{\text{Man-3}}$-O(C=O)*C*H$_3$), 20.7 (3C, C-6$_{\text{Man-2}}$-O(C=O)*C*H$_3$), 20.7 (3C, C-3$_{\text{Man-2}}$-O(C=O)*C*H$_3$), 20.6 (3C, C-4$_{\text{Man-2}}$-O(C=O)*C*H$_3$), 20.5 (3C, C-2$_{\text{Man-2}}$-O(C=O)*C*H$_3$), 20.5 (3C, C-2$_{\text{Man-3}}$-O(C=O)*C*H$_3$). **HR-MS** (ESI$^+$)**:** $m/z_{\text{cal.}}$ = 1400.1698 $[M+3H]^{3+}$, $m/z_{\text{exp.}}$ = 1400.1699 $[M+3H]^{3+}$; $m/z_{\text{cal.}}$ = 1400.5042 $[M+3H]^{3+}$, $m/z_{\text{exp.}}$ = 1400.5041 $[M+3H]^{3+}$. $\boldsymbol{R_f}$ (NP) = 0.30 (DCM/MeOH 20:1). **Optical rotation** (LM)**:** $[\alpha]_{\mathrm{D}}^{22}$ = +9.50 (CHCl$_3$). **IR** (ATR)**:** (cm^{-1}) = 2935, 1748, 1663, 1452, 1369, 1224, 1046, 979, 915, 715, 449.

4.1.25 Synthesis of *N*-(1-Bis(17-(4-(2,4-di-*O*-benzoyl-3,6-di-*O*-(2,3,4,6-tetra-*O*-acetyl-α-D-mannopyranosyl)-α-D-mannopyranosyloxymethyl)-1*H*-1,2,3-triazol-1-yl)-5-oxo-2,9,12,15-tetraoxa-6-azaheptadec-1-yl)-18-(4-(2,4-di-*O*-benzoyl-3,6-di-*O*-(2,3,4,6-tetra-*O*-acetyl-α-D-mannopyranosyl)-α-D-mannopyranosyloxymethyl)-1*H*-1,2,3-triazol-1-yl)-6-oxo-3,10,13,16-tetraoxa-7-azaoctadec-1-yl)-12-azido-4,7,10-pentaoxadodecanamide

The title compound was synthesized according to Scheme S 27 following modified synthesis protocols by *Cheng et al.*[44] and *Krumb et al.*[26]

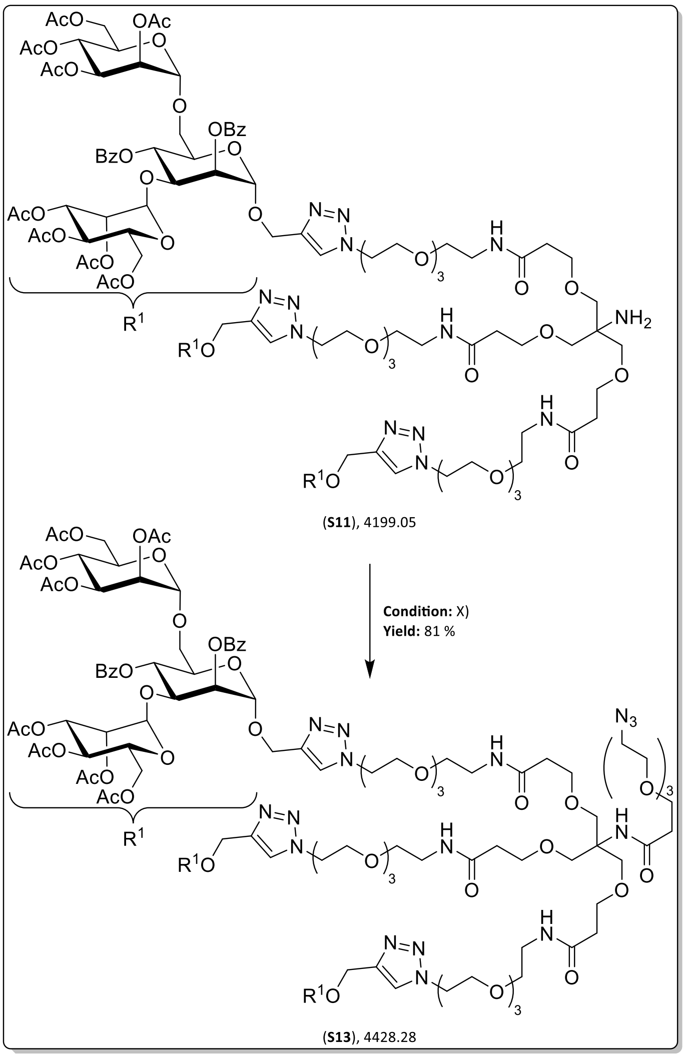

Scheme S 27: Synthesis of *N*-(1-Bis(17-(4-(2,4-di-*O*-benzoyl-3,6-di-*O*-(2,3,4,6-tetra-*O*-acetyl-α-D-mannopyranosyl)-α-D-mannopyranosyloxymethyl)-1*H*-1,2,3-triazol-1-yl)-5-oxo-2,9,12,15-tetraoxa-6-azaheptadec-1-yl)-18-(4-(2,4-di-*O*-benzoyl-3,6-di-*O*-(2,3,4,6-tetra-*O*-acetyl-α-D-mannopyranosyl)-α-D-mannopyranosyloxymethyl)-1*H*-1,2,3-triazol-1-yl)-6-oxo-3,10,13,16-tetraoxa-7-azaoctadec-1-yl)-12-azido-4,7,10-pentaoxadodecanamide (**S13**). Reaction condition X) (**3**), HATU, DIPEA, Ar-atm., DMF, rt, 48 h; yield: 81 %.

The reaction was carried out in a dried Schlenk vessel equipped with a magnetic stirring bar. 12-Azido-4,7,10-trioxadodecanoic acid (**3**, 0.11 g, 0.44 mmol, 10.0 eq.) was dissolved in absolute DMF (1.25 mL) under an atmosphere of argon. HATU (0.17 g, 0.44 mmol, 10.0 eq.) was added, followed by DIPEA (0.07 g, 0.09 mL, 0.52 mmol, 13.0 eq.) via syringe. The reaction mixture was stirred for half an hour at room temperature. *N*-(1-(4-(2,4-Di-*O*-benzoyl-3,6-di-*O*-(2,3,4,6-tetra-*O*-acetyl-α-D-mannopyranosyl)-α-D-mannopyranosyloxymethyl)-1*H*-1,2,3-triazol-1-yl)-3,6,9-trioxaundec-11-yl)-6-amino-6-(17-(4-

(2,4-di-*O*-benzoyl-3,6-di-*O*-(2,3,4,6-tetra-*O*-acetyl-α-D-mannopyranosyl)-α-D-manno-pyranosyloxy-methyl)-1*H*-1,2,3-triazol-1-yl)-5-oxo-2,9,12,15-tetraoxa-6-azaheptadec-1-yl)-11-oxo-4,8,15,18,21-pentaoxa-12-aza-(23-(4-(2,4-di-*O*-benzoyl-3,6-di-*O*-(2,3,4,6-tetra-*O*-acetyl-α-D-mannopyranosyl)-α-D-mannopyranosyloxymethyl)-1*H*-1,2,3-triazol-1-yl)tricosanamide (**S11**, 0.17 g, 0.04 mmol, 1.00 eq.) was dissolved in absolute DMF (1.75 mL) under an atmosphere of argon and slowly added dropwise to the yellowish reaction mixture using a syringe. The mixture was stirred at room temperature for 48 hours. Complete conversion of the limiting substrate and the formation of the desired product were determined by reaction control via TLC and HPLC-ELS-MS. All volatiles were removed in fine vacuum. Toluene (3 x 5.00 mL) was added to the review and all volatiles were removed in fine vacuum. The crude product (0.49 g) was obtained in the form of orange oil and purified by FCC (DCM/MeOH, isocratic at 10 % MeOH) followed by RP-FCC (MeCN/H_2O, gradient from 10 % to 85 % to 100 % MeCN, Isolera One Purification System, SNAP C_{18} 12 g cartridge). The pure product (**S13**) was obtained in the form of yellowish oil.

Yield: 0.13 g (0.03 mmol, 81 %), colorless oil.

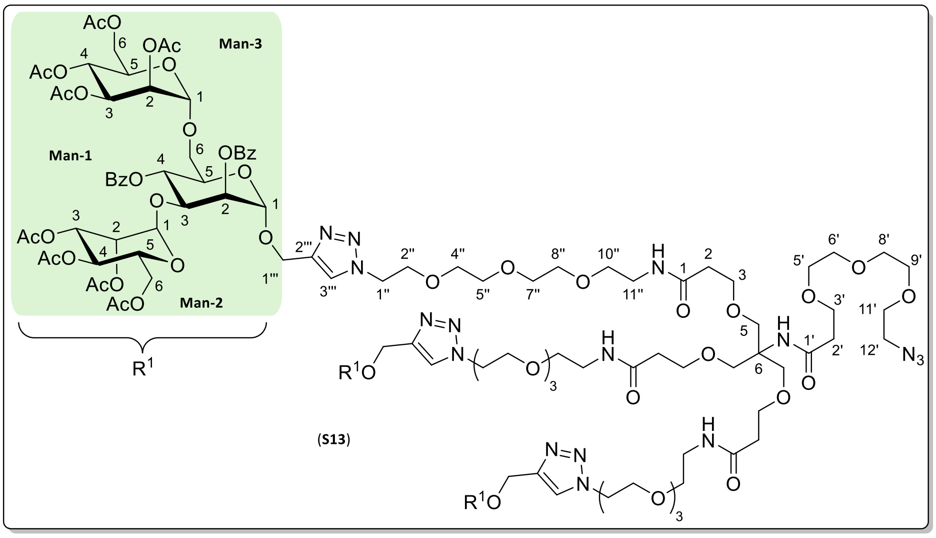

^{1}H-NMR (600 MHz, $CDCl_3$, 25 °C)**:** δ (ppm) = 8.15 – 8.12 (m, 6H, C-2$_{Man-1}$-O(C=O)Ph, H$_{ortho}$), 8.04 – 8.01 (m, 6H, C-4$_{Man-1}$-O(C=O)Ph, H$_{ortho}$), 7.83 (s, 3H, H-2‘‘‘), 7.64 – 7.60 (m, 3H, C-2$_{Man-1}$-O(C=O)Ph, H$_{para}$), 7.59 – 7.56 (m, 3H, C-4$_{Man-1}$-O(C=O)Ph, H$_{para}$), 7.56 – 7.53 (m, 6H, C-2$_{Man-1}$-O(C=O)Ph, H$_{meta}$), 7.46 – 7.42 (m, 6H, C-4$_{Man-1}$-O(C=O)Ph, H$_{meta}$), 6.72 (t, ^3J = 5.5 Hz, 3H, -N*H*-C-11‘‘), 6.60 (s, 1H, -N*H*-C-6), 5.70 (t$_{app}$, ^3J = 10.0 Hz, 3H, H-4$_{Man-1}$), 5.51 (dd, ^3J = 3.5, 1.8 Hz, 3H, H-2$_{Man-1}$), 5.34 (dd, ^3J = 10.1, 3.4 Hz, 3H, H-3$_{Man-3}$), 5.28 (dd, ^3J = 3.4, 1.7 Hz, 3H, H-2$_{Man-3}$), 5.24 (t, ^3J = 10.1 Hz, 3H, H-4$_{Man-3}$), 5.16 (d, ^3J = 1.7 Hz, 3H, H-1$_{Man-1}$), 5.10 – 5.04 (m, 6H, H-3$_{Man-2}$, H-4$_{Man-2}$), 4.94 (d, ^3J = 1.9 Hz, 3H, H-1$_{Man-2}$), 4.88 (d, ^2J = 12.1 Hz, 3H, H-1a‘‘‘), 4.85 (d, ^3J = 1.7 Hz, 3H, H-1$_{Man-3}$), 4.84 (dd, ^3J = 2.9, 1.9 Hz, 3H, H-2$_{Man-2}$), 4.71 (d, ^2J = 12.1 Hz, 3H, H-1b‘‘‘), 4.58 (t, ^3J = 5.3 Hz, 6H, H-1‘‘), 4.45 (dd, ^3J = 9.8, 3.4 Hz, 3H, H-3$_{Man-1}$), 4.25 (ddd, ^3J = 10.3, 6.0, 2.2 Hz, 3H, H-5$_{Man-1}$), 4.14 (ddd, ^2J = 12.2, 8.0, 5.5 Hz, 6H, H-6a$_{Man-2}$, H-6a$_{Man-3}$), 4.07 – 4.02 (m, 6H, H-5$_{Man-2}$, H-5$_{Man-3}$), 3.98 – 3.90 (m, 15H, H-5a, H-2a‘‘, H-6a$_{Man-1}$, H-6b$_{Man-2}$, H-6b$_{Man-3}$), 3.69 (t, ^3J = 5.6 Hz, 8H, H-2, H-3‘), 3.68 – 3.66 (m, 6H, H-5b, H-2b‘‘), 3.65 – 3.58 (m, 37H, H-5‘, H-6‘, H-8‘, H-9‘, H-11‘, H-4‘‘, H-5‘‘, H-7‘‘, H-8‘‘, H-6b$_{Man-1}$), 3.54 (t, ^3J = 5.5 Hz, 6H, H-10‘‘), 3.43 (q, ^3J = 5.5 Hz, 6H, H-11‘‘), 3.39 (t, ^3J = 5.0 Hz, 3H, H-12‘), 2.45 (t, ^3J = 6.0 Hz, 2H, H-2‘), 2.41 (t, ^3J = 5.9 Hz, 6H, H-3), 2.11 (s, 9H, C-4$_{Man-3}$-O(C=O)C*H*$_3$), 2.04 (s, 9H, C-6$_{Man-3}$-O(C=O)C*H*$_3$), 2.03 (s, 9H, C-3$_{Man-3}$-O(C=O)C*H*$_3$), 1.96 (s, 9H, C-6$_{Man-3}$-O(C=O)C*H*$_3$), 1.92 (s, 9H, C-3$_{Man-2}$-O(C=O)C*H*$_3$), 1.91 (s, 9H, C-4$_{Man-2}$-O(C=O)C*H*$_3$), 1.84 (s, 9H, C-2$_{Man-2}$-O(C=O)C*H*$_3$), 1.81 (s, 9H,

C-2$_{\text{Man-3}}$-O(C=O)C*H*$_3$). **^{13}C-NMR** (151 MHz, CDCl$_3$, 25 °C)**:** *δ* (ppm) = 171.7 (1C, C-1‘), 171.3 (3C, C-1), 170.7 (3C, C-6$_{\text{Man-2}}$-O(*C*=O)CH$_3$), 170.6 (3C, C-6$_{\text{Man-3}}$-O(*C*=O)CH$_3$), 170.0 (3C, C-4$_{\text{Man-3}}$-O(*C*=O)CH$_3$), 169.9 (3C, C-3$_{\text{Man-3}}$-O(*C*=O)CH$_3$), 169.8 (3C, C-3$_{\text{Man-2}}$-O(*C*=O)CH$_3$), 169.6 (3C, C-4$_{\text{Man-2}}$-O(*C*=O)CH$_3$), 169.2 (3C, C-2$_{\text{Man-3}}$-O(*C*=O)CH$_3$), 169.1 (3C, C-2$_{\text{Man-2}}$-O(*C*=O)CH$_3$), 165.9 (3C, C-2$_{\text{Man-1}}$-O(*C*=O)Ph), 165.2 (3C, C-4$_{\text{Man-1}}$-O(*C*=O)Ph), 142.9 (3C, C-2‘‘‘), 133.7 (3C, C-2$_{\text{Man-1}}$-O(*C*=O)Ph, C$_{para}$), 133.6 (3C, C-4$_{\text{Man-1}}$-O(*C*=O)Ph, C$_{para}$), 130.0 (6C, C-2$_{\text{Man-1}}$-O(*C*=O)Ph, C$_{ortho}$), 129.9 (6C, C-4$_{\text{Man-1}}$-O(*C*=O)Ph, C$_{ortho}$), 129.0 (3C, C-2$_{\text{Man-1}}$-O(*C*=O)Ph, C$_{ipso}$), 128.8 (6C, C-2$_{\text{Man-1}}$-O(*C*=O)Ph, C$_{meta}$), 128.7 (3C, C-4$_{\text{Man-1}}$-O(*C*=O)Ph, C$_{ipso}$), 128.5 (3C, C-4$_{\text{Man-1}}$-O(*C*=O)Ph, C$_{meta}$), 124.4 (3C, C-3‘‘‘), 99.4 (3C, C-1$_{\text{Man-2}}$), 97.3 (3C, C-1$_{\text{Man-3}}$), 96.6 (3C, C-1$_{\text{Man-1}}$), 75.5 (3C, C-3$_{\text{Man-1}}$), 71.6 (3C, C-2$_{\text{Man-1}}$), 70.6, 70.5, 70.5, 70.5, 70.4, 70.2, 70.1, 70.0 (16C, H-5‘, H-6‘, H-8‘, H-9‘, H-4‘‘, H-5‘‘, H-7‘‘, H-8‘‘), 69.8 (3C, C-10‘‘), 69.4 (3C, C-5$_{\text{Man-1}}$), 69.4 (3C, C-5), 69.3 (3C, C-2‘‘), 69.2 (3C, C-2$_{\text{Man-2}}$), 69.2 (3C, C-2$_{\text{Man-3}}$), 69.2 (3C, C-3$_{\text{Man-3}}$), 69.2 (1C, C-11‘), 68.6 (3C, C-5$_{\text{Man-3}}$), 68.5 (3C, C-4$_{\text{Man-1}}$), 68.2 (3C, C-3$_{\text{Man-2}}$), 67.3 (3C, C-2), 67.3 (3C, C-3‘), 66.4 (3C, C-6$_{\text{Man-1}}$), 65.9 (3C, C-4$_{\text{Man-2}}$), 65.7 (3C, C-4$_{\text{Man-3}}$), 62.3 (3C, C-6$_{\text{Man-2}}$), 62.2 (3C, C-6$_{\text{Man-3}}$), 60.4 (3C, C-1‘‘‘), 59.7 (1C, C-6), 50.6 (1C, C-12‘), 50.2 (3C, C-1‘‘), 39.2 (3C, C-11‘‘), 37.4 (1C, C-2‘), 36.6 (3C, C-3), 20.9 (3C, C-4$_{\text{Man-3}}$-O(C=O)*C*H$_3$), 20.8 (3C, C-6$_{\text{Man-3}}$-O(C=O)*C*H$_3$), 20.7 (3C, C-3$_{\text{Man-3}}$-O(C=O)*C*H$_3$), 20.7 (3C, C-6$_{\text{Man-2}}$-O(C=O)*C*H$_3$), 20.7 (3C, C-3$_{\text{Man-2}}$-O(C=O)*C*H$_3$), 20.6 (3C, C-4$_{\text{Man-2}}$-O(C=O)*C*H$_3$), 20.5 (3C, C-2$_{\text{Man-2}}$-O(C=O)*C*H$_3$), 20.5 (3C, C-2$_{\text{Man-3}}$-O(C=O)*C*H$_3$). **HR-MS** (ESI$^+$)**:** $m/z_{\text{cal.}}$ = 1476.5385 [M+3H]$^{3+}$, $m/z_{\text{exp.}}$ = 1476.5380 [M+3H]$^{3+}$; $m/z_{\text{cal.}}$ = 1476.8730 [M+3H]$^{3+}$, $m/z_{\text{exp.}}$ = 1476.8724 [M+3H]$^{3+}$; $m/z_{\text{cal.}}$ = 2214.3042 [M+2H]$^{2+}$, $m/z_{\text{exp.}}$ = 2214.3033 [M+2H]$^{2+}$; $m/z_{\text{cal.}}$ = 2214.8058 [M+2H]$^{2+}$, $m/z_{\text{exp.}}$ = 2214.8053 [M+2H]$^{2+}$. ***R_f*** (NP) = 0.20 (DCM/MeOH 25:1), 0.48 (DCM/MeOH 10:1). **Optical rotation** (LM)**:** $[\boldsymbol{\alpha}]_{\mathbf{D}}^{\mathbf{22}}$ = +11.0 (CHCl$_3$). **IR** (ATR)**:** (cm^{-1}) = 2919, 2260, 2185, 2104, 2039, 2023, 1979, 1749, 1670, 1453, 1370, 1225, 1092, 1048, 983, 715, 598, 548, 537, 524, 497, 464, 456, 437, 422, 408.

4.1.26 Synthesis of *N*-(1-Bis(17-(4-(3,6-di-*O*-(α-D-mannopyranosyl)-α-D-mannopyranosyloxymethyl)-1*H*-1,2,3-triazol-1-yl)-5-oxo-2,9,12,15-tetraoxa-6-azaheptadec-1-yl)-18-(4-(3,6-di-*O*-(α-D-mannopyranosyl)-α-D-mannopyranosyloxymethyl)-1*H*-1,2,3-triazol-1-yl)-6-oxo-3,10,13,16-tetraoxa-7-azaoctadec-1-yl)-12-azido-4,7,10-pentaoxadodecanamide

The title compound was synthesized according to Scheme S 28 following modified synthesis protocols by *Krumb et al.*[26] sowie *Percec et al.*[11]

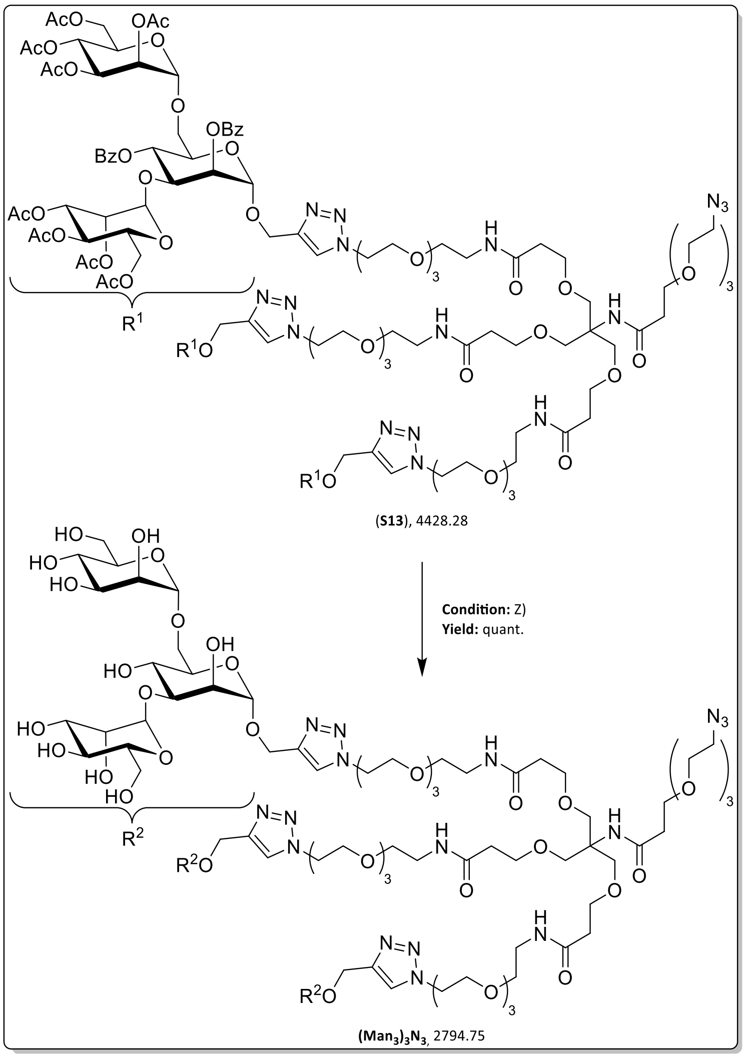

Scheme S 28: Synthesis of *N*-(1-Bis(17-(4-(3,6-di-*O*-(α-D-mannopyranosyl)-α-D-mannopyranosyloxymethyl)-1*H*-1,2,3-triazol-1-yl)-5-oxo-2,9,12,15-tetraoxa-6-azaheptadec-1-yl)-18-(4-(3,6-di-*O*-(α-D-mannopyranosyl)-α-D-mannopyranosyloxymethyl)-1*H*-1,2,3-triazol-1-yl)-6-oxo-3,10,13,16-tetraoxa-7-azaoctadec-1-yl)-12-azido-4,7,10-pentaoxadodecanamide **$(Man_3)_3N_3$**. Reaction conditions: Z) NaOMe, NaOH, MeOH, Ar-atm., rt, 18 h; yield: quant.

The reaction was carried out in a dried Schlenk vessel, which had previously been equipped with a magnetic stirring bar. *N*-(1-Bis(17-(4-(2,4-di-*O*-benzoyl-3,6-di-*O*-(2,3,4,6-tetra-*O*-acetyl-α-D-mannopyranosyl)-α-D-mannopyranosyloxymethyl)-1*H*-1,2,3-triazol-1-yl)-5-oxo-2,9,12,15-tetraoxa-6-azaheptadec-1-yl)-18-(4-(2,4-di-*O*-benzoyl-3,6-di-*O*-(2,3,4,6-tetra-*O*-acetyl-α-D-mannopyranosyl)-α-D-mannopyranosyloxymethyl)-1*H*-1,2,3-triazol-1-yl)-6-oxo-3,10,13,16-tetraoxa-7-azaoctadec-1-yl)-12-azido-4,7,10-pentaoxadodecanamide (**S13**, 0.10 g, 0.02 mmol, 1.00 eq.) was dissolved in absolute MeOH (10.0 mL) under an atmosphere of argon. NaOMe (4.00 mg, 0.07 mmol, 3.00 eq.) and freshly ground NaOH (3.00 mg, 0.07 mmol, 3.00 eq.) were added, and the resulting solution was stirred at room temperature for 18 hours. Complete conversion of the limiting substrate and the formation of the desired

product were determined by reaction control via TLC and HPLC-ELS-MS. The reaction was ended by addition of the ion exchange resin *Amberlite IR 120* (0.20 g), and the mixture was stirred for 10 minutes until a constant pH (pH = 6 – 7) was reached. The reaction mixture was filtered through a glass frit with *Celite* and eluted with MeOH (1 x 50.0 mL). All volatiles were removed in vacuo. The crude product (0.18 g) was obtained in the form of yellowish oil and purified by RP-FCC (MeCN/H_2O, gradient from 5 % to 25 % to 100 % MeCN, Isolera One Flash Purification System, SNAP C_{18} 12 g cartridge). The pure product **$(Man_3)_3N_3$** was obtained in the form of a yellowish lyophilizate.

Yield: 0.06 g (0.02 mmol, quant.), yellowish lyophilizate.

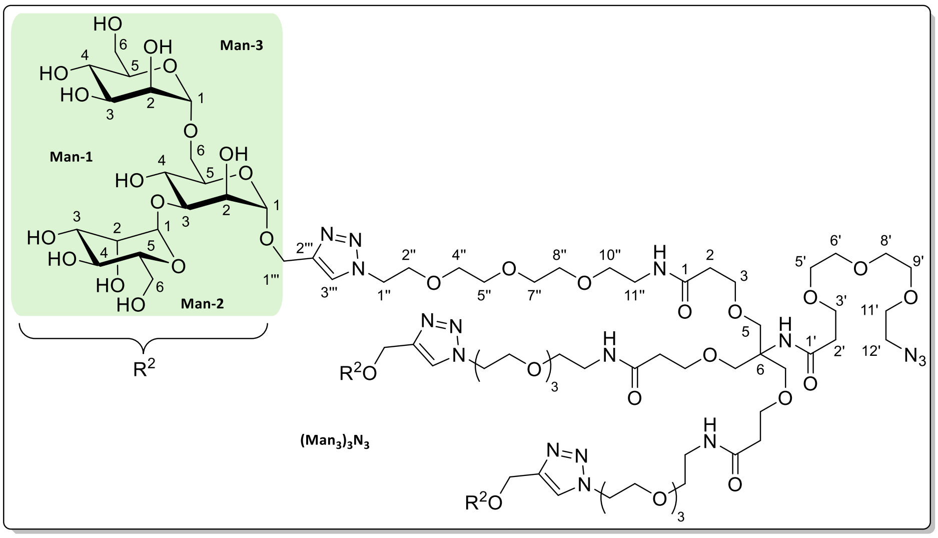

^{1}H-NMR (600 MHz, D_2O, 25 °C)**:** δ (ppm) = 8.07 (s, 3H, H-3‘‘‘), 5.01 (s, 3H, H-1$_{Man-2}$), 4.87 (s, 3H, H-1$_{Man-3}$), 4.83 (s, 3H, H-1$_{Man-3}$), 4.75 (d, ^2J = 12.5 Hz, 3H, H-1a‘‘‘), 4.66 (d, ^2J = 12.5 Hz, 3H, H-1b‘‘‘), 4.58 (t, ^3J = 5.0 Hz, 6H, H-1‘‘), 4.03 (s, 3H, H-2$_{Man-1}$), 3.99 (dd, ^3J = 3.5, 1.8 Hz, 3H, H-2$_{Man-2}$), 3.97 – 3.90 (m, 21H, H-2‘‘, H-10‘‘, H-2$_{Man-3}$, H-6$_{Man-1}$), 3.87 – 3.76 (m, 15H, H-3$_{Man-1}$, H-3$_{Man-2}$, H-3$_{Man-3}$, H-4$_{Man-1}$, H-6a$_{Man-3}$), 3.76 – 3.50 (m, 86H, H-2, H-5, H-3‘, H-5‘, H-6‘, H-8‘, H-9‘, H-11‘, H-2‘‘, H-4‘‘, H-5‘‘, H-7‘‘, H-8‘‘, H-10‘‘, H-4$_{Man-2}$, H-4$_{Man-3}$, H-5$_{Man-1}$, H-5$_{Man-2}$, H-5$_{Man-3}$, H-6$_{Man-2}$, H-6b$_{Man-3}$), 3.42 (t, ^3J = 4.9 Hz, 3H, H-12‘), 3.33 (t, ^3J = 5.5 Hz, 6H, H-11‘‘), 2.43 (t, ^3J = 6.3 Hz, 8H, H-3, H-2‘). **^{13}C-NMR** (151 MHz, D_2O, 25 °C)**:** δ (ppm) = 174.1 (3C, C-1), 173.7 (3C, C-1‘), 143.9 (3C, C-2‘‘‘), 125.7 (3C, C-3‘‘‘), 102.4 (3C, C-1$_{Man-2}$), 99.7 (3C, C-1$_{Man-3}$), 99.4 (3C, C-1$_{Man-1}$), 78.6 (3C, C-3$_{Man-1}$), 73.3 (3C, C-5$_{Man-2}$), 72.7 (3C, C-1$_{Man-3}$), 71.3 (3C, C-5$_{Man-1}$), 70.6 (3C, C-3$_{Man-3}$), 70.3 (3C, C-3$_{Man-2}$), 70.0 (3C, C-2$_{Man-2}$), 69.9 (3C, C-2$_{Man-3}$), 69.6, 69.6, 69.5, 69.5, 69.5, 69.5 (17C, C-5‘, C-6‘, C-8‘, C-9‘, C-4‘‘, C-5‘‘, C-7‘‘, C-8‘‘, H-2$_{Man-1}$), 69.4 (3C, C-5), 69.2 (1C, C-11‘), 68.8 (3C, C-10‘‘), 68.7 (3C, C-2‘‘), 68.4 (1C, C-3‘), 67.5 (3C, C-2), 66.7 (3C, C-4$_{Man-3}$), 66.6 (3C, C-6$_{Man-2}$), 65.4 (3C, C-4$_{Man-1}$), 64.9 (3C, C-6$_{Man-1}$), 60.9 (3C, C-6$_{Man-3}$), 60.3 (1C, C-6), 60.0 (3C, C-1‘‘‘), 50.1 (3C, C-1‘‘), 50.0 (1C, C-12‘), 39.0 (3C, C-11‘‘), 36.4 (1C, C-2‘), 36.1 (3C, C-3). **HR-MS** (ESI$^+$)**:** $m/z_{cal.}$ = 932.0671 $[M+3H]^{3+}$, $m/z_{exp.}$ = 932.0679 $[M+3H]^{3+}$; $m/z_{cal.}$ = 932.4016 $[M+3H]^{3+}$, $m/z_{exp.}$ = 932.4034 $[M+3H]^{3+}$; $m/z_{cal.}$ = 1419.5790 $[M+2Na]^{2+}$, $m/z_{exp.}$ = 1419.5789 $[M+2Na]^{2+}$; $m/z_{cal.}$ = 1420.0807 $[M+2Na]^{2+}$, $m/z_{exp.}$ = 1420.0812 $[M+2Na]^{2+}$. **R_f** (RP) = 0.79 (MeCN/H_2O 1:1). **Optical rotation** (LM)**:** $[\alpha]_D^{22}$ = +90.7 (H_2O). **IR** (ATR)**:** (cm^{-1}) = 3386, 2913, 2129, 1641, 1544, 1458, 1051, 809, 545, 443.

4.2 Synthesis of the TS_n-HSA neoglycoalbumins

Prior to use, native HSA was dissolved in MQ-water, subjected to spin-filtration through a 300 kDa ultracentrifugation tube to remove large aggregates, and lyophilized. The mass of all HSA conjugates is expressed as the *m/z* value of the peak of the singly charged species and denoted as *M*. In the case of repeated synthesis, synthesis protocols are reported representatively. Scheme S 29 and Scheme S 30 show an overview of the synthesis route for all NCs.

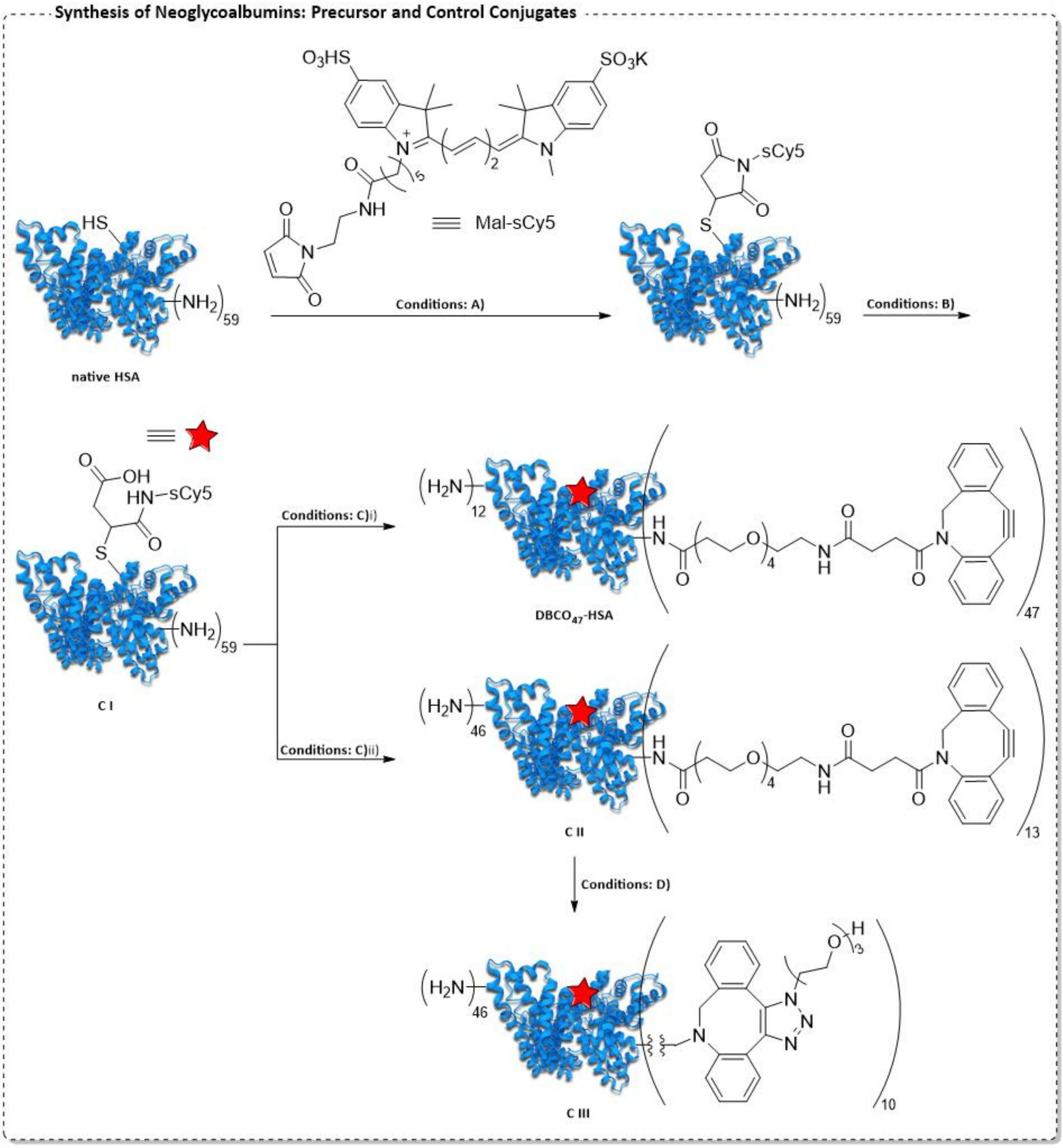

Scheme S 29: Synthesis of the precursor conjugates and control conjugates C I–III. Reaction conditions: A) 50 mM PB pH 7.4, RT, 4 h; B) 50 mM Na-borate buffer pH 9.2, 37 °C, o.n.; C) 50 mM PB pH 7.4, RT, 1 d; i) 19 eq. NHS-PEG_4-DBCO; ii) 150 eq. NHS-PEG_4-DBCO; D) N_3-PEG_3-OH, 50 mM PB (8 M urea, 2 mM EDTA) pH 8, RT.

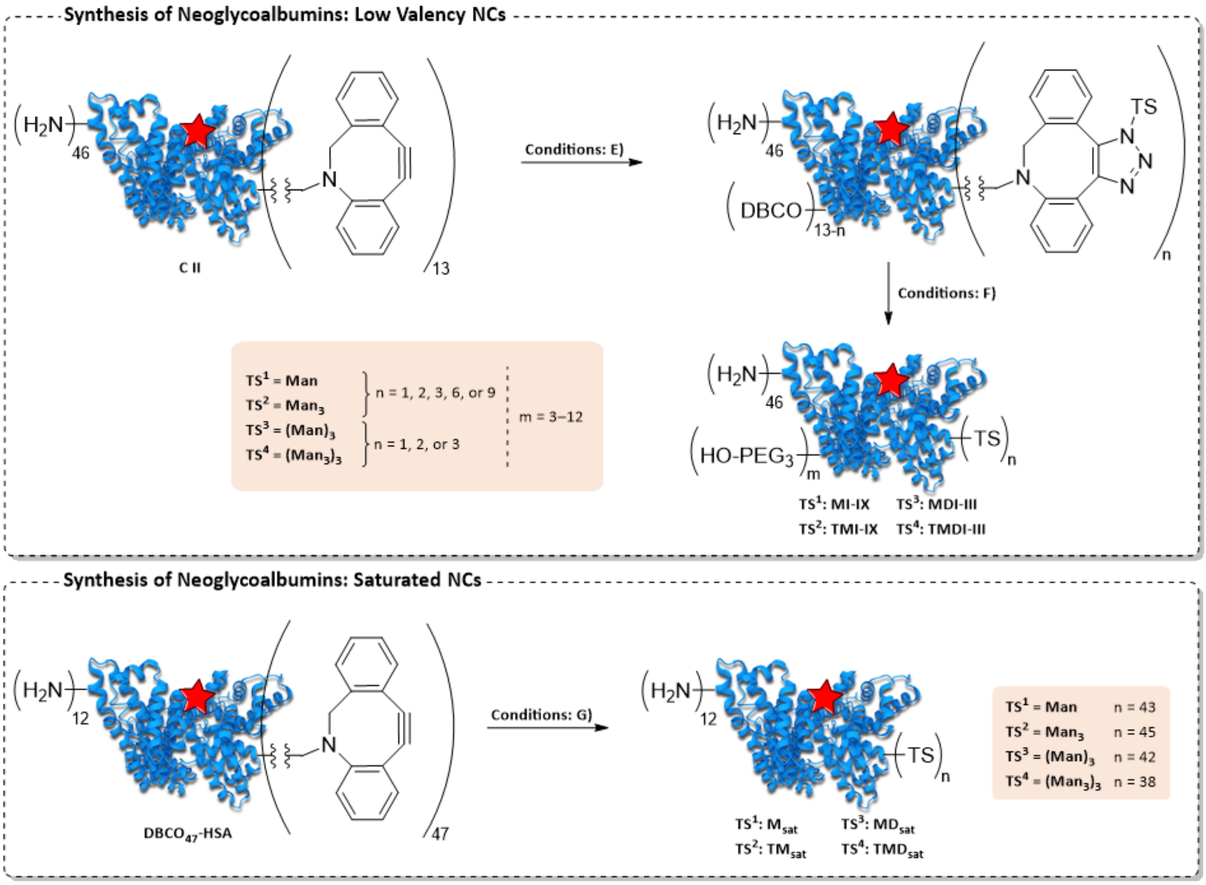

Scheme S 30: Synthesis of low (top) and high (bottom) valency NCs. Reaction conditions: 50 mM PB (8 M urea, 2 mM EDTA) pH 8, RT; E) 1–12 eq. TS; F) 63–100 eq. N_3-PEG$_3$-OH; G) 264 eq. TS.

Degree of TS modification via SPAAC was monitored with MALDI-ToF-MS and calculated with conjugate M III ([Man]3-HSA) used as an example as follows:

$$\mathrm{DoM_{TS}} = \frac{(M_{MIII\,post\,glycosylation} - M_{CII})}{(M_{Man})} = \frac{(75815 g\cdot mol^{-1} - 74440 g\cdot mol^{-1})}{(462.46 g\cdot mol^{-1})} = \frac{1375 g\cdot mol^{-1}}{462.46 g\cdot mol^{-1}} = 3.0$$

$\mathrm{DoM_{TS}} = 3.0$

After glycosylation, residual DBCO groups were capped using 2-[2-(2-azidoethoxy)ethoxy]ethan-1-ol (N_3-PEG$_3$-OH). Conjugate masses *M* are always final, capped NCs unless otherwise noted.

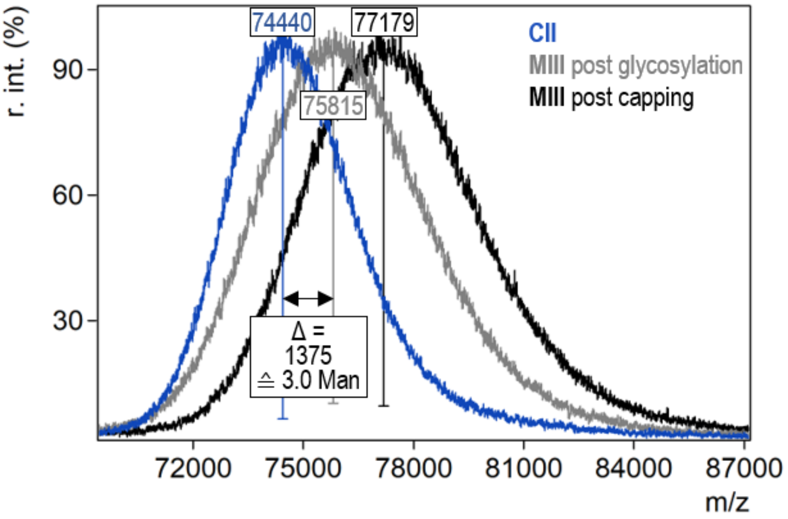

4.2.1 Preparation of dye-labeled HSA via thiol-maleimide Michael addition

Native HSA (40 mg, 0.60 µmol, 1.0 eq.) was dissolved in 50 mM sodium phosphate buffer (pH = 7.4) to obtain a protein concentration of 5 mg/mL. Sulfo-Cy5 maleimide (967 µg, 1.20 µmol, 2.0 eq.) dissolved in MQ (25 mg/mL) was added to the native HSA. The reaction was shaken at room temperature for 4 hours and afterwards purified (5 x MQ) and concentrated *via* ultracentrifugation spin filter (Vivaspin, PES, 10 kDa). The product was lyophilized and obtained in the form of a blue lyophilizate.

Recovery rate: 31 mg (0.46 µmol, 77 %), blue lyophilizate.

MALDI-ToF-MS: m/z = 67474 $[M+H]^+$

4.2.2 Maleimide ring opening of labeled HSA (C I)

Labeled HSA (31 mg, 0.46 µmol) was diluted in 50 mM sodium borate buffer (pH = 9.2) to a final protein concentration of 1 mg/mL and shaken at 37 °C for 24 h. Afterwards, it was concentrated and purified (5 x MQ) *via* ultracentrifugation spin filter (Vivaspin, PES, 10 kDa). The product, henceforth referred to as HSA, was lyophilized and obtained in the form of a blue lyophilizate.

Recovery rate: 31 mg (0.46 µmol, quantitative), blue lyophilizate.

MALDI-ToF-MS: m/z = 67422 $[M+H]^+$

4.2.3 Preparation of $DBCO_{13}$-HSA *via* NHS-ester activated DBCO-linker (C II)

Labeled, ring-opened HSA (22.5 mg, 339 nmol, 1.0 eq) was diluted in 50mM phosphate buffer (pH = 7.4, 11.3 mL) to a protein concentration of 2 mg/mL. DBCO-PEG_4-NHS (4.19 mg, 6.44 µmol, 19 eq.) dissolved in DMSO (100 mg/mL) was diluted in 50mM phosphate buffer (pH = 7.4, 11.3 mL) and added. The reaction was shaken at room temperature for 1 day. Afterwards, the reaction solution was purified (5 x MQ) and concentrated *via* ultracentrifugation spin filter (Vivaspin, PES, 10 kDa). The product was lyophilized and obtained in the form of a blue lyophilizate.

Recovery rate: 22 mg (298 nmol, 88 %), blue lyophilizate.

MALDI-ToF-MS: m/z = 74440 $[M+H]^+$

4.2.4 Preparation of $DBCO_{47}$-HSA *via* NHS-ester activated DBCO-linker

Labeled, ring-opened HSA (2.0 mg, 30 nmol, 1.0 eq) was diluted in 50mM phosphate buffer (pH = 7.4, 0.75 mL) to a protein concentration of 2.7 mg/mL. DBCO-PEG_4-NHS (2.9 mg, 495 µmol, 149 eq.) dissolved in DMSO (100 mg/mL) was added in several portions and the reaction solution was shaken at room temperature for 1 day. Afterwards, the reaction solution was purified (5 x MQ) and concentrated *via* ultracentrifugation spin filter (Vivaspin, PES, 10 kDa). The product was lyophilized and obtained in the form of a blue lyophilizate.

Recovery rate: 2.9 mg (32 nmol, quantitative), blue lyophilizate.

MALDI-ToF-MS: m/z = 90973 $[M+H]^+$

4.2.5 Preparation of HO-PEG_3-HSA control C III

$DBCO_{13}$-HSA (0.50 mg, 6.72 nmol, 1.0 eq.) was dissolved in 50 mM phosphate buffer with 8 M urea and 2 mM EDTA (pH = 8) to a final protein concentration of 1 mg/mL. N_3-PEG_3-OH (295 nmol, 44.0 eq. or 3.4 eq/DBCO) diluted in DMSO (71 mM, 4.16 µL) was added in two portions and the reaction solution was inverted (10 rpm) at room temperature until no further functionalization could be observed. Afterwards, the reaction solution was purified (5 x MQ) and concentrated via

ultracentrifugation spin filter (Vivaspin, PES, 10 kDa). The product was lyophilized and obtained in the form of a blue lyophilizate.

Recovery rate: 0.45 mg (5.92 nmol, 88 %), blue lyophilizate.

MALDI-ToF-MS: *m/z* = 76184 $[M+H]^+$

4.2.6 Preparation of low valency TS-HSA neoglycoalbumins

General Procedure: $DBCO_{13}$-HSA (reaction scale: 0.5 – 1.0 mg, 6.7 – 13.4 nmol) was dissolved in 50 mM phosphate buffer with 8 M urea and 2 mM EDTA (pH = 8) to a final protein concentration of 1 mg/mL. The azide functionalized TS (1.2 – 12 eq., see Table S 1) dissolved in MQ (10 mg/mL) was added and the reaction solution was inverted (10 rpm) at room temperature. The reaction was closely monitored *via* Maldi-ToF-MS analysis until the desired modification rate was achieved (3 – 70 h). Afterwards, the reaction solution was purified (2 – 3 x reaction buffer) and capping reagent (71 mM in DMSO, 63 – 100 eq.) was added in excess overnight. The product was purified (1 x reaction buffer, 5 x MQ) and concentrated via ultracentrifugation spin filter (Vivaspin, PES, 10 kDa). The product was lyophilized and obtained in the form of a blue lyophilizate. All low valency conjugates were obtained with recovery rates ranging from 38 % to quantitative.

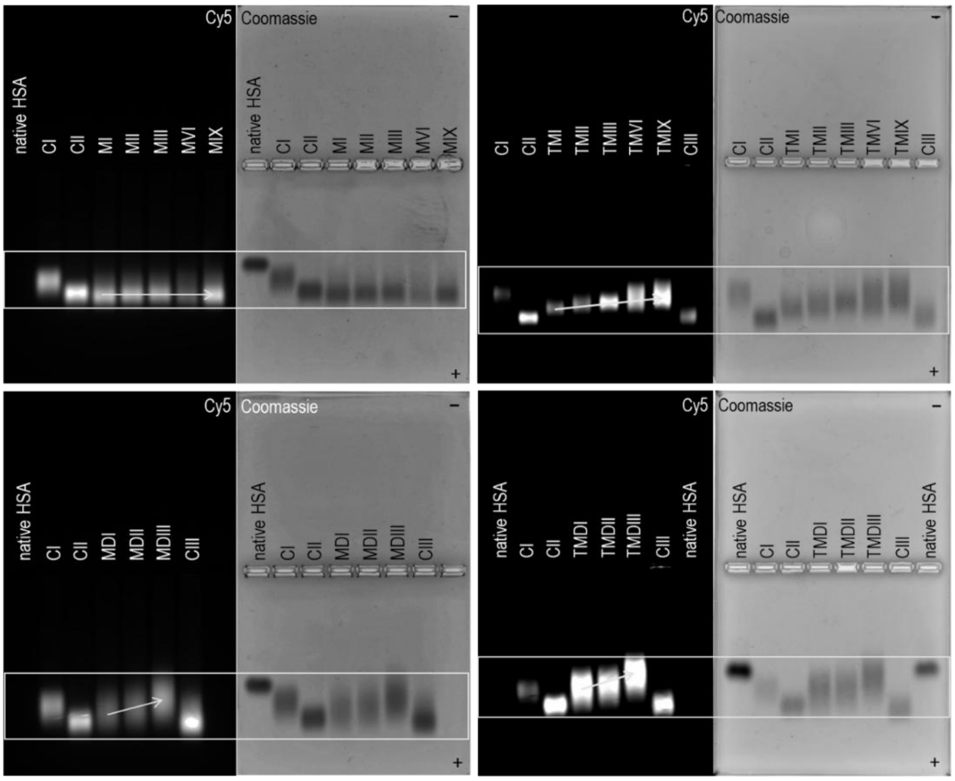

Figure S 1: Agarose gel electrophoresis of low valency NCs carrying varying amounts of different TSs (top left: Man, top right: Man$_3$, bottom left: (Man)$_3$, bottom right: (Man$_3$)$_3$) as well as control conjugates C I, C II and C III (always left Cy5-channel, right Coomassie brilliant blue staining).

Table S 1: Summary of synthesized low valency conjugates with varying degrees of modification (DoM), number of TS equivalents used and Maldi-ToF-MS characterization.

Code	Conjugate	TS eq	MALDI-ToF-MS post glycosylation $[M+H]^+$ (*m/z*)	MALDI-ToF-MS post capping / final $[M+H]^+$ (*m/z*)	DoM: TS
**MI**	$[Man]_1$-HSA	1.2	75020	76340	1
**MII**	$[Man]_2$-HSA	2.3	75370	76681	2
**MIII**	$[Man]_3$-HSA	3.3	75815	77179	3
**MVI**	$[Man]_6$-HSA	6.3	77375	78061	6
**MIX**	$[Man]_9$-HSA	9.6	78485	78940	9
**TMI**	$[Man_3]_1$-HSA	3.3	75190	76648	1
**TMII**	$[Man_3]_2$-HSA	6.3	76163	77408	2
**TMIII**	$[Man_3]_3$-HSA	6.3	77100	78354	3
**TMVI**	$[Man_3]_6$-HSA	9.0	79086	79328	6
**TMIX**	$[Man_3]_9$-HSA	12.0	81503	81621	9
**MDI**	$[(Man)_3]_1$-HSA	2.5	76696	78893	1
**MDII**	$[(Man)_3]_2$-HSA	5.0	78220	79811	2
**MDIII**	$[(Man)_3]_3$-HSA	5.0	79910	81571	3
**TMDI**	$[(Man_3)_3]_1$-HSA	2.0	77087	78370	1
**TMDII**	$[(Man_3)_3]_2$-HSA	2.0	79471	79704	2
**TMDIII**	$[(Man_3)_3]_3$-HSA	4.0	82958	83200	3

Table S 2: Sizes (d. nm) and PDIs of low valency TS-HSA conjugates determined by DLS measurements (10–20 uM in MQ). Measurements were performed in triplicates; errors are given as standard deviation.

Conjugate	Size d. nm	PDI
**CI**	6.8 ± 0.3	0.703
**CII**	5.7 ± 0.2	0.844
**CIII**	4.8 ± 0.5	0.975
**MI**	3.6 ± 1.2	0.966
**MII**	4.2 ± 1.4	1.000
**MIII**	2.8 ± 0.4	0.926
**MVI**	4.6 ± 1.3	1.000
**MIX**	7.0 ± 1.0	0.827
**TMI**	9.6 ± 1.0	0.978
**TMII**	6.2 ± 1.1	0.754
**TMIII**	6.7 ± 0.4	0.855
**TMVI**	6.3 ± 1.0	0.752
**TMIX**	5.4 ± 0.2	0.848
**MDI**	8.0 ± 1.0	0.628
**MDII**	7.7 ± 1.1	0.916
**MDIII**	3.7 ± 0.6	0.816
**TMDI**	6.2 ± 0.2	0.691
**TMDII**	7.6 ± 0.1	0.829
**TMDIII**	6.8 ± 0.8	0.697

4.2.7 Preparation of saturated TS-HSA neoglycoalbumins

$DBCO_{47}$-HSA (0.3 mg, 3.24 nmol, 1 eq.) was dissolved in 50 mM phosphate buffer with 8 M urea and 2 mM EDTA (pH = 8) to a final protein concentration of 1 mg/mL. The azide functionalized TS (855 µmol, 264 eq. or 5.6 eq/DBCO) dissolved in MQ (10 mg/mL) was added and the reaction solution was inverted (10 rpm) at room temperature for 1 day. Afterwards, the reaction solution was purified (1 x reaction buffer, 5 x MQ) and concentrated via ultracentrifugation spin filter (Vivaspin, PES, 10 kDa). The product was lyophilized and obtained in the form of a blue lyophilizate quantitatively.

Table S 3: Summary table of synthesized conjugates of the saturated valency regime.

Code	Conjugate	MALDI-ToF-MS $[M+H]^+$ (*m/z*)	DoM: TS
**M_{sat}**	$[Man]_{43}$-HSA	112500	43
**TM_{sat}**	$[Man_3]_{45}$-HSA	127913	45
**MD_{sat}**	$[(Man)_3]_{42}$-HSA	169250	42
**TMD_{sat}**	$[(Man_3)_3]_{38}$-HSA	198400	38

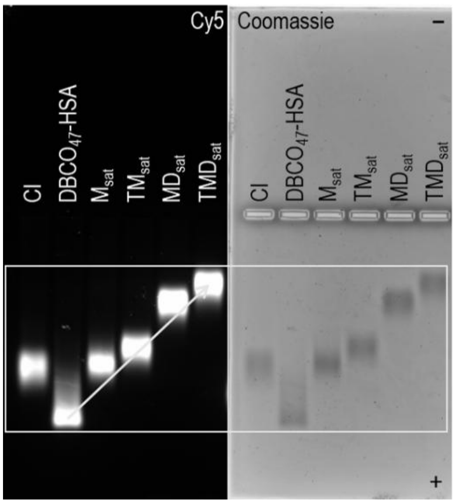

Figure S 2: Agarose gel electrophoresis of saturated NCs carrying different TSs (Number of Ts: Man: 43, Man_3: 45, $(Man)_3$: 42, $(Man_3)_3$: 38) as well as precursor conjugates $DBCO_{47}$-HSA and control conjugate C I (left Cy5-channel, right Coomassie brilliant blue staining).

Table S 4: Sizes (d. nm) and PDIs of saturated TS-HSA conjugates determined by DLS measurements (10–20 uM in MQ). Measurements were performed in triplicates; errors are given as standard deviation.

Conjugate	Size (d. nm)	PDI
**M_{sat}**	6.3 ± 1.3	0.581
**TM_{sat}**	6.8 ± 0.1	0.692
**MD_{sat}**	8.3 ± 0.1	0.570
**TMD_{sat}**	11.7 ± 0.8	0.697

5 Prediction of binding modes for monovalent TSs

TS Man (**7**) and Man_3 (**8**) with a truncated linker were docked into the primary carbohydrate-recognition site (CRD) of either CD209 (PDB: 1SL4)[49] or CRD4 of CD206 (PDB: 7JUF)[50] using SeeSAR 13.01.1.[51-53] To recreate experimentally observed mannose-binding modes for CD209, the template docking mode was employed, while for CD206, a constraint to include any oxygen was applied to two oxygen atoms (Figure S 3A). For each ligand, ten poses were generated, ranked by the estimated binding affinity,[53] and the highest-ranked pose was selected for binding mode analysis. The crystallographic binding mode of the reference ligand was visually compared to the predicted pose to assess pose prediction accuracy (Figure S 3B, C). The root mean square deviation (RMSD) values were calculated as 0.41 Å for CD206 and 1.27 Å for CD209.

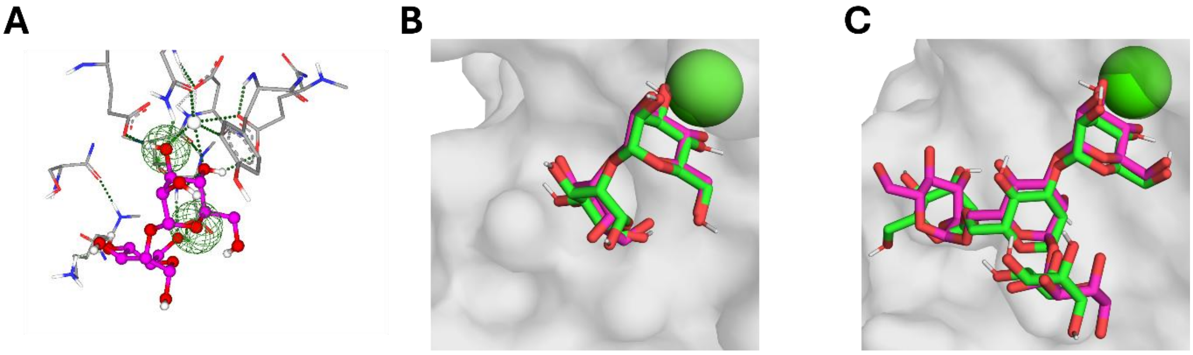

Figure S 3: **A)** Constraints (green spheres) put on reference ligand oxygen atoms for CD206 CRD4. **B)** Redocking the reference TSs for CD206 CRD4 (PDB: 7JUF, RMSD: 0.41 Å). **C)** Redocking the reference TSs for CD209 CRD (PDB: 1SL4, RMSD: 1.27 Å). The poses from the crystal structures are shown with pink carbon atoms, and the docking poses generated by SeeSAR 13.01.1 are shown with green carbon atoms. The protein surfaces are depicted in grey, and the Ca^{2+}-cation inside the binding pocket is represented as a green sphere.

6 NanoDSF binding study for mono- and trivalent TSs

6.1 Recombinant protein expression and purification.

6.1.1 Carbohydrate recognition domain 4 of CD206

CRD 4 of CD206 was expressed as described previously.[50] Briefly, a pT5T expression vector containing CRD4 of CD206 was transformed into competent *Escherichia coli* (E. coli) BL21 (DE3) cells. The cells were grown in LB medium containing 100 µg/mL ampicillin at 37 °C and 160 rpm until they reached an optical density (OD_{600}) of approximately 0.7. Overexpression was induced by adding 0.4 mM Isopropyl-β-D-thiogalactopyranoside (IPTG) for approximately 2.5 hours at 37 °C. Cells were harvested by centrifugation (10.000 × g at 4 °C for 15 minutes) and resuspended in cold buffer A (10 mM Tris-Cl, pH 7.8). Lysis was performed by thoroughly sonicating the cell pellets. Cell debris and inclusion bodies were collected by centrifugation (18,000 × g at 4 °C for 15 minutes), and the supernatant was discarded. The inclusion bodies were then solubilized in 100 mL of denaturing buffer B (6 M guanidine-HCl, 100 mM Tris-Cl, pH 7.0). After adding 2-mercaptoethanol (10 µL), the suspension was incubated at 4 °C on ice for 30 minutes. Insoluble impurities were removed by centrifugation (18,000 × g at 4 °C for 30 minutes). The supernatant was refolded by three-step dialysis against 2 L of buffer C (0.5 M NaCl, 25 mM Tris-Cl, pH 7.8, 25 mM $CaCl_2$) at 4 °C overnight. After centrifugation (18,000 × g at 4 °C for 30 minutes), the renatured CRD4 was purified using a 10 mL column of mannose-conjugated Sepharose prepared by divinyl sulfone coupling.[54] Finally, after washing with one column volume (CV) of buffer

D (150 mM NaCl, 25 mM Tris-Cl, pH 7.8, 25 mM $CaCl_2$), the bound protein was eluted with buffer E (150 mM NaCl, 25 mM Tris-Cl, pH 7.8, 2.5 mM EDTA).

6.1.2 Carbohydrate recognition domain of CD209

The recombinant CRD of CD209 was expressed as described previously.[55] Briefly, a pET-30 expression vector containing the CRD of CD209 was transformed into competent *Escherichia coli* (E. coli) BL21 (DE3) cells. The cells were grown in LB medium containing 100 µg/mL kanamycin at 37 °C and 160 rpm until they reached an optical density (OD_{600}) of approximately 0.7. Overexpression was induced by adding 0.5 mM IPTG for approximately 3 hours at 37 °C. Cells were harvested by centrifugation (10.000 × g at 4 °C for 15 minutes) and resuspended in cold buffer A (25 mM Tris-Cl, 150 mM NaCl, pH 8) supplemented with lysozyme, one tablet of protease inhibitor (cOmplete™, Roche), and DNase I. Lysis was performed by thoroughly sonicating the resuspended cell pellets. Cell debris and inclusion bodies were collected by centrifugation (20,000 × g at 4 °C for 1 hour), and the supernatant was discarded. The inclusion bodies were resuspended in buffer A supplemented with 2 M urea and 1% Triton X-100. After a second centrifugation step, the inclusion bodies were solubilized in buffer A supplemented with 6 M guanidine hydrochloride and 0.01 % 2-mercaptoethanol. Insoluble impurities were removed by centrifugation (20.000 × g at 4 °C for 1 hour). The resulting supernatant was dialyzed against 2 L of buffer B (25 mM Tris, pH 8) at 4 °C overnight. Next, refolding was performed by three-step dialysis against 2 L of buffer C (25 mM Tris-Cl, 150 mM NaCl, pH 8, 4 mM $CaCl_2$) at 4 °C overnight. After centrifugation (20.000 × g at 4 °C for 1 hour), the renatured CRD was purified using a HisTrap HP column pre-equilibrated with buffer D (150 mM NaCl, 25 mM Tris, pH 8, 4 mM $CaCl_2$, 10 mM imidazole). Unbound material was washed with 10 CV of buffer D, and the protein was eluted with buffer E (150 mM NaCl, 25 mM Tris, pH 8, 4 mM $CaCl_2$, 500 mM imidazole). Finally, functional protein was separated from non-functional protein by a 10 mL mannose-agarose column equilibrated with buffer C.

6.2 Differential Scanning Fluorimetry (NanoDSF).

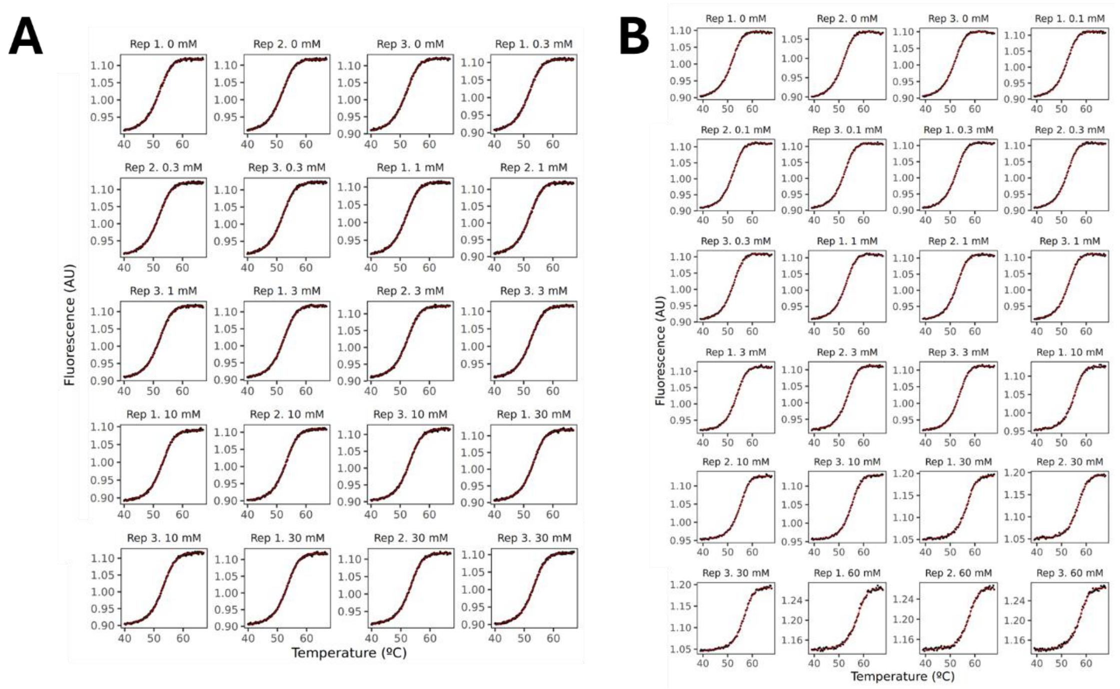

Figure S 4: **A)** Fitting of the fluorescence-based melting curves of protein CD206 CRD4 at different concentrations of the TS $ManN_3$. **B)** Fitting of the fluorescence-based melting curves of protein CD206 CRD4 at different concentrations of the TS Man_3N_3. Plot created by FoldAffinity (spc.embl-hamburg.de).[56]

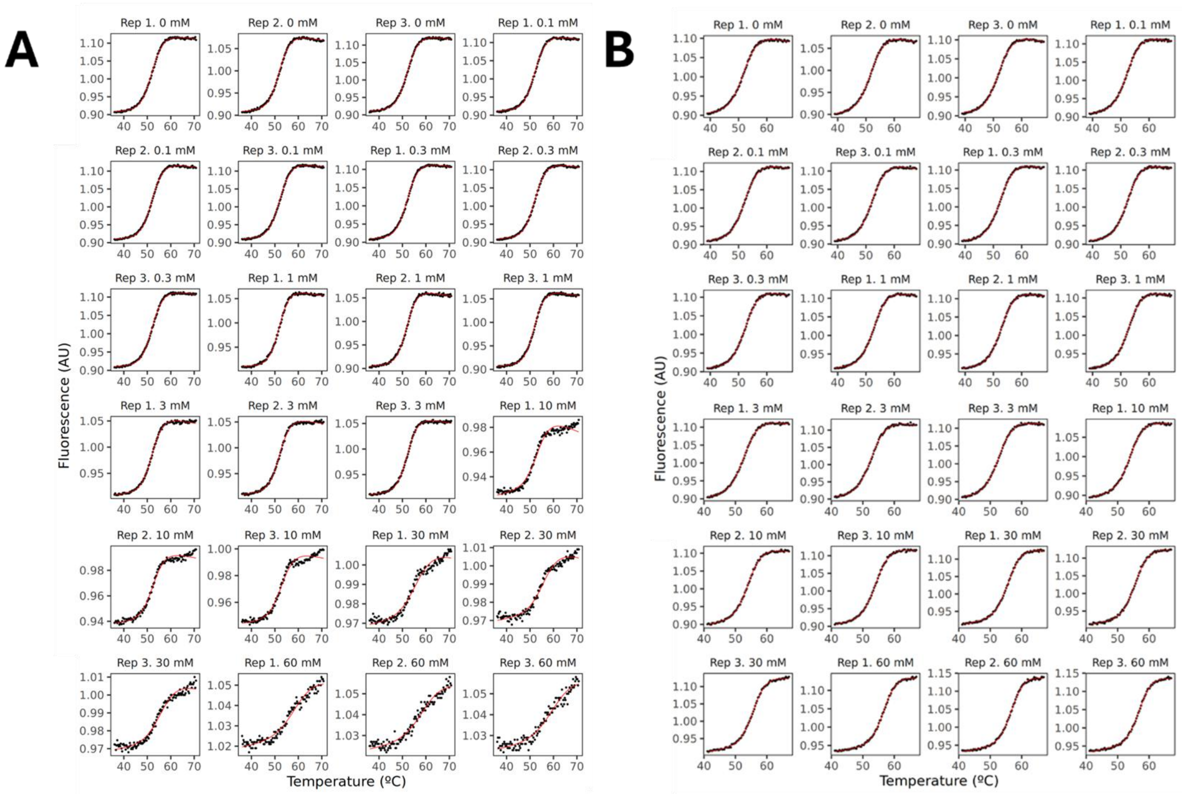

Figure S 5: **A)** Fitting of the fluorescence-based melting curves of protein CD206 CRD4 at different concentrations of the TS $(Man)_3N_3$. **B)** Fitting of the fluorescence-based melting curves of protein CD206 CRD4 at different concentrations of the TS $(Man_3)_3N_3$. Plot created by FoldAffinity (spc.embl-hamburg.de).[56]

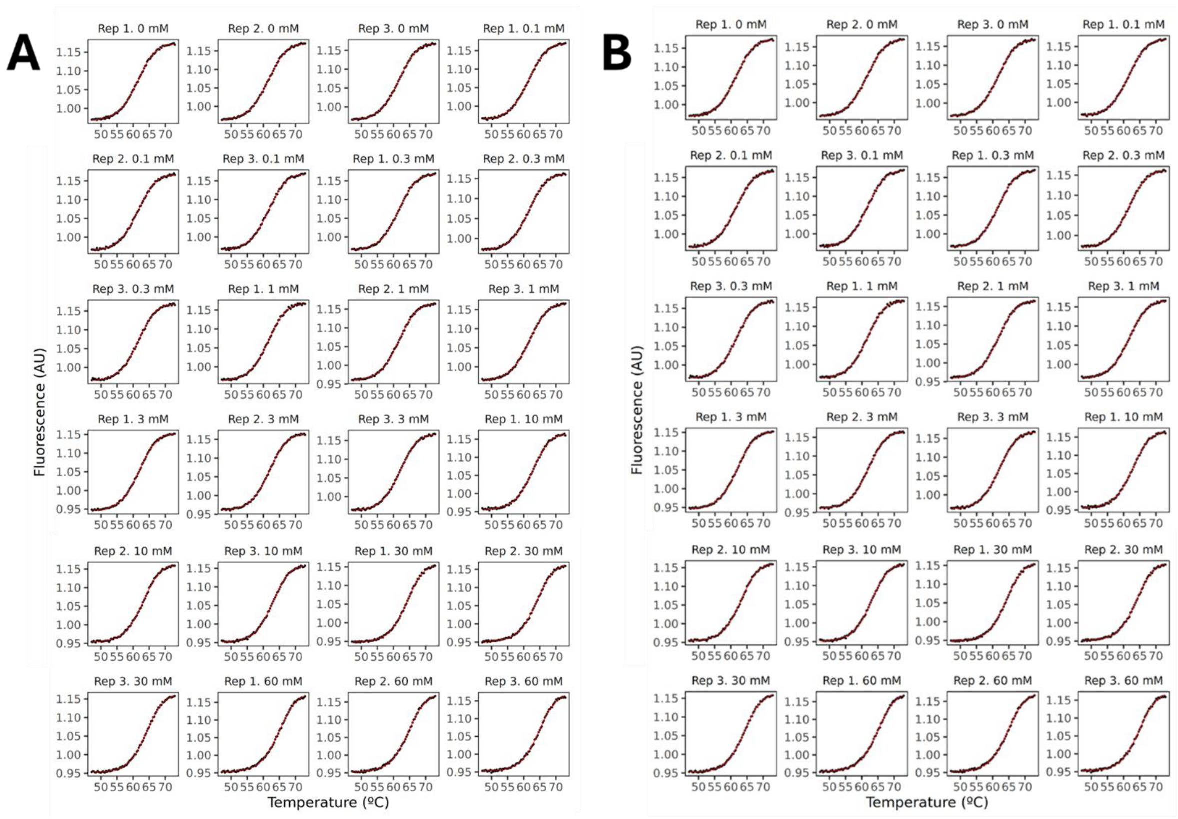

Figure S 6: **A)** Fitting of the fluorescence-based melting curves of protein CD209 CRD at different concentrations of the TS $ManN_3$. **B)** Fitting of the fluorescence-based melting curves of protein CD209 CRD at different concentrations of the TS Man_3N_3. Plot created by FoldAffinity (spc.embl-hamburg.de).[56]

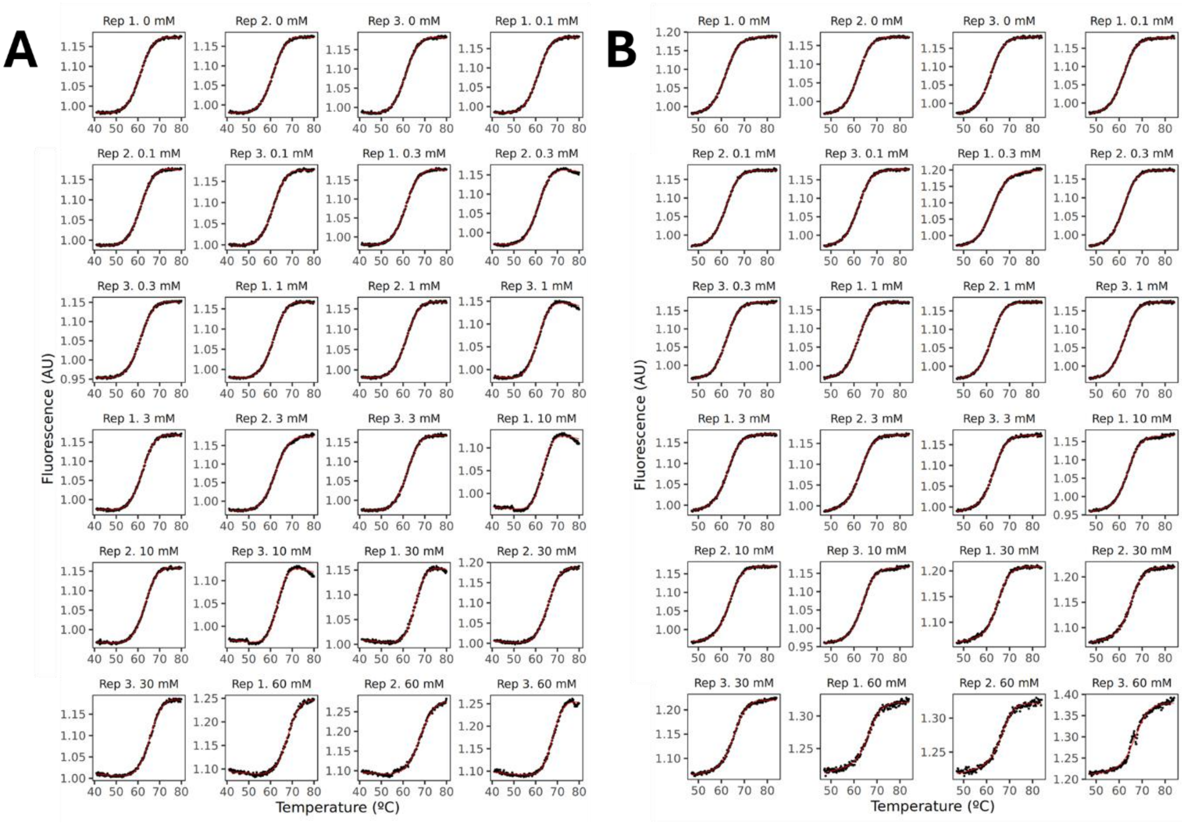

Figure S 7: **A)** Fitting of the fluorescence-based melting curves of protein CD209 CRD at different concentrations of the TS $(Man)_3N_3$. **B)** Fitting of the fluorescence-based melting curves of protein CD209 CRD at different concentrations of the TS $(Man_3)_3N_3$. Plot created by FoldAffinity (spc.embl-hamburg.de).[56]

7 Prediction of distances of mannose epitopes of a trivalent TS

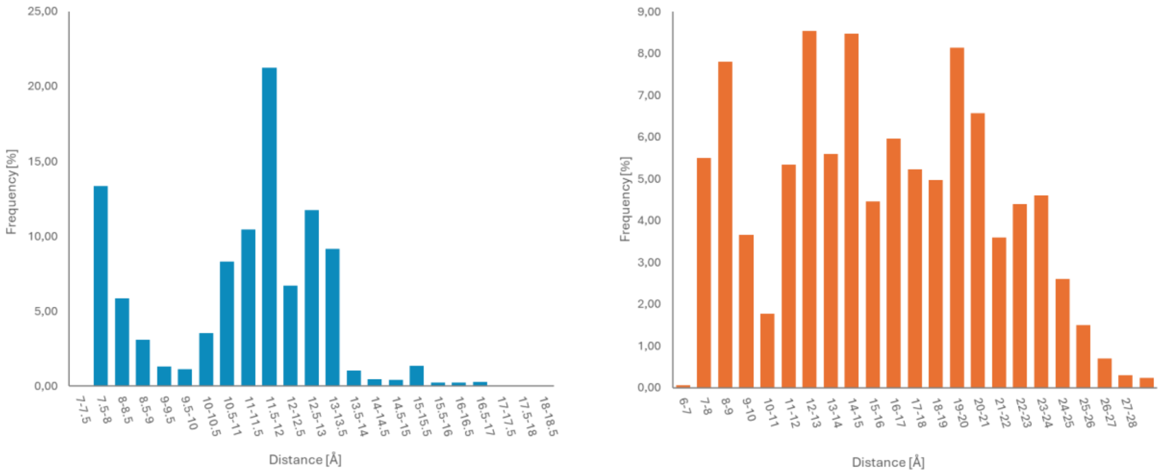

Figure S 8: Distribution of distances between one epitope and the focal point of the glycodendron measured between central carbon and hydroxy groups at *C*-4 (left). Distribution of distances spanned by two Man epitopes measured between hydroxy groups at *C*-4 (right).

8 Appendix

8.1 List of references

(1) Still, W. C.; Kahn, M.; Mitra, A. Rapid chromatographic technique for preparative separations with moderate resolution. *J. Org. Chem.* **1978**, *43* (14), 2923-2925. DOI: 10.1021/jo00408a041.

(2) Cavalli, E. S.; Mies, T.; Rzepa, H. S.; White, A. J. P.; Parsons, P. J.; Barrett, A. G. M. Pyrimidine Nucleosides Syntheses by Late-Stage Base Heterocyclization Reactions. *Org. Lett.* **2022**, *24* (49), 8931-8935. DOI: 10.1021/acs.orglett.2c03152.

(3) Kramer, J. R.; Deming, T. J. Glycopolypeptides via Living Polymerization of Glycosylated-l-lysine N-Carboxyanhydrides. *J. Am. Chem. Soc.* **2010**, *132* (42), 15068-15071. DOI: 10.1021/ja107425f.

(4) Ekholm, F. S.; Poláková, M.; Pawłowicz, A. J.; Leino, R. Synthesis of divalent 2, 2'-linked mannose derivatives by homodimerization. *Synth.* **2009**, *2009* (4), 567-576. DOI: 10.1055/s-0028-1083283.

(5) Nielsen, M. M.; Holmstrøm, T.; Pedersen, C. M. Stereoselective O-Glycosylations by Pyrylium Salt Organocatalysis. *Angew. Chem. Int. Ed.* **2022**, *61* (6), e202115394. DOI: 10.1002/anie.202115394.

(6) Kramer, S.; Langhanki, J.; Krumb, M.; Opatz, T.; Bros, M.; Zentel, R. HPMA-Based Nanocarriers for Effective Immune System Stimulation. *Macromol. Biosci.* **2019**, *19* (6), 1800481. DOI: 10.1002/mabi.201800481.

(7) León, E. I.; Martín, Á.; Pérez-Martín, I.; Quintanal, L. M.; Suárez, E. C–C Bond Formation by Sequential Intramolecular Hydrogen Atom Transfer/Intermolecular Radical Allylation Reaction in Carbohydrate Systems. *Eur. J. Org. Chem.* **2012**, *2012* (20), 3818-3829. DOI: 10.1002/ejoc.201200300.

(8) Schibilla, F.; Voskuhl, J.; Fokina, N. A.; Dahl, J. E. P.; Schreiner, P. R.; Ravoo, B. J. Host–Guest Complexes of Cyclodextrins and Nanodiamonds as a Strong Non-Covalent Binding Motif for Self-Assembled Nanomaterials. *Chem. Eur. J.* **2017**, *23* (63), 16059-16065. DOI: 10.1002/chem.201703392.

(9) Tanzi, L.; Robescu, M. S.; Marzatico, S.; Recca, T.; Zhang, Y.; Terreni, M.; Bavaro, T. Developing a Library of Mannose-Based Mono- and Disaccharides: A General Chemoenzymatic Approach to Monohydroxylated Building Blocks. *Molecules* **2020**, *25* (23), 5764. DOI: 10.3390/molecules25235764.

(10) Poláková, M.; Beláňová, M.; Mikušová, K.; Lattová, E.; Perreault, H. Synthesis of 1,2,3-Triazolo-Linked Octyl (1→6)-α-d-Oligomannosides and Their Evaluation in Mycobacterial Mannosyltransferase Assay. *Bioconjugate Chem.* **2011**, *22* (2), 289-298. DOI: 10.1021/bc100421g.

(11) Percec, V.; Leowanawat, P.; Sun, H.-J.; Kulikov, O.; Nusbaum, C. D.; Tran, T. M.; Bertin, A.; Wilson, D. A.; Peterca, M.; Zhang, S.; Heiney, P. A. Modular synthesis of amphiphilic Janus glycodendrimers and their self-assembly into glycodendrimersomes and other complex architectures with bioactivity to biomedically relevant lectins. *J. Am. Chem. Soc.* **2013**, *135* (24), 9055-9077. DOI: 10.1021/ja403323y.

(12) Reintjens, N. R. M.; Tondini, E.; Vis, C.; McGlinn, T.; Meeuwenoord, N. J.; Hogervorst, T. P.; Overkleeft, H. S.; Filippov, D. V.; van der Marel, G. A.; Ossendorp, F.; Codée, J. D. C. Multivalent, Stabilized Mannose-6-Phosphates for the Targeted Delivery of Toll-Like Receptor Ligands and Peptide Antigens. *ChemBioChem* **2021**, *22* (2), 434-440. DOI: 10.1002/cbic.202000538.

(13) Krabicová, I.; Dolenský, B.; Řezanka, M. Selectivity of 1-O-Propargyl-d-Mannose Preparations. *Molecules* **2022**, *27* (5), 1483. DOI: 10.3390/molecules27051483.

(14) Arias-Pérez, M.; Santos, M. An efficient approach to partially O-methylated α-D-mannopyranosides using bis-tert-butyldiphenylsilyl ethers as intermediates. *Tetrahedron* **1996**, *52* (32), 10785-10798. DOI: 10.1016/0040-4020(96)00600-X.

(15) Du, Y.; Zhang, M.; Kong, F. Highly efficient and practical synthesis of 3, 6-branched oligosaccharides. *Org. Lett.* **2000**, *2* (24), 3797-3800. DOI: 10.1021/ol000243w.

(16) Halmos, T.; Montserret, R.; Filippi, J.; Antonakis, K. Studies of the selective silylation of methyl α- and β-d-aldohexopyranosides: stability of the partially protected derivatives in polar solvents. *Carbohydr. Res.* **1987**, *170* (1), 57-69. DOI: 10.1016/0008-6215(87)85005-X.

(17) Mark, E.; Zbiral, E.; Brandstetter, H. H. Strukturelle Abwandlungen an partiell silylierten Kohlenhydraten mittels Triphenylphosphan/Azodicarbonsäurediethylester, 4. Mitt.: Transformationen an Mannose und Galaktose. *Monatsh. Chem.* **1980**, *111* (1), 289-307. DOI: 10.1007/BF00938735.

(18) Ramos-Soriano, J.; de la Fuente, M. C.; de la Cruz, N.; Figueiredo, R. C.; Rojo, J.; Reina, J. J. Straightforward synthesis of Man9, the relevant epitope of the high-mannose oligosaccharide. *Org. Biomol. Chem.* **2017**, *15* (42), 8877-8882. DOI: 10.1039/C7OB02286G.

(19) Teumelsan, N.; Huang, X. Synthesis of branched Man5 oligosaccharides and an unusual stereochemical observation. *J. Org. Chem.* **2007**, *72* (23), 8976-8979. DOI: 10.1021/jo7013824.

(20) Traboni, S.; Bedini, E.; Iadonisi, A. Orthogonal protection of saccharide polyols through solvent-free one-pot sequences based on regioselective silylations. *Beilstein J. Org. Chem.* **2016**, *12* (1), 2748-2756. DOI: 10.3762/bjoc.12.271.

(21) van den Bos, L. J.; Dinkelaar, J.; Overkleeft, H. S.; van der Marel, G. A. Stereocontrolled synthesis of β-D-mannuronic acid esters: synthesis of an alginate trisaccharide. *J. Am. Chem. Soc.* **2006**, *128* (40), 13066-13067. DOI: 10.1021/ja064787q.

(22) Zhang, Y.; Chen, C.; Jin, L.; Tan, H.; Wang, F.; Cao, H. Synthesis of unsymmetrical 3,6-branched Man5 oligosaccharide: a comparison between one-pot sequential glycosylation and stepwise synthesis. *Carbohydr. Res.* **2015**, *401*, 109-114. DOI: 10.1016/j.carres.2014.09.010.

(23) Kanaya, T.; Mashio, R.; Watanabe, T.; Schweizer, F.; Hada, N. Synthesis of glycosphingolipids from the fungus Hirsutella rhossiliensis. *Tetrahedron* **2017**, *73* (49), 6847-6855. DOI: 10.1016/j.tet.2017.10.034.

(24) Bakleh, M. E.; Sol, V.; Estieu-Gionnet, K.; Granet, R.; Déléris, G.; Krausz, P. An efficient route to VEGF-like peptide porphyrin conjugates via microwave-assisted 'click-chemistry'. *Tetrahedron* **2009**, *65* (36), 7385-7392. DOI: 10.1016/j.tet.2009.07.028.

(25) Davila, J.; Chassepot, A.; Longo, J.; Boulmedais, F.; Reisch, A.; Frisch, B.; Meyer, F.; Voegel, J.-C.; Mésini, P. J.; Senger, B.; Metz-Boutigue, M.-H.; Hemmerlé, J.; Lavalle, P.; Schaaf, P.; Jierry, L. Cyto-mechanoresponsive Polyelectrolyte Multilayer Films. *J. Am. Chem. Soc.* **2012**, *134* (1), 83-86. DOI: 10.1021/ja208970b.

(26) Krumb, M.; Frey, M.-L.; Langhanki, J.; Forster, R.; Kowalczyk, D.; Mailänder, V.; Landfester, K.; Opatz, T. Multivalency Beats Complexity: A Study on the Cell Uptake of Carbohydrate Functionalized Nanocarriers to Dendritic Cells. *Cells* **2020**, *9* (9), 2087. DOI: 10.3390/cells9092087.

(27) Tavernaro, I.; Hartmann, S.; Sommer, L.; Hausmann, H.; Rohner, C.; Ruehl, M.; Hoffmann-Roeder, A.; Schlecht, S. Synthesis of tumor-associated MUC1-glycopeptides and their multivalent presentation by functionalized gold colloids. *Org. Biomol. Chem.* **2015**, *13* (1), 81-97, 10.1039/C4OB01339E. DOI: 10.1039/C4OB01339E.

(28) Tsakama, M.; Shang, Y.; He, Y.; Fan, B.; Wang, F.; Chen, W.; Dai, X. Synthesis and optical properties of a novel sugar coated poly(p-phenyleneethynylene) effectively quenched by concanavalin A. *Tetrahedron Lett.* **2016**, *57* (16), 1739-1742. DOI: 10.1016/j.tetlet.2016.01.102.

(29) Landeros, J. M.; Silvestre, H. A.; Guadarrama, P. Synthesis of branched cores by poly-O-alkylation reaction under phase transfer conditions. A systematic study. *J. Mol. Struct.* **2013**, *1037*, 412-419. DOI: 10.1016/j.molstruc.2013.01.034.

(30) Newkome, G. R.; Lin, X. Symmetrical, four-directional, poly (ether-amide) cascade polymers. *Macromolecules* **1991**, *24* (6), 1443-1444. DOI: 10.1021/ma00006a042.

(31) Appel, R.; Fuchs, J.; Tyrrell, S. M.; Korevaar, P. A.; Stuart, M. C.; Voets, I. K.; Schonhoff, M.; Besenius, P. Steric Constraints Induced Frustrated Growth of Supramolecular Nanorods in Water. *Chemistry* **2015**, *21* (52), 19257-19264. DOI: 10.1002/chem.201503616.

(32) Cardona, C. M.; Gawley, R. E. An improved synthesis of a trifurcated newkome-type monomer and orthogonally protected two-generation dendrons. *J. Org. Chem.* **2002**, *67* (4), 1411-1413. DOI: 10.1021/jo0161678.

(33) Baldoli, C.; Rigamonti, C.; Maiorana, S.; Licandro, E.; Falciola, L.; Mussini, P. R. A New Triferrocenyl-tris(hydroxymethyl)aminomethane Derivative as a Highly Sensitive Electrochemical Marker of Biomolecules: Application to the Labelling of PNA Monomers and Their Electrochemical Characterization. *Chem. Eur. J.* **2006**, *12* (15), 4091-4100. DOI: 10.1002/chem.200501466.

(34) Odaka, M.; Furuta, T.; Kobayashi, Y.; Iwamura, M. Synthesis, Photoreactivity and Cytotoxic Activity of Caged Compounds of L-Leucyl-L-Leucine Methyl Ester, an Apoptosis Inducer. *Photochem. Photobiol.* **1996**, *63* (6), 800-806. DOI: 10.1111/j.1751-1097.1996.tb09633.x.

(35) Spitzer, D.; Rodrigues, L. L.; Straßburger, D.; Mezger, M.; Besenius, P. Tuneable Transient Thermogels Mediated by a pH- and Redox-Regulated Supramolecular Polymerization. *Angew. Chem. Int. Ed.* **2017**, *56* (48), 15461-15465. DOI: 10.1002/anie.201708857.

(36) André, S.; Cañada, F. J.; Shiao, T. C.; Largartera, L.; Diercks, T.; Bergeron-Brlek, M.; el Biari, K.; Papadopoulos, A.; Ribeiro, J. P.; Touaibia, M.; Solís, D.; Menéndez, M.; Jiménez-Barbero, J.; Roy, R.; Gabius, H.-J. Fluorinated Carbohydrates as Lectin Ligands: Biorelevant Sensors with Capacity to Monitor Anomer Affinity in 19F-NMR-Based Inhibitor Screening. *Eur. J. Org. Chem.* **2012**, *2012* (23), 4354-4364. DOI: 10.1002/ejoc.201200397.

(37) Hellmuth, I.; Freund, I.; Schlöder, J.; Seidu-Larry, S.; Thüring, K.; Slama, K.; Langhanki, J.; Kaloyanova, S.; Eigenbrod, T.; Krumb, M.; Röhm, S.; Peneva, K.; Opatz, T.; Jonuleit, H.; Dalpke, A. H.; Helm, M. Bioconjugation of Small Molecules to RNA Impedes Its Recognition by Toll-Like Receptor 7. *Front. Immunol.* **2017**, *8*, Original Research. DOI: 10.3389/fimmu.2017.00312.

(38) Lu, W.-Y.; Sun, X.-W.; Zhu, C.; Xu, J.-H.; Lin, G.-Q. Expanding the application scope of glycosidases using click chemistry. *Tetrahedron* **2010**, *66* (3), 750-757. DOI: 10.1016/j.tet.2009.11.044.

(39) Yamamoto, S.; Nakahama, S.; Yamaguchi, K. A Heterobifunctional Linker Bearing Azide-reactive Alkyne and Thiol-reactive Maleimide Connected with N-(2-Nitrobenzyl)imide to Synthesize Photocleavable Diblock Copolymers. *Chem. Lett.* **2013**, *42* (8), 791-793. DOI: 10.1246/cl.130235 (acccessed 2/11/2025).

(40) Lindhorst, T. K.; Bruegge, K.; Fuchs, A.; Sperling, O. A bivalent glycopeptide to target two putative carbohydrate binding sites on FimH. *Beilstein J. Org. Chem.* **2010**, *6*, 801-809. DOI: 10.3762/bjoc.6.90.

(41) Reina, J. J.; Rioboo, A.; Montenegro, J. Glycosyl Aldehydes: New Scaffolds for the Synthesis of Neoglycoconjugates via Bioorthogonal Oxime Bond Formation. *Synth.* **2018**, *50* (04), 831-845. DOI: 10.1055/s-0036-1591082.

(42) Amit, B.; Hazum, E.; Fridkin, M.; Patchornik, A. A photolabile protecting group for the phenolic hydroxyl function of tyrosine. *Int. J. Pept. Protein Res.* **1977**, *9* (2), 91-96. DOI: 10.1111/j.1399-3011.1977.tb03468.x.

(43) Amit, B.; Zehavi, U.; Patchornik, A. Photosensitive protecting groups of amino sugars and their use in glycoside synthesis. 2-Nitrobenzyloxycarbonylamino and 6-nitroveratryloxycarbonylamino derivatives. *J. Org. Chem.* **1974**, *39* (2), 192-196. DOI: 10.1021/jo00916a015.

(44) Cheng, S.; Feng, Y.; Li, W.; Liu, T.; Lv, X.; Tong, X.; Xi, G.; Ye, X.; Li, X. Development of novel antivrial agents that induce the degradation of the main protease of human-infecting coronaviruses. *Eur. J. Med. Chem.* **2024**, *275*, 116629. DOI: 10.1016/j.ejmech.2024.116629.

(45) Krumb, M.; Lucas, T.; Opatz, T. Visible Light Enables Aerobic Iodine Catalyzed Glycosylation. *Eur. J. Org. Chem.* **2019**, *2019* (28), 4517-4521. DOI: 10.1002/ejoc.201900143.

(46) Chakraborti, A. K.; Gulhane, R. Perchloric acid adsorbed on silica gel as a new, highly efficient, and versatile catalyst for acetylation of phenols, thiols, alcohols, and amines. *Chem. Commun.* **2003**, (15), 1896-1897. DOI: 10.1039/B304178F.

(47) Dyer, R. G.; Turnbull, K. D. Hydrolytic Stabilization of Protected p-Hydroxybenzyl Halides Designed as Latent Quinone Methide Precursors. *J. Org. Chem.* **1999**, *64* (21), 7988-7995. DOI: 10.1021/jo991085t.

(48) Zhou, K.; Li, G.; Pan, R.; Xin, S.; Wen, W.; Wang, H.; Luo, C.; Han, R. P. S.; Gu, Y.; Tu, Y. Preclinical evaluation of AGTR1-Targeting molecular probe for colorectal cancer imaging in orthotopic and liver metastasis mouse models. *Eur. J. Med. Chem.* **2024**, *271*, 116452. DOI: 10.1016/j.ejmech.2024.116452.

(49) Guo, Y.; Feinberg, H.; Conroy, E.; Mitchell, D. A.; Alvarez, R.; Blixt, O.; Taylor, M. E.; Weis, W. I.; Drickamer, K. Structural basis for distinct ligand-binding and targeting properties of the receptors DC-SIGN and DC-SIGNR. *Nat. Struct. Mol. Biol.* **2004**, *11* (7), 591-598. DOI: 10.1038/nsmb784.

(50) Feinberg, H.; Jégouzo, S. A.; Lasanajak, Y.; Smith, D. F.; Drickamer, K.; Weis, W. I.; Taylor, M. E. Structural analysis of carbohydrate binding by the macrophage mannose receptor CD206. *J. Biol. Chem.* **2021**, *296*, 100368. DOI: 10.1016/j.jbc.2021.100368

(51) SeeSAR, version 13.01.1 BioSolveIT GmbH, St. Augustin, Germany, https://www.biosolveit.de/SeeSAR.

(52) Rarey, M.; Kramer, B.; Lengauer, T.; Klebe, G. A Fast Flexible Docking Method using an Incremental Construction Algorithm. *J. Mol. Biol.* **1996**, *261* (3), 470-489. DOI: 10.1006/jmbi.1996.0477.

(53) Reulecke, I.; Lange, G.; Albrecht, J.; Klein, R.; Rarey, M. Towards an Integrated Description of Hydrogen Bonding and Dehydration: Decreasing False Positives in Virtual Screening with the HYDE Scoring Function. *ChemMedChem* **2008**, *3* (6), 885-897. DOI: 10.1002/cmdc.200700319.

(54) Fornstedt, N.; Porath, J. Characterization studies on a new lectin found in seeds of Vicia ervilia. *FEBS Lett.* **1975**, *57* (2), 187-191. DOI: 10.1016/0014-5793(75)80713-7.

(55) Thépaut, M.; Guzzi, C.; Sutkeviciute, I.; Sattin, S.; Ribeiro-Viana, R.; Varga, N.; Chabrol, E.; Rojo, J.; Bernardi, A.; Angulo, J.; Nieto, P. M.; Fieschi, F. Structure of a Glycomimetic Ligand in the Carbohydrate Recognition Domain of C-type Lectin DC-SIGN. Structural Requirements for Selectivity and Ligand Design. *J. Am. Chem. Soc.* **2013**, *135* (7), 2518-2529. DOI: 10.1021/ja3053305.

(56) Bai, N.; Roder, H.; Dickson, A.; Karanicolas, J. Isothermal Analysis of ThermoFluor Data can readily provide Quantitative Binding Affinities. *Sci. Rep.* **2019**, *9* (1), 2650. DOI: 10.1038/s41598-018-37072-x.

8.2 List of abbreviations

$(HF)_X$·Pyr	Hydrogen fluoride pyridine complex
Ac_2O	Acetic anhydride
AgOTf	Silver triflate
Ar-atm.	Argon atmosphere
ATR	Attenuated total reflection
BF_3·OEt_2	Boron trifluoride diethyl etherate
BzCl	Benzoyl chloride
C_{18}	C_{18}-alkyl chains modified silica for RP chromatography
CDXX	Cluster of differentiation (number = XX)
CD_3CN	Deuterated Acetonitrile
CD_3OD	Deuterated methanol
$CDCl_3$	Deuterated Chloroform
^{c}Hex	Cyclohexane
COSY	Correlated spectroscopy
CRD	Carbohydrate recognition domain
CuBr	Copper(I)bromide
Cy5	Cyanine 5 dye
DC	Dendritic cell
D_2O	Deuterium oxide
DAD	Diode array detector
DBCO	Dibenzocyclooctyne
DCM	Dichloromethane
DIPEA	*N,N*-Diisopropylethylamine
DMAP	*N,N*-Dimethylpyridin-4-amine
DMF	Dimethylformamide
DMSO	Dimethyl sulfoxide
DoM	Degree of modification
EDTA	Ethylenediaminetetraacetic acid
ELS	Evaporative light scattering
Eq.	Equivalent
ESI	Electrospray ionization
Et_2O	Diethylether
EtOAc	Ethyl acetate
EtOH	Ethanol
FCC	Flash column chromatography
H_2O (MQ)	Ultrapure water
H_2SO_4	Sulfuric acid
HCl	Hydrochloric acid
HCOOH	Formic acid
HMBC	Heteronuclear multiple bond correlation
HOPrg	Propargyl alcohol
HPLC	High performance liquid chromatography
HR	High resolution
HSA	Human serum albumin
HSQC	Heteronuclear Single Quantum Coherence
HSQC-NoDec	HSQC without decoupling
I_2	Molecular iodine
ICAM-3	Intercellular adhesion molecule 3 (CD50)
IR	Infra-red
$KMnO_4$	Potassium permanganate
M	Molar mass
MALDI-ToF-MS	Matrix assisted laser desorption ionization with time-of-flight MS
MeOH	Methanol
MS	Mass spectrometry
MsCl	Mesylchloride
MS 3 Å	Molecular sieves (Pore size 3 Å)
$Na_2S_2O_3$	Sodium thiosulfate
Na_2SO_4	Sodium sulfate
NaCl	Sodium chloride
$NaHCO_3$	Sodium bicarbonate
NaN_3	Sodium azide
NanoDSF	Nano differential scanning fluorimetry
NaOH	Sodium hydroxide
NaOMe	Sodium methoxide
NEt_3	Triethylamine
NHS	*N*-Hydroxysuccinimide

NIS	*N*-Iodosuccinimide
NMR	Nuclear magnetic resonance
NOESY	Nuclear Overhauser enhancement spectroscopy
NP	Normal phase chromatography
OVA	Ovalbumin
PEG	Polyethylene glycol
pH	Potentia hydrogenii
PhSH	Thiophenol
PMDTA	N^1-[2-(Dimethylamino)ethyl]-N^1,N^2,N^2-trimethylethane-1,2-diamine
Pyr.	Pyridine
R_f	Ratio of fronts
RP	Reversed phase chromate-graphy
TBAI	Tetrabutylammonium iodide
TBDMS	*tert*-Butyldimethylsilyl
TFA	Trifluoromethanesulfonic acid
THF	Tetrahydrofuran
TLC	Thin layer chromatography
TM	Trimannose
TOCSY	Total correlation spectroscopy
t_R	Retention time
TRIS	Tris(hydroxymethyl)amin
TS	Targeting structure
TsCl	Tosyl chloride

8.3 NMR spectra

¹H-NMR, (400 MHz, $CDCl_3$): **(I1)**

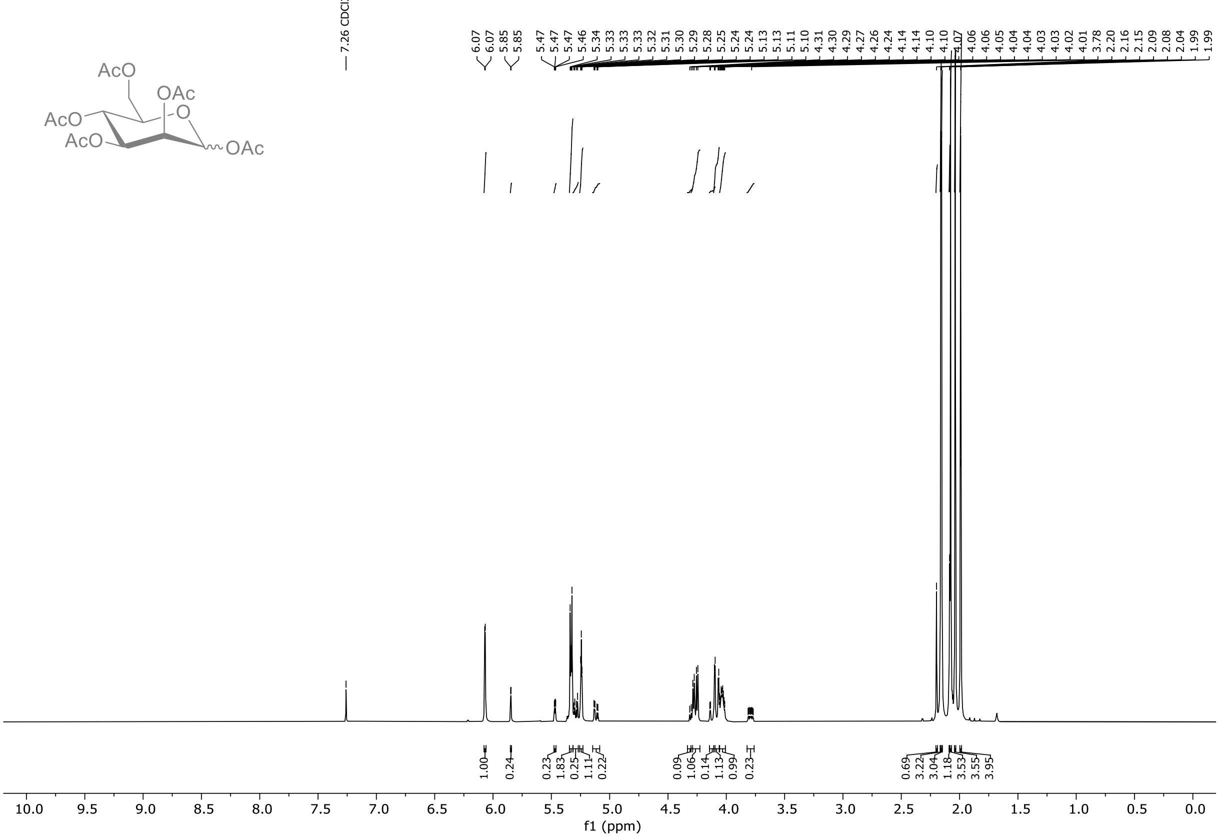

¹³C-NMR, (101 MHz, $CDCl_3$): **(I1)**

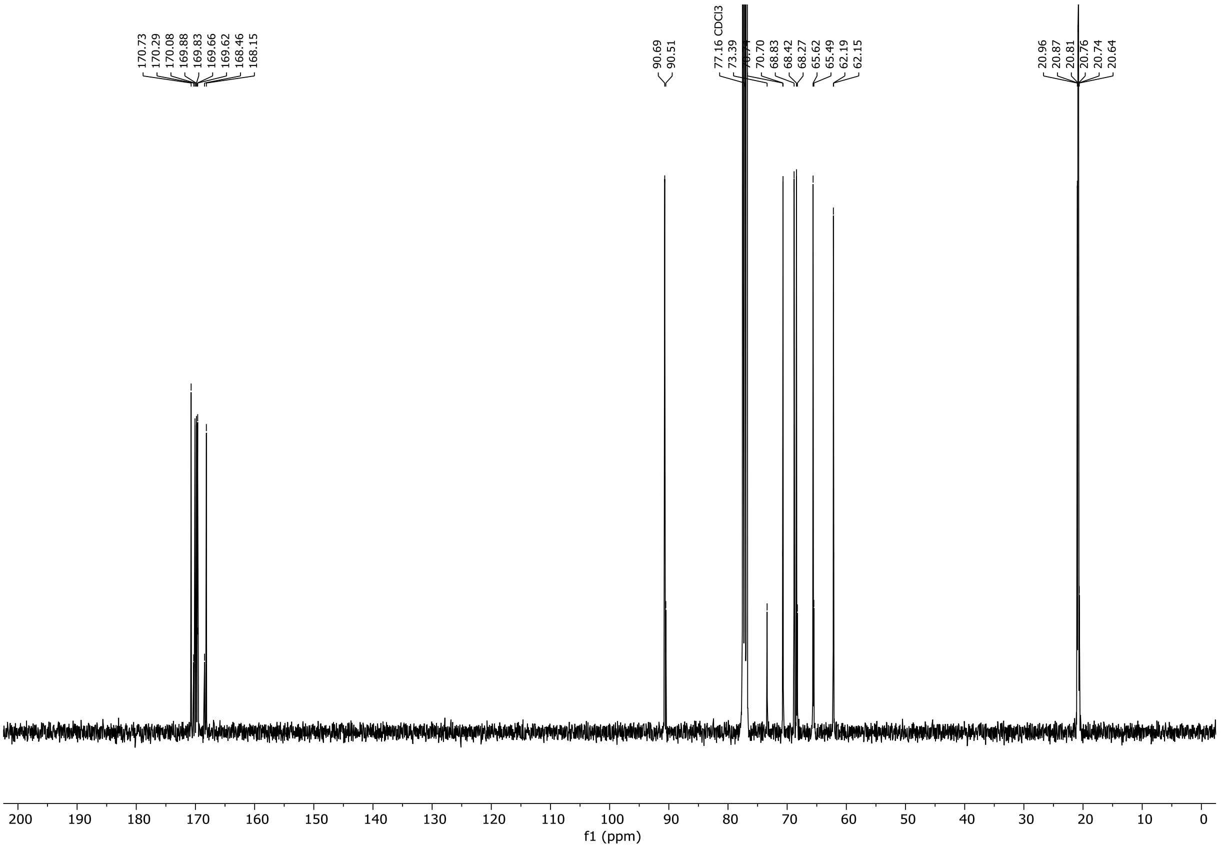

^{1}H-NMR, (400 MHz, $CDCl_3$): **(S1)**

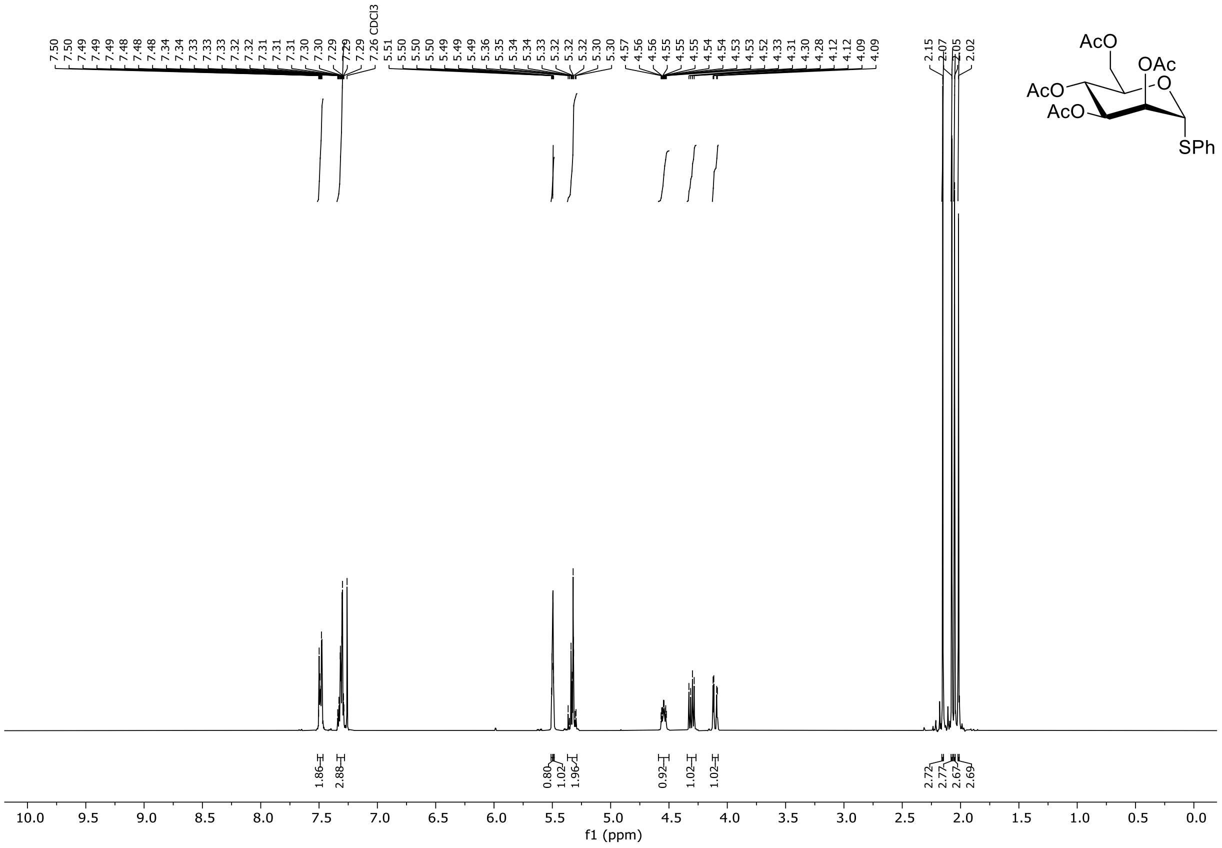

^{13}C-NMR, (101 MHz, $CDCl_3$): **(S1)**

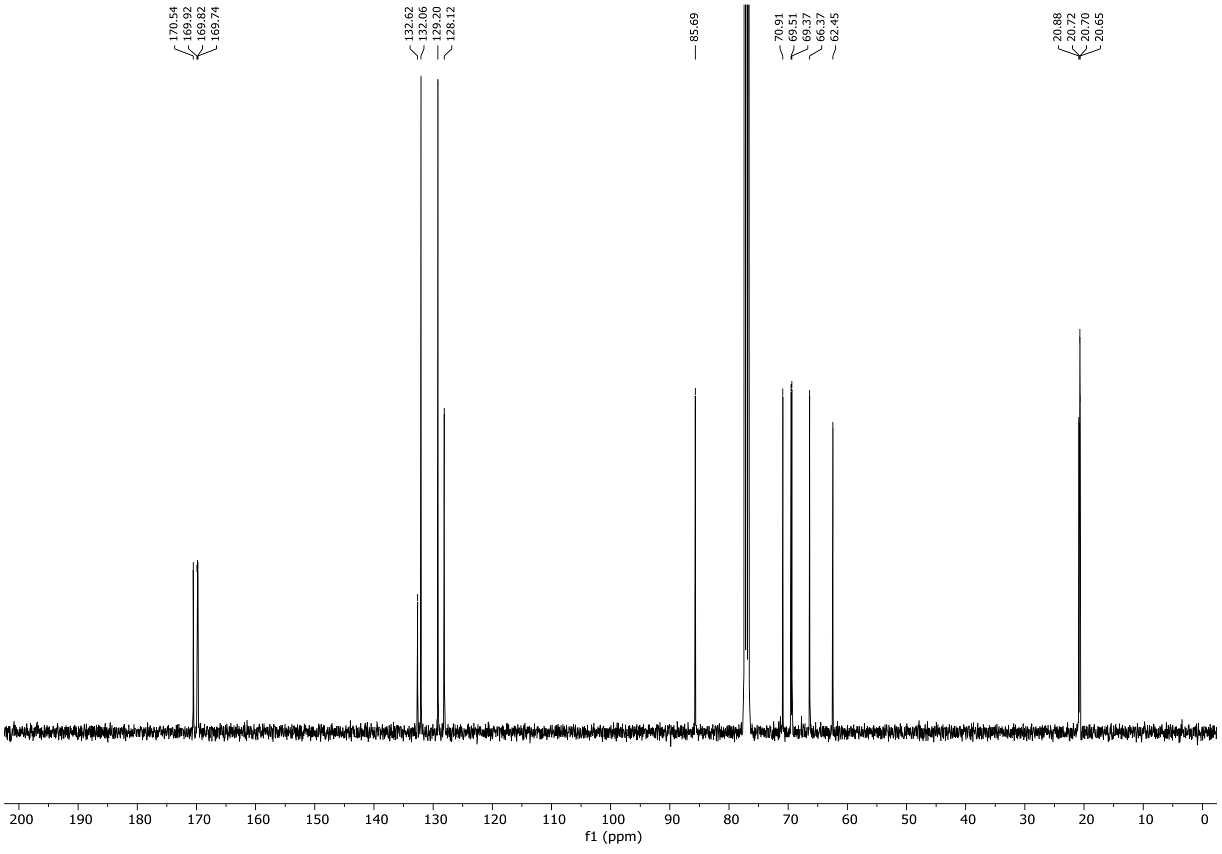

^{1}H-NMR, (400 MHz, $CDCl_3$): (**S7**)

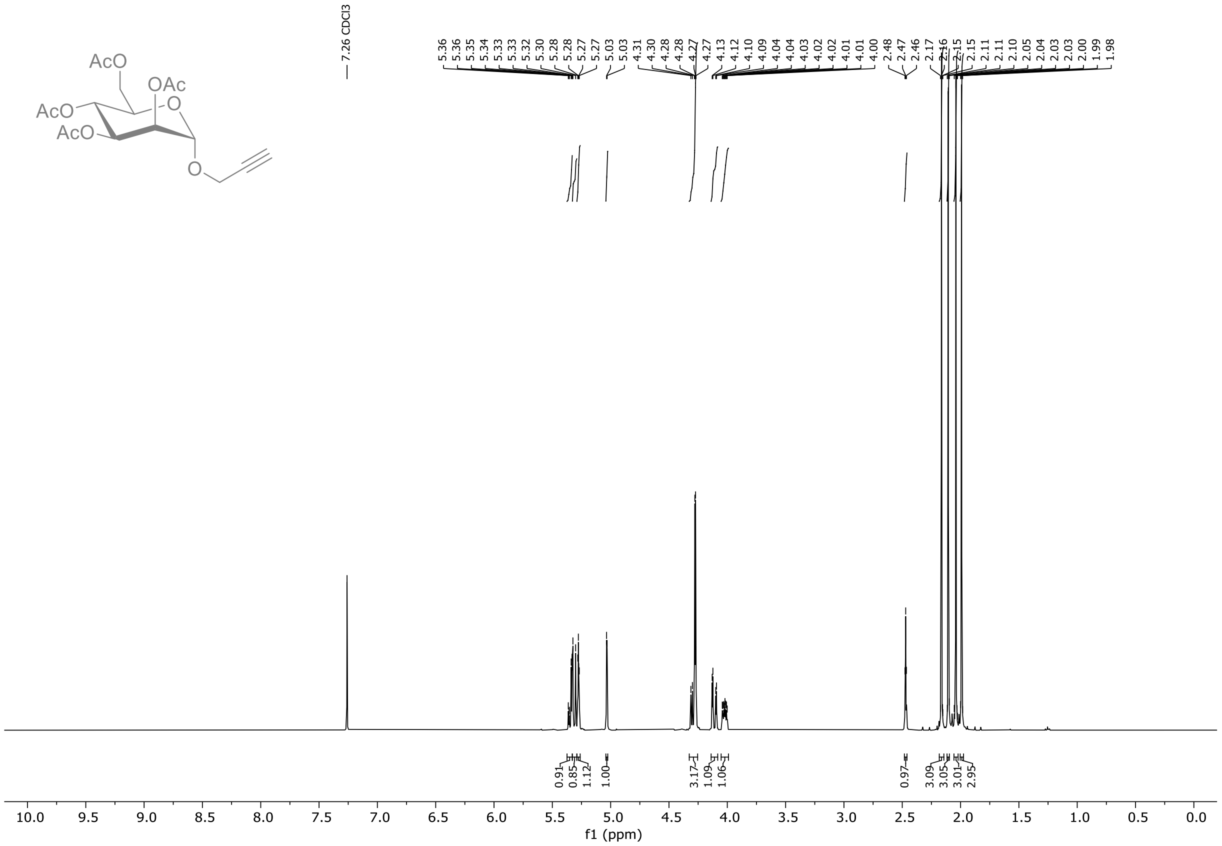

^{13}C-NMR, (101 MHz, $CDCl_3$): (**S7**)

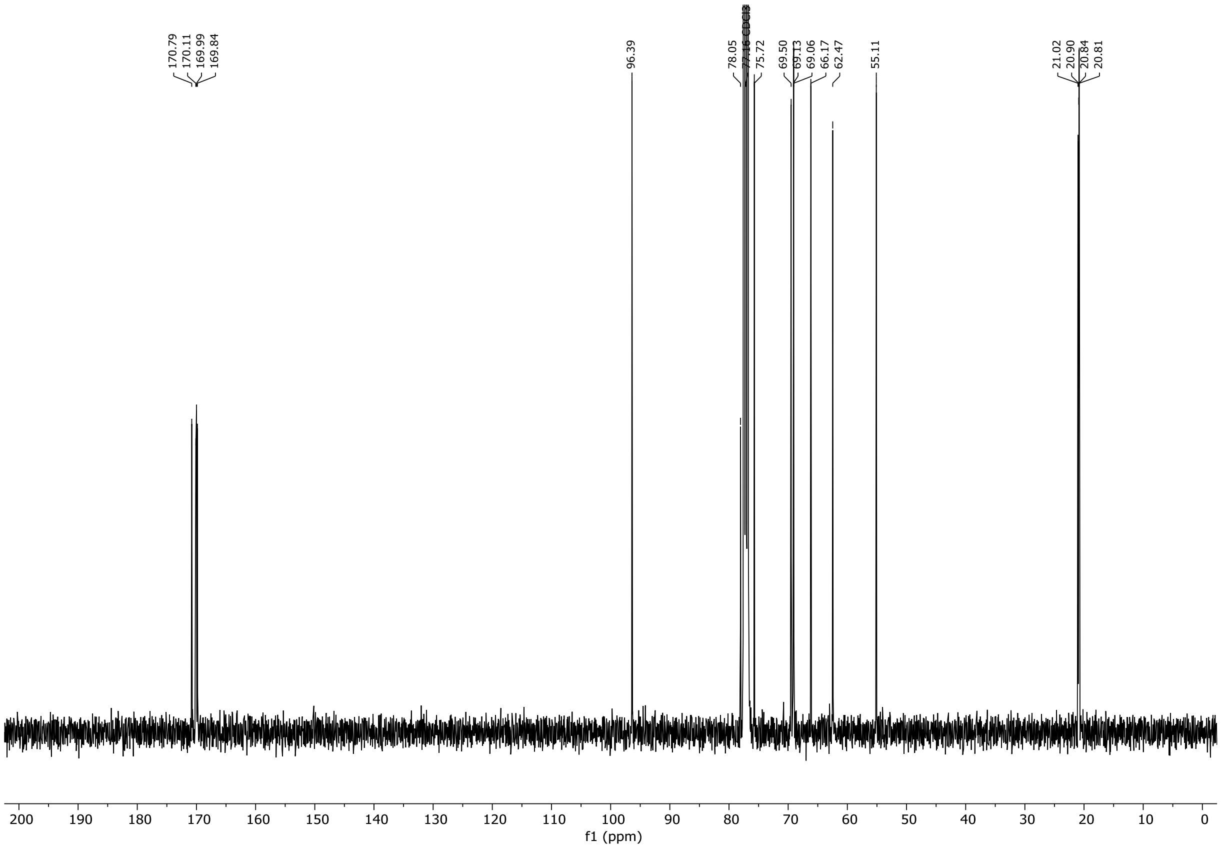

^{1}H-NMR, (400 MHz, CD_3OD): (**1**)

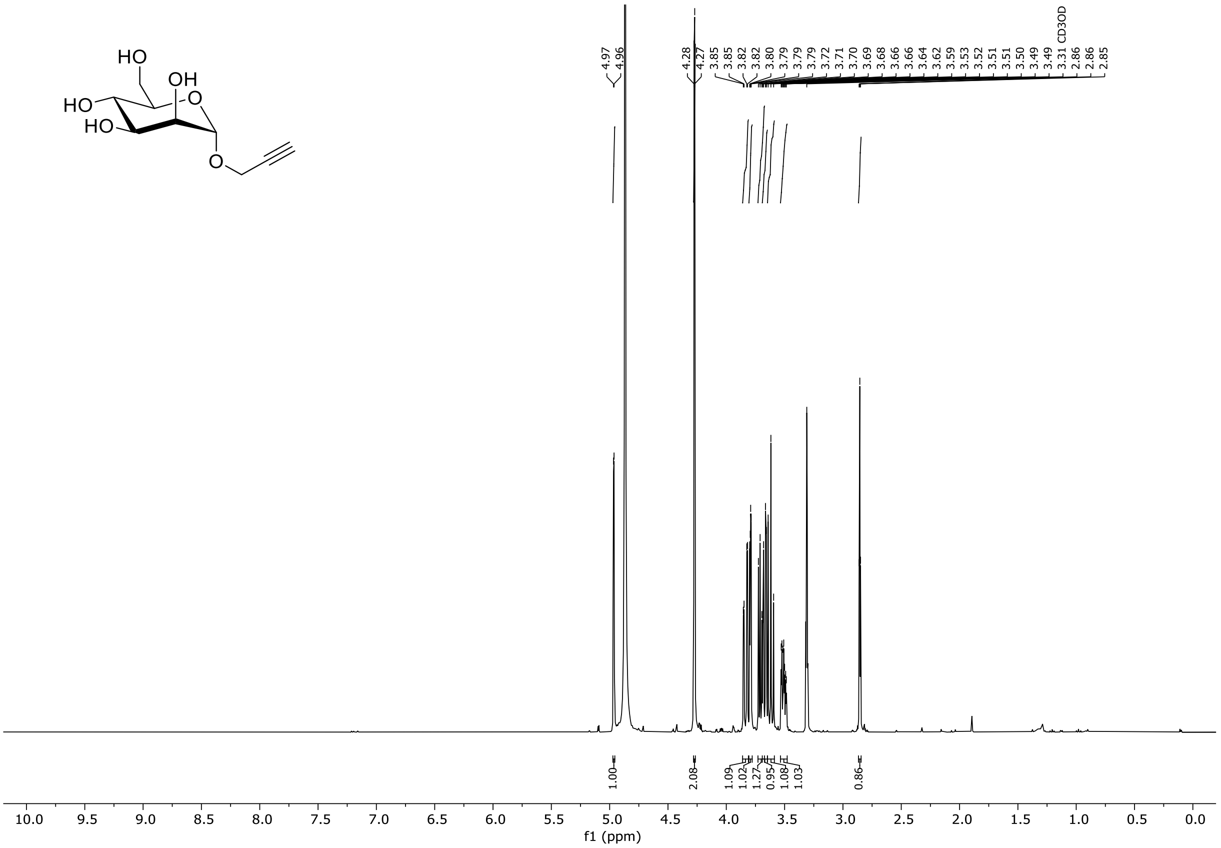

^{13}C-NMR, (101 MHz, CD_3OD): (**1**)

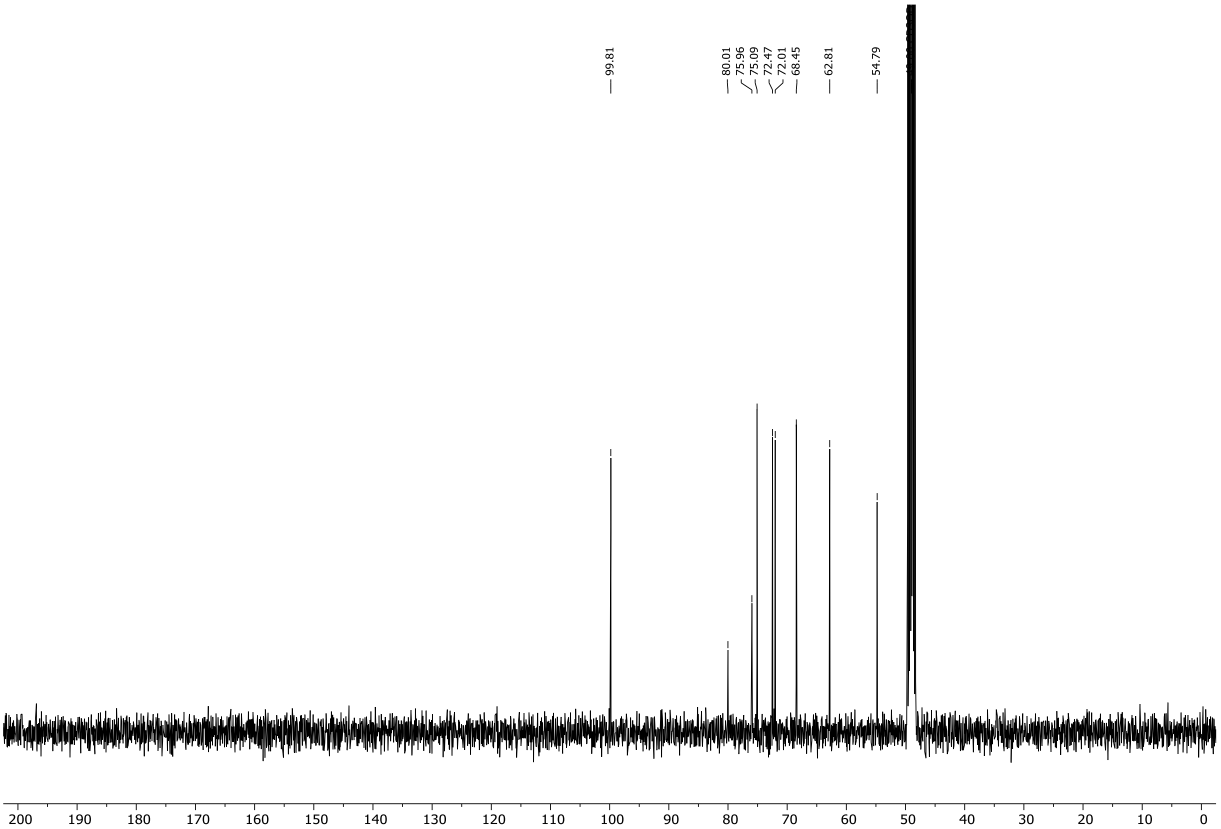

^{1}H-NMR, (400 MHz, $CDCl_3$): (**I2**)

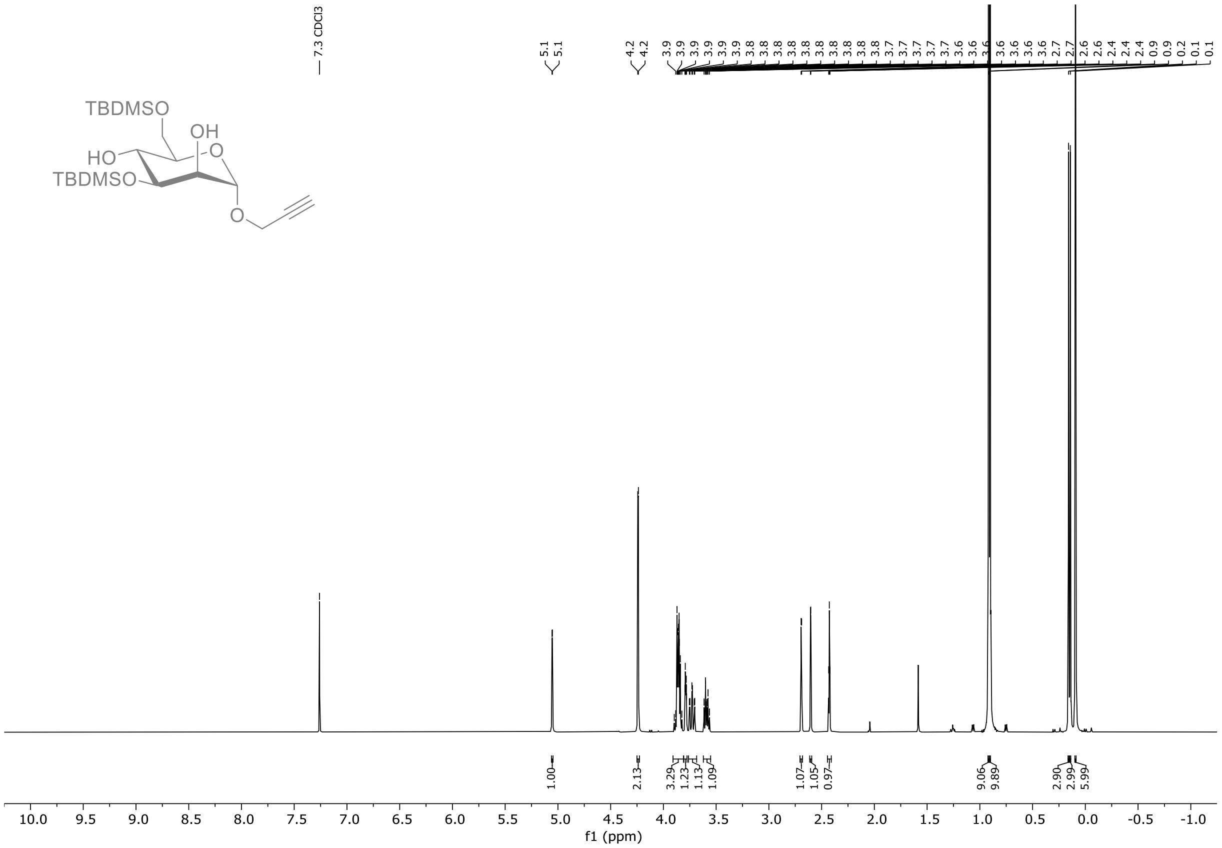

^{13}C-NMR, (101 MHz, $CDCl_3$): (**I2**)

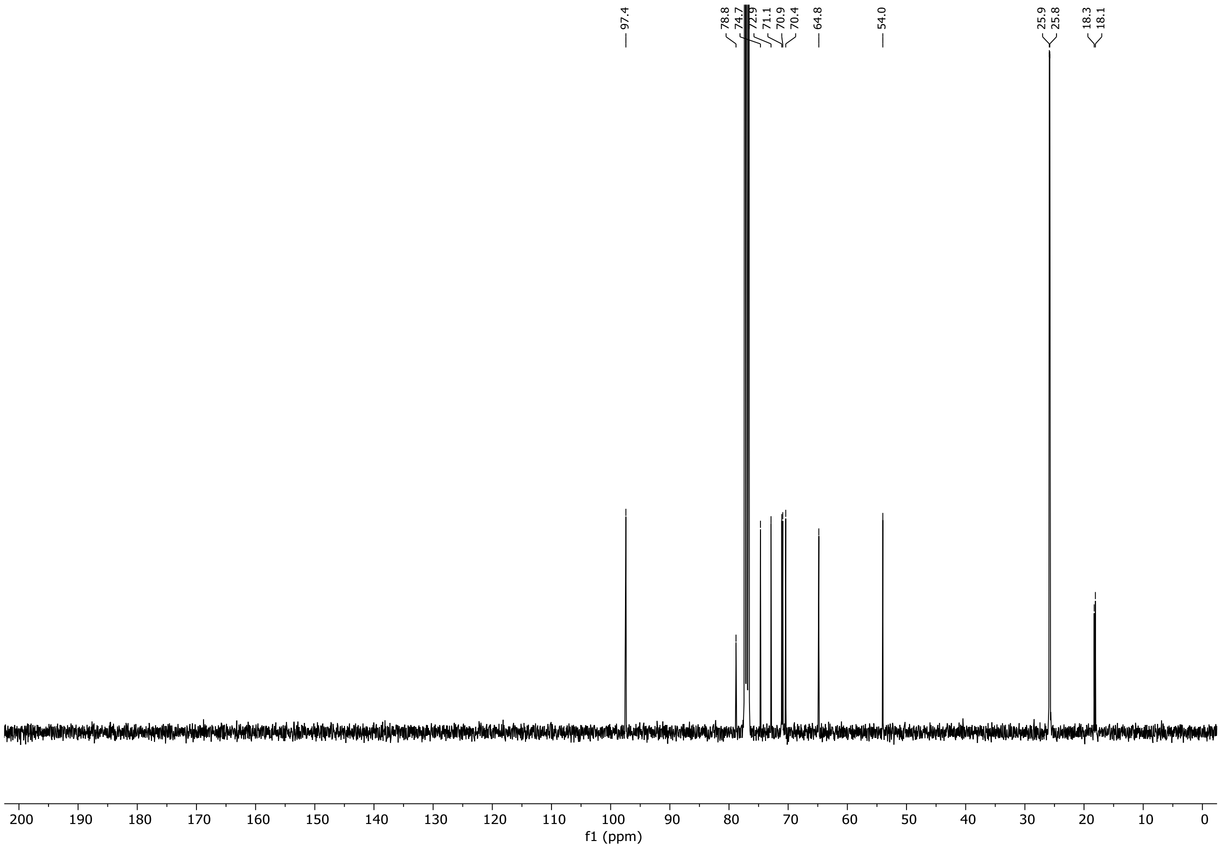

^{1}H-NMR, (400 MHz, $CDCl_3$): (**I3**)

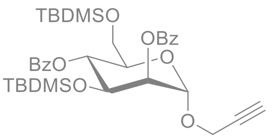

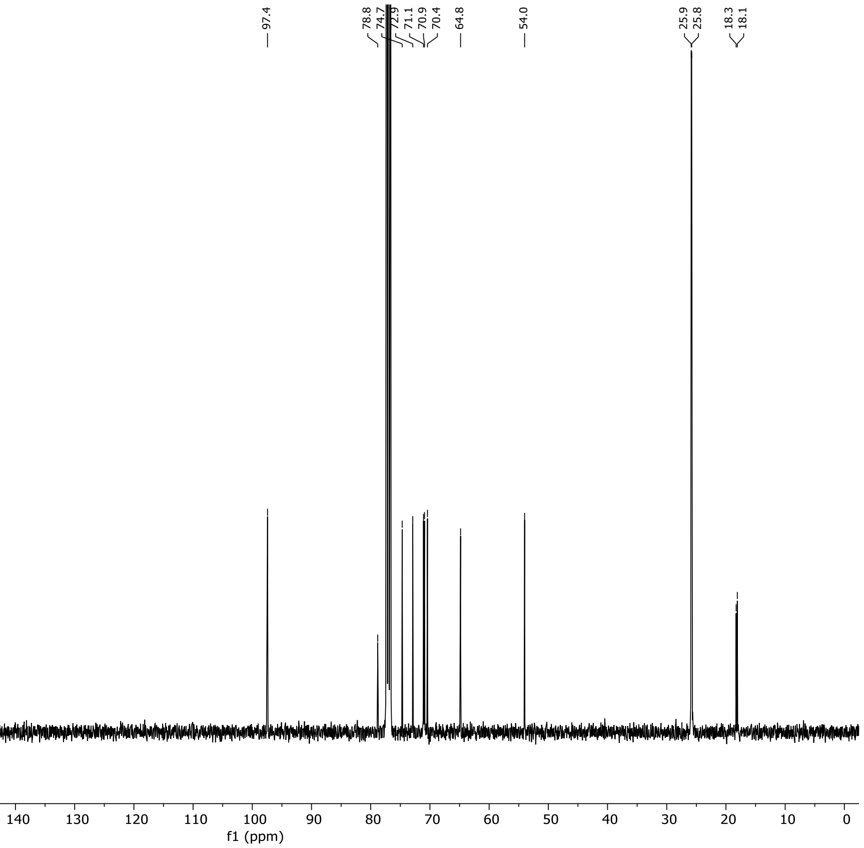

^{13}C-NMR, (101 MHz, $CDCl_3$): (**I3**)

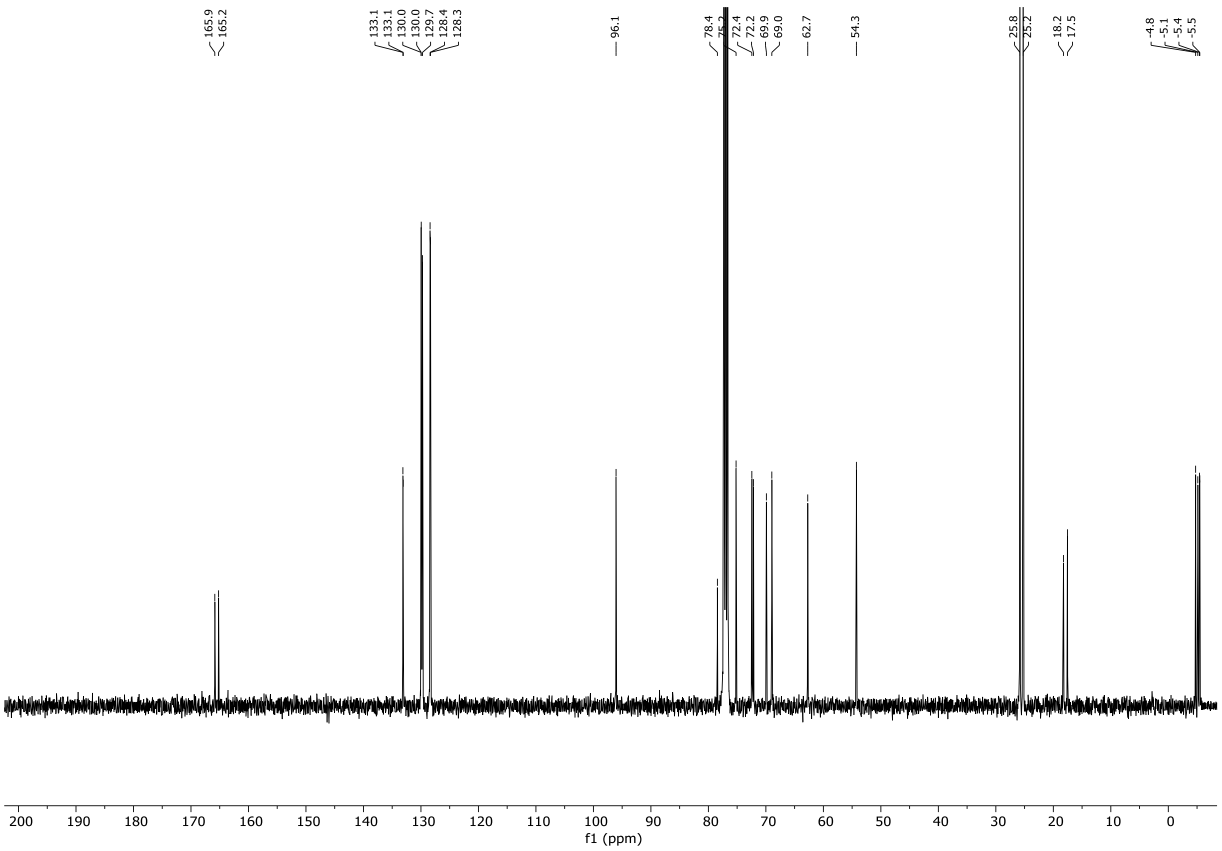

¹H-NMR, (400 MHz, $CDCl_3$): (**S2**)

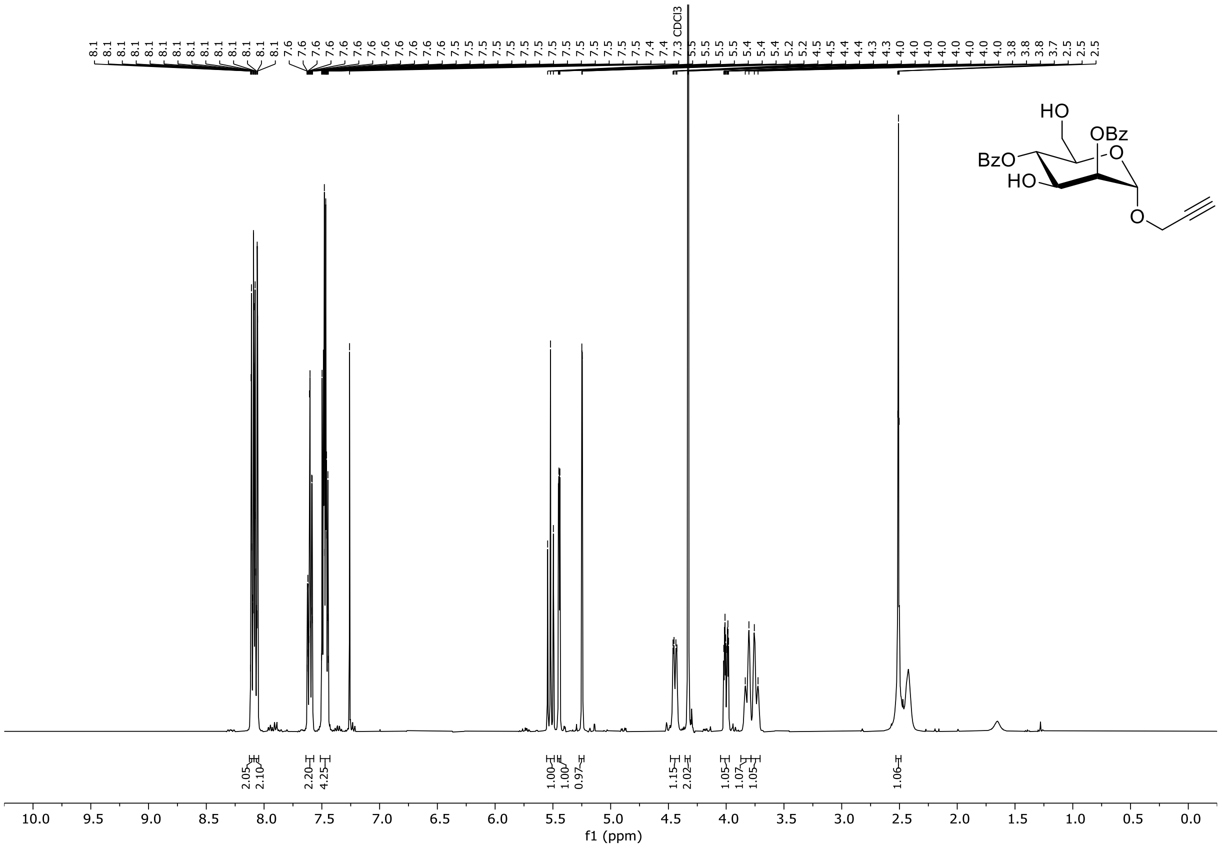

¹³C-NMR, (101 MHz, $CDCl_3$): (**S2**)

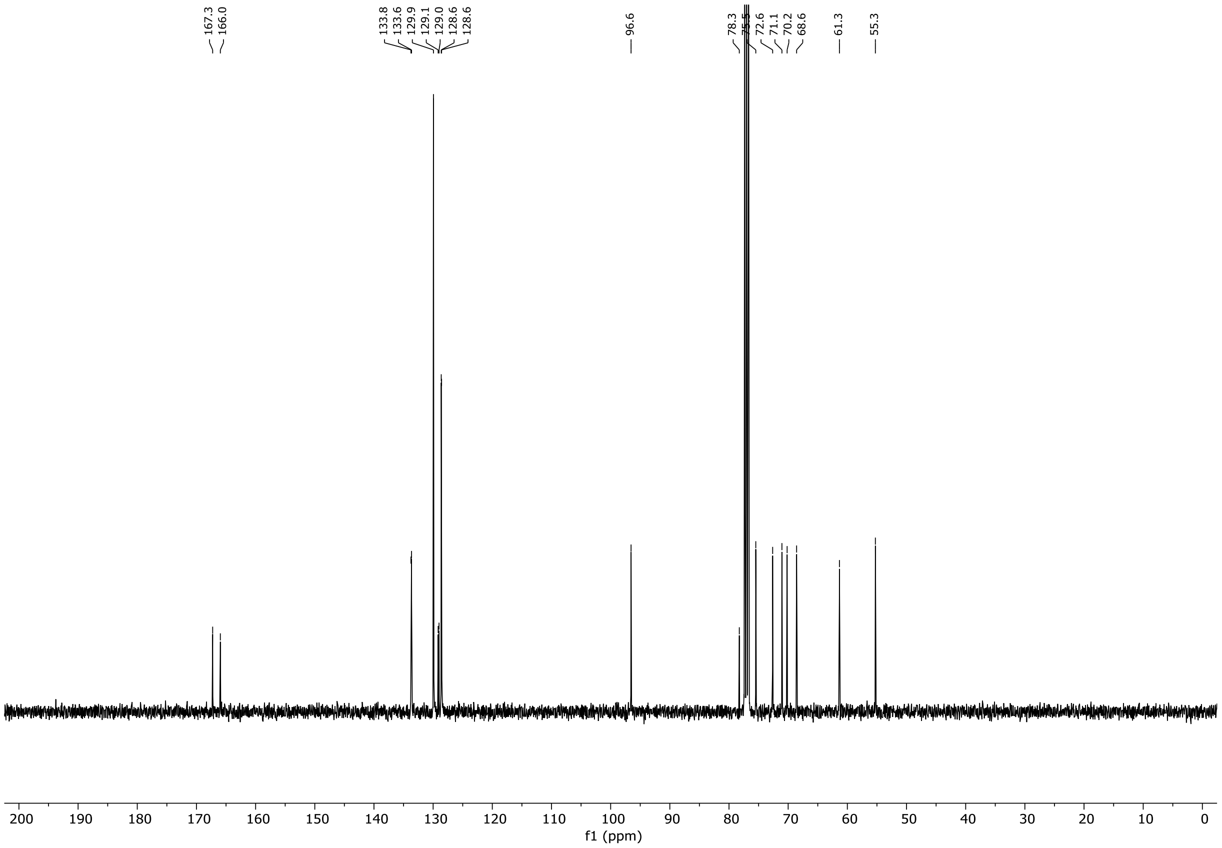

^{1}H-NMR, (600 MHz, $CDCl_3$): (**1**)

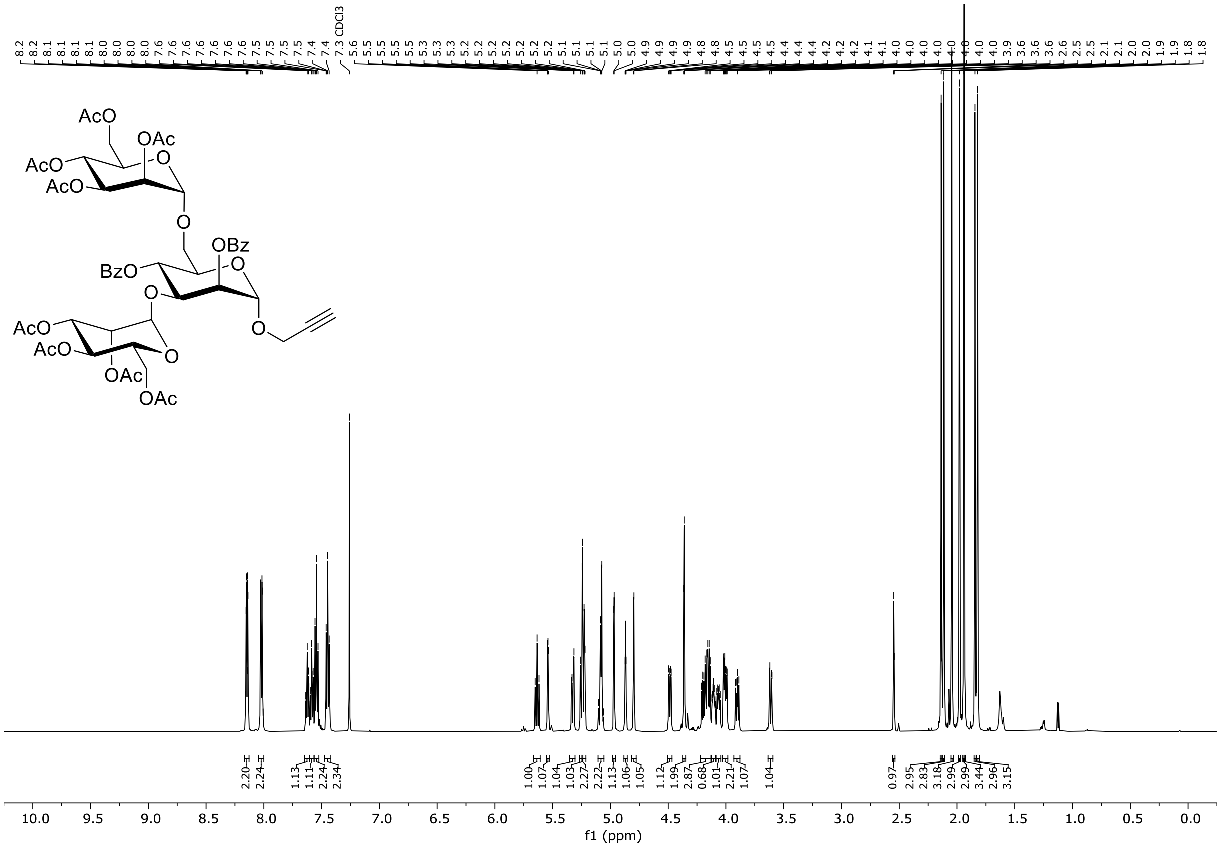

^{13}C-NMR, (151 MHz, $CDCl_3$): (**1**)

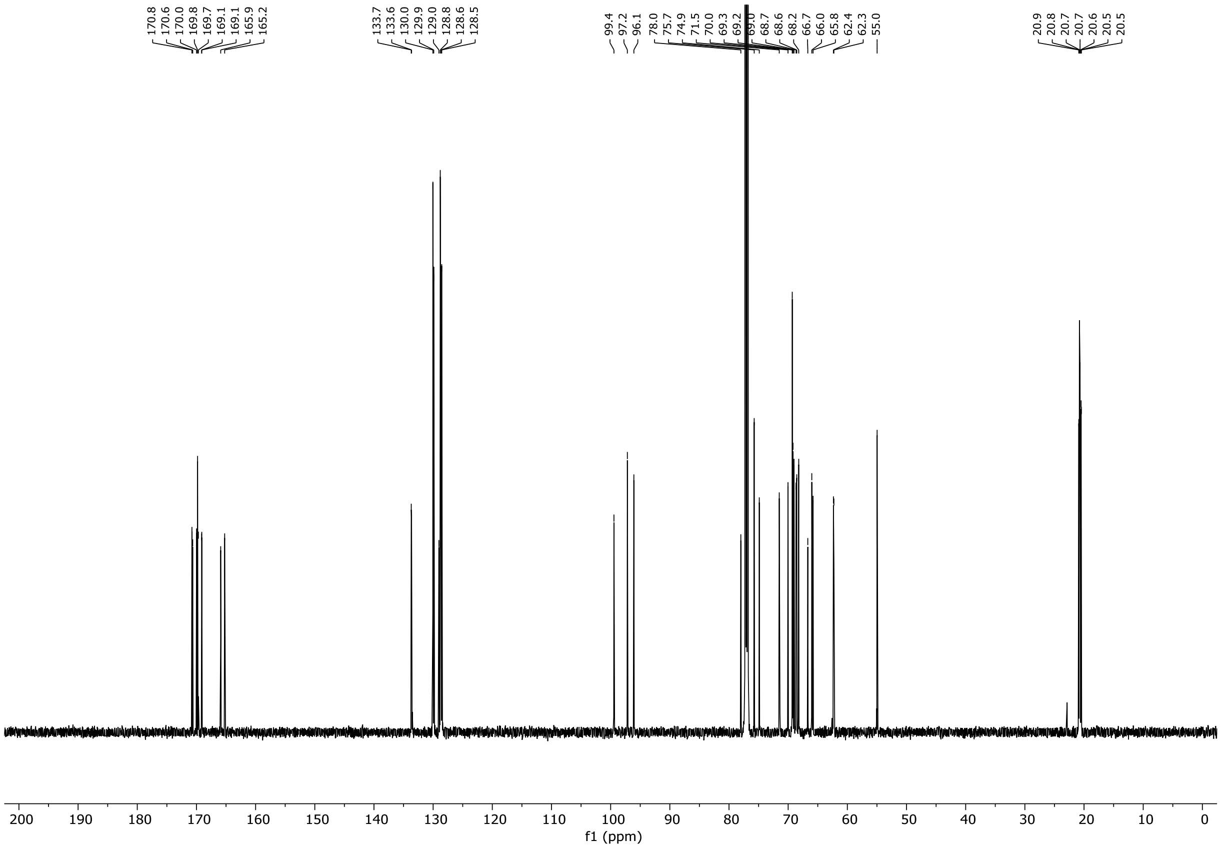

^{1}H-NMR, (400 MHz, $CDCl_3$): (**S14**)

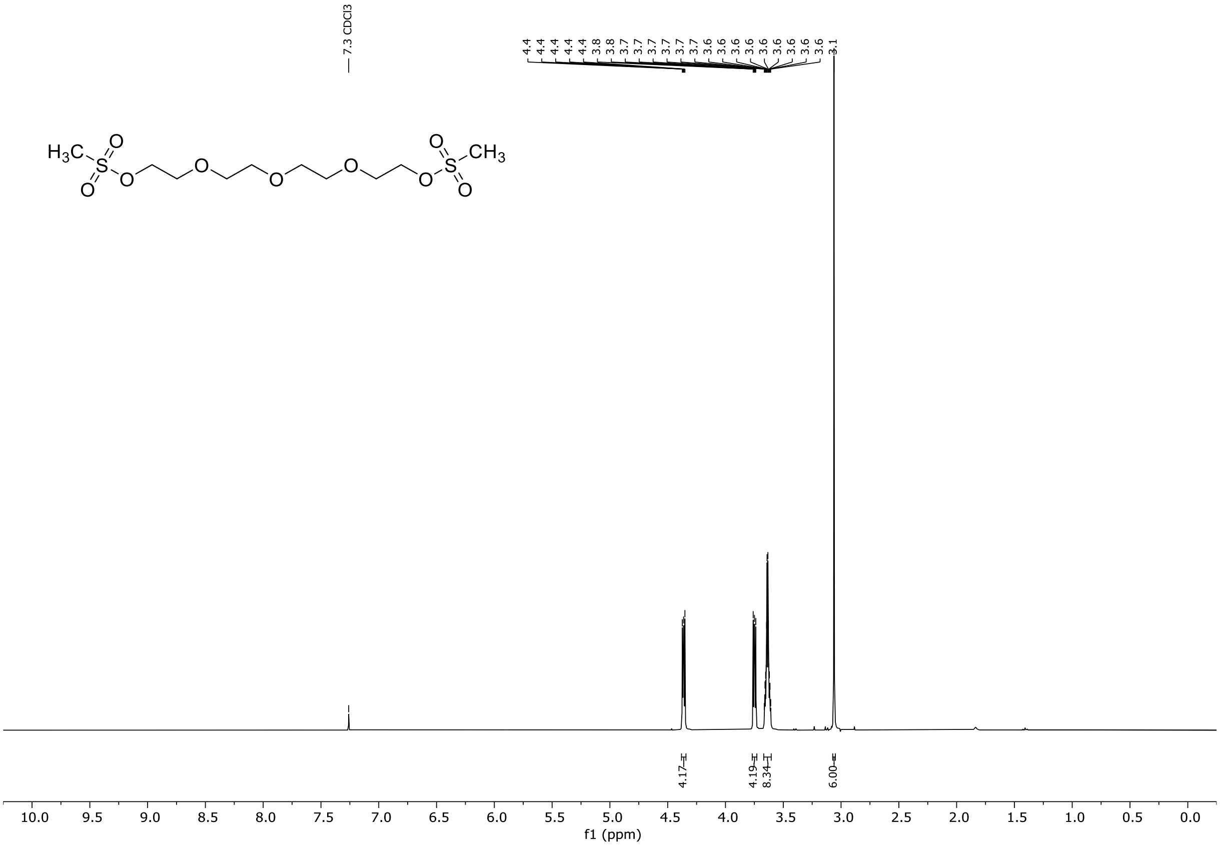

^{13}C-NMR, (101 MHz, $CDCl_3$): (**S14**)

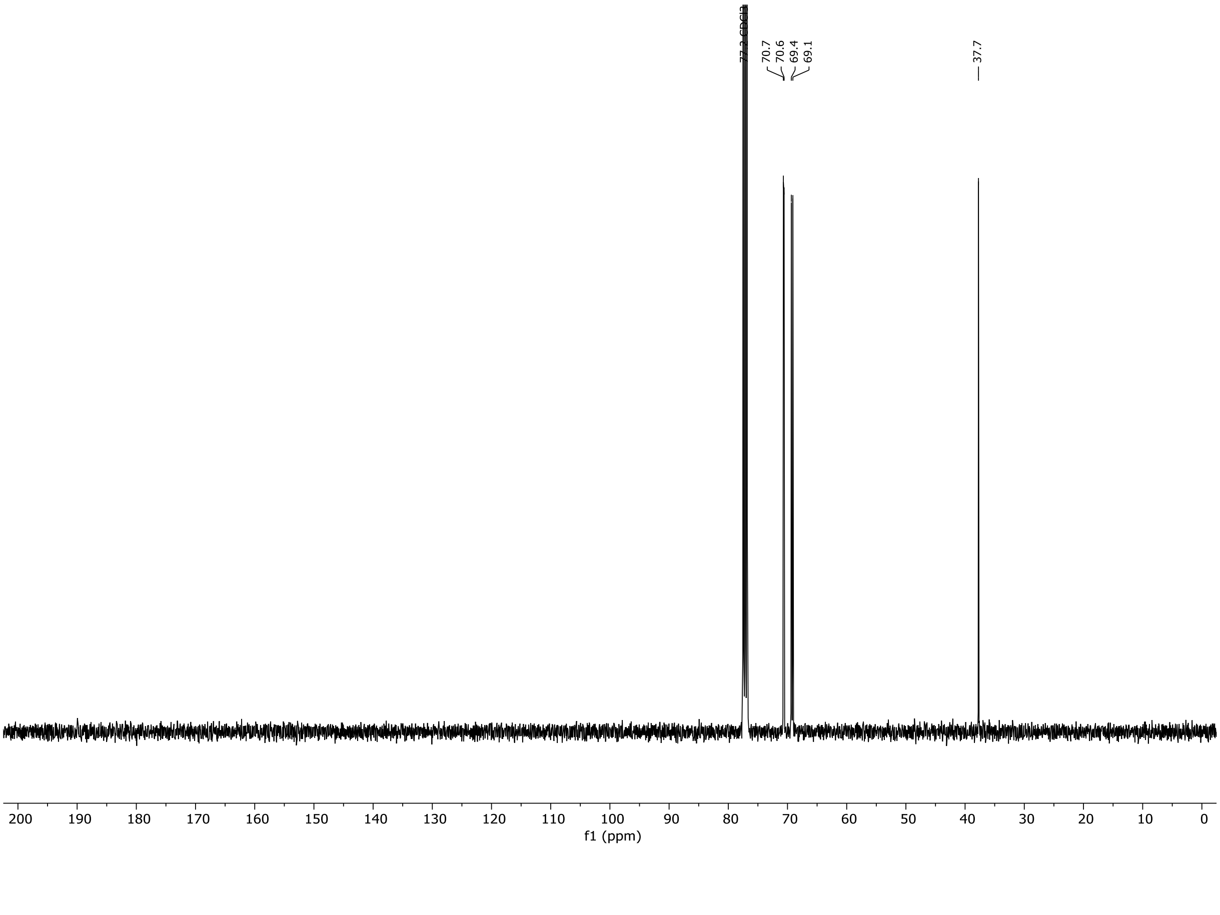

^{1}H-NMR, (400 MHz, $CDCl_3$): (**5**)

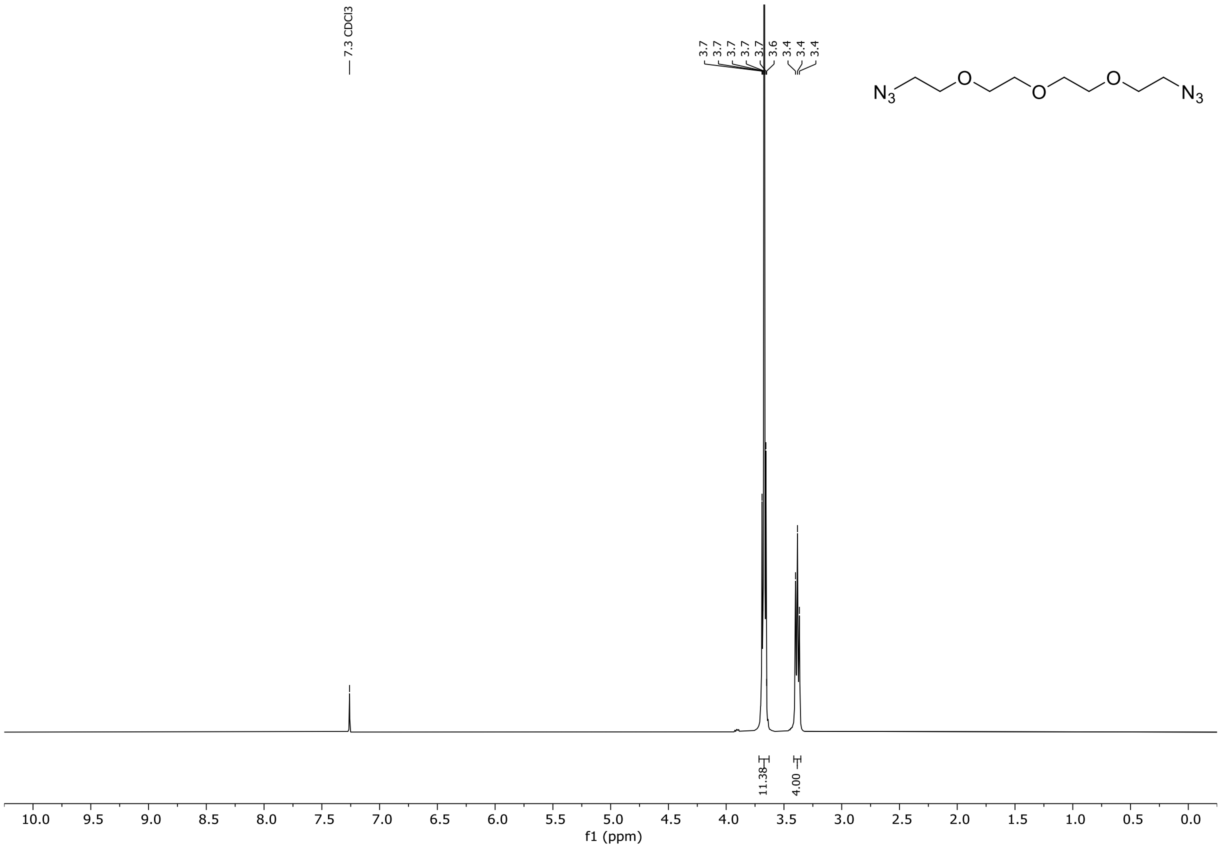

^{13}C-NMR, (101 MHz, $CDCl_3$): (**5**)

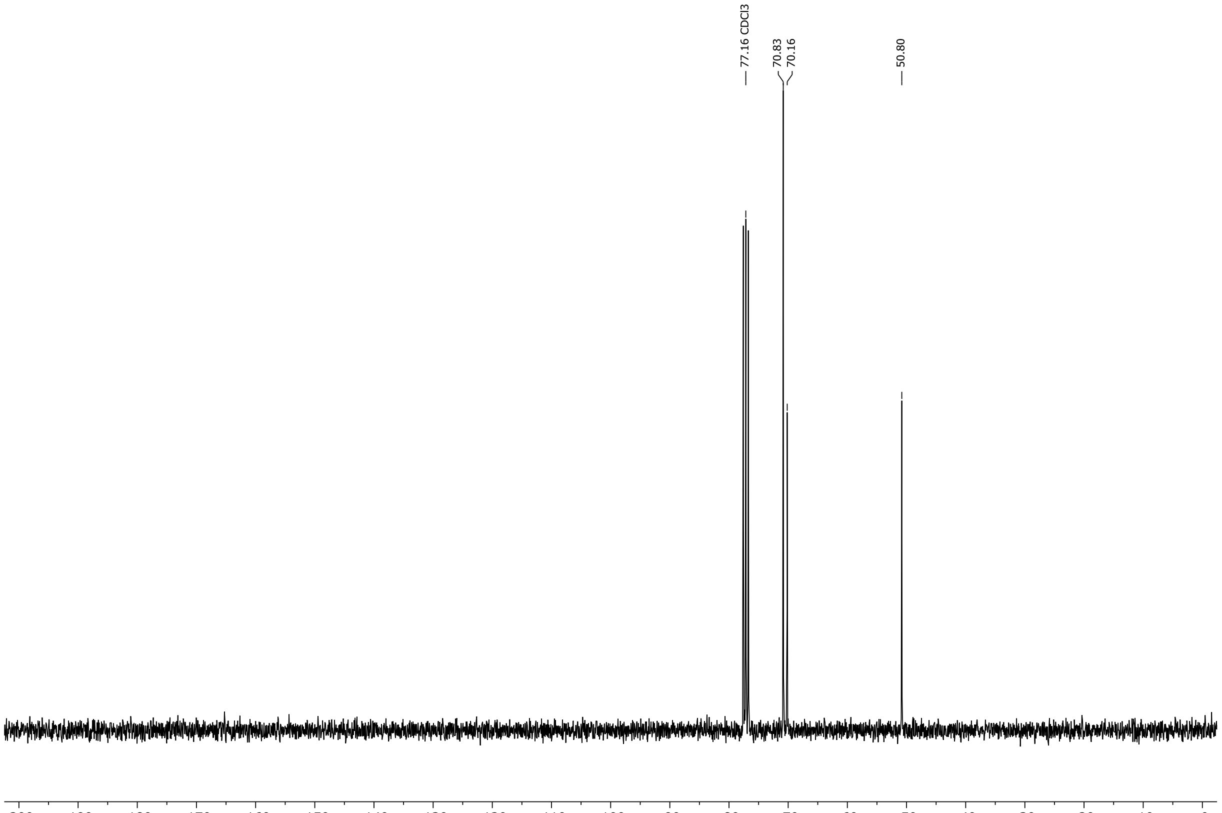

^{1}H-NMR, (300 MHz, $CDCl_3$): (**4**)

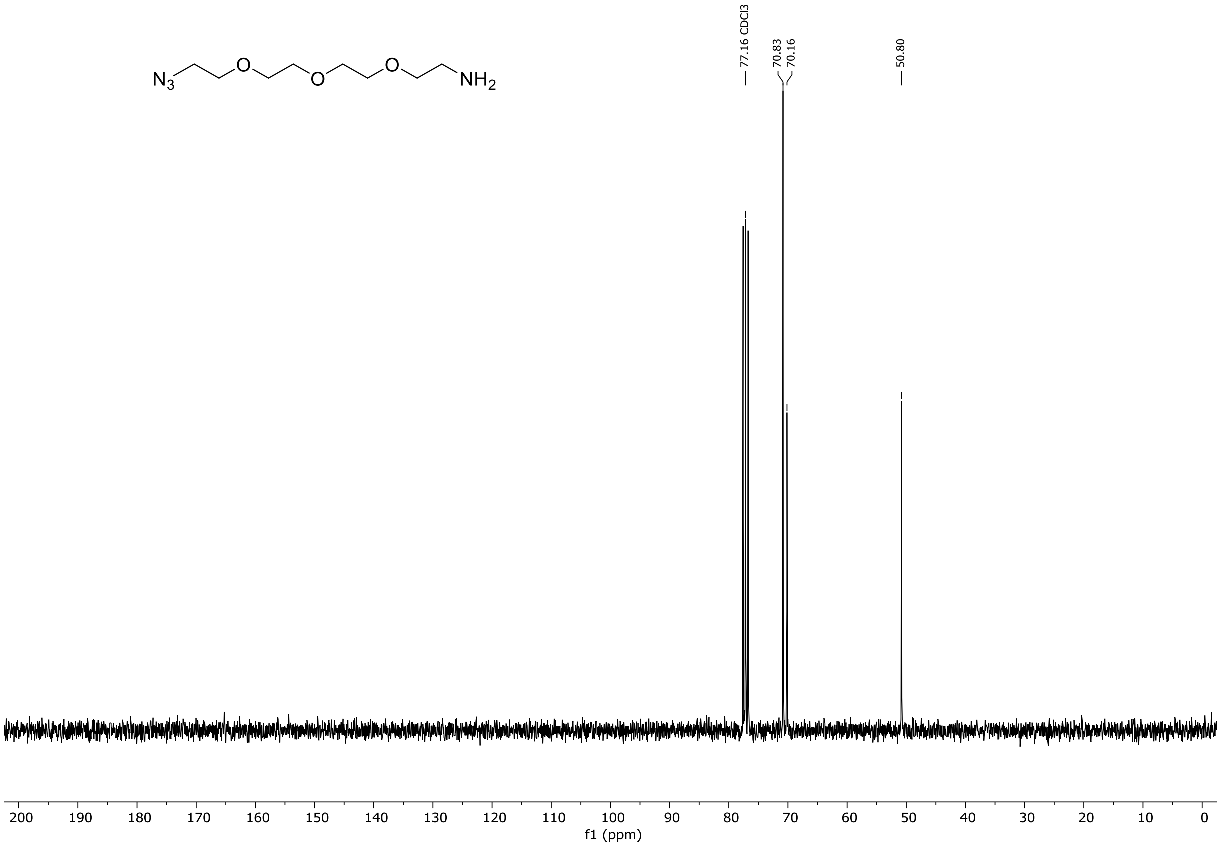

^{13}C-NMR, (75.5 MHz, $CDCl_3$): (**4**)

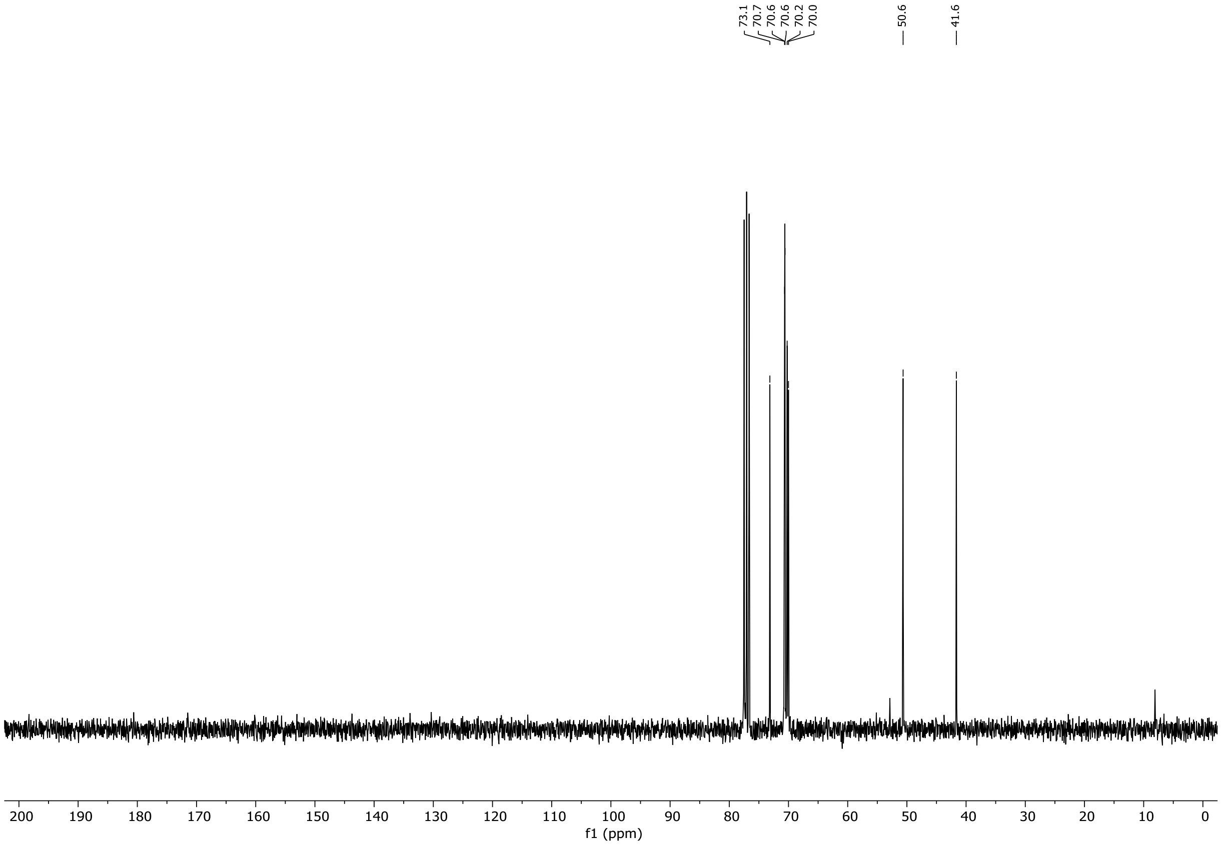

^{1}H-NMR, (400 MHz, $CDCl_3$): (**S15**)

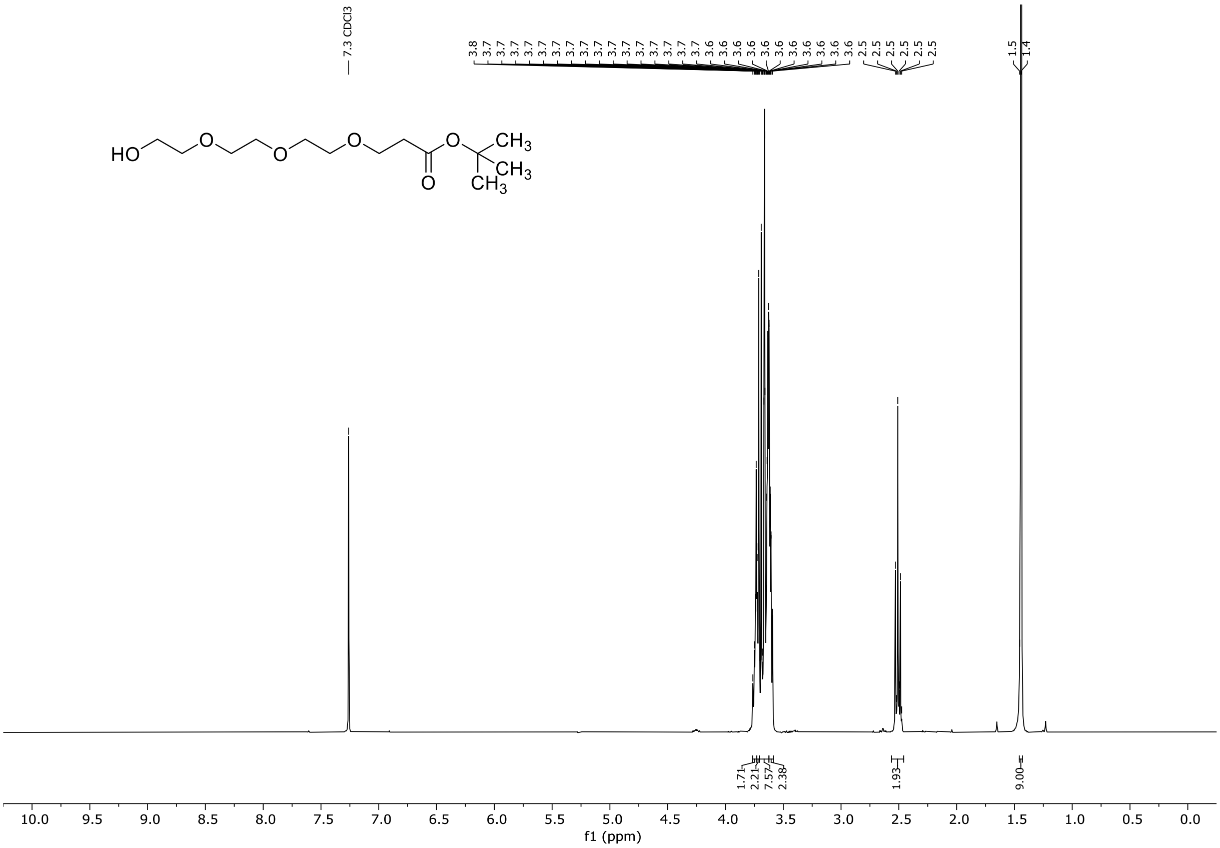

^{13}C-NMR, (101 MHz, $CDCl_3$): (**S15**)

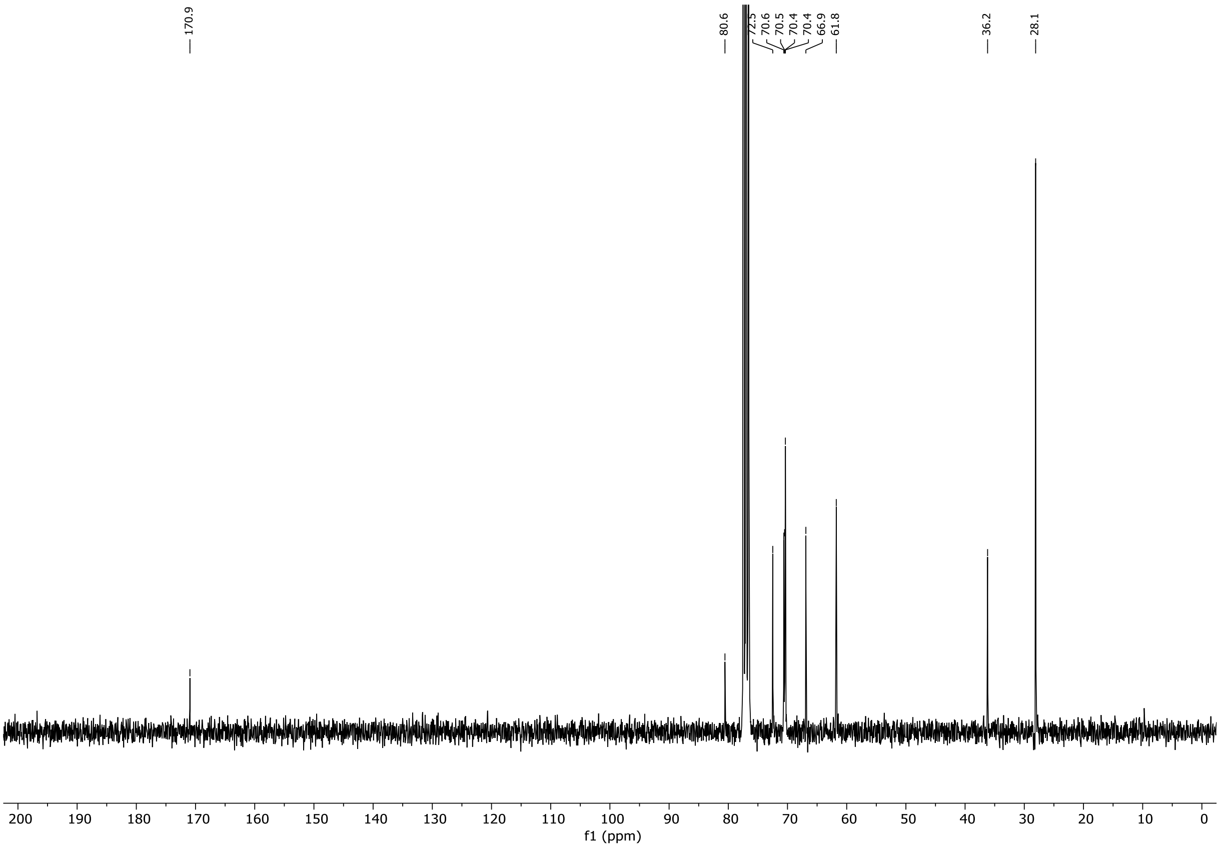

^{1}H-NMR, (400 MHz, $CDCl_3$): (**S16**)

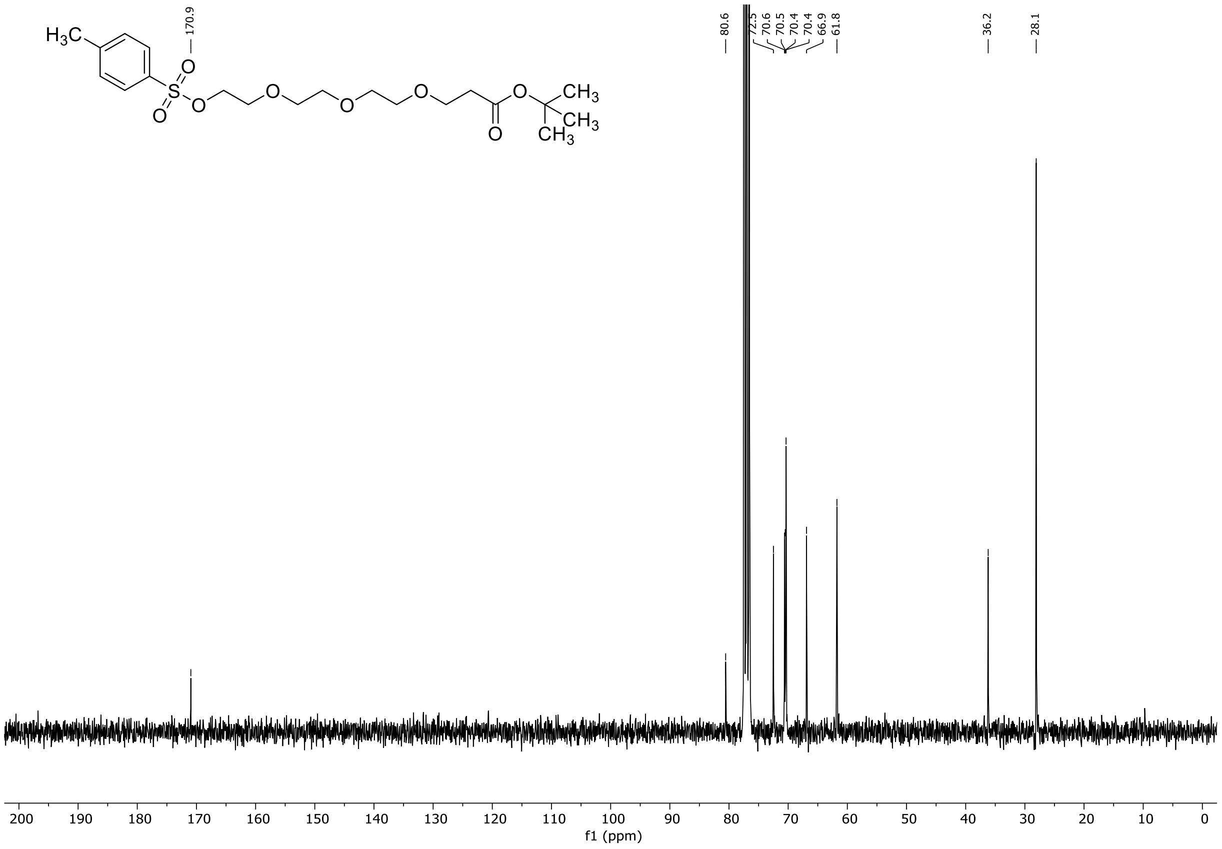

^{13}C-NMR, (101 MHz, $CDCl_3$): (**S16**)

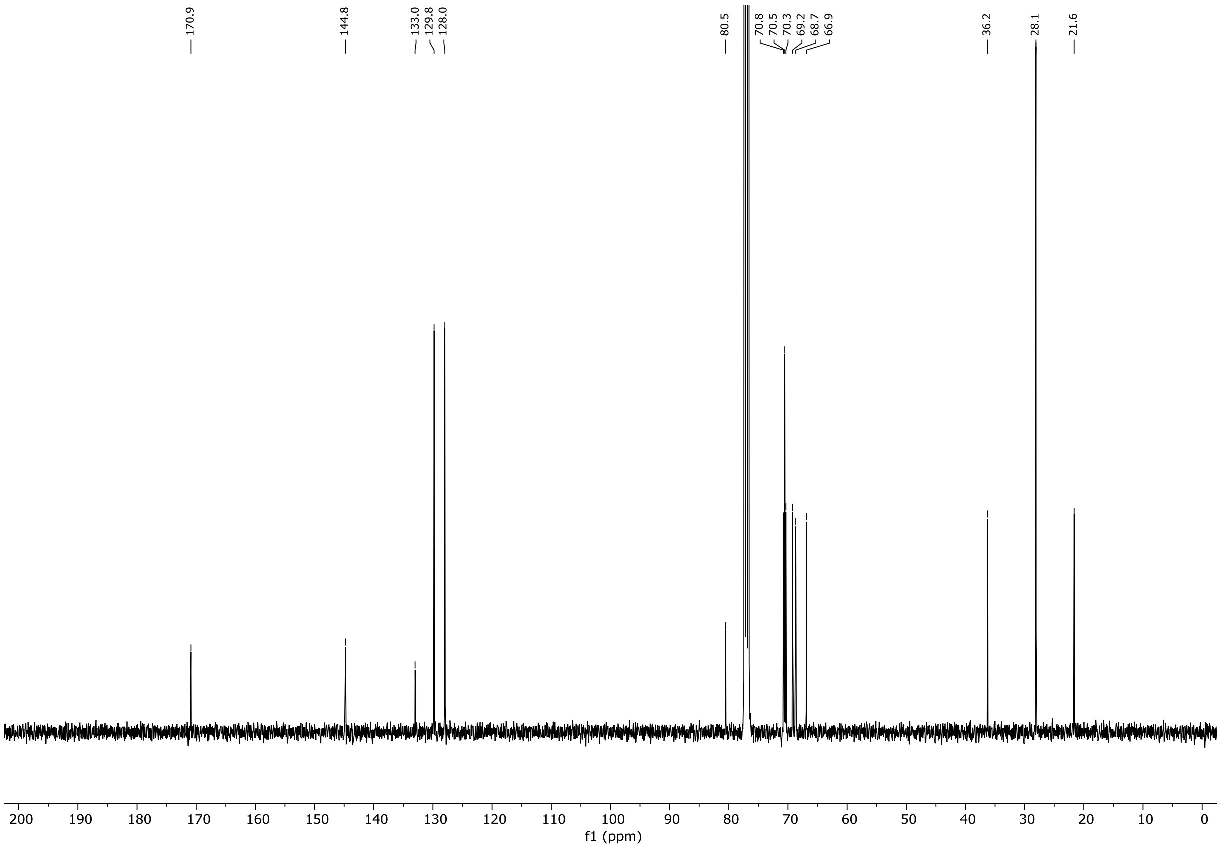

^{1}H-NMR, (400 MHz, $CDCl_3$): (**S3**)

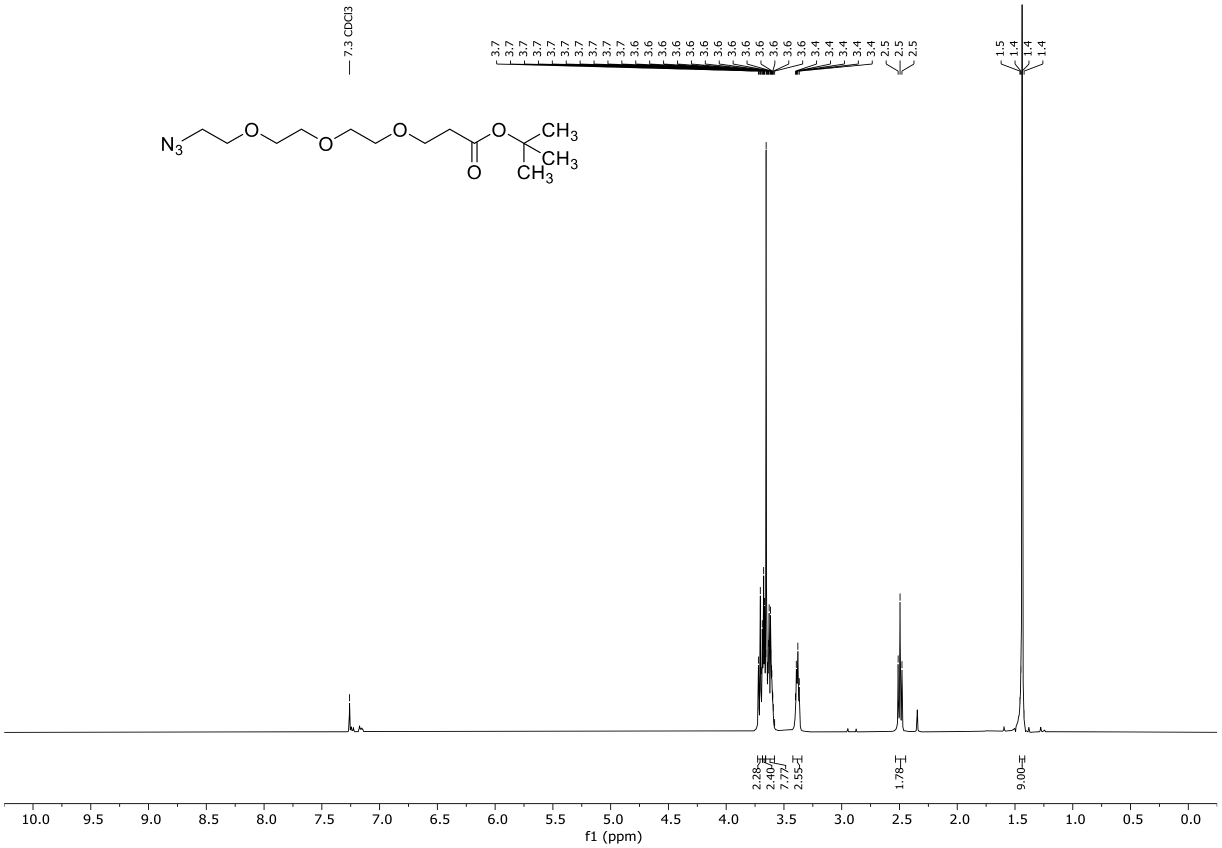

^{13}C-NMR, (101 MHz, $CDCl_3$): (**S3**)

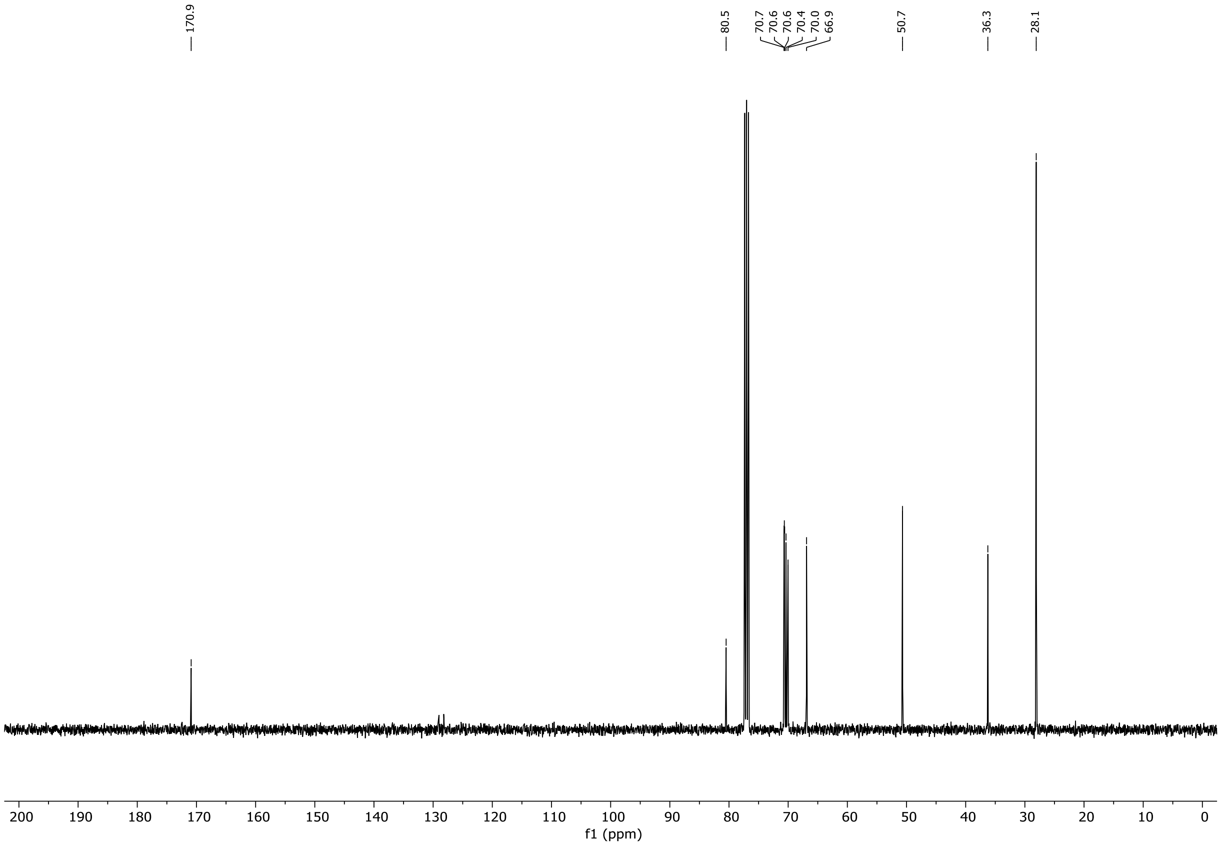

¹H-NMR, (300 MHz, $CDCl_3$): (**3**)

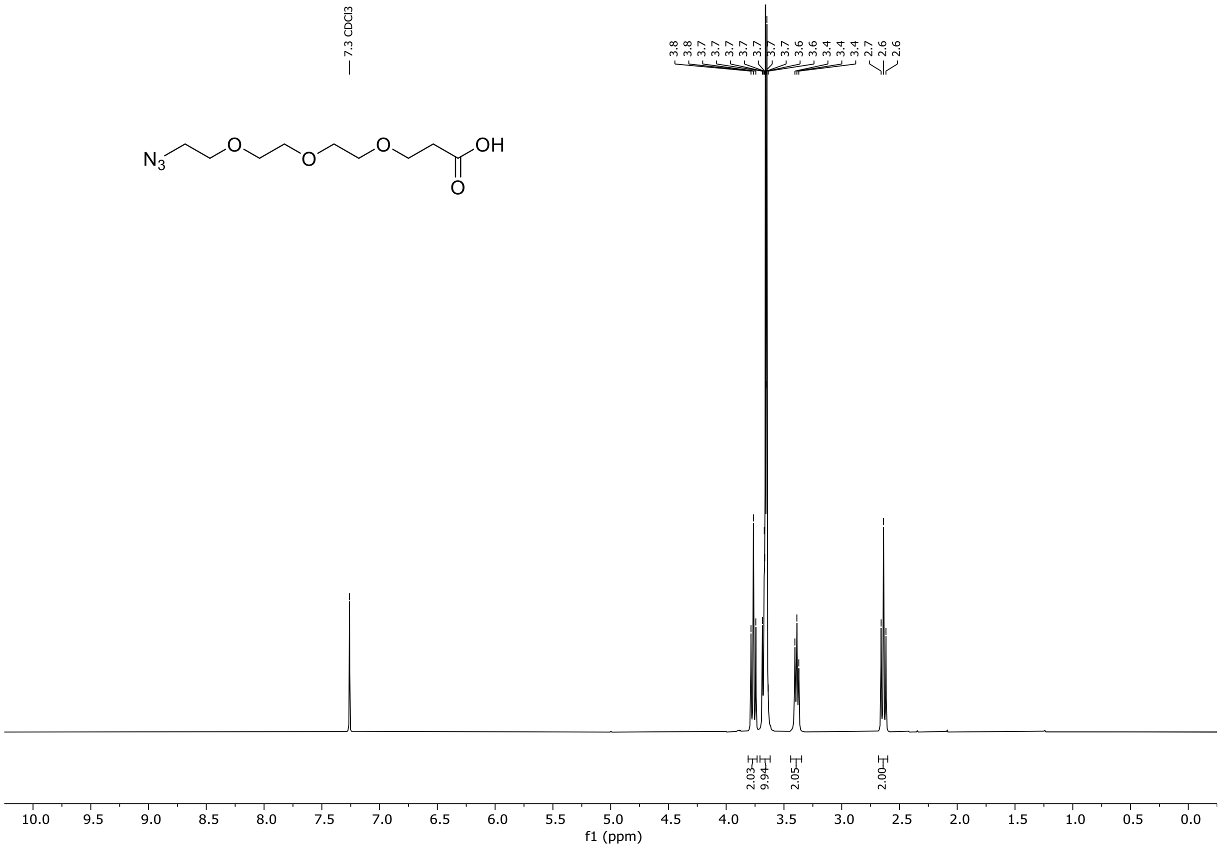

¹³C-NMR, (75.5 MHz, $CDCl_3$): (**3**)

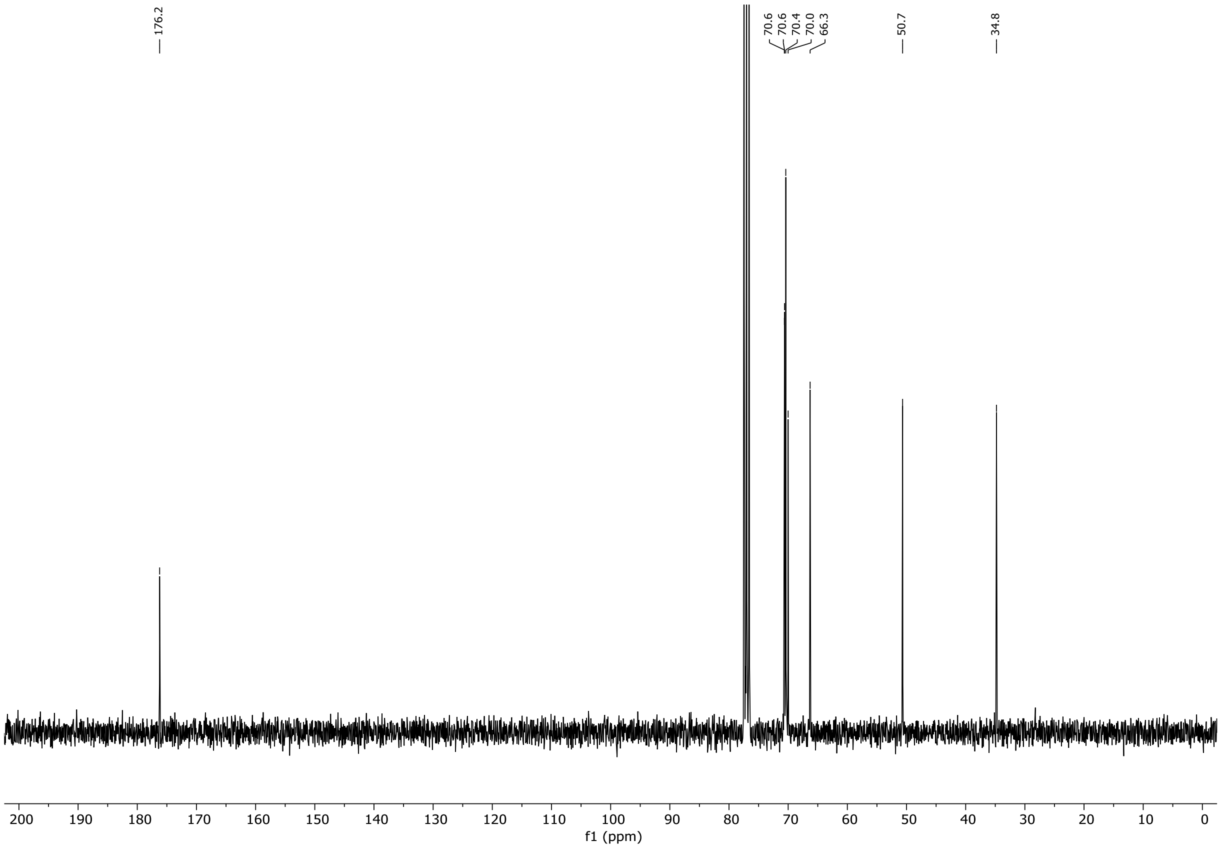

¹H-NMR, (400 MHz, $CDCl_3$): (**S4**)

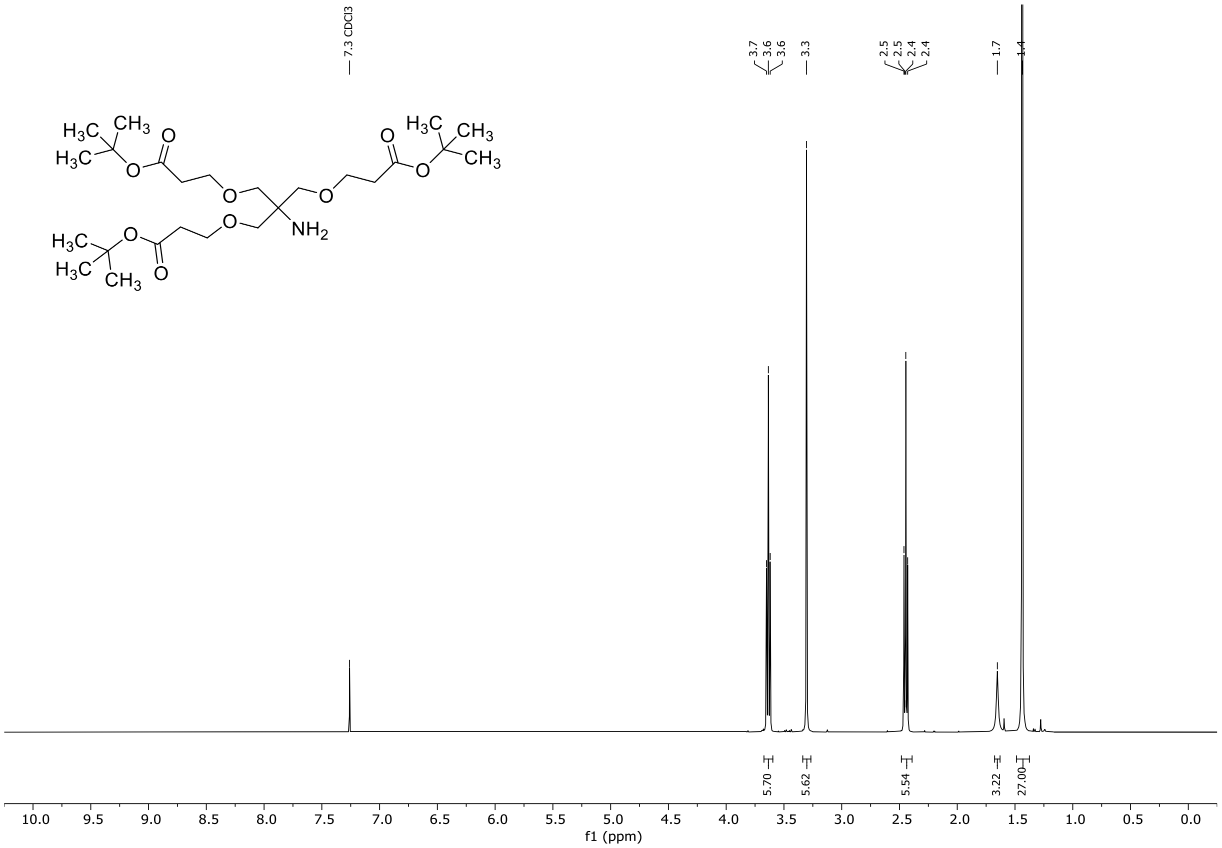

¹³C-NMR, (101 MHz, $CDCl_3$): (**S4**)

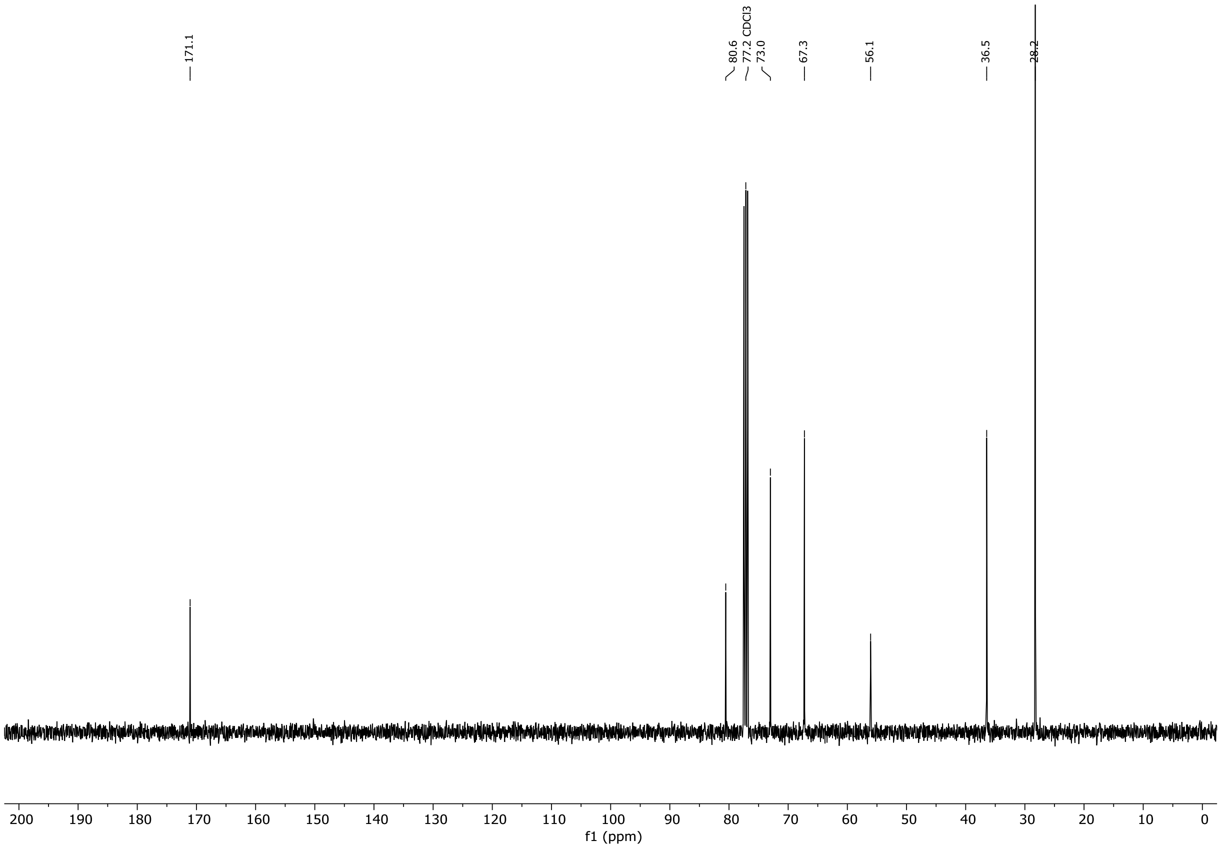

[1]**H-NMR**, (400 MHz, $CDCl_3$): (**I4**)

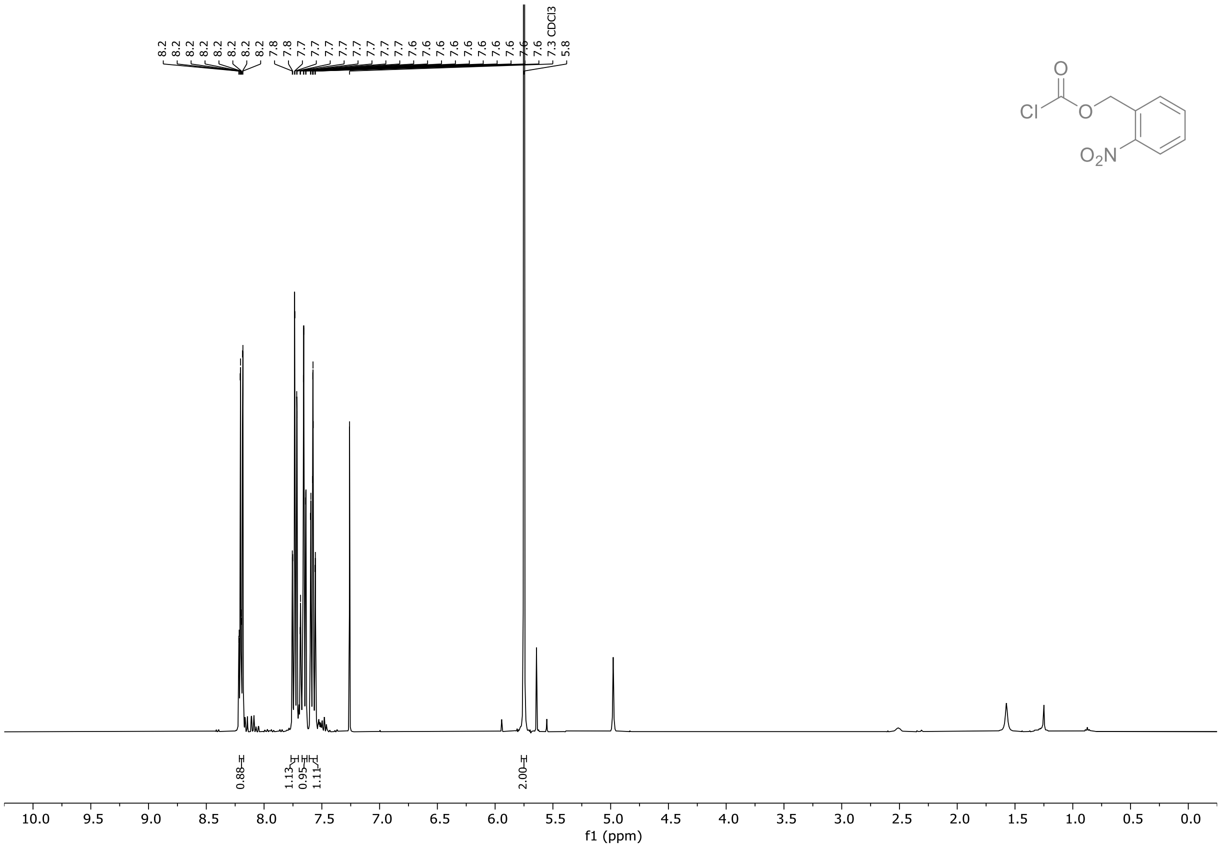

[13]**C-NMR**, (101 MHz, $CDCl_3$): (**I4**)

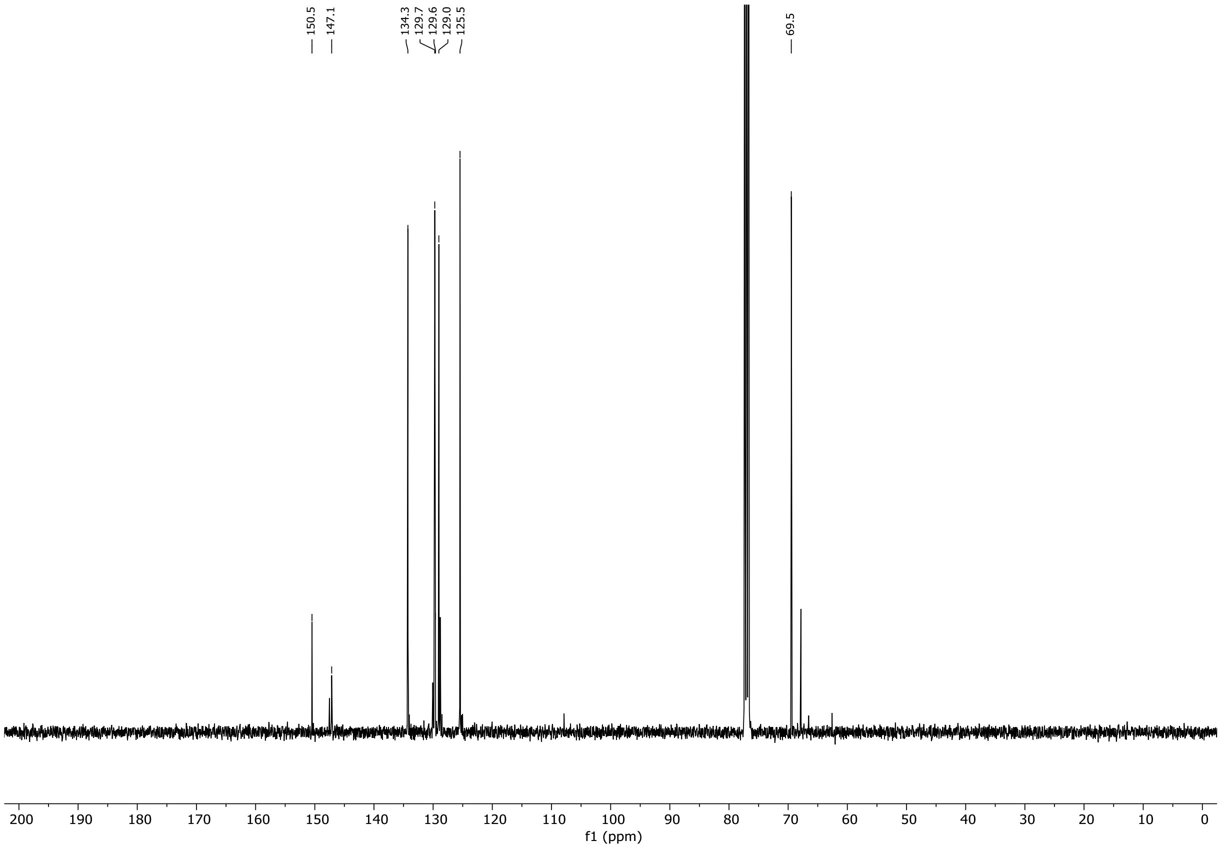

^{1}H-NMR, (400 MHz, $CDCl_3$): (**S5**)

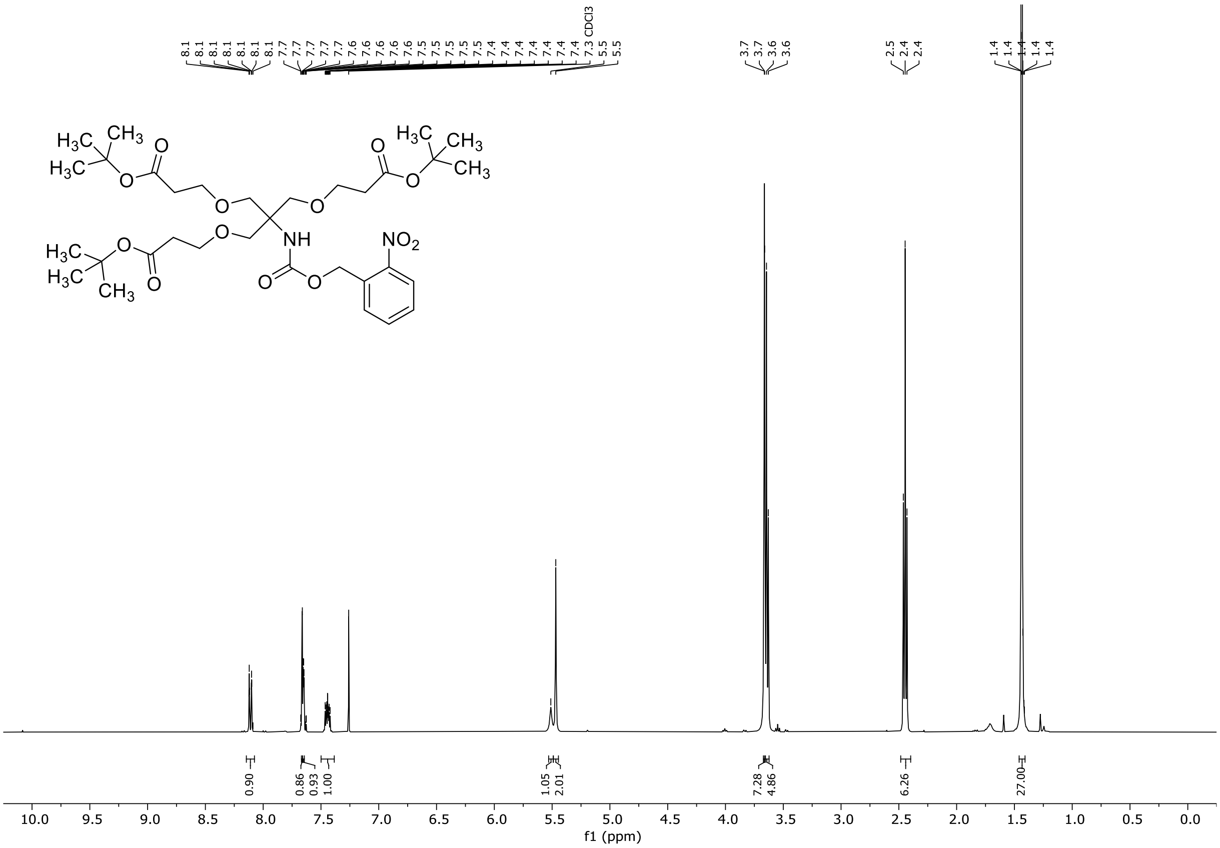

^{13}C-NMR, (101 MHz, $CDCl_3$): (**S5**)

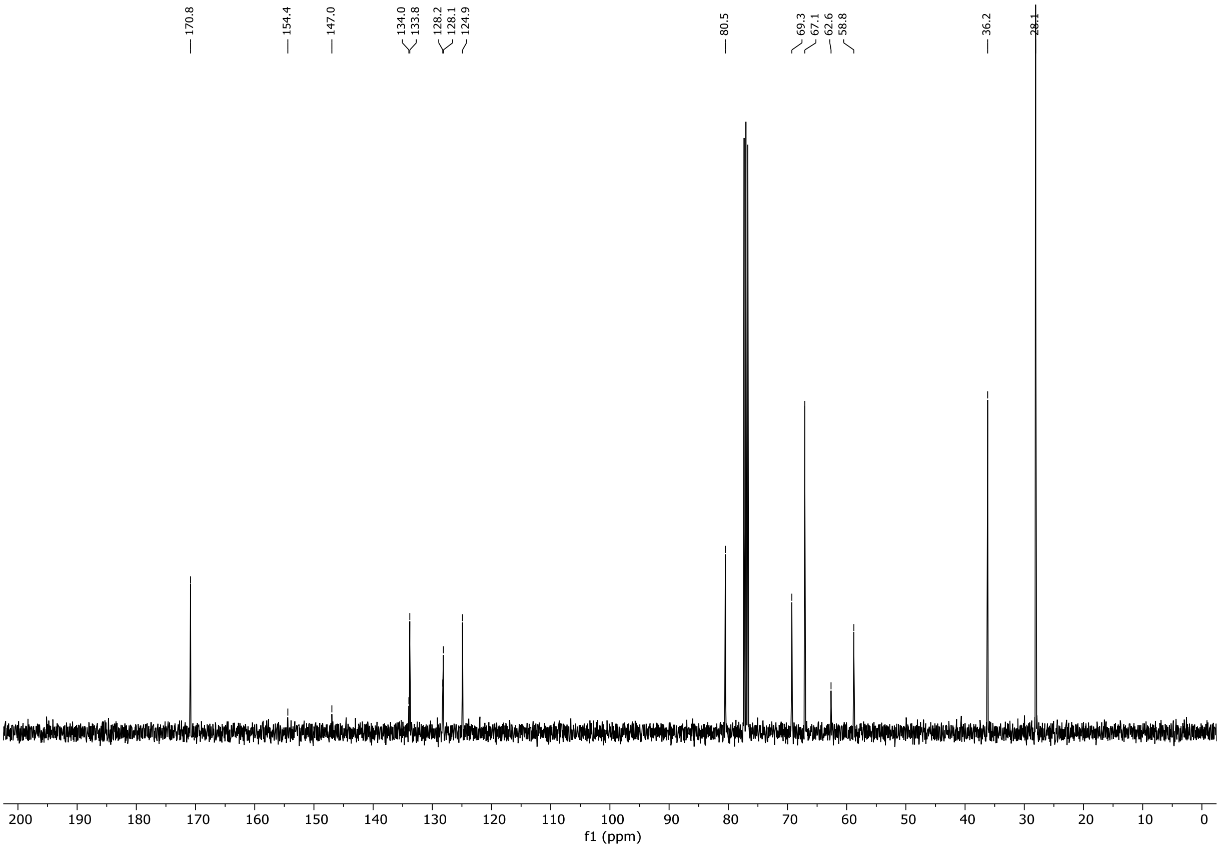

^{1}H-NMR, (400 MHz, $(CD_3)_2SO$): (**S6**)

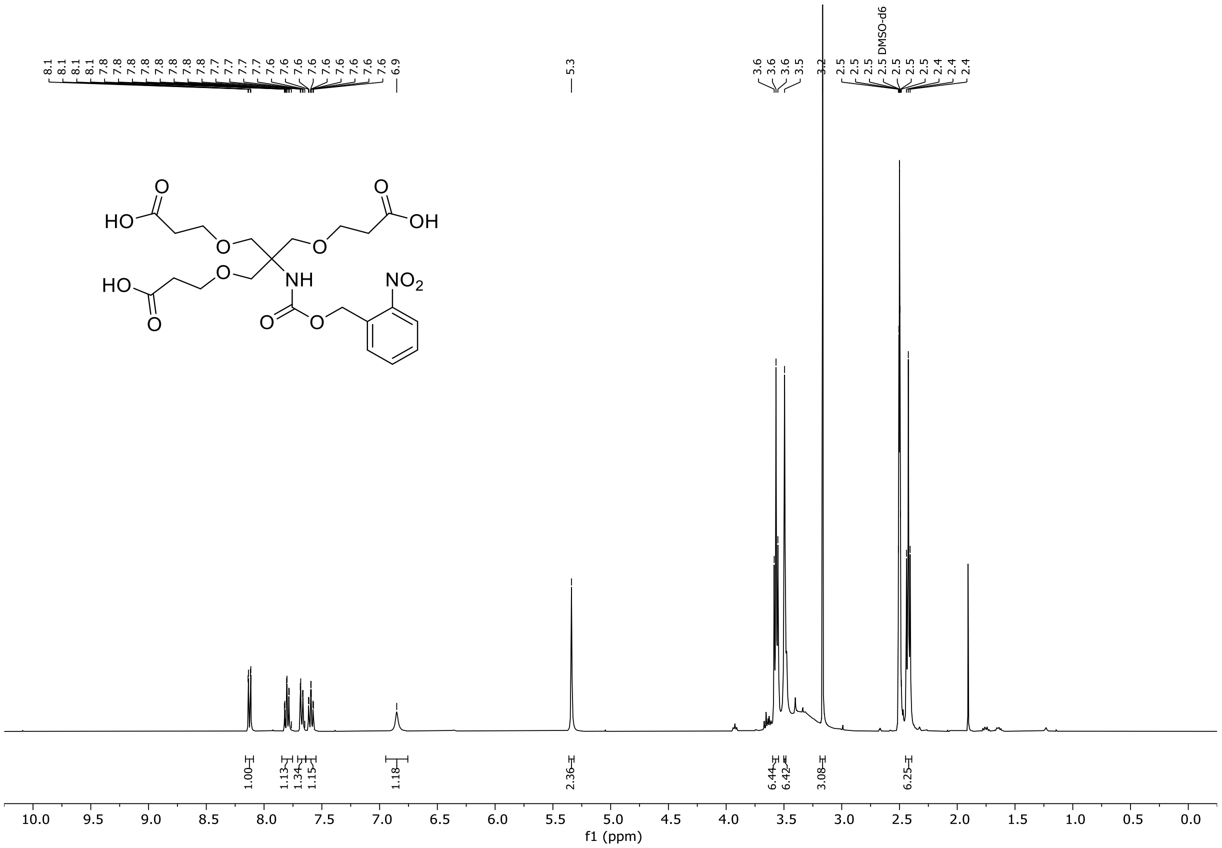

^{13}C-NMR, (101 MHz, $(CD_3)_2SO$): (**S6**)

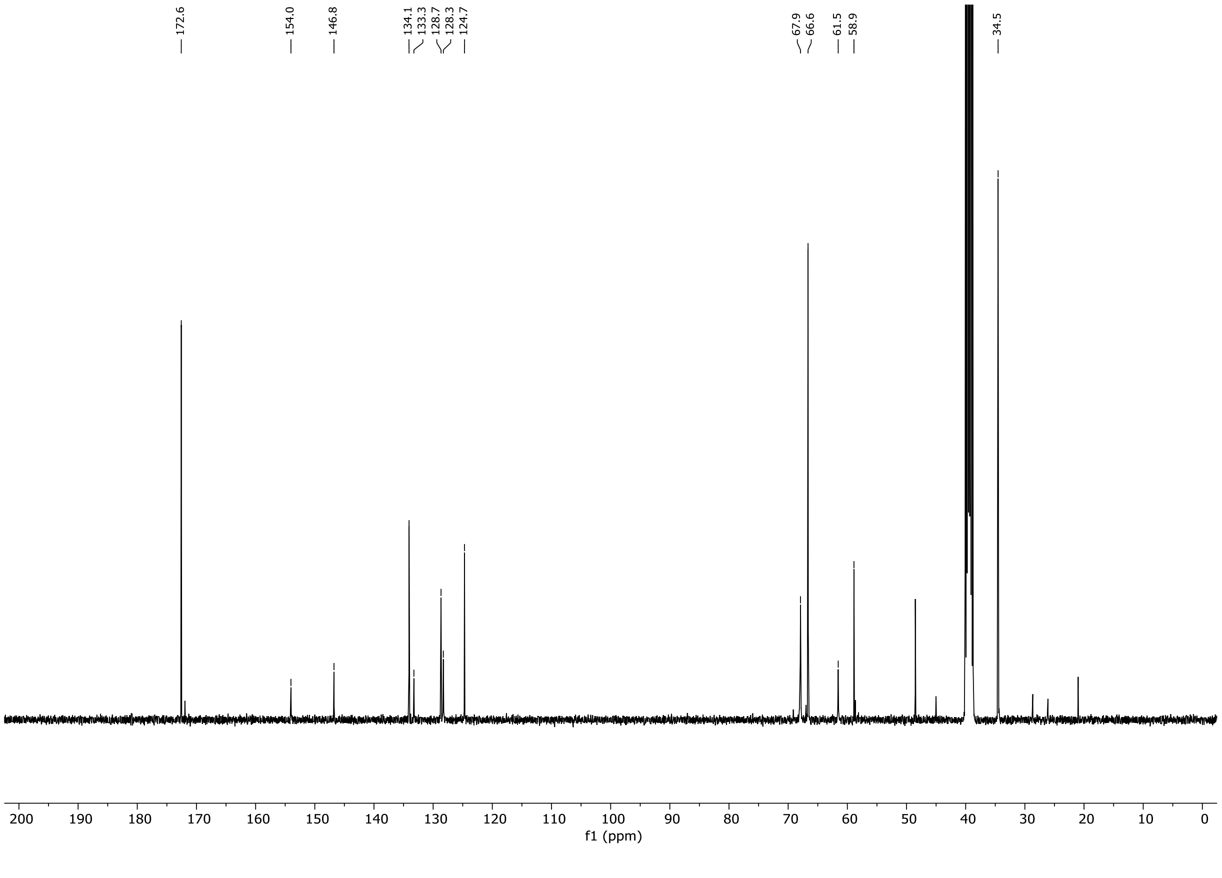

^{1}H-NMR, (600 MHz, $CDCl_3$): (**6**)

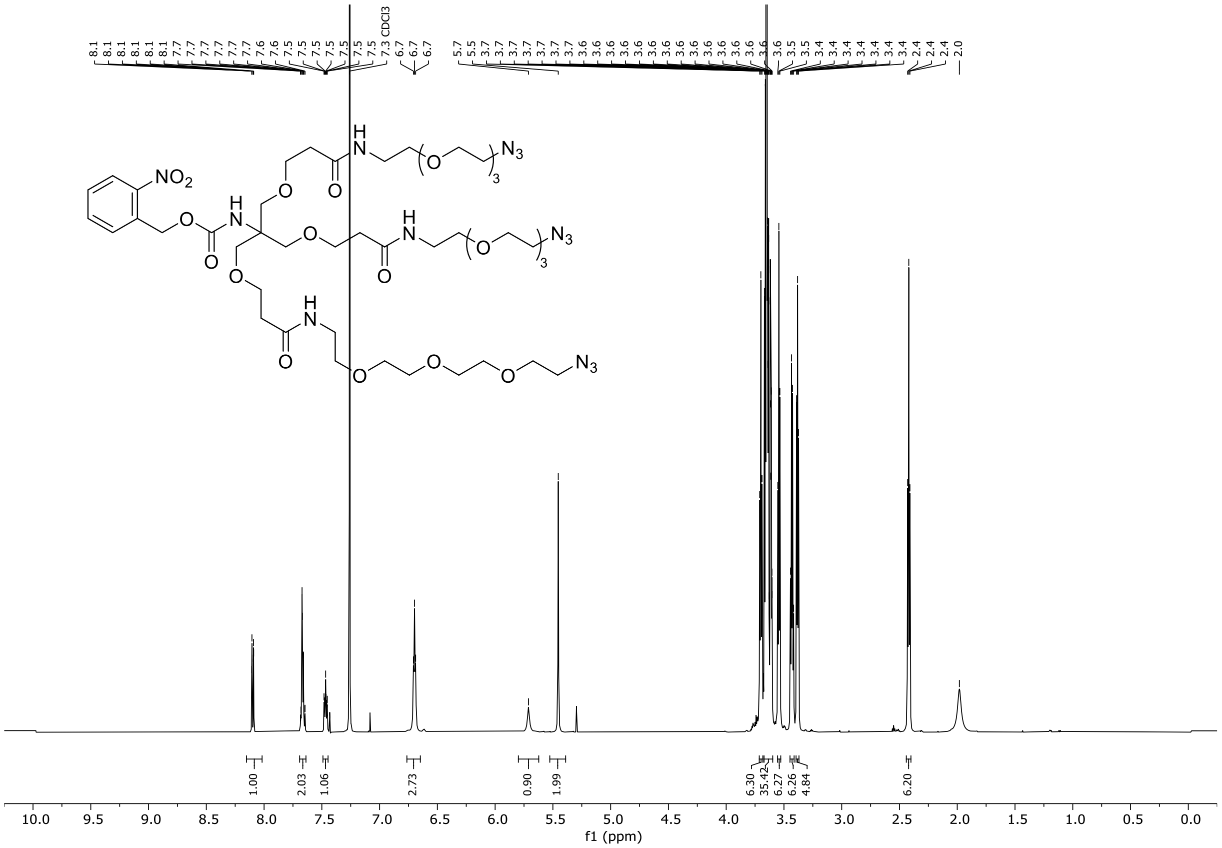

^{13}C-NMR, (151 MHz, $CDCl_3$): (**6**)

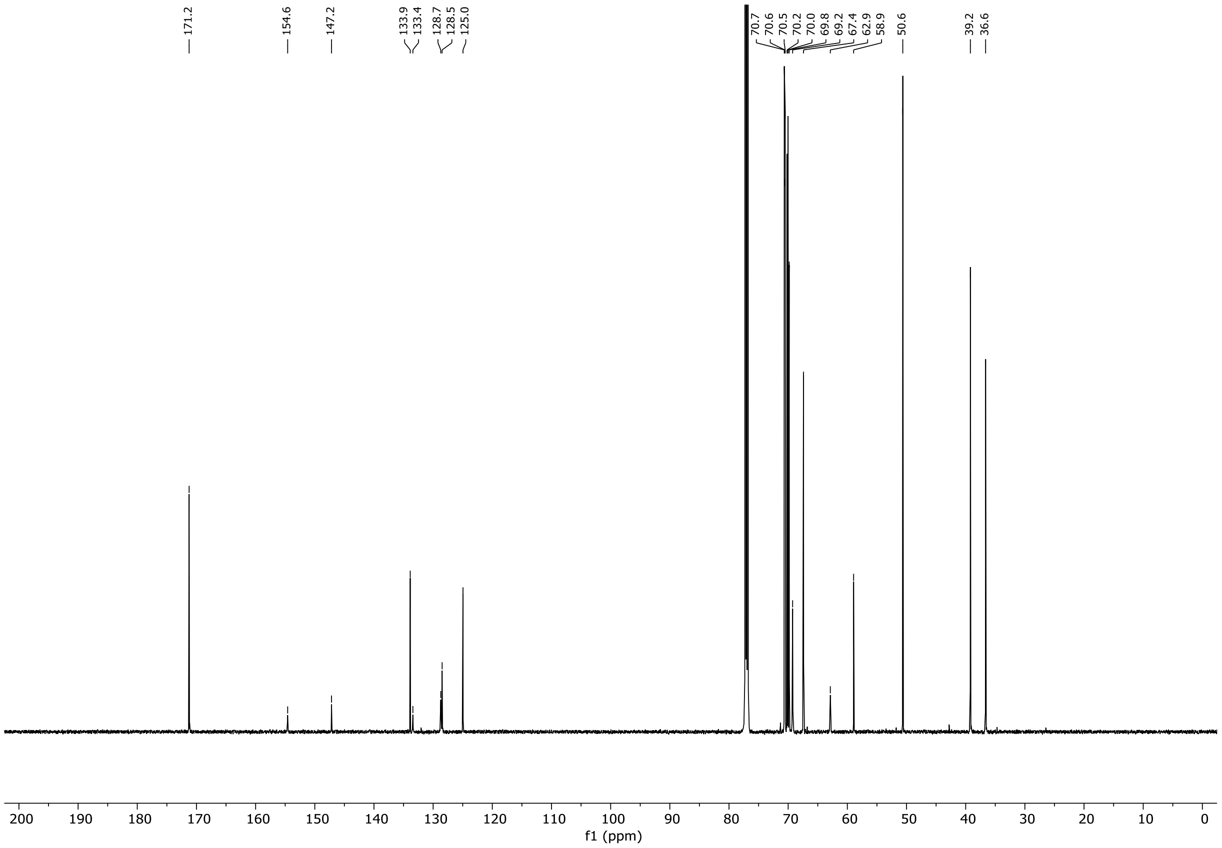

^{1}H-NMR, (600 MHz, CD_3OD): (**Man$_3$N$_3$**)

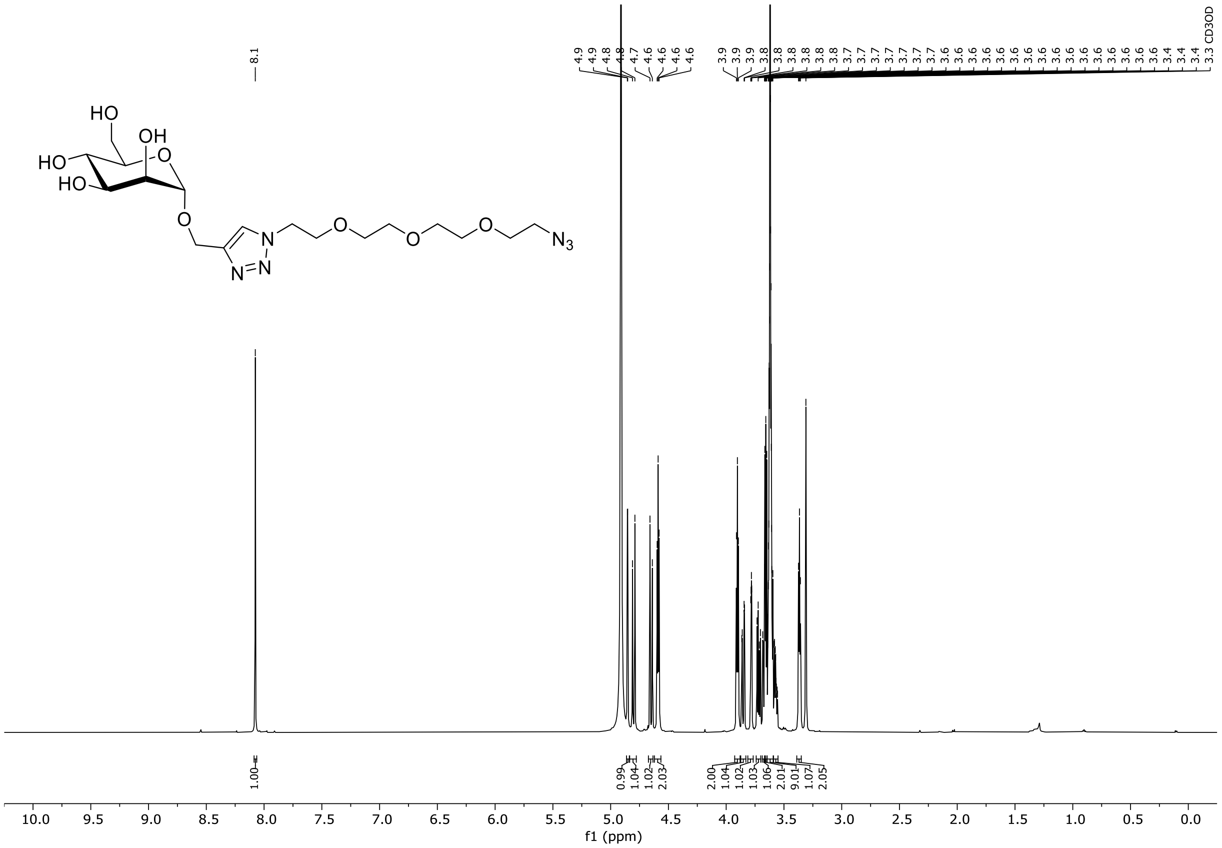

^{13}C-NMR, (151 MHz, CD_3OD): (**Man$_3$N$_3$**)

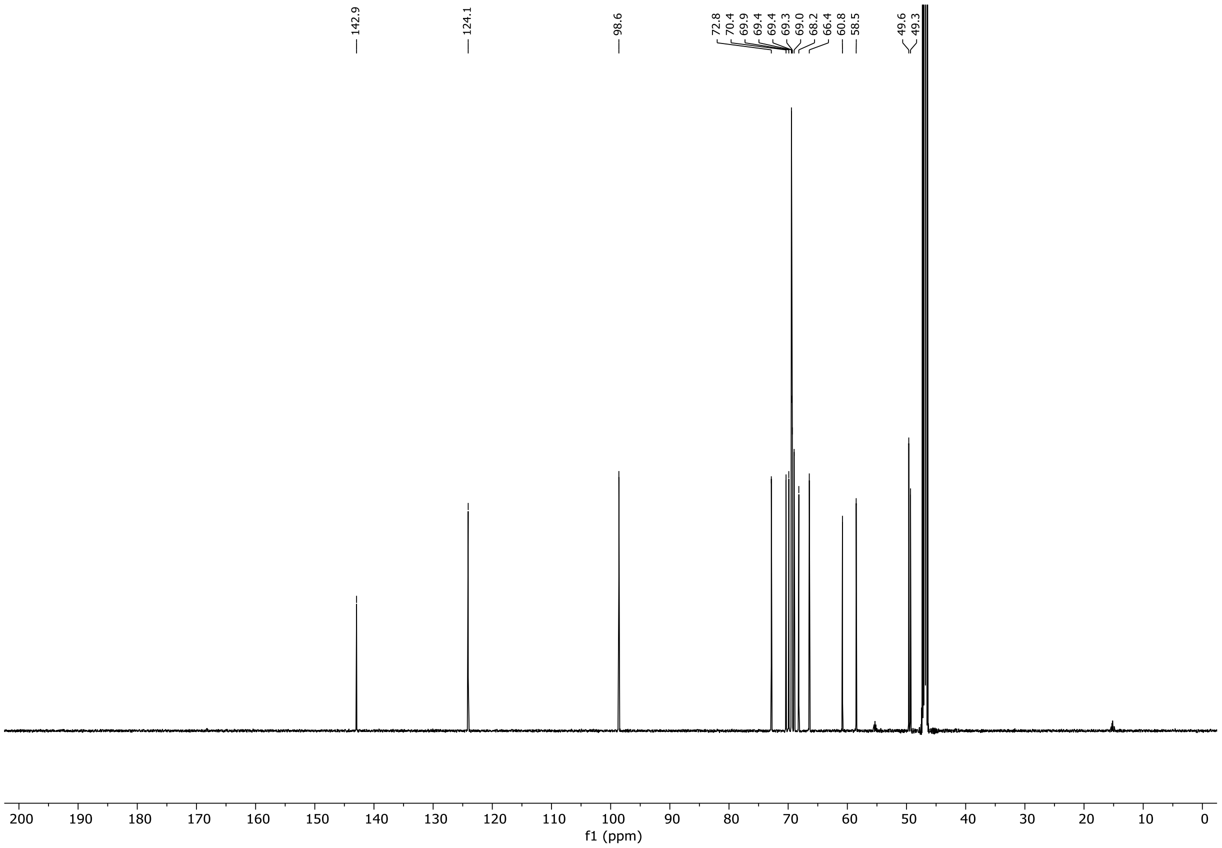

^{1}H-NMR, (600 MHz, $CDCl_3$): (**S17**)

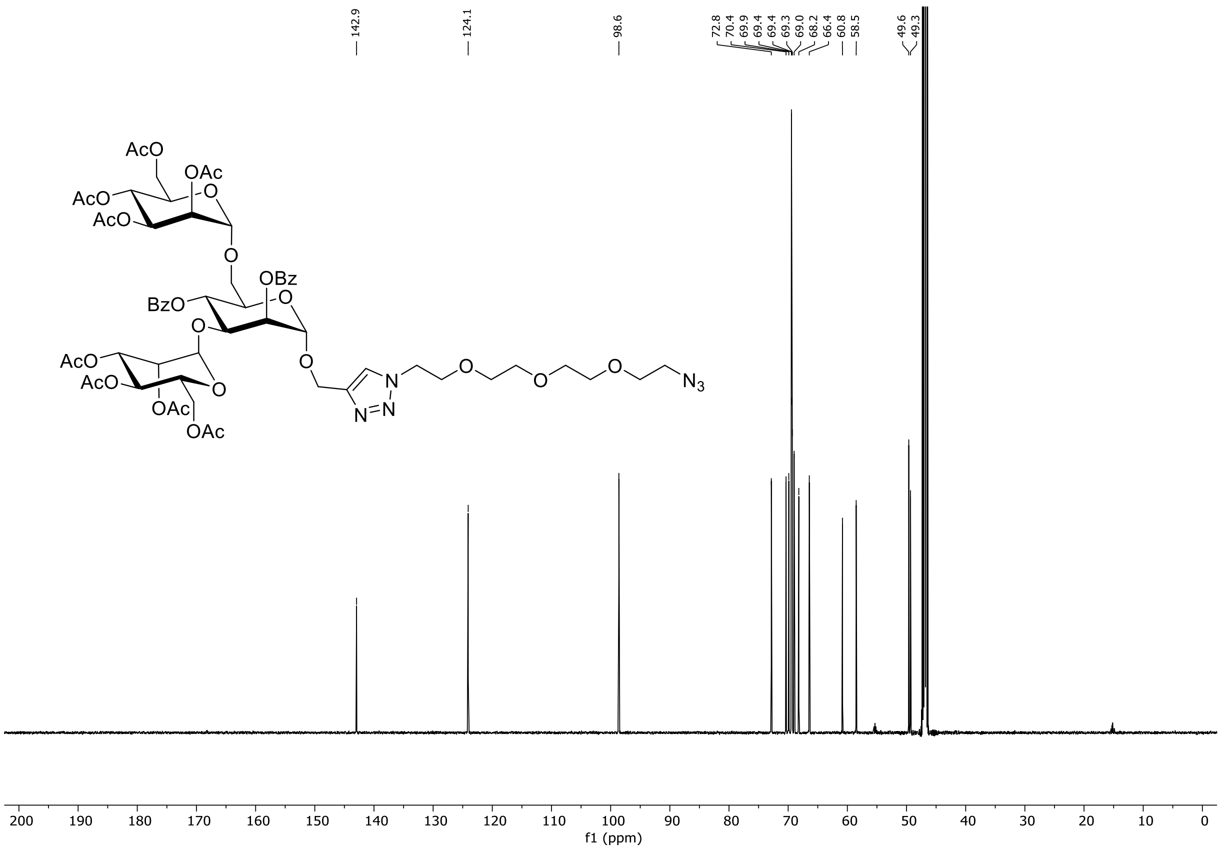

^{13}C-NMR, (151 MHz, $CDCl_3$): (**S17**)

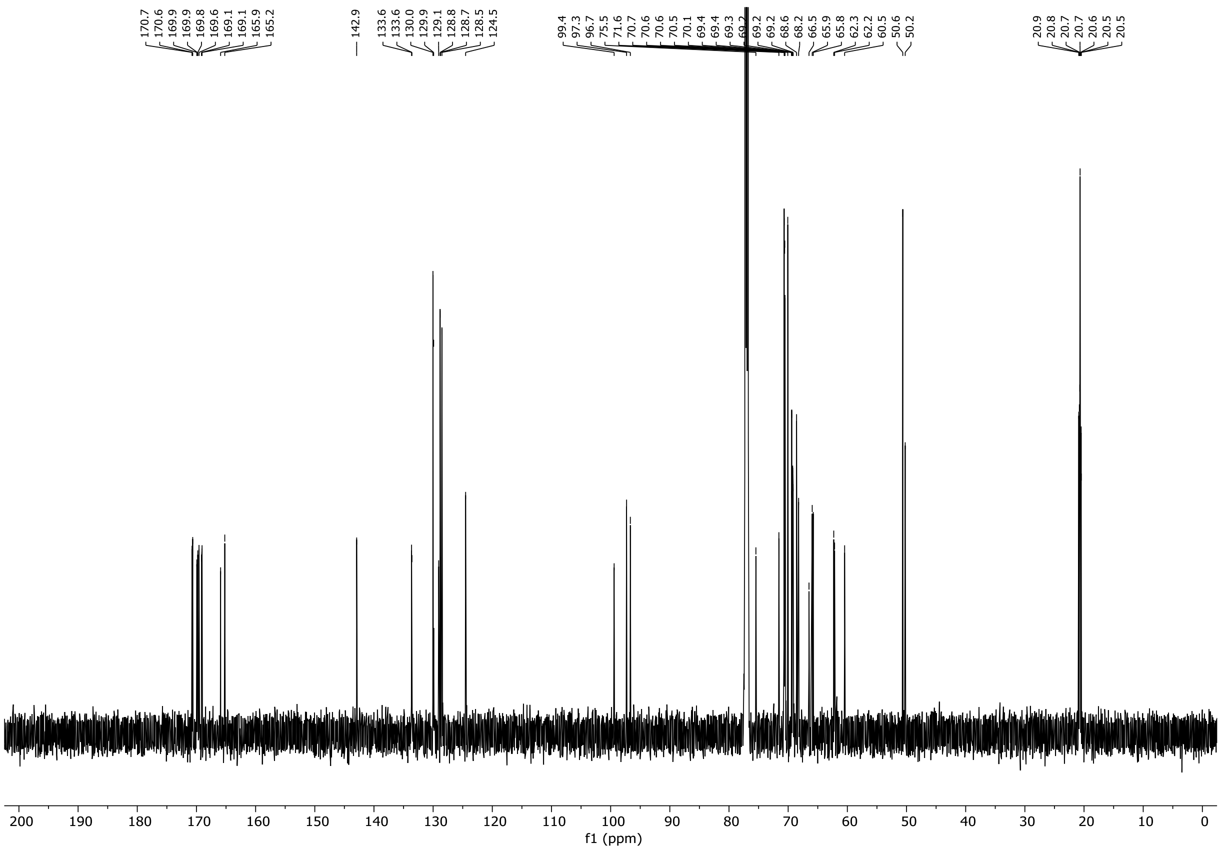

^{1}H-NMR, (600 MHz, D_2O): (**Man_3N_3**)

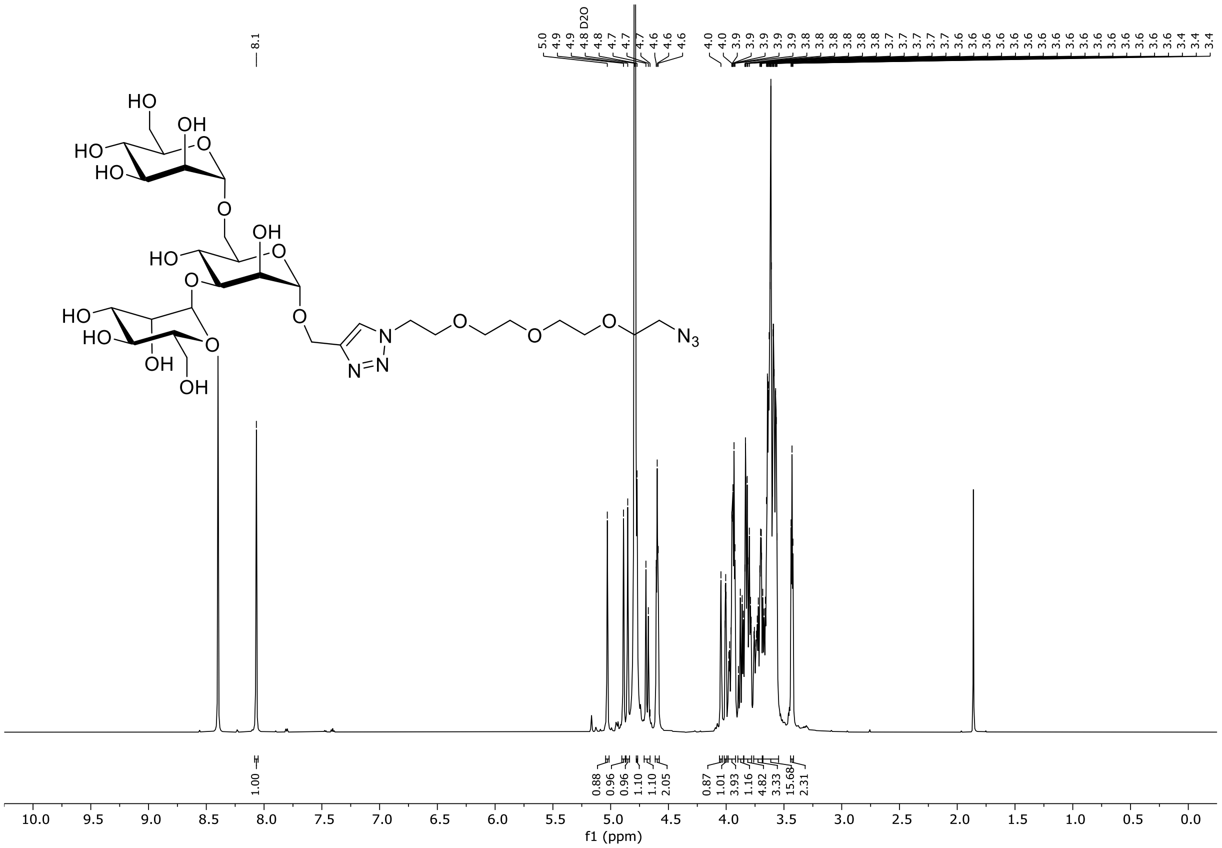

^{13}C-NMR, (151 MHz, D_2O): (**Man_3N_3**)

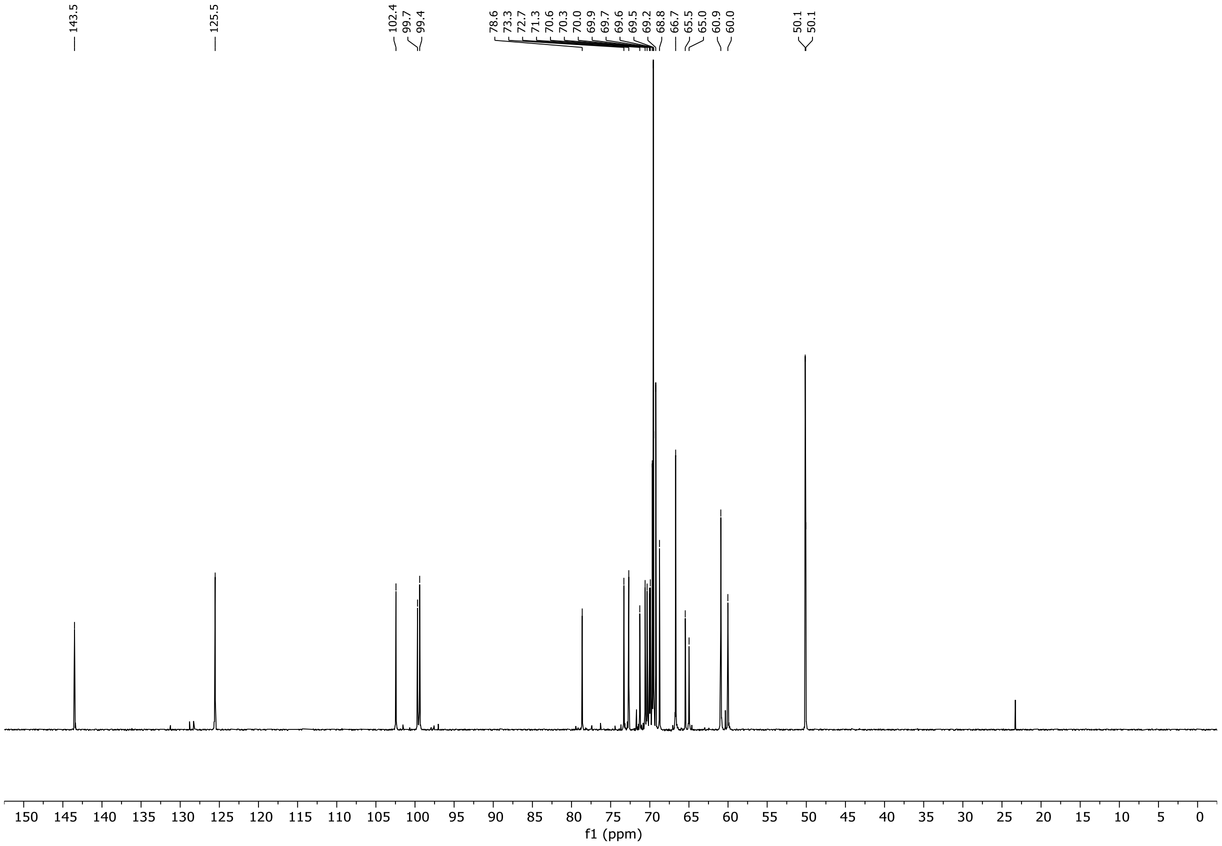

^{1}H-NMR, (600 MHz, $CDCl_3$): (**S8**)

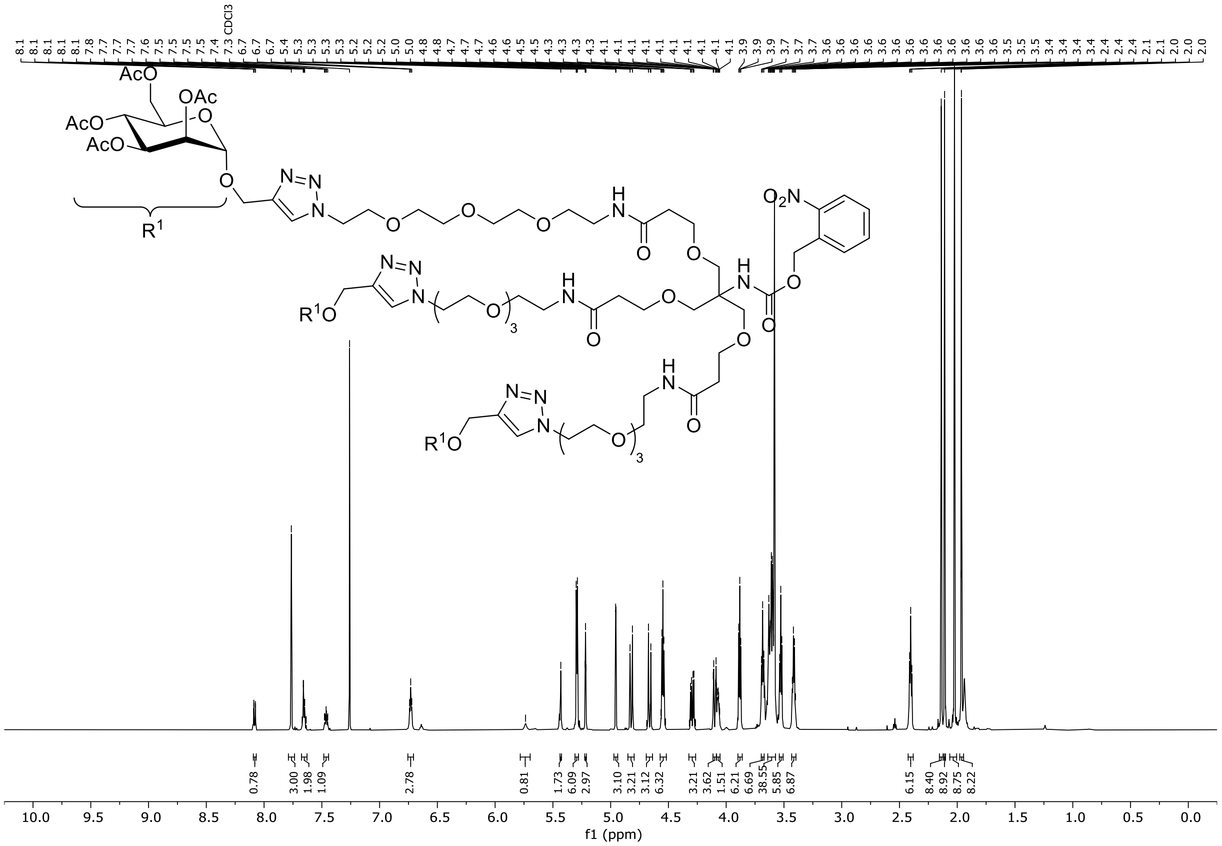

^{13}C-NMR, (151 MHz, $CDCl_3$): (**S8**)

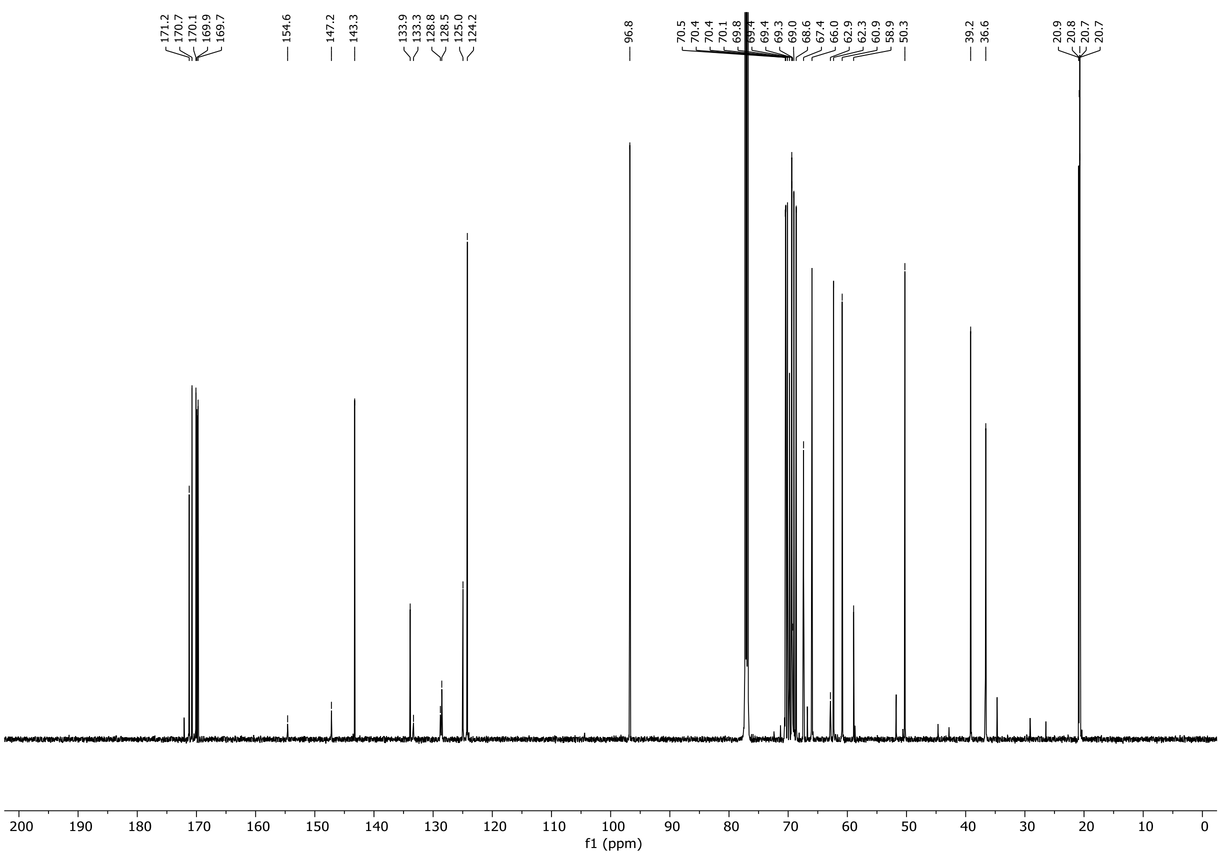

^{1}H-NMR, (600 MHz, $CDCl_3$): (**S10**)

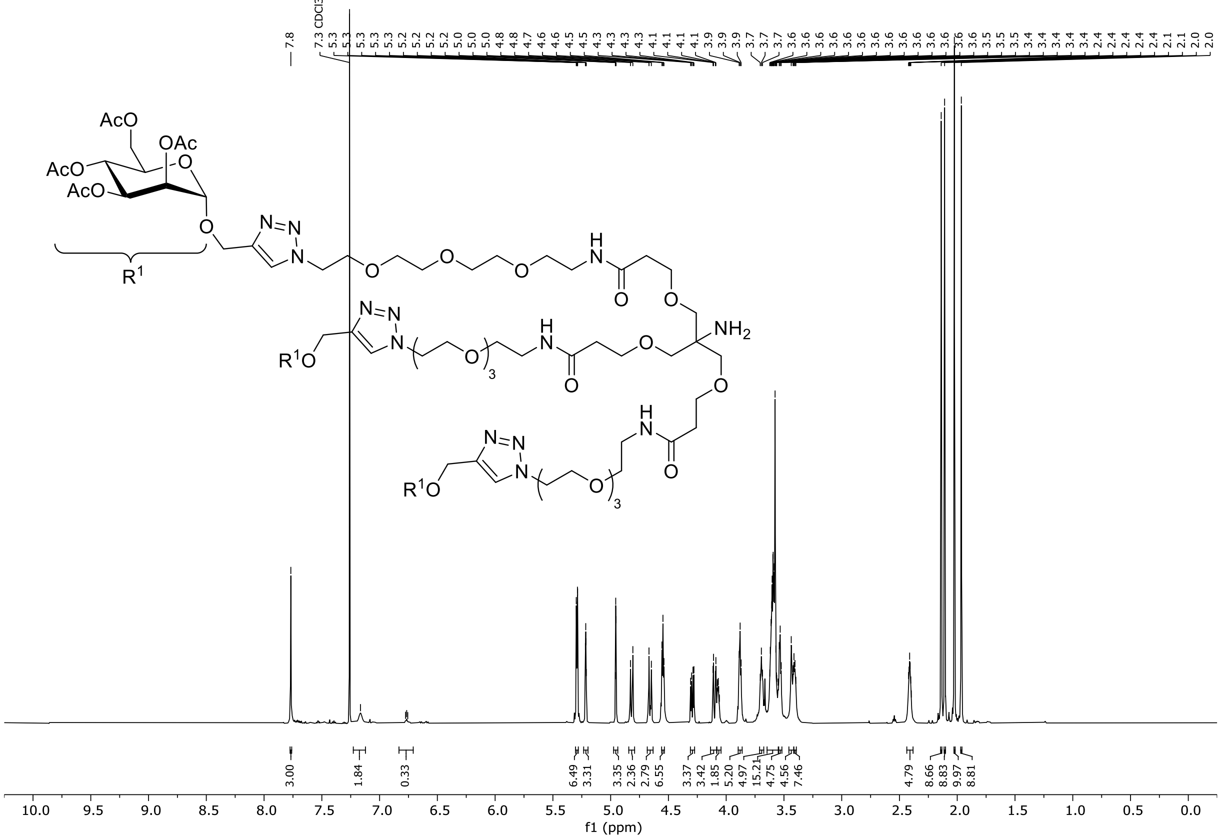

^{13}C-NMR, (151 MHz, $CDCl_3$): (**S10**)

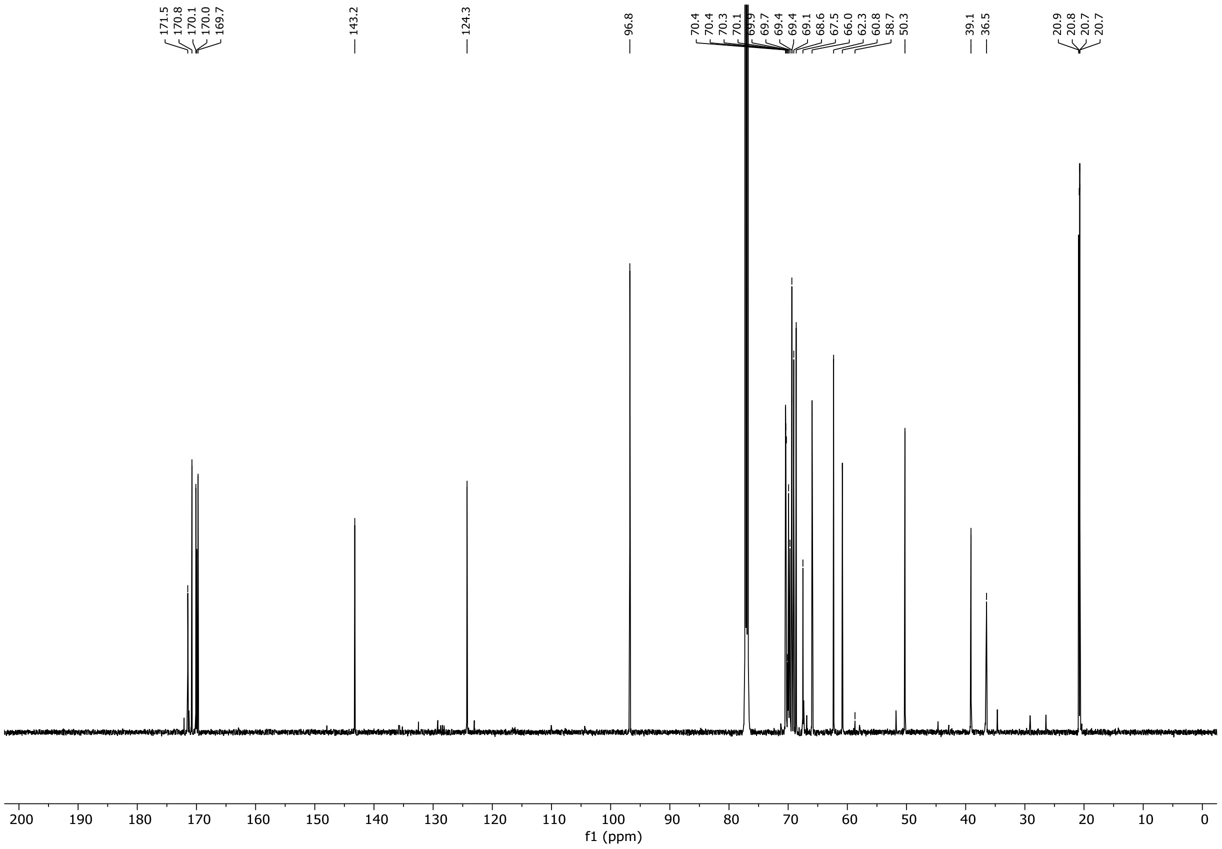

¹H-NMR, (600 MHz, $CDCl_3$): (**S12**)

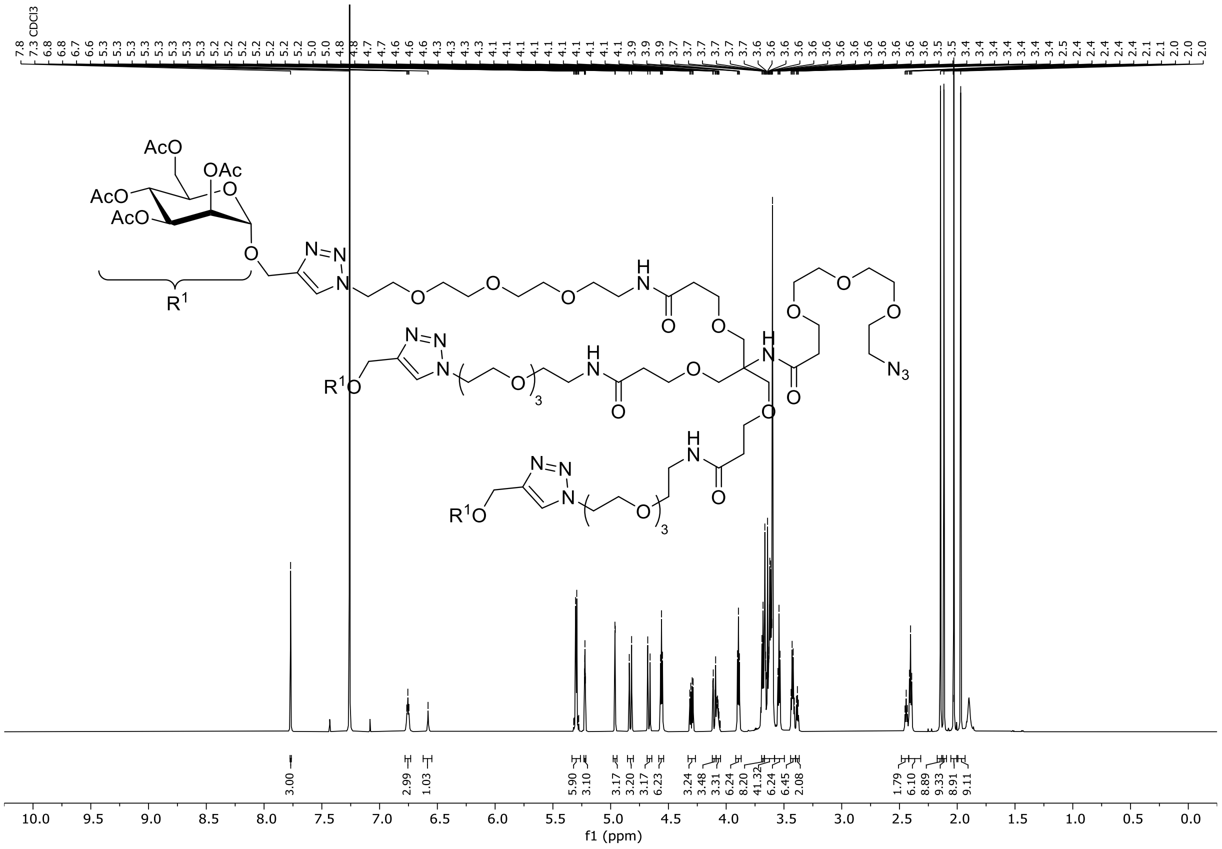

¹³C-NMR, (151 MHz, $CDCl_3$): (**S12**)

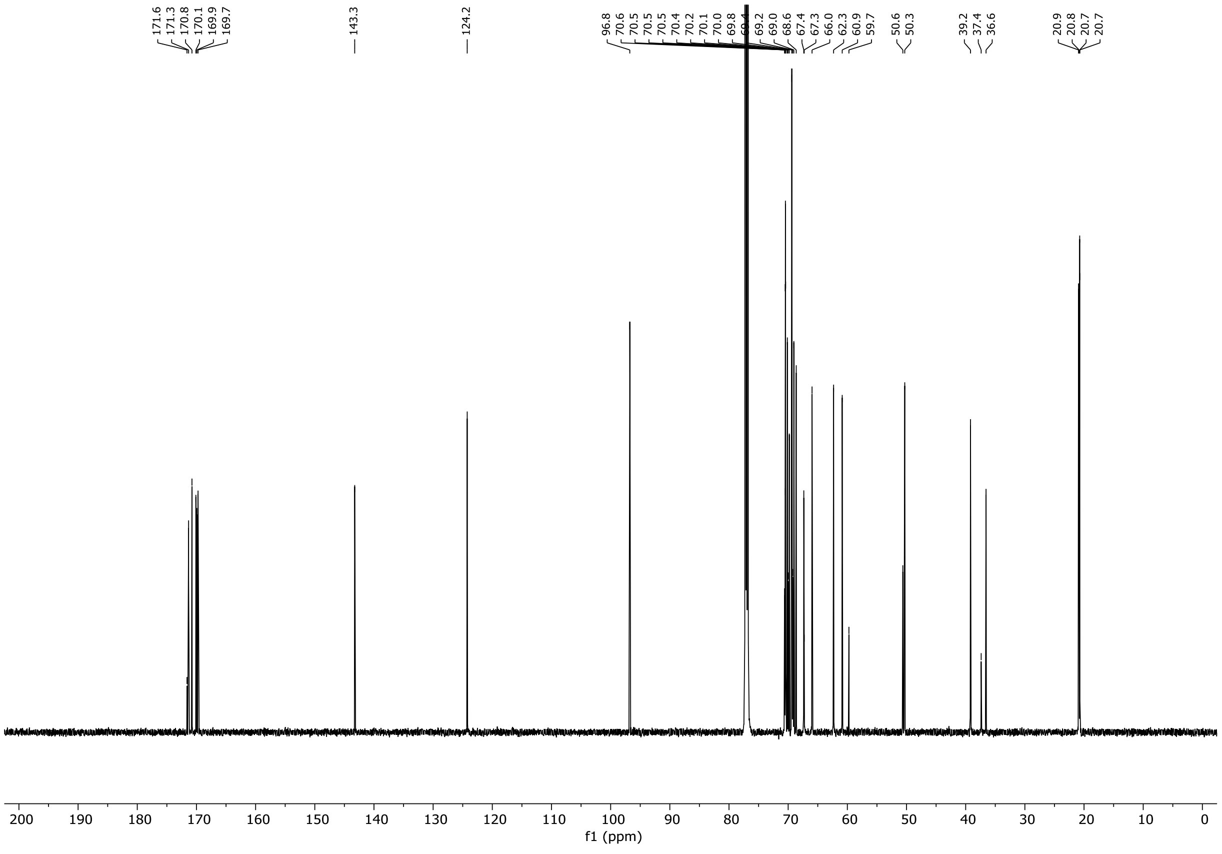

[1]**H-NMR**, (600 MHz, D2O): **(Man)3N3**

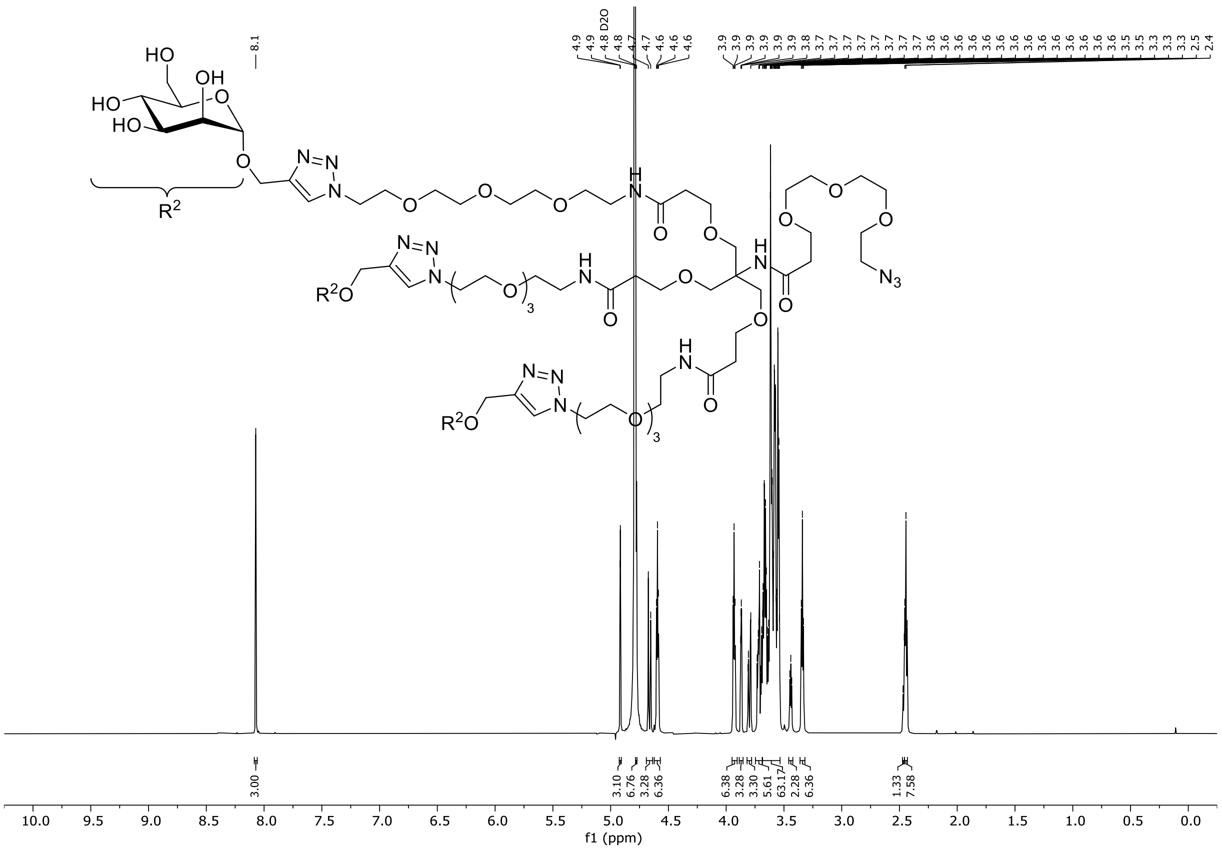

[13]**C-NMR**, (151 MHz, CDCl3): **(Man)3N3**

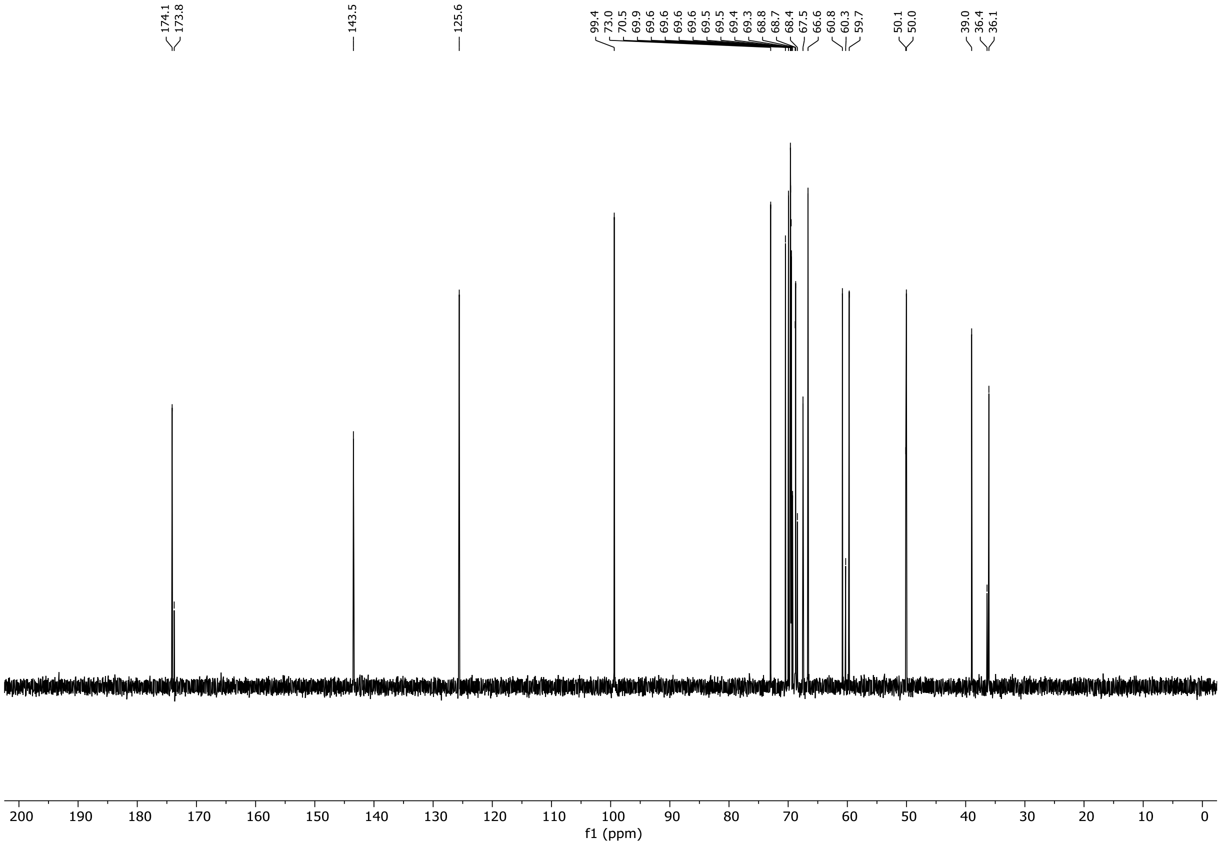

^{1}H-NMR, (600 MHz, $CDCl_3$): (**S9**)

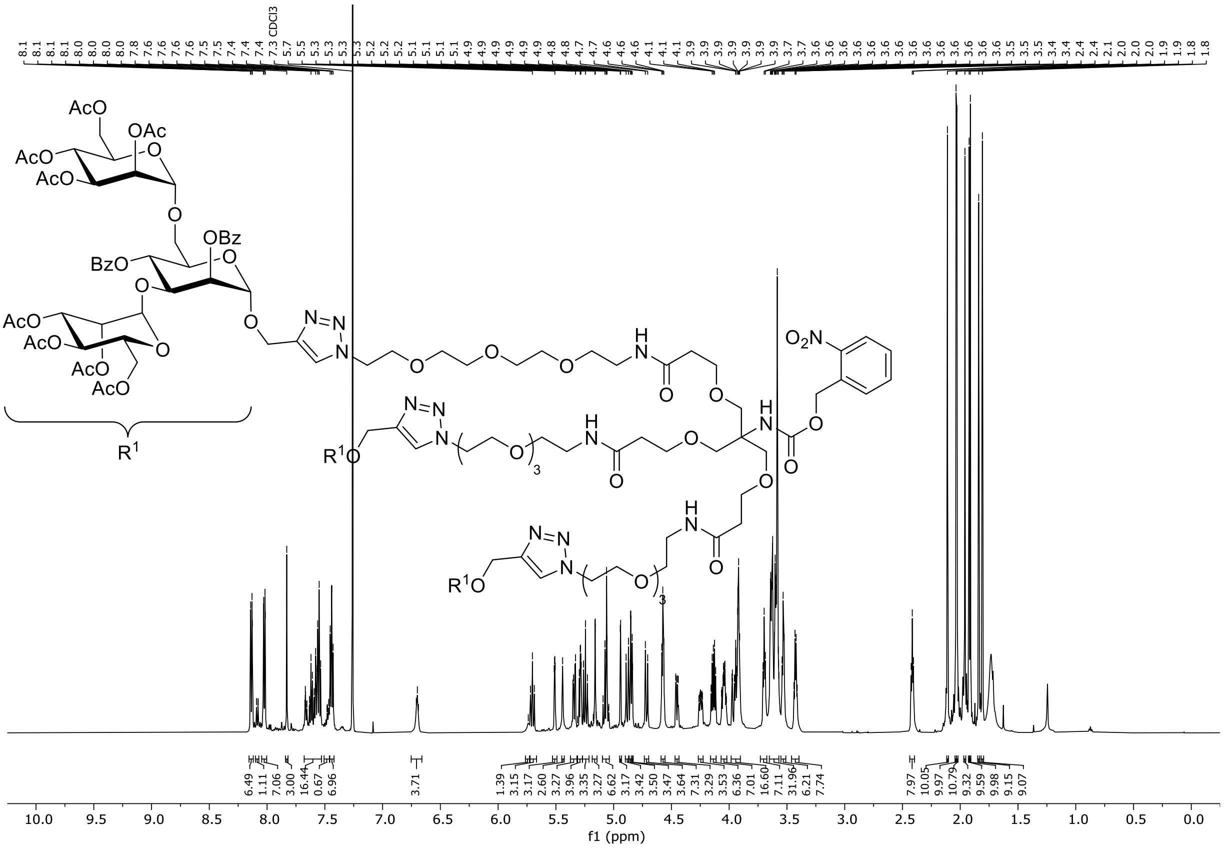

^{13}C-NMR, (151 MHz, $CDCl_3$): (**S9**)

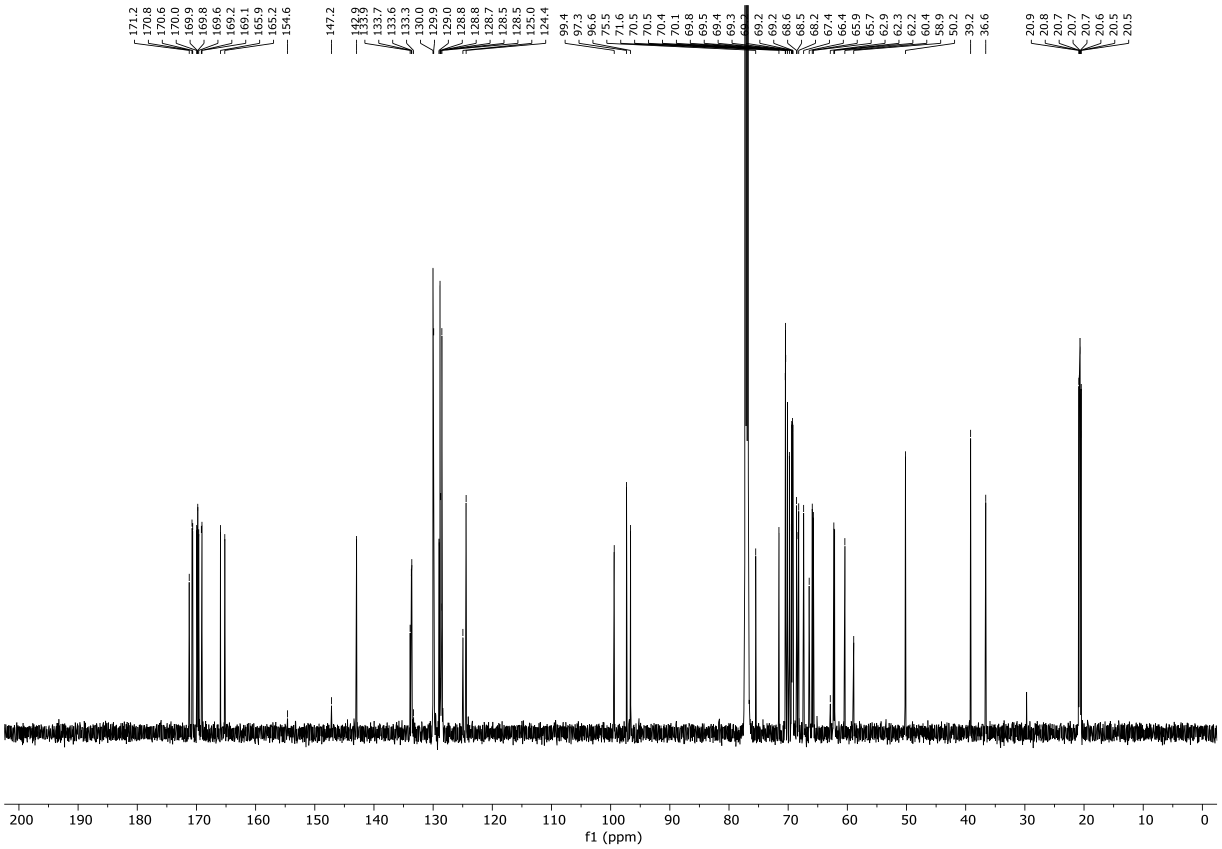

^{1}H-NMR, (600 MHz, $CDCl_3$): (**S11**)

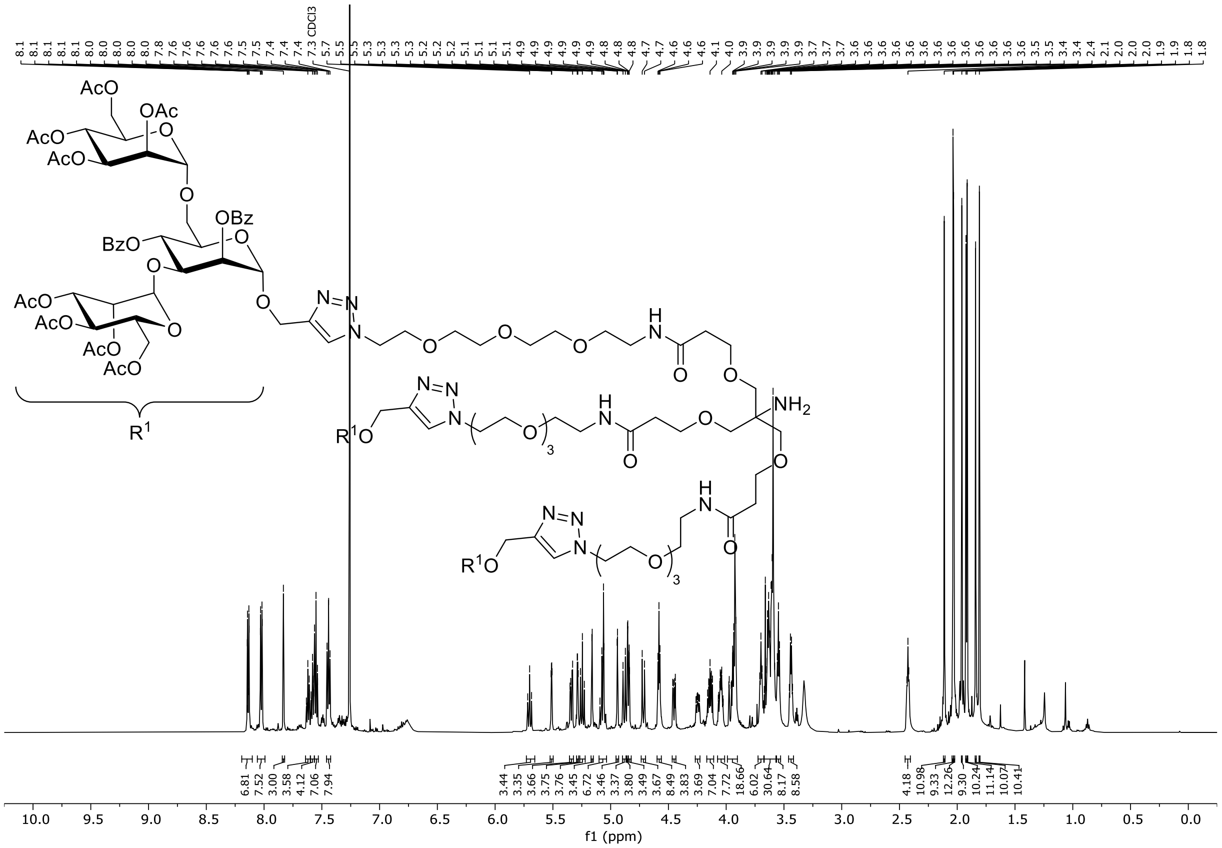

^{13}C-NMR, (151 MHz, $CDCl_3$): (**S11**)

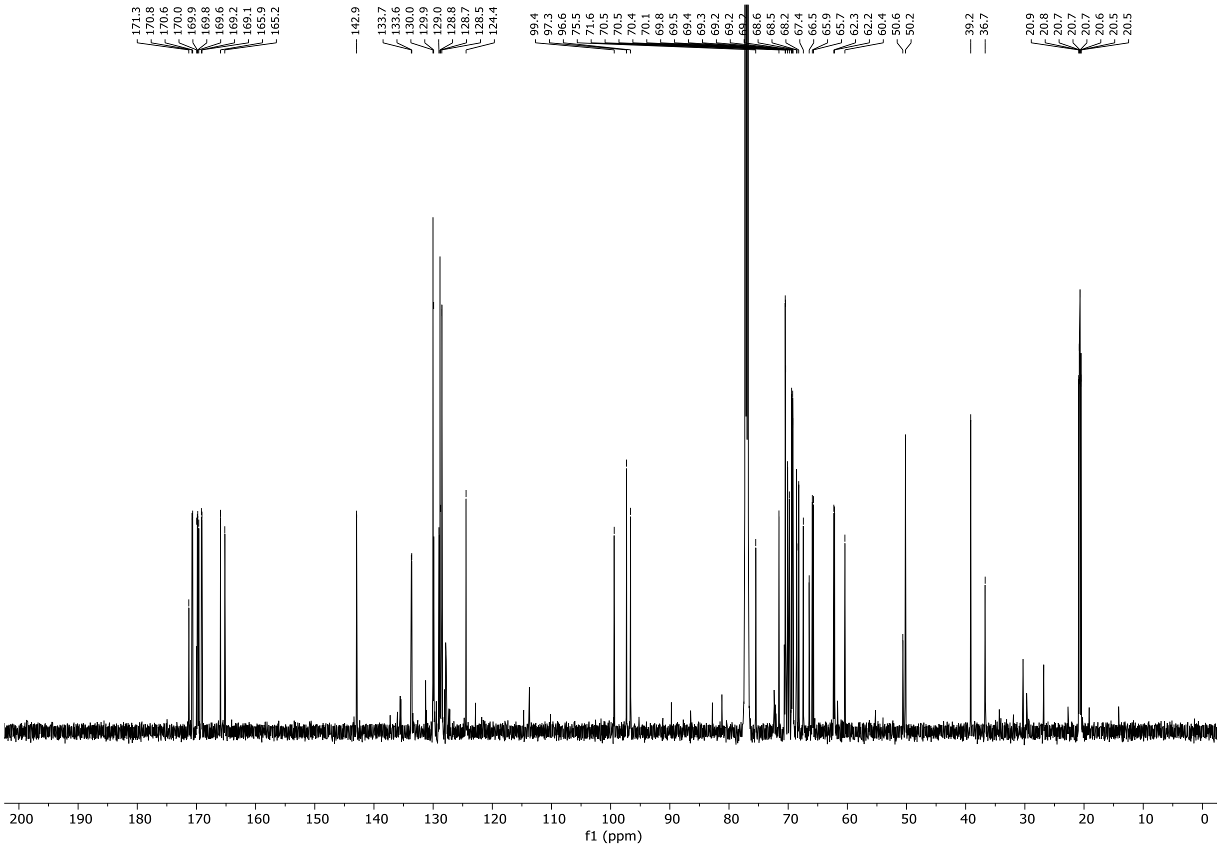

^{1}H-NMR, (600 MHz, $CDCl_3$): (**S13**)

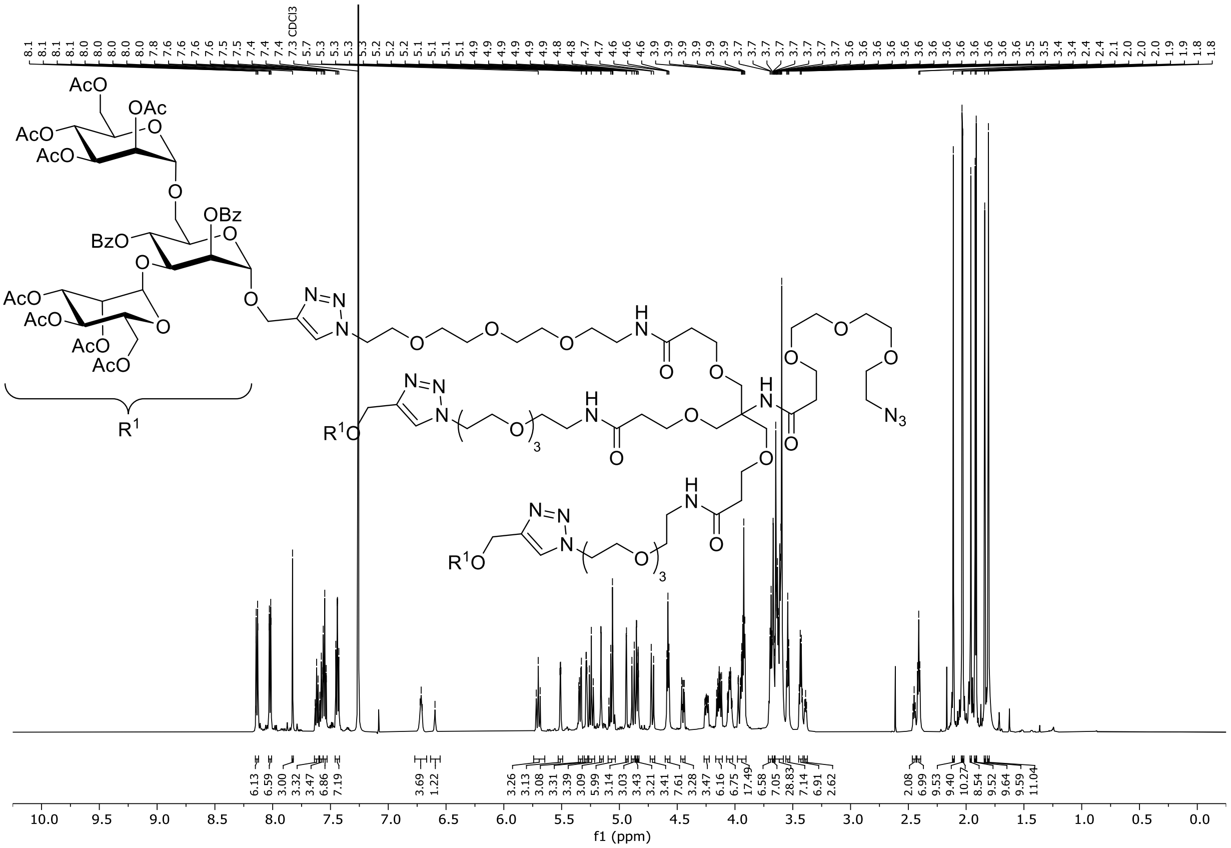

^{13}C-NMR, (151 MHz, $CDCl_3$): (**S13**)

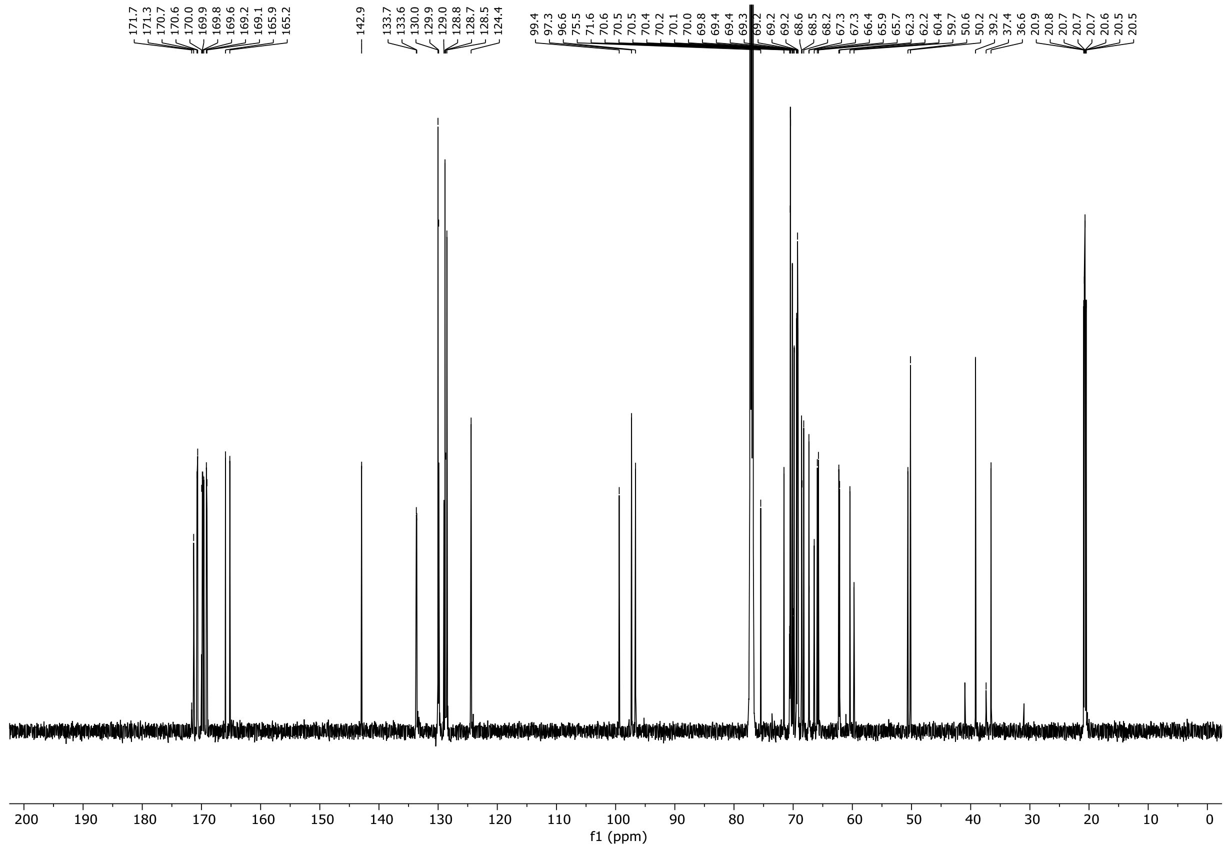

^{1}H-NMR, (600 MHz, $CDCl_3$): **$(Man_3)_3N_3$**

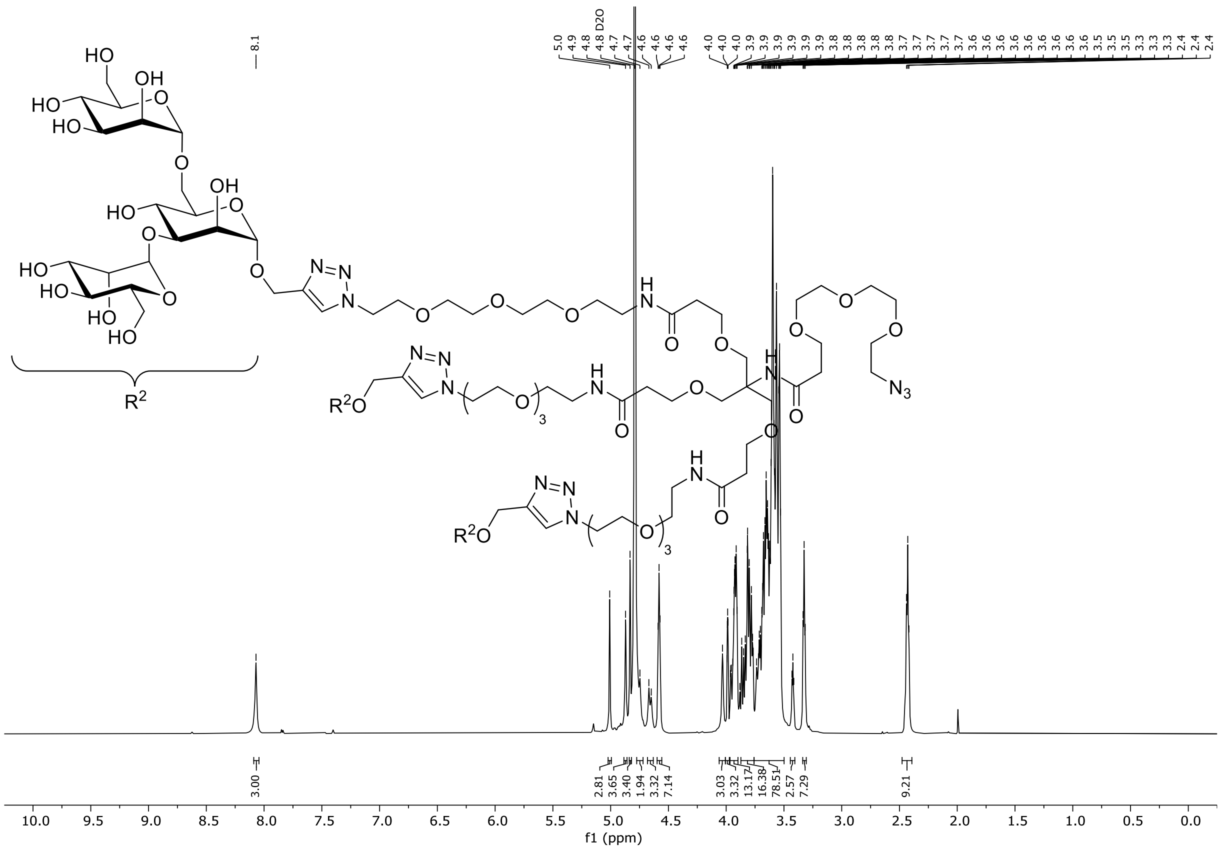

^{13}C-NMR, (151 MHz, $CDCl_3$): **$(Man_3)_3N_3$**

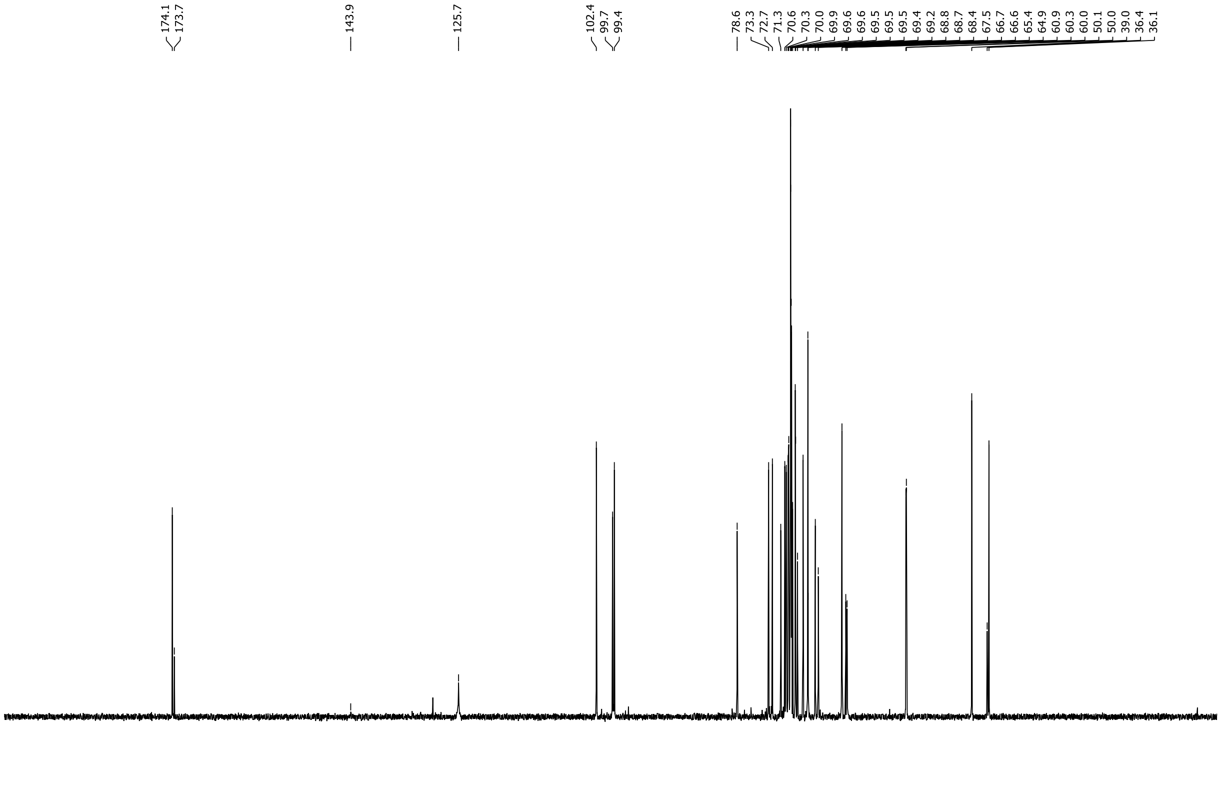

8.4 MALDI-ToF-MS spectra

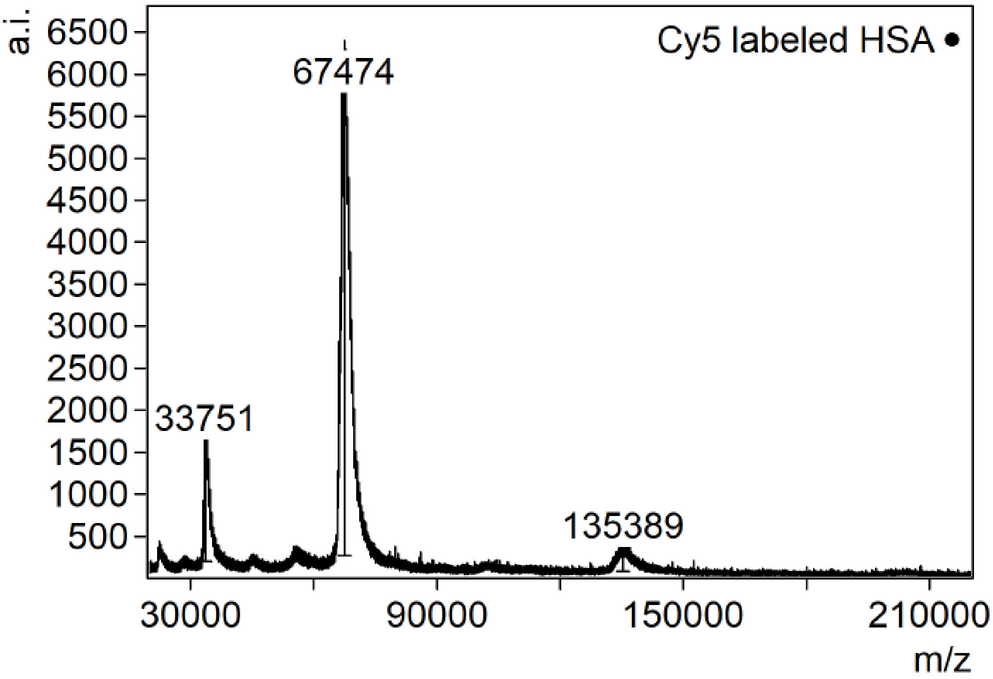

Figure S 9: MALDI-ToF-spectrum of Cy5-labeled HSA. Found m/z = 67474 $[M+H]^+$.

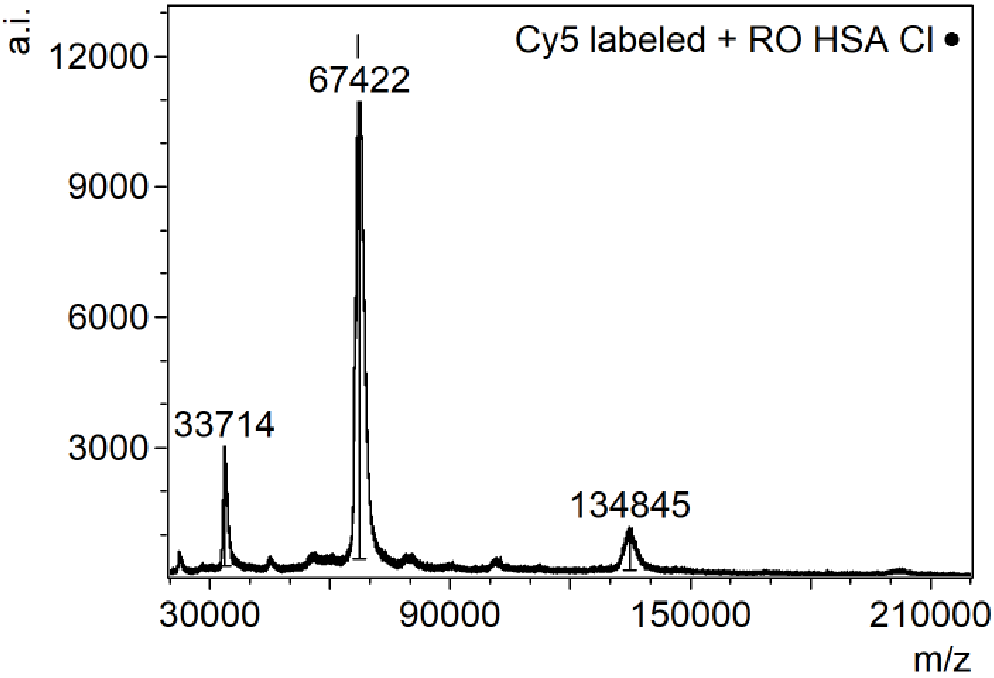

Figure S 10: MALDI-ToF-spectrum of Cy5-labeled HSA after maleimide ring opening (RO) (control conjugae C I). Found m/z = 67422 $[M+H]^+$.

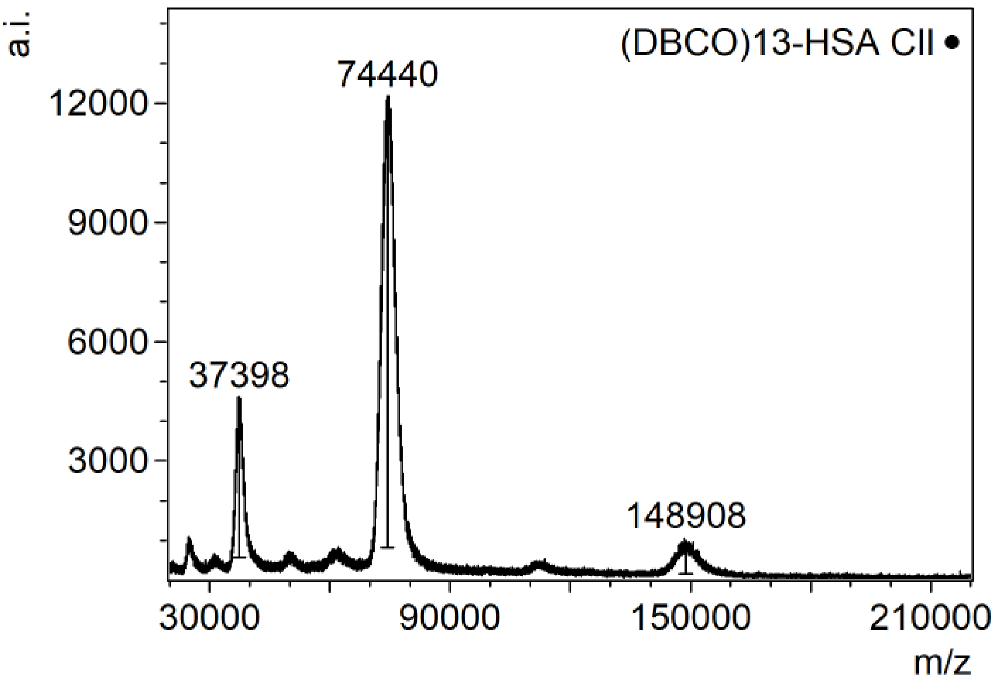

Figure S 11: MALDI-ToF-spectrum of DBCO-functionalized HSA (precursor and control conjugate C II). Found *m/z* = 74440 $[M+H]^+$.

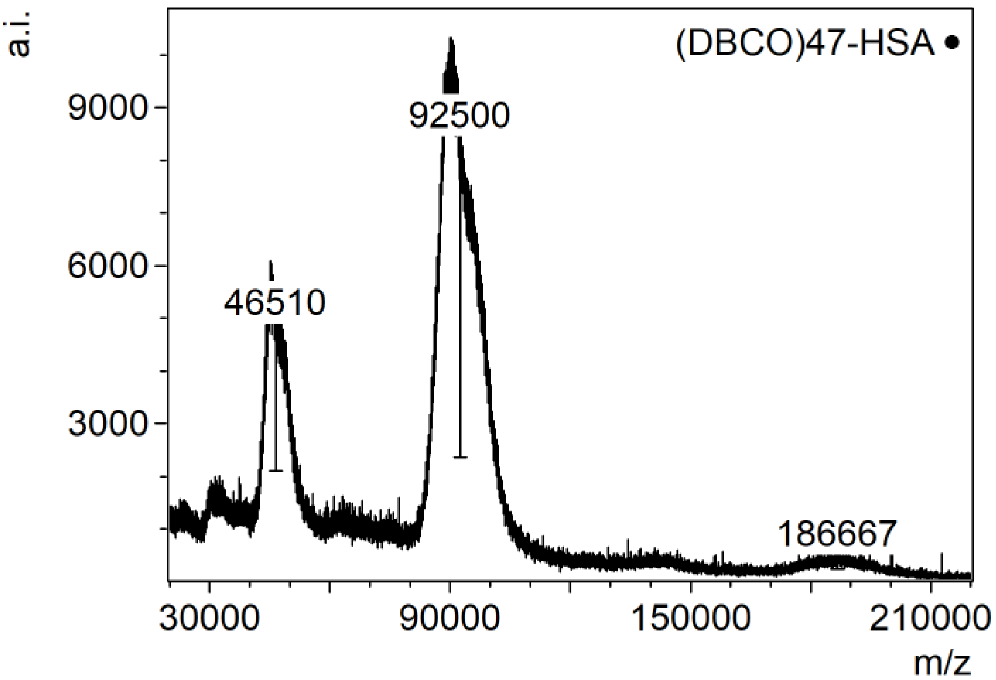

Figure S 12: MALDI-ToF-spectrum of DBCO-functionalized HSA (precursor conjugate). Found *m/z* = 92500 $[M+H]^+$.

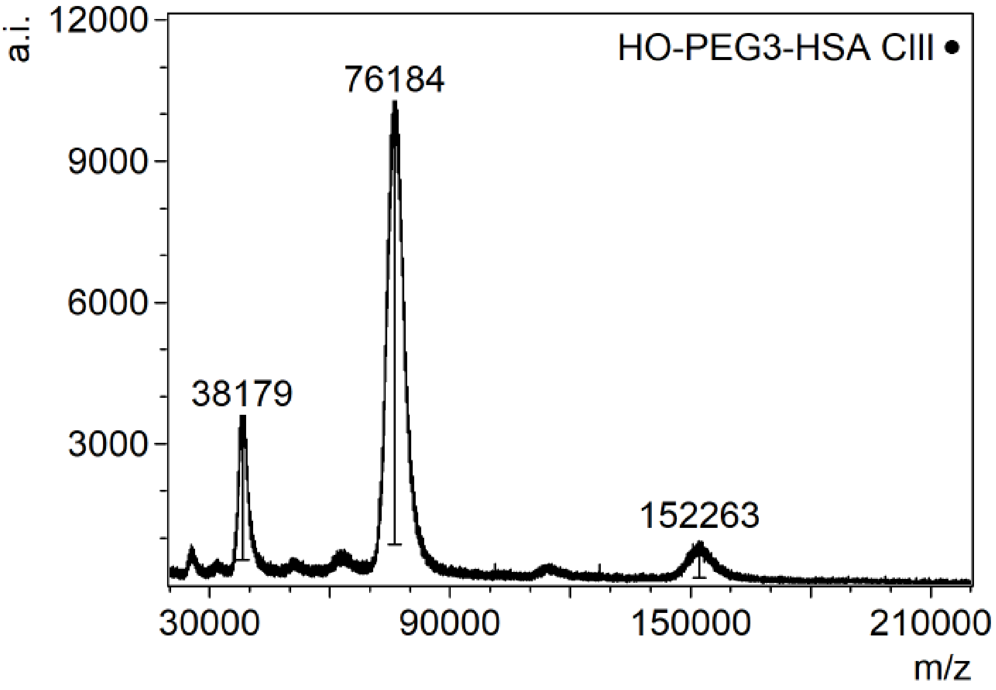

Figure S 13: MALDI-ToF-spectrum of triethylenglycol-functionalized HSA (control conjugate C III). Found *m/z* = 76184 [M+H]$^+$.

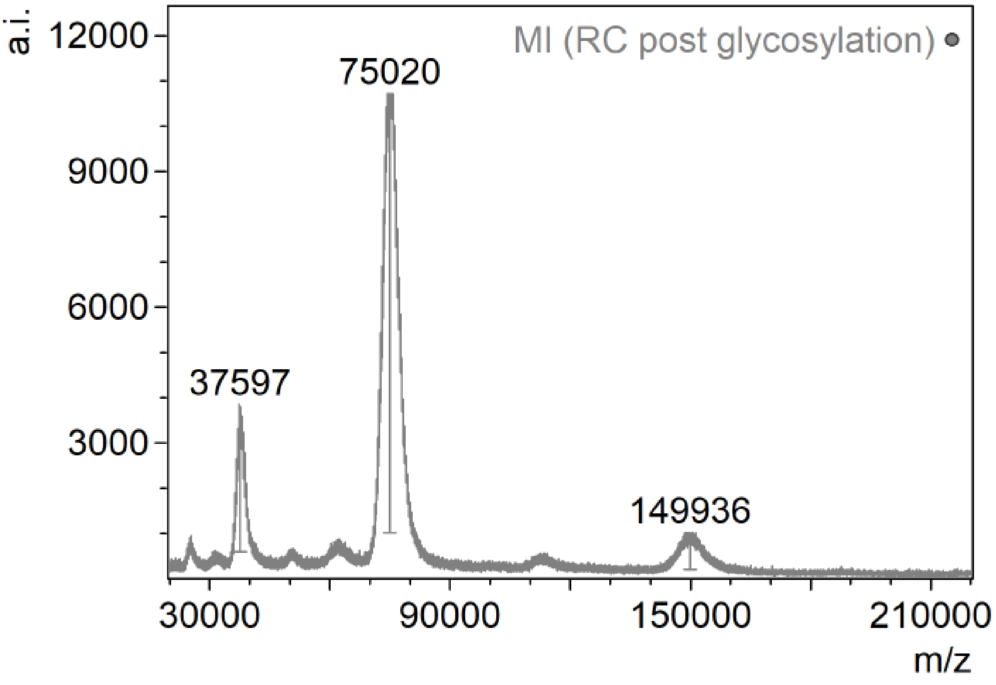

Figure S 14: MALDI-ToF-spectrum as reaction control (RC) of Man-functionalized HSA after glycosylation. Found *m/z* = 75020 [M+H]$^+$.

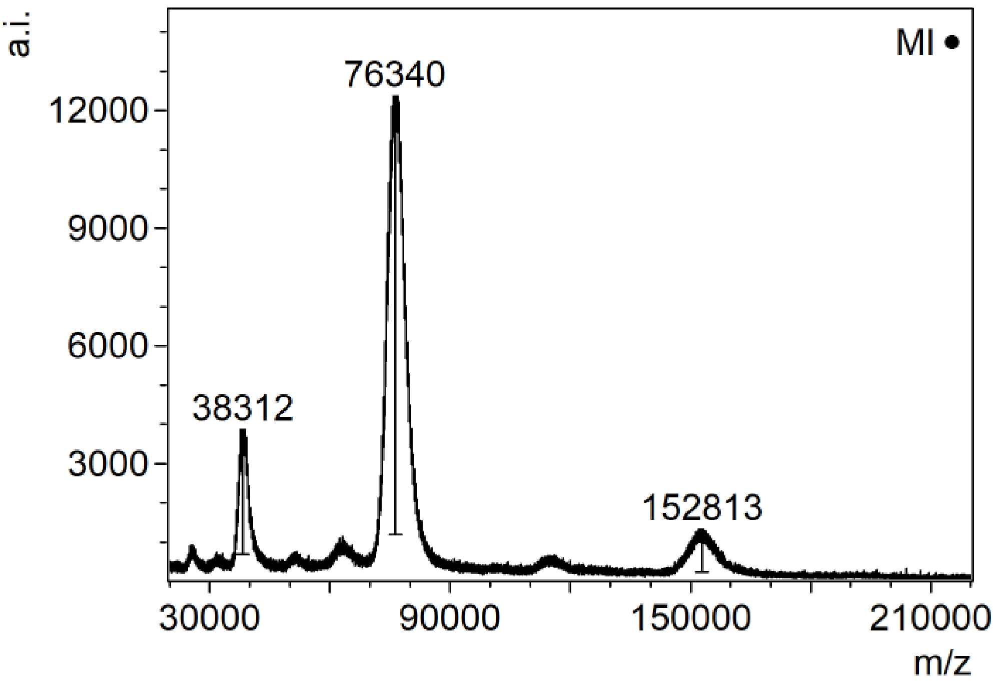

Figure S 15: MALDI-ToF-spectrum of capped, Man-functionalized HSA (conjugate M I). Found *m/z* = 76340 $[M+H]^+$.

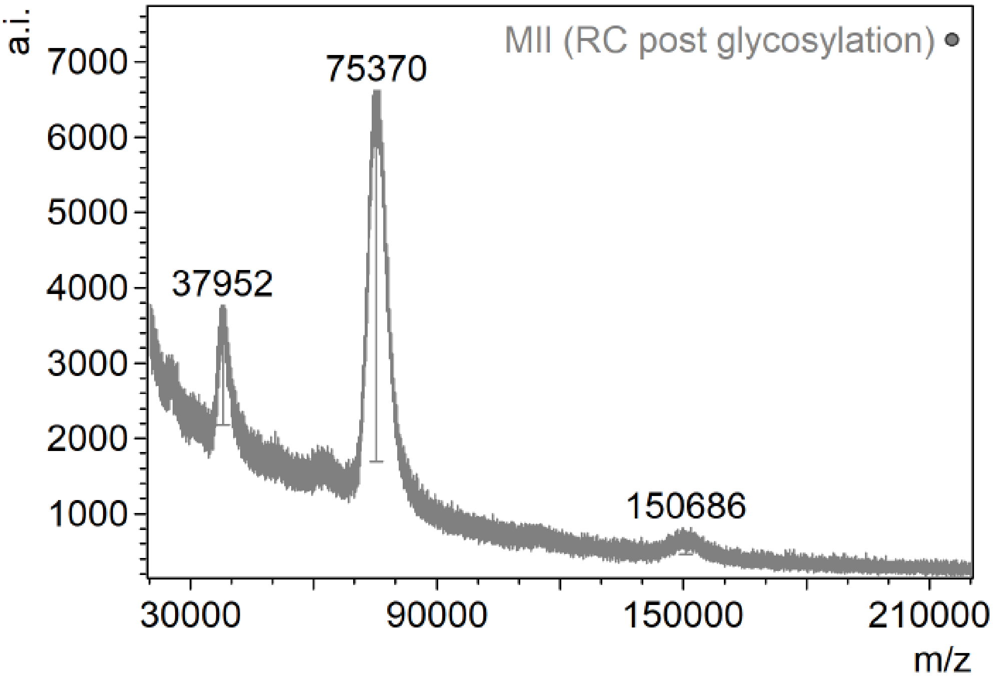

Figure S 16: MALDI-ToF-spectrum as reaction control (RC) of Man-functionalized HSA after glycosylation. Found *m/z* = 75370 $[M+H]^+$.

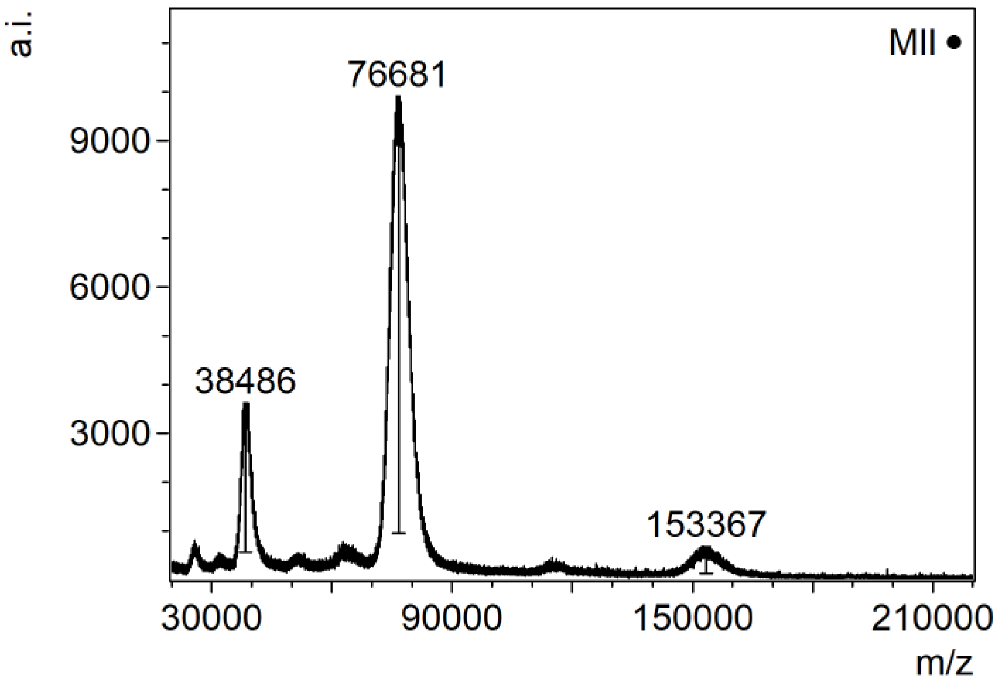

Figure S 17: MALDI-ToF-spectrum of capped, Man-functionalized HSA (conjugate M II). Found m/z = 76681 $[M+H]^+$.

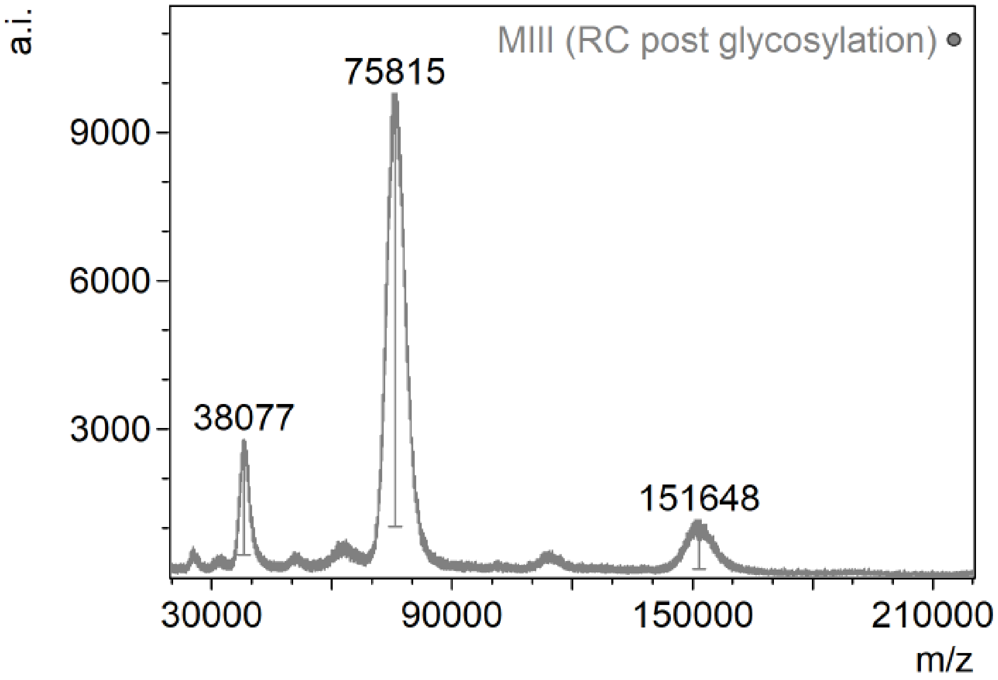

Figure S 18: MALDI-ToF-spectrum as reaction control (RC) of Man-functionalized HSA after glycosylation. Found m/z = 75815 $[M+H]^+$.

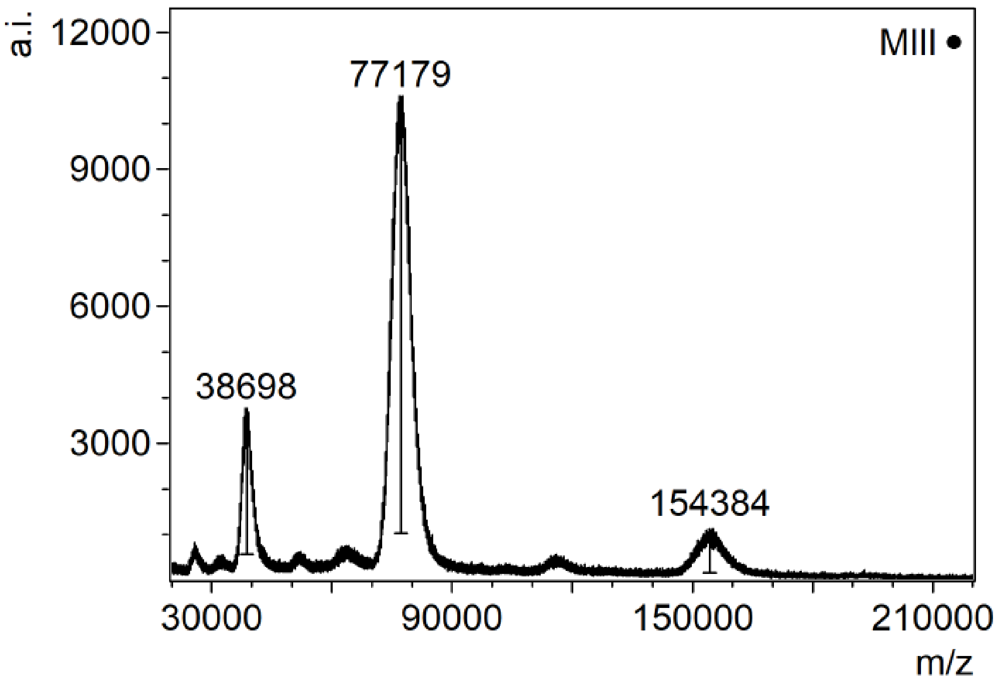

Figure S 19: MALDI-ToF-spectrum of capped, Man-functionalized HSA (conjugate M III). Found *m/z* = 77179 $[M+H]^+$.

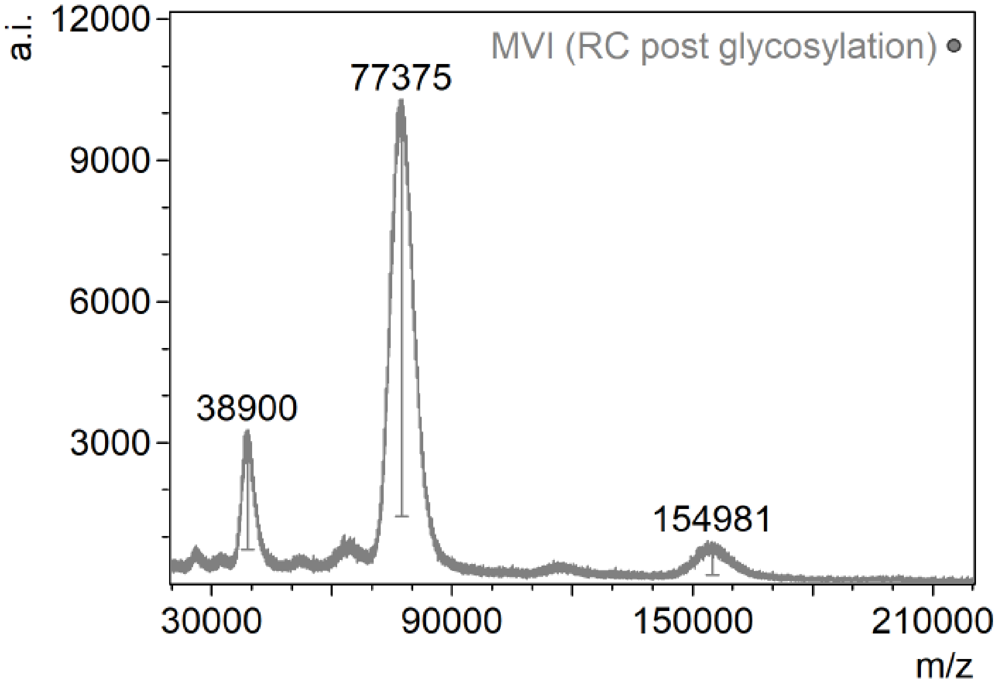

Figure S 20: MALDI-ToF-spectrum as reaction control (RC) of Man-functionalized HSA after glycosylation. Found *m/z* = 77375 $[M+H]^+$.

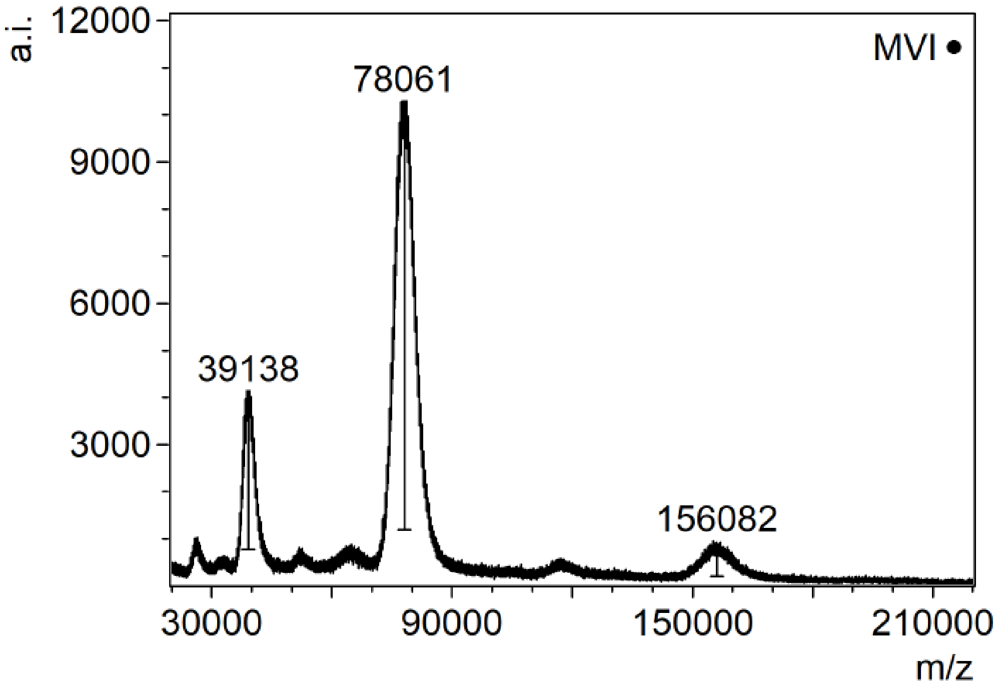

Figure S 21: MALDI-ToF-spectrum of capped, Man-functionalized HSA (conjugate M VI). Found *m/z* = 78061 $[M+H]^{+}$.

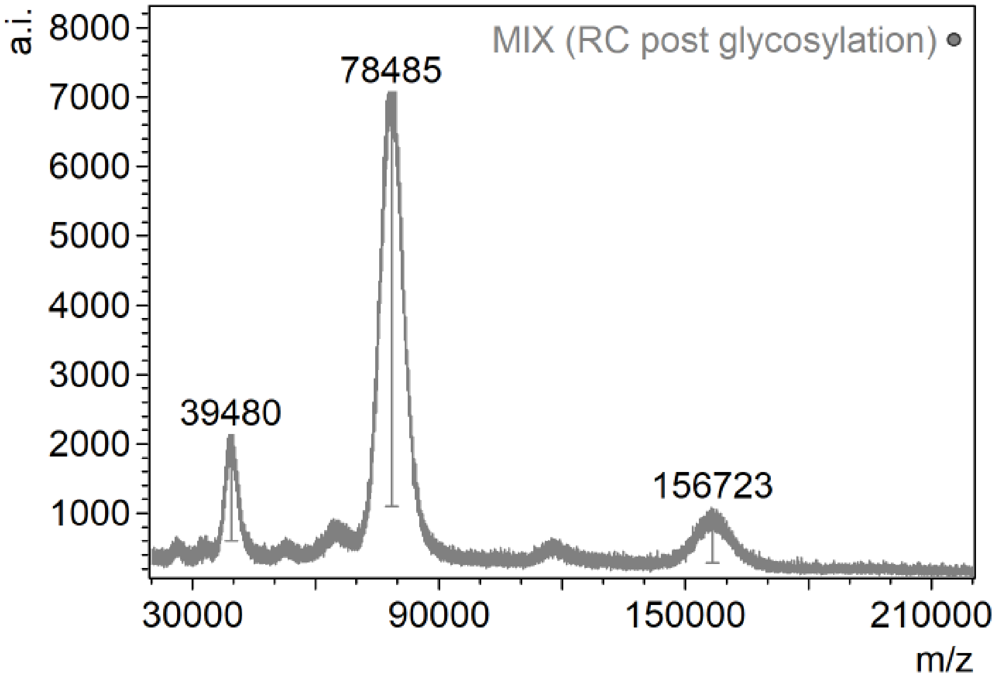

Figure S 22: MALDI-ToF-spectrum as reaction control (RC) of Man-functionalized HSA after glycosylation. Found *m/z* = 78485 $[M+H]^{+}$.

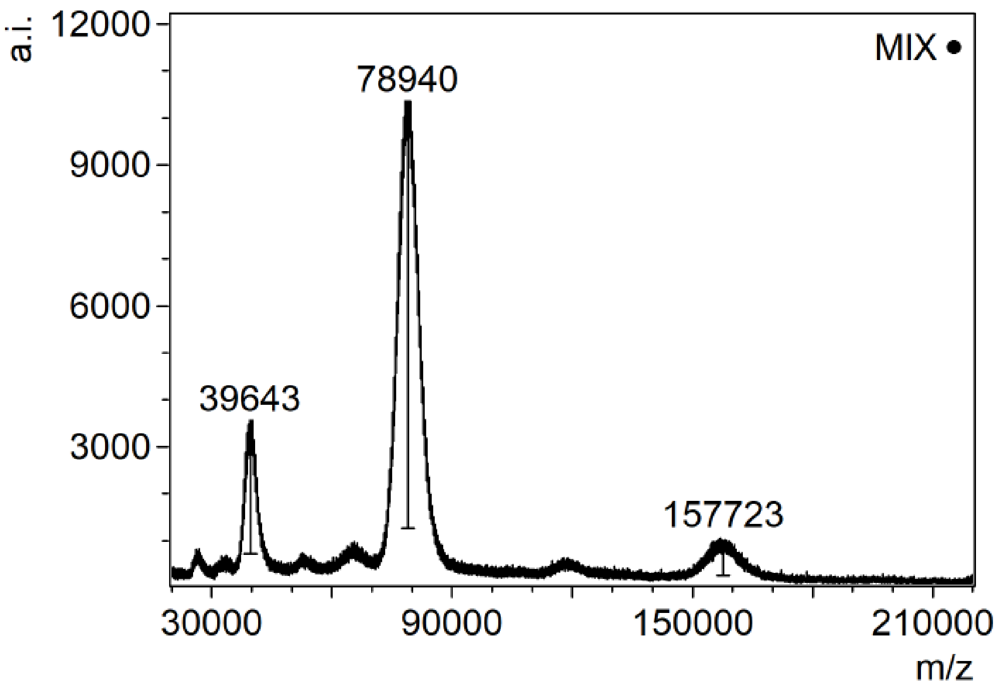

Figure S 23: MALDI-ToF-spectrum of capped, Man-functionalized HSA (conjugate M IX). Found *m/z* = 78940 [M+H]+.

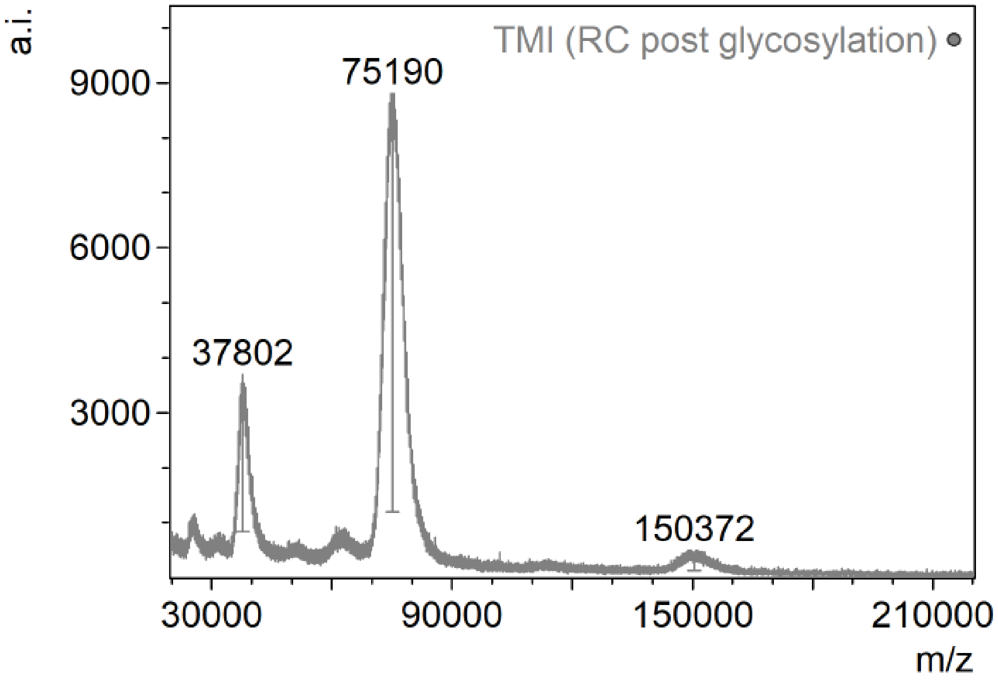

Figure S 24: MALDI-ToF-spectrum as reaction control (RC) of Man_3-functionalized HSA after glycosylation. Found *m/z* = 75190 [M+H]+.

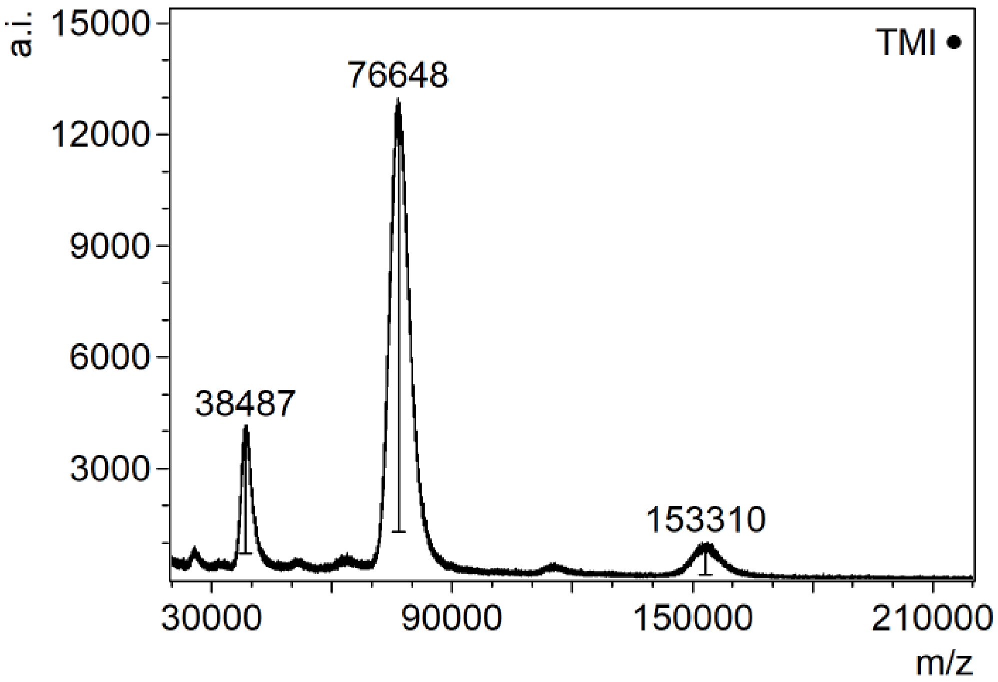

Figure S 25: MALDI-ToF-spectrum of capped, Man_3-functionalized HSA (conjugate TM I). Found m/z = 76648 $[M+H]^+$.

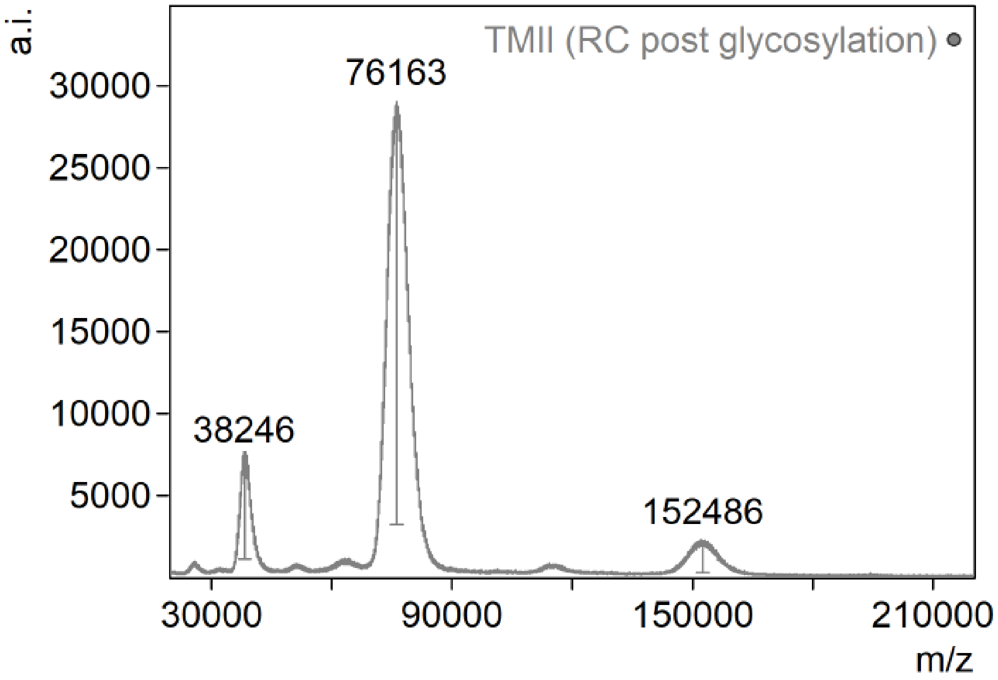

Figure S 26: MALDI-ToF-spectrum as reaction control (RC) of Man_3-functionalized HSA after glycosylation. Found m/z = 76163 $[M+H]^+$.

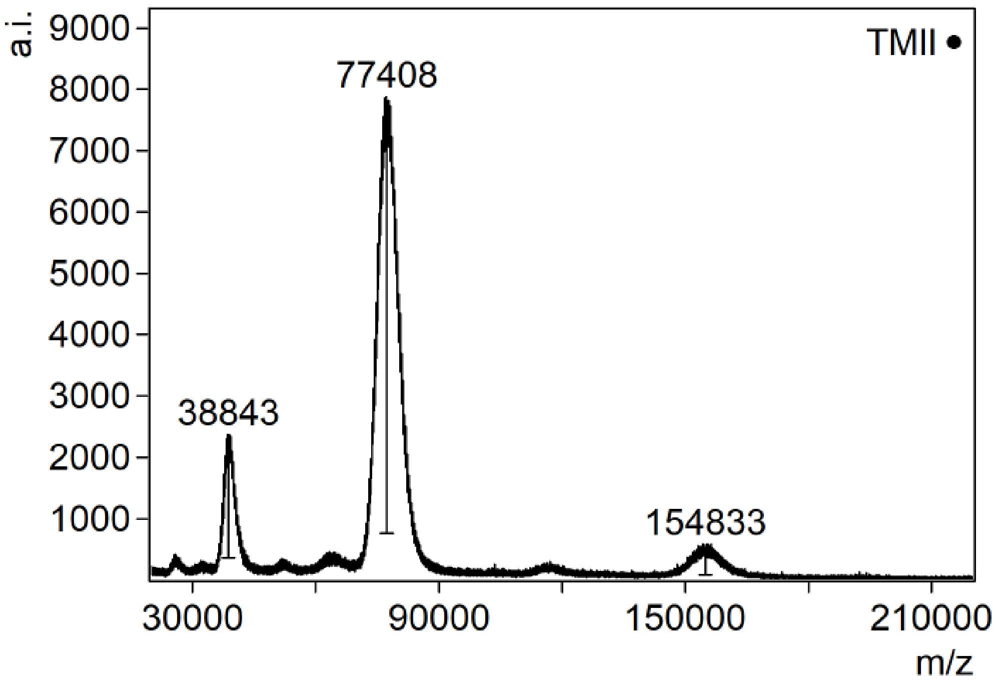

Figure S 27: MALDI-ToF-spectrum of capped, Man_3-functionalized HSA (conjugate TM II). Found *m/z* = 77408 $[M+H]^+$.

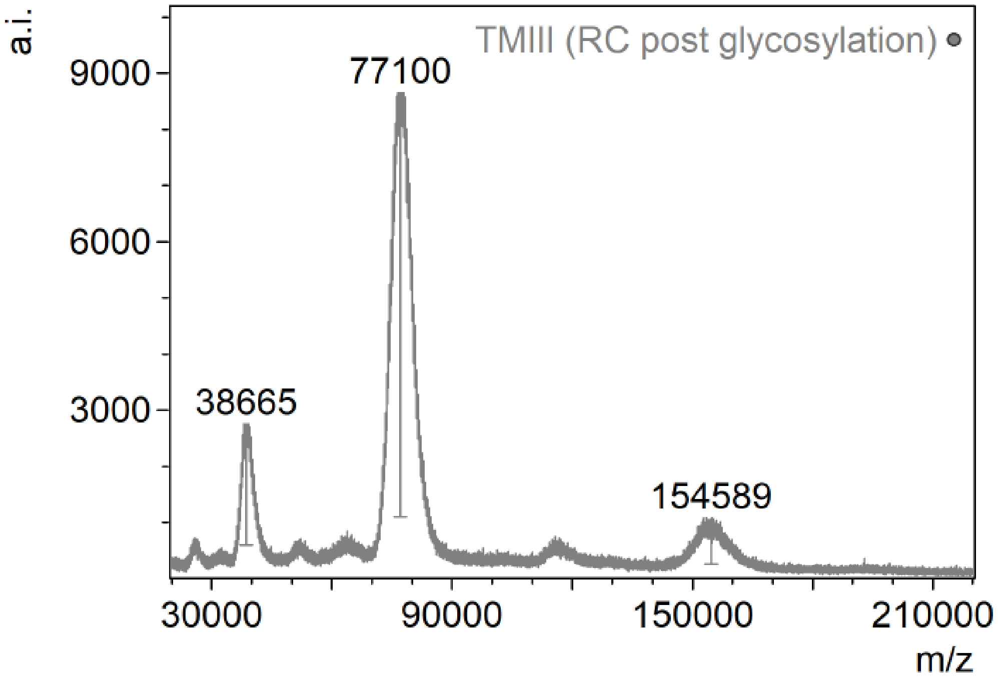

Figure S 28: MALDI-ToF-spectrum as reaction control (RC) of Man_3-functionalized HSA after glycosylation. Found *m/z* = 77100 $[M+H]^+$.

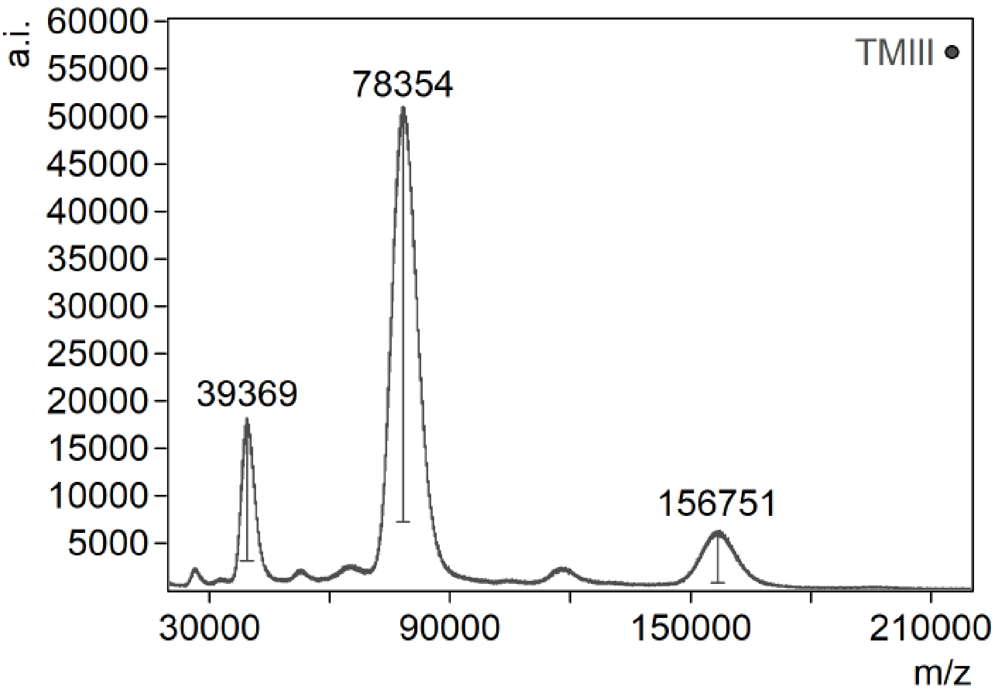

Figure S 29: MALDI-ToF-spectrum of capped, Man_3-functionalized HSA (conjugate TM III). Found *m/z* = 78354 $[M+H]^+$.

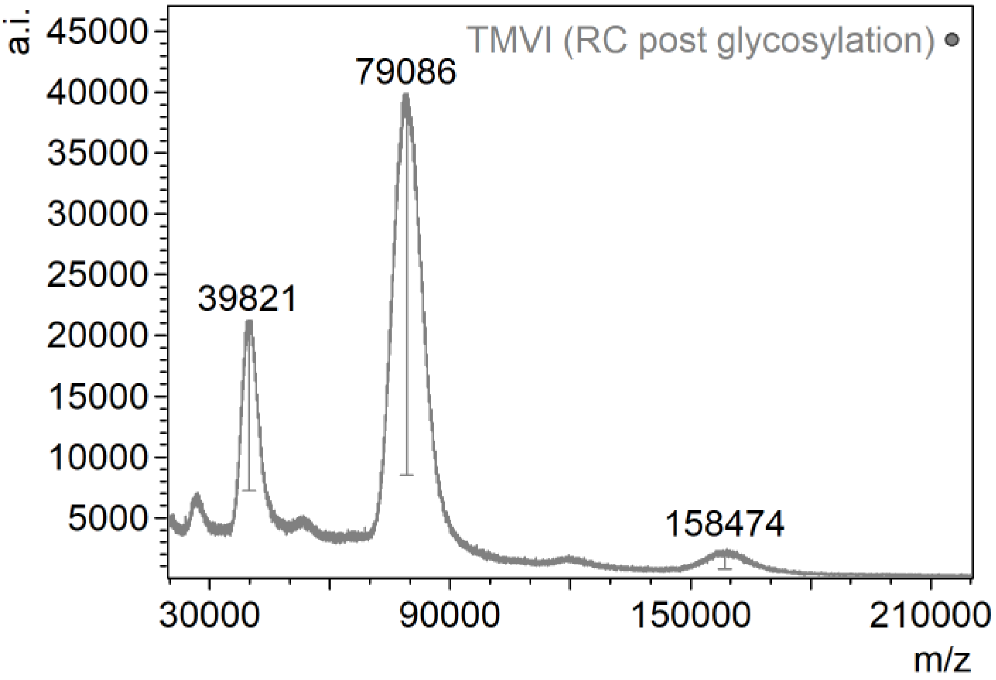

Figure S 30: MALDI-ToF-spectrum as reaction control (RC) of Man_3-functionalized HSA after glycosylation. Found *m/z* = 79086 $[M+H]^+$.

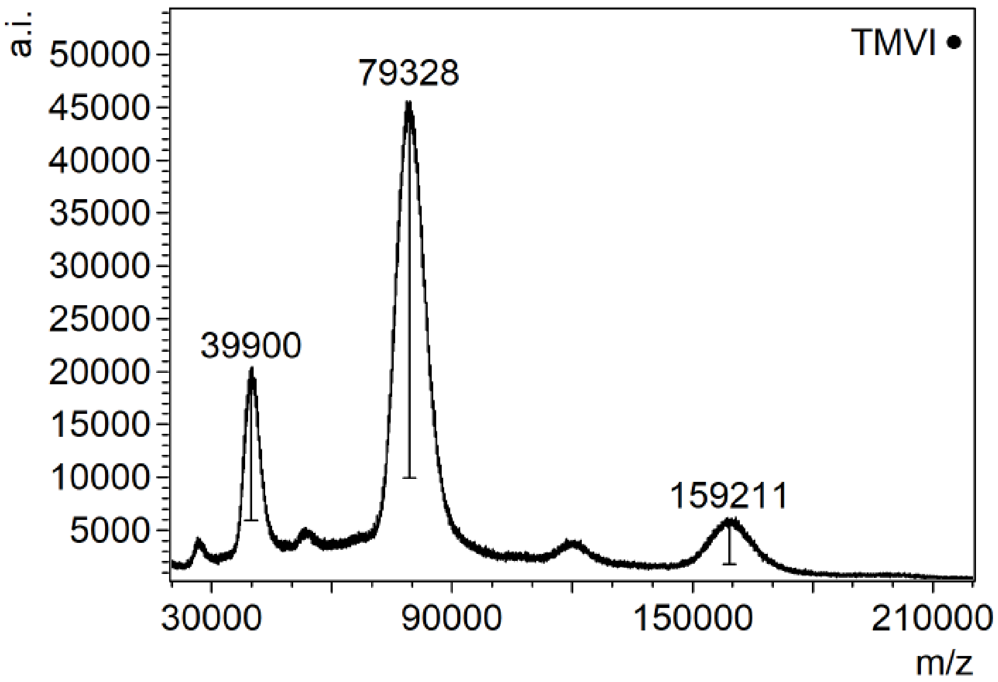

Figure S 31: MALDI-ToF-spectrum of capped, Man_3-functionalized HSA (conjugate TM VI). Found *m/z* = 79328 $[M+H]^+$.

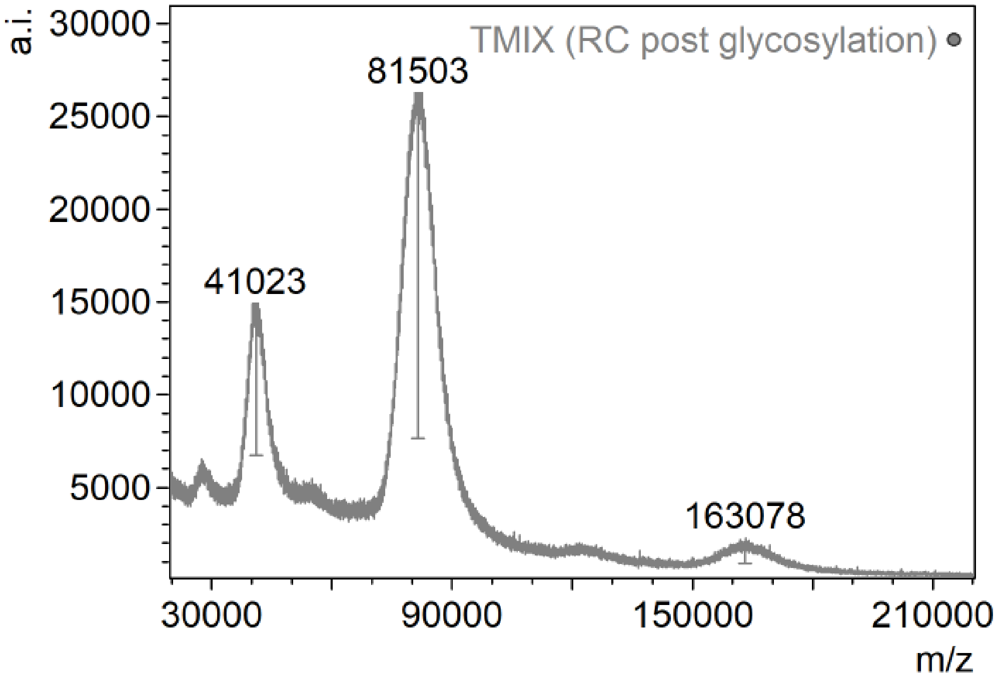

Figure S 32: MALDI-ToF-spectrum as reaction control (RC) of Man_3-functionalized HSA after glycosylation. Found *m/z* = 81503 $[M+H]^+$.

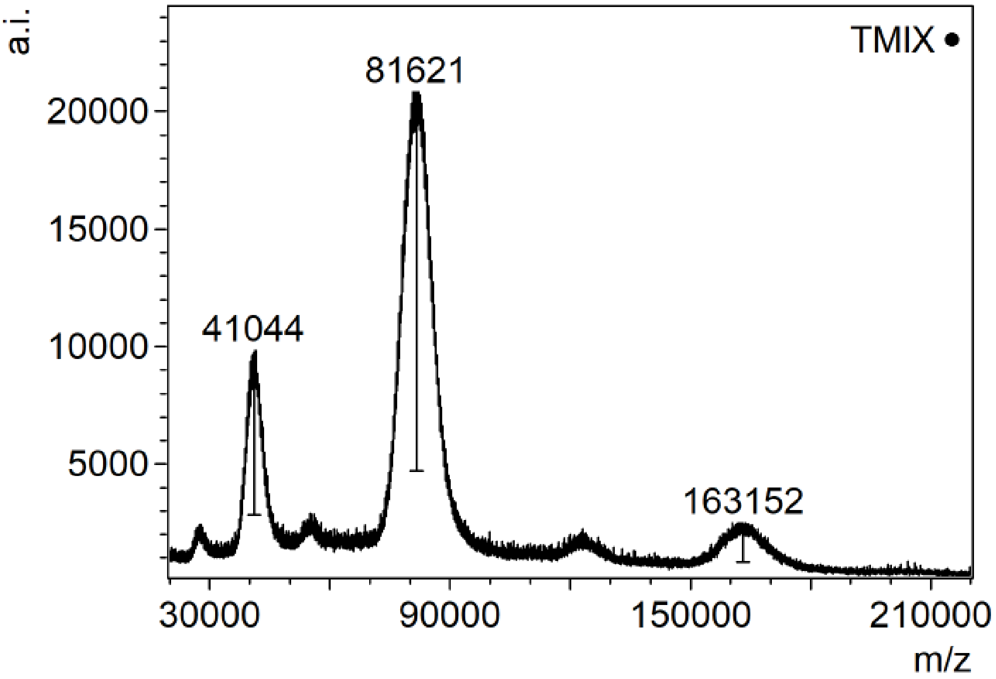

Figure S 33: MALDI-ToF-spectrum of capped, Man_3-functionalized HSA (conjugate TM IX). Found *m/z* = 81621 $[M+H]^+$.

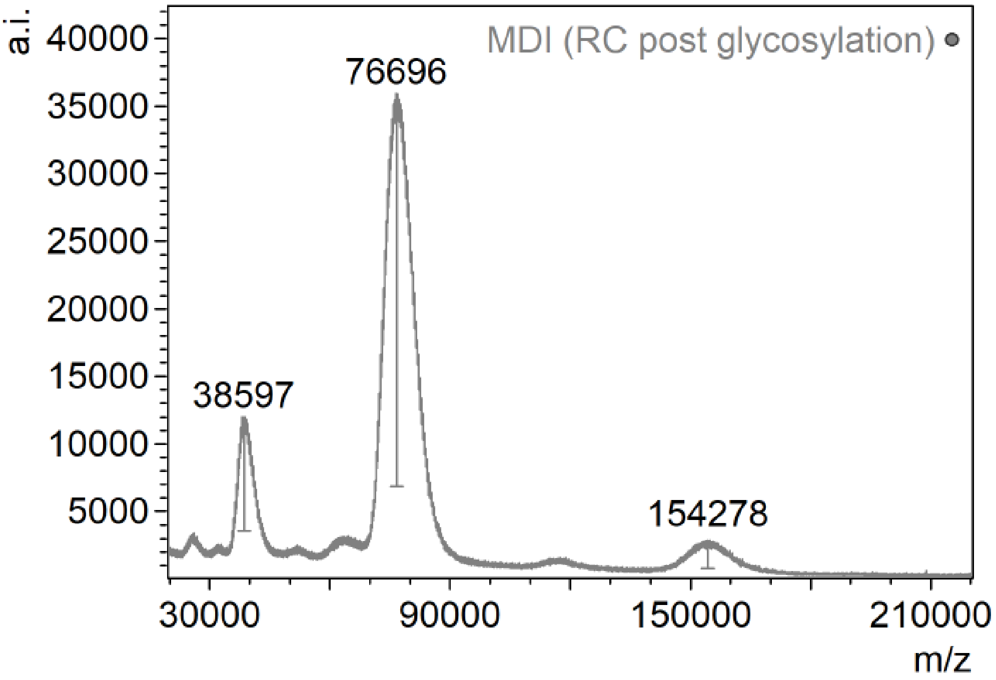

Figure S 34: MALDI-ToF-spectrum as reaction control (RC) of $(Man)_3$-functionalized HSA after glycosylation. Found *m/z* = 76696 $[M+H]^+$.

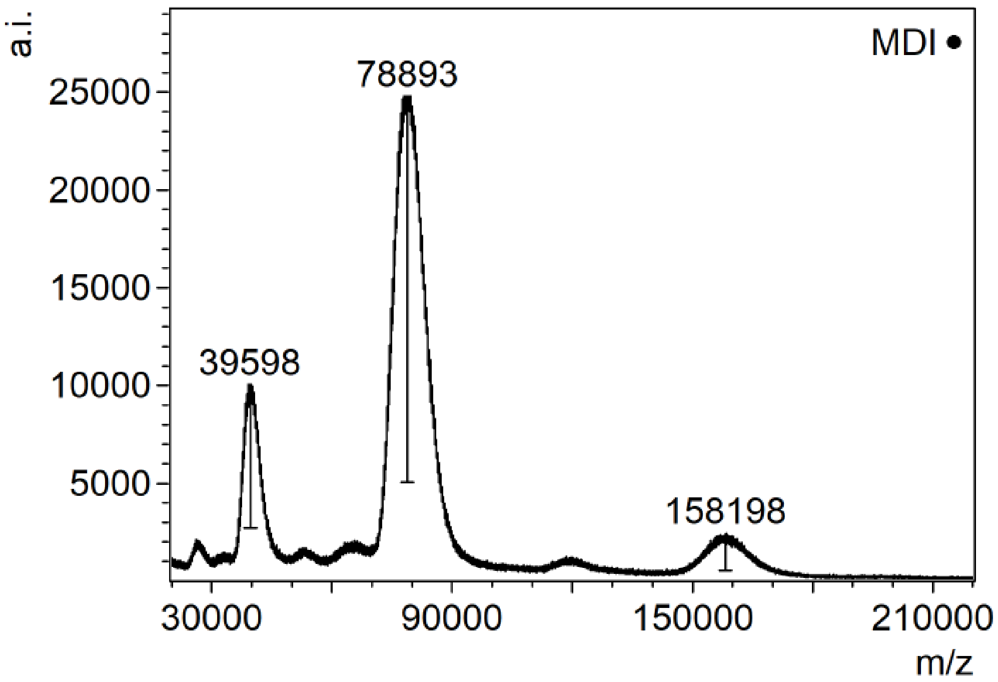

Figure S 35: MALDI-ToF-spectrum of capped, $(Man)_3$-functionalized HSA (conjugate MD I). Found *m/z* = 76340 $[M+H]^+$.

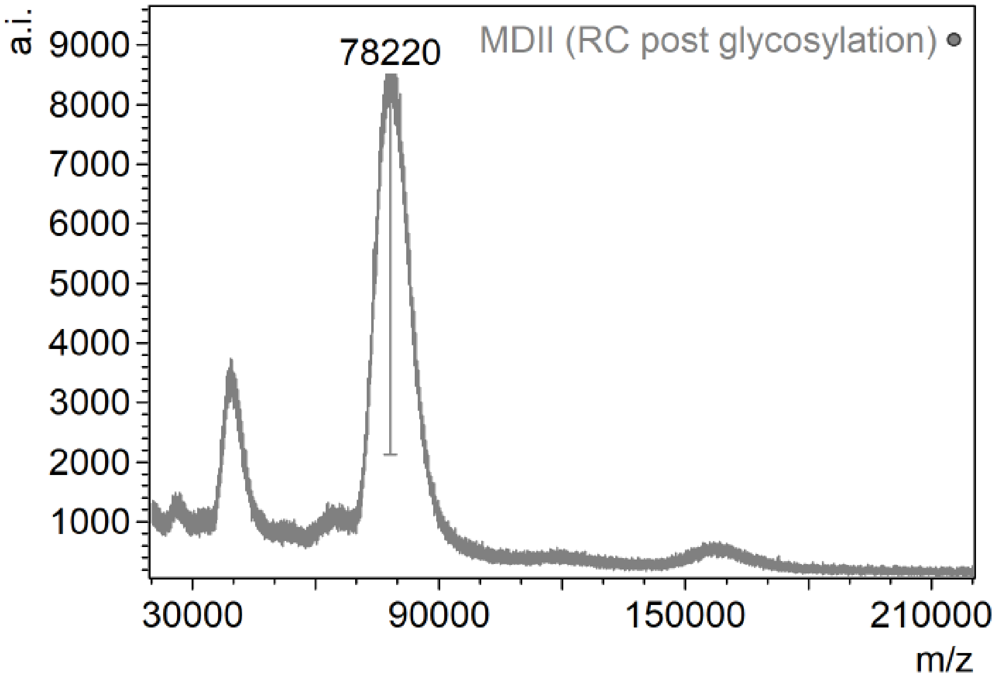

Figure S 36: MALDI-ToF-spectrum as reaction control (RC) of $(Man)_3$-functionalized HSA after glycosylation. Found *m/z* = 78220 $[M+H]^+$.

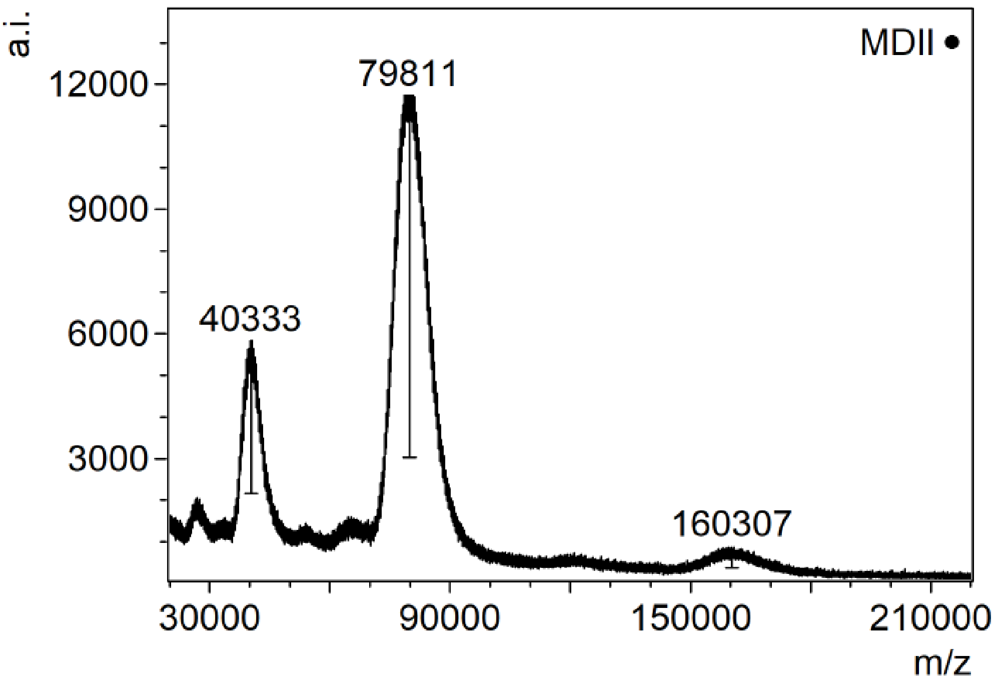

Figure S 37: MALDI-ToF-spectrum of capped, $(Man)_3$-functionalized HSA (conjugate MD II). Found *m/z* = 79811 $[M+H]^+$.

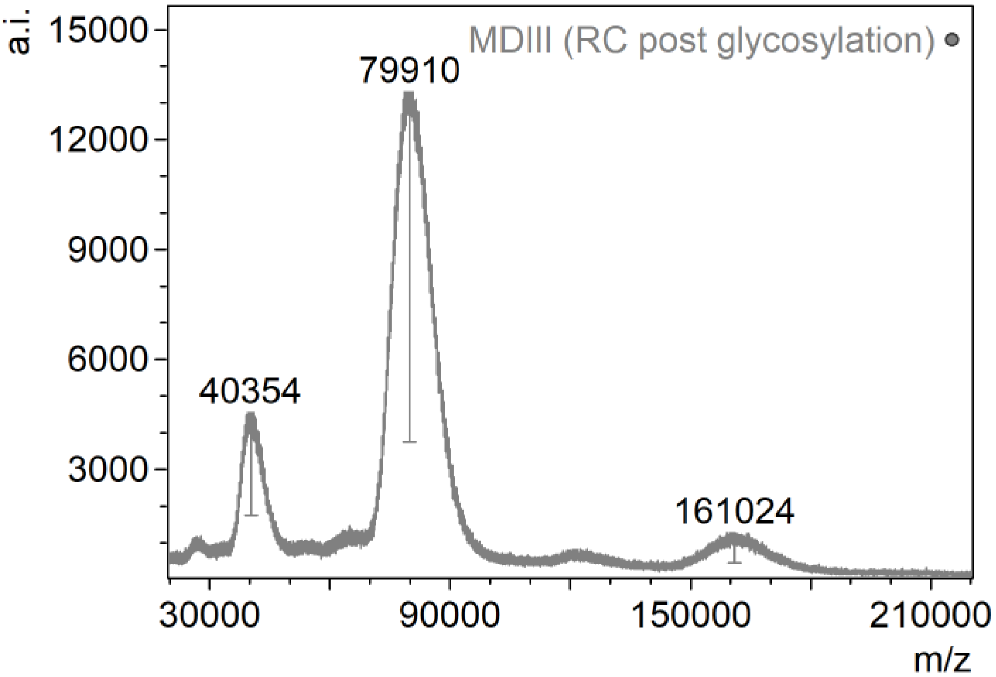

Figure S 38: MALDI-ToF-spectrum as reaction control (RC) of $(Man)_3$-functionalized HSA after glycosylation. Found *m/z* = 79910 $[M+H]^+$.

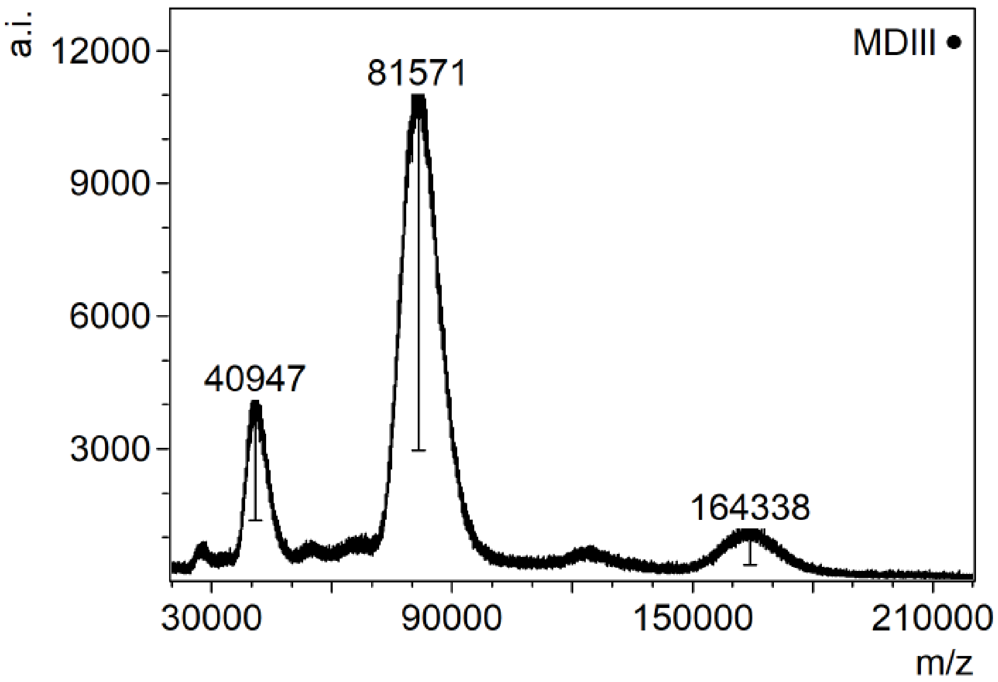

Figure S 39: MALDI-ToF-spectrum of capped, $(Man)_3$-functionalized HSA (conjugate MD I). Found *m/z* = 81571 $[M+H]^+$.

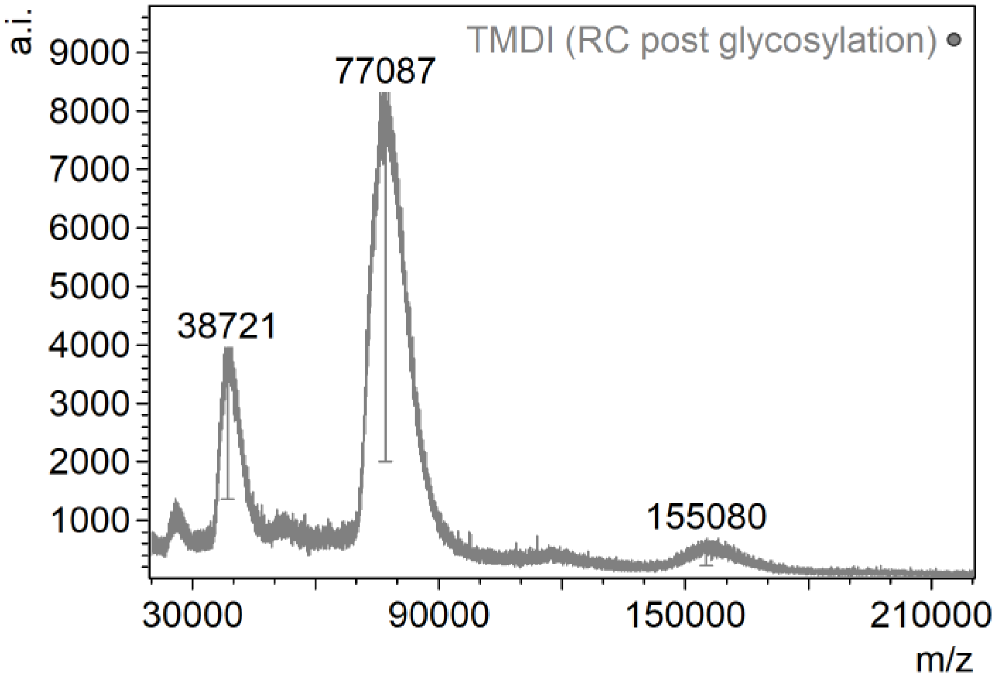

Figure S 40: MALDI-ToF-spectrum as reaction control (RC) of $(Man_3)_3$-functionalized HSA after glycosylation. Found *m/z* = 77087 $[M+H]^+$.

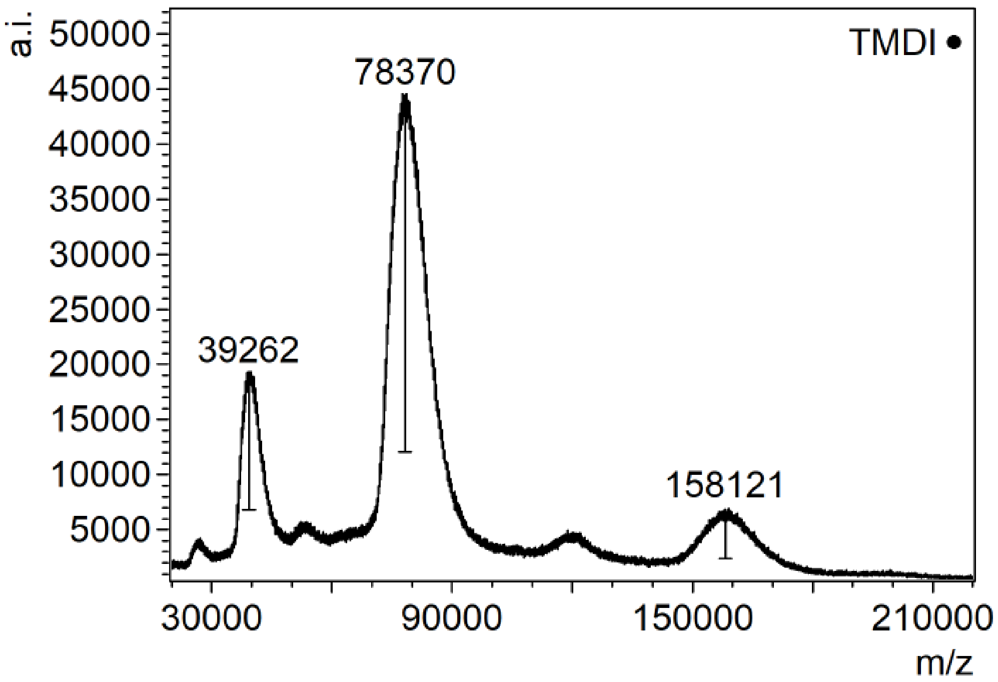

Figure S 41: MALDI-ToF-spectrum of capped, $(Man_3)_3$-functionalized HSA (conjugate TMD I). Found *m/z* = 78370 $[M+H]^+$.

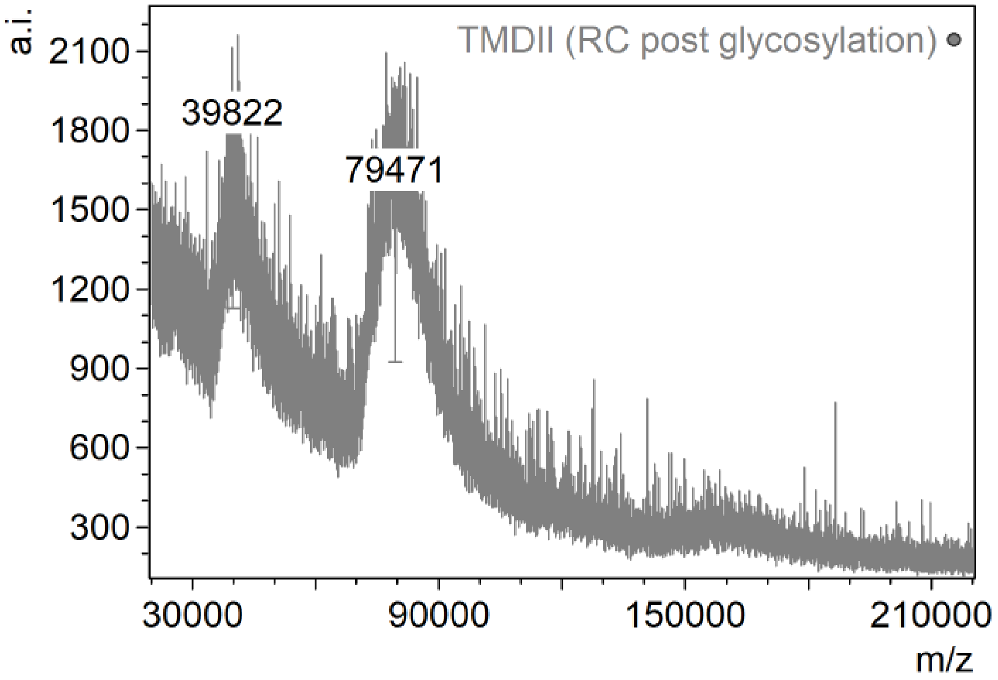

Figure S 42: MALDI-ToF-spectrum as reaction control (RC) of $(Man_3)_3$-functionalized HSA after glycosylation. Found *m/z* = 79471 $[M+H]^+$.

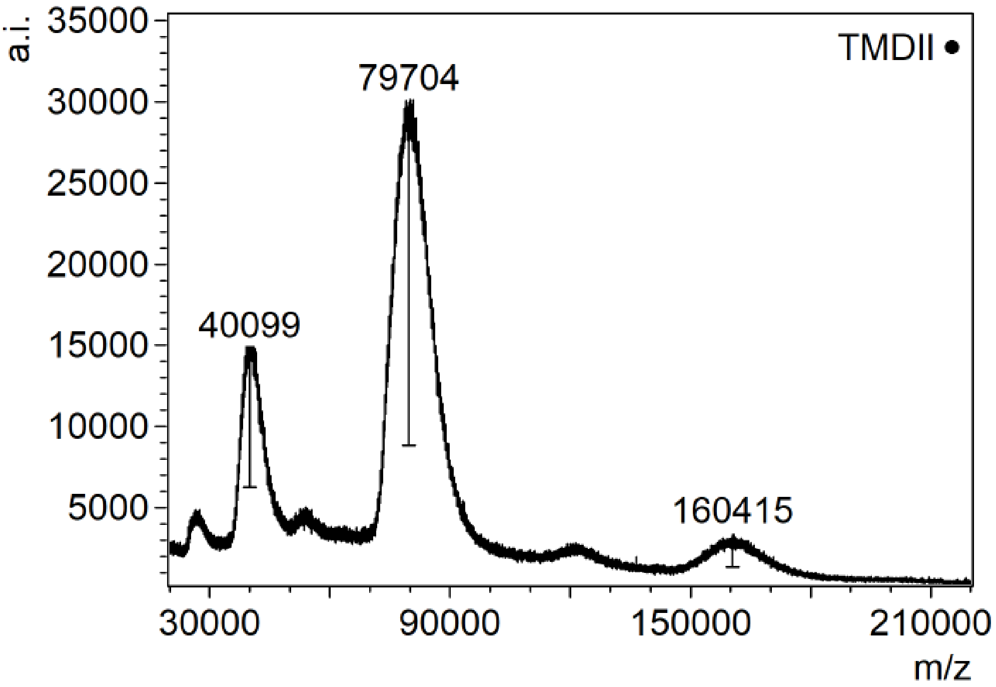

Figure S 43: MALDI-ToF-spectrum of capped, $(Man_3)_3$-functionalized HSA (conjugate TMD II). Found *m/z* = 79704 $[M+H]^+$.

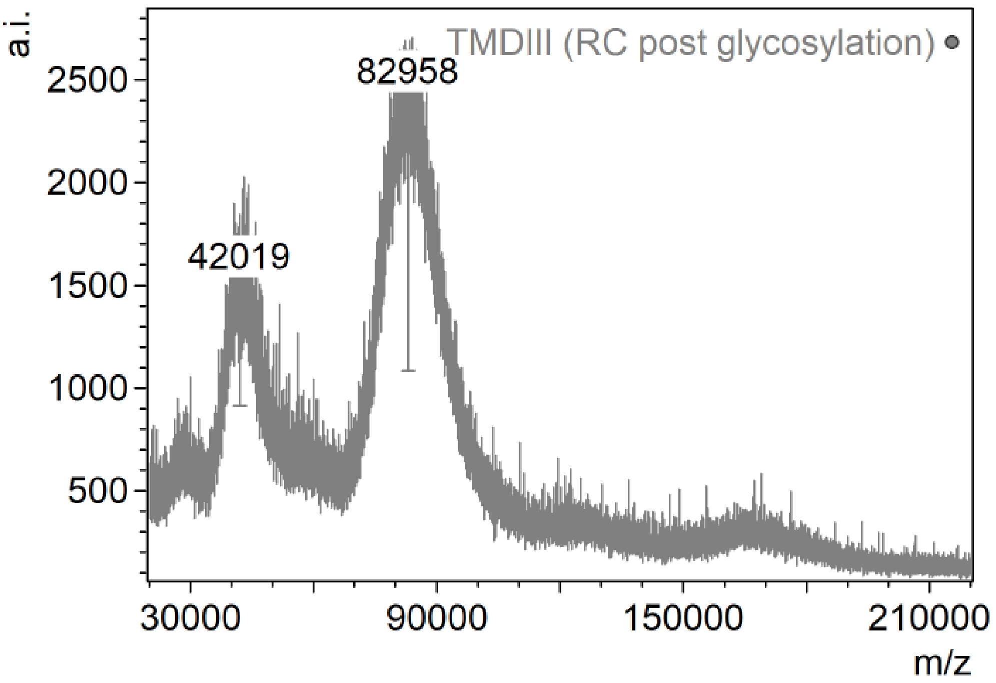

Figure S 44: MALDI-ToF-spectrum as reaction control (RC) of $(Man_3)_3$-functionalized HSA after glycosylation. Found *m/z* = 82958 $[M+H]^+$.

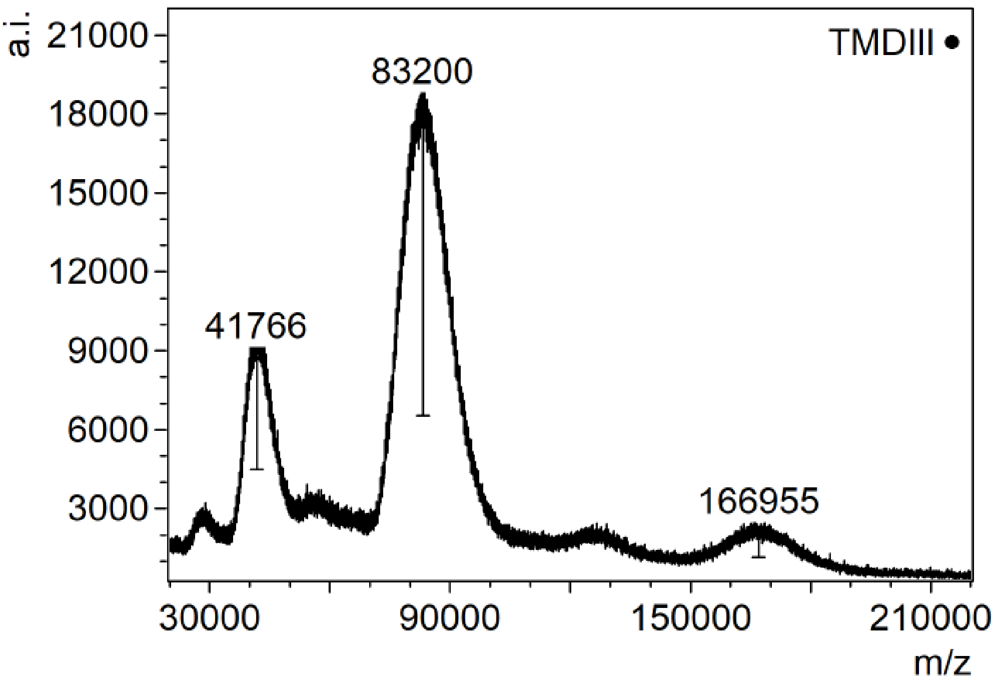

Figure S 45: MALDI-ToF-spectrum of capped, $(Man_3)_3$-functionalized HSA (conjugate TMD III). Found *m/z* = 83200 $[M+H]^+$.

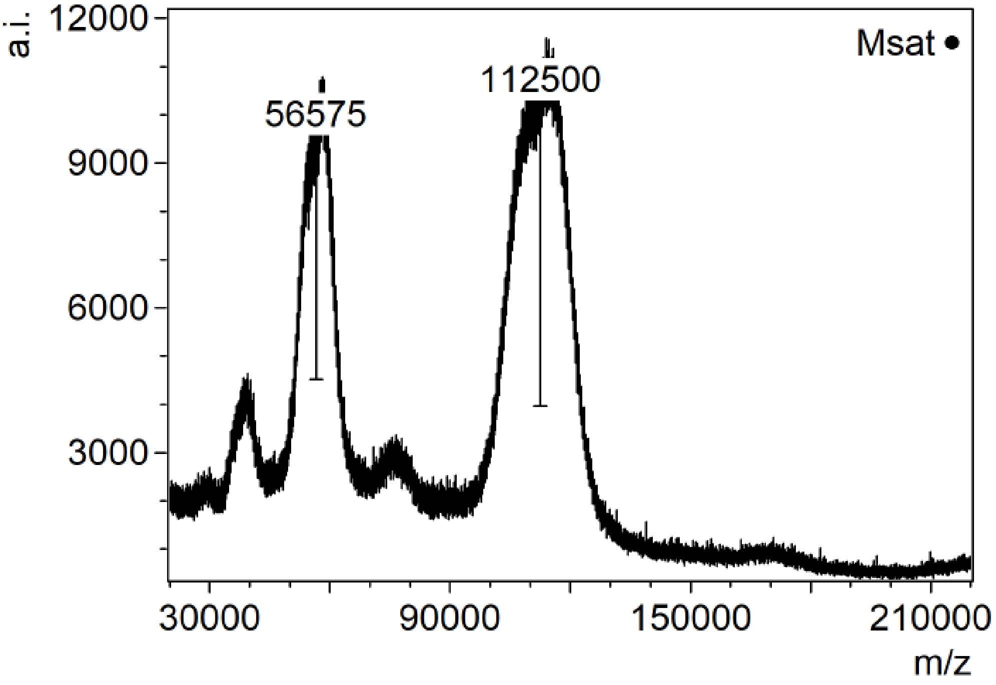

Figure S 46: MALDI-ToF-spectrum of saturated Man-functionalized HSA (conjugate M_{sat}). Found *m/z* = 112500 $[M+H]^+$.

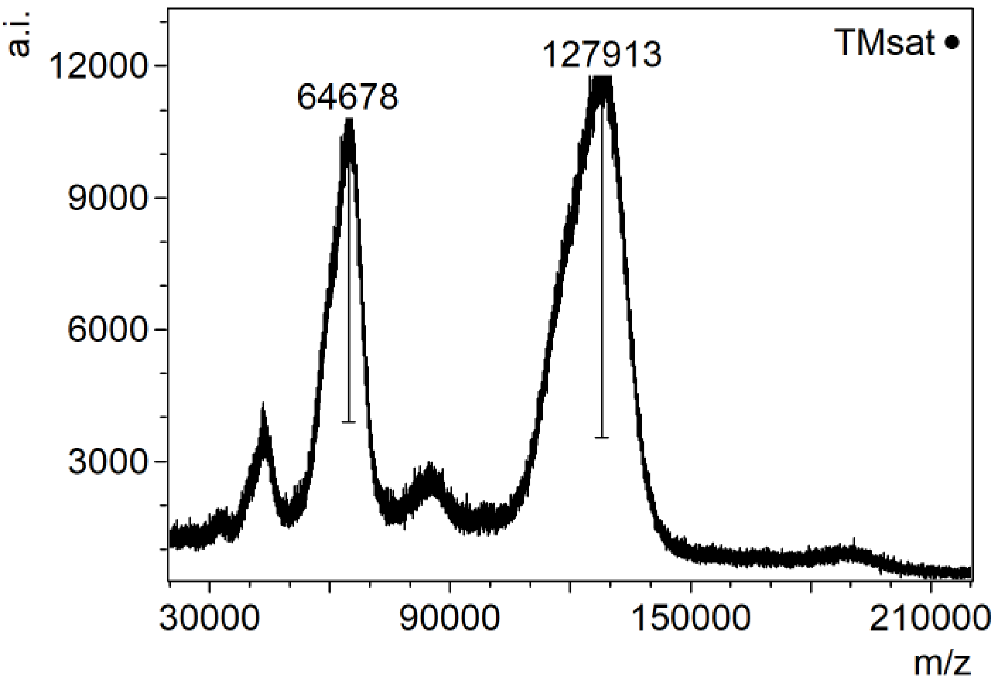

Figure S 47: MALDI-ToF-spectrum of saturated Man_3-functionalized HSA (conjugate TM_{sat}). Found *m/z* = 127913 $[M+H]^+$.

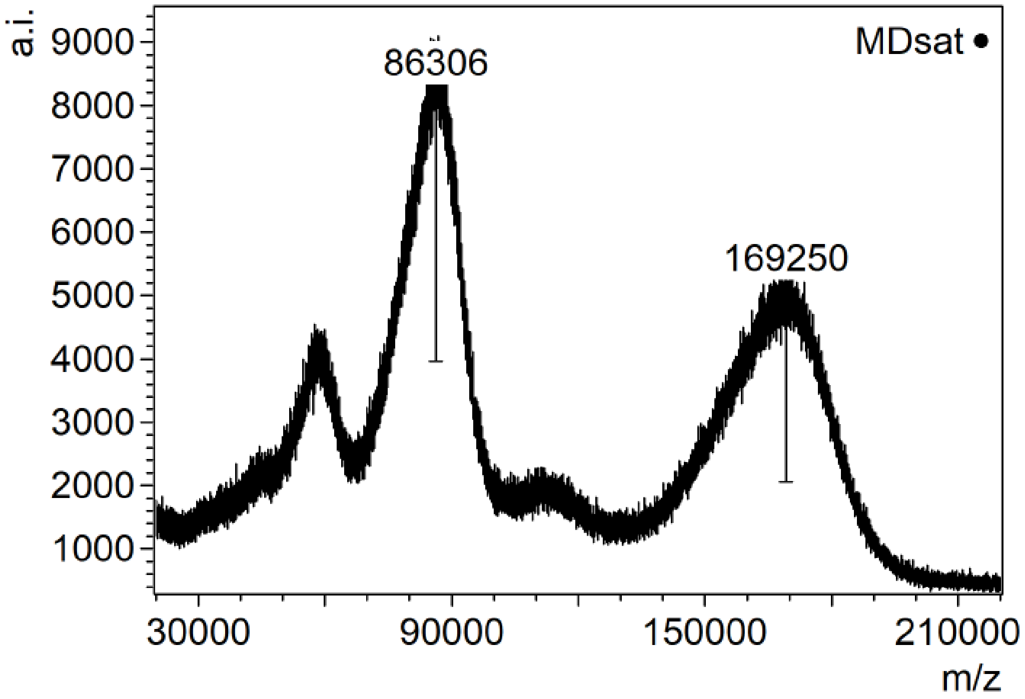

Figure S 48: MALDI-ToF-spectrum of saturated $(Man)_3$-functionalized HSA (conjugate MD_{sat}). Found *m/z* = 169250 $[M+H]^+$.

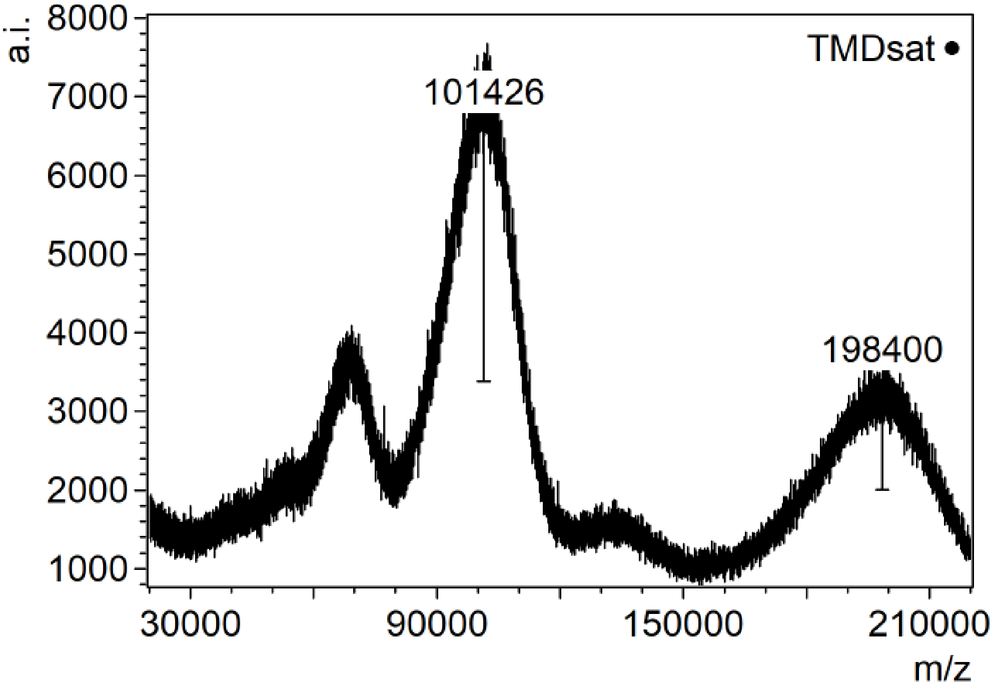

Figure S 49: MALDI-ToF-spectrum of saturated $(Man_3)_3$-functionalized HSA (conjugate TMD_{sat}). Found *m/z* = 198400 $[M+H]^+$.
